# Supplementary material for: Characterization of the VHH-Fc construct rimteravimab in healthy adults and patients hospitalized for mild-to-moderate COVID-19: Two Phase 1 randomized clinical trials
Source: PLoS Med. 2026 May 29;23(5):e1004609. doi: 10.1371/journal.pmed.1004609 (PMC13221044; doi:10.1371/journal.pmed.1004609)
Supplement: S1 Appendix — (PDF) [file pmed.1004609.s001.pdf]

S1 APPENDIX

Introduction

XVR011 was able to neutralize the D614G and Delta variant with IC_{50} values between 13.19- 38.26 ng/mL. However, for the Omicron BA.1 variant, ± 250-fold reduction in IC_{50} value was observed for XVR011 compared to D614G variant, while there was no detectable neutralization against the Omicron BA.2 variant (Table S1).

Table A: Neutralisation of authentic SARS-CoV-2 virus (D614G, Delta, Omicron BA.1 and Omicron BA.2) determined by microneutralisation method.

**Geometric mean IC_{50} (ng/mL)**	**XVR011**	**S309**
D614G	38,26	310,66
Delta	13,19	113,96
Omicron BA.1	9816,01	605,22
Omicron BA.2	ND	5851,25

Data are presented as geometric mean IC_{50} based on 3 independent experiments. ND: no neutralization detected up to 10 µg/mL. IC_{50}: 50% inhibitory concentration.

Methods

Study Design

EXEVIR0102 (EudraCT number: 2021-003707-17; Figure A)

Healthy volunteers were recruited through advertisement by PRA Healthcare (ad on website, mailing to pool of potential participants). Patients were compensated for their participation. Screening assessments were performed within 21 days prior to study-drug administration. Eligible volunteers were enrolled in 1 of 3 sequential dose cohorts (Cohorts 1 [250 mg], 2 [500 mg], and 3 [1000 mg]) and were dosed according to a sentinel dosing design to ensure safety (10 volunteers per cohort, the 2 first in each cohort being sentinel participants; 1 receiving XVR011 and 1 receiving placebo in parallel). In each cohort, if 24 hours after dosing of the 2 sentinels no stopping criteria were met and safety and tolerability were acceptable to the investigator, the remaining 8 volunteers (7 active and 1 placebo) in that cohort were dosed. Dosing in the next dose cohort could only start if no stopping criteria were met and after all available safety and PK data from the preceding dose cohort(s) were reviewed by the investigator and sponsor, and if deemed necessary, by the independent ethics committee.

After obtaining informed consent, subjects received a screening number and were screened according to the inclusion and exclusion criteria. Subjects who met all inclusion criteria and none of the exclusion criteria received a subject number upon inclusion in the study (Subject Numbers 01 to 30). They received the subject number just prior to dosing according to the randomization code (see Appendix 16.1.7). The subject number ensured identification throughout the study after study drug administration. Replacement subjects were to receive the number of the subject to be replaced, increased by 100 (eg, 101 would be the replacement number for Subject 01), and were to be administered the same treatment. Subjects were assigned to a dose group based on their availability while trying to admit subjects with equal sex distribution across dose groups. Treatments within a dose group were assigned according to the randomization code. Sentinel dosing was applied in each group. For the 2 sentinel subjects, randomization ensured that 1 subject received XVR011 and the other subject received placebo. For the remaining 8 subjects in each group, randomization ensured that 7 subjects received XVR011 and 1 subject received placebo. The randomization code was produced by the Biostatistics Department of PRA. The PRA study biostatistician created a draft randomization list and a peer biostatistician reviewed and approved that draft list. The final randomization list was created, reviewed, and approved by 2 designated biostatisticians who were not members of the study team. After the final randomization list was approved, it was transferred

to the PRA Pharmacy and kept in a restricted area to which only the PRA Pharmacy staff had access. 2 unblinded biostatisticians also had access to the randomization list. Code break envelopes were prepared and made available to the clinical staff for emergency unblinding.

Volunteers were admitted at the study site on Day -1. On Day 1, volunteers were randomized and given XVR011 or placebo as a single IV infusion under supervision of the investigator or designee. Volunteers were closely monitored during and until 2 hours after the infusion and discharged approximately 24 hours after the infusion (Day 2). They returned to the study site for study assessments during ambulatory visits on Days 8, 15, 29, 43, 57, and 85 (end of study, Figure S1). In each dose cohort, simple randomization was applied, namely pure randomization based on a single allocation ratio as follows: randomizing sentinel participants in a 1:1 (XVR011: placebo) ratio and non-sentinel participants in a 7:1 ratio (7 active and 1 placebo).

In the event that stopping rules were met in the parallel patient study EXEVIR0101, the Investigator and site team for the healthy individual study EXEVIR0102 were to be notified immediately. Further study drug administration would be suspended for Study EXEVIR0102 pending the recommendation of the independent data monitoring committee or data safety monitoring board (IDMC, see below) for Study EXEVIR0101 on whether or not to stop further treatment and/or enrolment. There was no formal IDMC established for EXEVRI0102.

EXEVIR0101 (NCT04884295; Figure A)

Patients were recruited through the hospitals associated to the study sites. They were not compensated for their participation. Patients were hospitalized on Day -1 and underwent screening assessments preferably within 24 hours of admission. In alignment with the Phase 1a study, eligible patients were enrolled in 1 of 3 sequential dose cohorts (Cohorts 1 [250 mg], 2 [500 mg], and 3 [1000 mg]) and a sentinel dosing design was applied (9 patients per cohort, the first 2 per cohort being sentinel participants).

An IDMC was established prior to screening of any patients for the study EXEVRI0101. The IDMC consisted of 4 independent experts in the fields of infectious disease, pulmonology/respiratory medicine and/or immunology. The Sponsor was responsible for establishing the IDMC and appointing its members. The role and responsibilities of the IDMC were defined in the IDMC charter and reflected the stopping/suspension rules described in the protocol. Stopping criteria were applied within cohorts, based on sentinel dosing, and for dose escalation to the next cohort. If no stopping criteria were met, the Sponsor performed a review of all available data and prepared a summary and recommendation to be reviewed with the Investigators who had included patients in that cohort and with ExeVir Chief Medical Officer to decide on progression to the next dose cohort. If stopping

criteria were met, the IDMC was responsible for data review and making the recommendation to the Sponsor on progression to the next dose cohort. At pre-specified time points, the IDMC also did a formal review of available data from EXEVIR0101 (and EXEVIR0102) and made a recommendation with supporting rationale to the Sponsor who was to make final decisions.

Pharmacokinetic Assessment

The experimental procedure was as follows. Briefly, MSD Streptavidin 96-well plates were blocked with blocking buffer. Following blocking incubation, the blocking buffer was tapped out of the plate. The plate was then coated with the biotinylated anti-capture antibody (BSN.14400.rblgG.114) and incubated for 1 hour at room temperature (RT, set to 22°C) under shaking conditions (450 rpm). After washing, samples, calibrators and controls were added to the wells and incubated for 1 hour at RT under shaking conditions (450 rpm). The plate was washed again and sulfo-tagged detection antibody (anti-ID BSN.14402.rblgG.112) was added. After 1 hour incubation under shaking conditions (450 rpm), the plate was washed and MSD Read buffer was added and the plate was analysed using an MSD Sector Imager 600. The amount of light generated (Relative Light Units, RLU) was directly proportional to the amount of XVR011 in the sample/standard. The XVR011 concentration of the unknown specimen was determined by back calculating the emitted amount of light from the sample from the standard curve.

Immunogenicity Assessment

The experimental procedure was as follows. Briefly, on Day 1 samples were diluted in TBS containing casein, followed by an additional dilution with HBR-3. These samples were incubated for 30 minutes (no shaking) at RT to block out non-specific binding. The diluted samples were then loaded onto a new plate, followed by the addition of Glycine (pH 2.5) and incubated for 30 minutes, (shaking at 450 rpm, at RT). Next, 1 M Trizma base (pH 9.5) was added onto the acidified samples and samples were incubated for 5 minutes at RT (shaking at 450 rpm). The biotinylated drug was prepared and added to the samples (incubated overnight in a 22°C incubator, shaking at 300 rpm). On Day 2, a streptawell high bind streptavidin plate was blocked with casein in TBS buffer for 30 minutes at RT (no shaking). Samples were thereafter added to the streptawell plate and incubated for 1 hour at RT (shaking at 450 rpm). The streptawell plate was washed and 20 mM Glycine (pH 2.5) was added to the plate to elute the anti-XVR011 antibodies. The acidified samples were then added to the MSD plate, and incubated at 450 rpm, for 1 hour at RT. The MSD plate was washed and blocked with casein in TBS and incubated (while shaking at 450 rpm) for 1 hour at RT. A detection reagent was prepared containing sulfo-tag labelled drug at 0.25 µg/mL. In the confirmatory assay, unlabelled drug at 10

µg/mL was incubated with the sulfo-tag labelled drug. Competition of unlabelled drug with labelled drug should inhibit the assay responses of samples with anti-XVR011 antibodies. The MSD plate was washed, and the detection reagent was added to the washed MSD plate and incubated (shaking at 450 rpm) for 1 hour at RT. Finally, the plate was washed, MSD Read buffer GOLD was added and read on the MSD plate reader. The intensity of the chemiluminescence was proportional to the amount of anti-XVR011 present in the sample.

Statistical Analysis

For Phase 1a study, the PK Set included all volunteers in the Safety Set for whom sufficient bioanalytical assessment results were available to calculate reliable estimates of the PK parameters. For Phase 1b study, the PK Set included all patients in the Safety Set with at least 1 drug concentration value. All XVR011 serum concentrations and PK parameters were summarized using descriptive statistics. In Phase 1a study (healthy volunteers), the PK parameters C_{max}, $AUC_{0\text{-}t}$, and $AUC_{0\text{-}inf}$ for XVR011 were compared across dose cohorts to assess dose-proportionality using a power model with mixed effects. The estimate of the slope was reported along with its 95% confidence interval (CI) and associated p-value. To describe the PK variability between participants, and to facilitate the design of future studies, a provisional non-linear mixed effects population PK modelling analysis was carried out on the pooled dataset from the 2 studies. Standard PK models were fitted to the data using the mixed effects modelling software NONMEM v7.5.0 (Beal et al, 1989-2020), and different variability models were explored. Models were selected using diagnostic plots, including visual predictive checks, and alternative models were tested assuming objective function changes for hierarchical models follow a χ^2 distribution. Effects of study and/or population were explored on Clearance (CL), volumes of distribution and on the residual error. The effects of study or health status (healthy individual versus COVID-19 patient) on the PK parameters were also tested.

Neutralization assay using authentic SARS-CoV-2 virus

SARS-CoV-2 viruses belonging to different lineages (D614G, Delta, Omicron BA.1 and Omicron BA.2) were isolated from nasopharyngeal swabs taken from patients/travelers between January 2020 and July 2022. The following clinical isolates were used: SARS-CoV-2 Isolate BavPat1/2020/Germany (09 Feb 2020); Delta variant SARS-Related Coronavirus 2, Isolate hCoV- 19/USA/MD-HP05647/2021; Omicron BA.1 variant SARS-Co V-2 hCoV-19/Netherlands/NH-RIVM-72291/2021; and Omicron BA.2 variant Clinical isolate hCoV-19/Netherlands/VCB-20220303-1/2022. Dose-dependent neutralization of XVR011, the positive control (S309) and a negative control (isotype control) were assessed in an authentic virus neutralization assay. For each variant, three independent runs were performed.

Different system controls were included in the assay: cell only (medium only), virus only, and an internal positive control (human serum). Briefly, 5-fold serial dilutions of XVR011 and controls were incubated with a fixed amount of plaque-forming units (PFUs) of the virus for 1 hour at room temperature. Afterwards, the Vero E6 cell monolayer was inoculated with virus antibody mixtures for 1 hour at 37°C.

In a next step, the inoculum was removed and cells were incubated at 37°C with infection medium (up to 18-24 hours post-infection). Afterwards, the SARS-CoV-2 infected cells were fixed and immunostained with a SARS-CoV Nucleocapsid Antibody (Sino Biological, Catalogue number: 40143-MM05), followed by HRP-conjugated Goat anti-Mouse IgG (H+L) Secondary Antibody (Invitrogen, catalogue number A16072). Spots (infected cells) were counted using an Immunospot Image Analyser.

For each test item, the compound concentration showing 50 % reduction in infection (IC50) was calculated based on the Zielinska method. The geometric mean values were calculated based on three

independent runs.

PK/PD modeling

A model-based drug development approach was used to model human PK and PD data (in terms of XVR011 inhibiting viral activity) to support the dose range finding for the planned clinical studies. The approach to dose determination combines the simulation of human PK profiles of XVR011 with a dose response model based on antiviral efficacy of XVR011 in Syrian hamsters. The dose levels were determined using nonclinical PK data from nonhuman primates to model expected human PK profiles together with a PK/PD model derived from SARS-CoV-2 infection studies in Syrian hamsters. A probabilistic exposure-response model was chosen as it appeared to best describe the data and to be suitable for viral infection description. Using this model, it was possible to determine threshold serum concentrations describing 95% probability of viral reduction. Data from the hamster viral infection studies showed a binary outcome (responder vs non-responder) when using a desired viral load decrease relative to plasma concentrations of XVR011. A logistic regression model was implemented for analysing the PK/PD binary data. The probability to obtain a positive outcome was defined in function of serum concentrations. The relationship between 95 % probability to have a positive viral knockdown and XVR011 concentrations allowed to define a threshold concentration CP95 and its confidence interval.

Rationale for Phase 1a EXEVIR0102 study protocol amendment

The first original version of the protocol was never submitted, so that only amendment 1 was used.

Rationale for Phase 1b EXEVIR0101 study protocol amendments

The following protocol versions have been made available for Part 1 of this study:

Protocol Version Date	Protocol Version No.	Country-specific protocol	Submitted in	Approved in	Comment
18-Dec-2020	1.0	No	NAP	NAP	Created to start up vendor processes and master document creation
19-Jan-2021	2.0	No	Belgium, Italy	Italy	
15-Feb-2021	3.0	No	Belgium	NAP	Created upon comments FAMHP
25-Feb-2021	3.1	No	Belgium, Romania, Italy	Belgium, Romania, Italy	Created upon commitment FAMHP<br><br>INITIAL PROTOCOL
10-Jun-2021	1.0	Yes, Brazil & South Africa	NAP	NAP	
16-Jul-2021	4.0	No	Belgium, Romania, Italy, Moldova	Belgium, Romania, Italy, Moldova	Created to clarify evolving practice and available clinical data of treatment with neutralizing monoclonal antibodies, as well as to facilitate enrolment to the Part 1 inpatients. All (except for one) were enrolled under this protocol

NAP: not applicable; FAMHP: Belgian Federal Agency for Medicines and Health Products.

Study Ethics

The study protocols and informed consent forms (and their amendments) for both studies were approved by responsible local and/or national independent ethics committees (Table S1).

Table B : List of Independent Ethics Committees.

Study	Country	Site	Name and Address of Local Ethics Committee	Name and Address of Central Ethics Committee	Reference number
EXEVIR 0101	Belgium	UZ Gent	Commissie Voor Medische Ethiek UZ Gent - C. Heymanslaan 10, 9000 Gent - Belgium	/	BC-09562
	Belgium	Centre Hospitalier Universitaire de Liège	Comité d'éthique Hospitalo-Facultaire Universitaire de Liège, Domaine Universitaire du Sart Tilman, 4000 Liège - Belgium	/	BC-09562
	Belgium	AZ Sint-Maarten	AZ Sint-Maarten - Commissie	/	BC-09562

			Ethiek vzw Emmaüs - Liersesteenwer 435, 2800 Mechelen - Belgium		
	Moldova	Clinical Republican Hospital “Timofei Mosneaga”	/	Comitetul Național de Expertiză Etică a Studiului Clinic MD 2009 , str. A.Cosmescu, nr. 3, Chișinău, Republica Moldova	1214
	Italy	ASST FBF	COMITATO ETICO MILANO AREA 1 c/o ASST FBF Sacco - P.O. L. Sacco Via G.B. Grassi n. 74 20157 Milano -Italy	COMITATO ETICO DELL'ISTITUTO NAZIONALE PER LE MALATTIE INFETTIVE " LAZZARO SPALLANZANI" IRCCS c/o Direzione Scientifica Via Portuense, 292, 00149 Roma, Italy	341
	Italy	ASST Grande Ospedale Metropolitano Niguarda	COMITATO ETICO MILANO AREA 3 Piazza Ospedale Maggiore 3 - 20162 – Milano - Italy	COMITATO ETICO DELL'ISTITUTO NAZIONALE PER LE MALATTIE INFETTIVE " LAZZARO SPALLANZANI" IRCCS c/o Direzione Scientifica Via Portuense, 292, 00149 Roma, Italy	341
EXEVIR 0102	The Netherlands	PRA Health Sciences – Early Development Services Van Swietenlaan 6 9728 NZ Groningen The Netherlands	/	IEC of the Foundation “Beoordeling Ethiek Biomedisch Onderzoek” (English translation: “Assessment of Ethics of Biomedical Research”) Dr. Nassaulaan 10 9401 HK Assen The Netherlands	NL78439.056.21

Study Design

Figure A: Study Design. PK: parmacokinetics. IV/ intravenous. ADA: anti-drug antibodies.

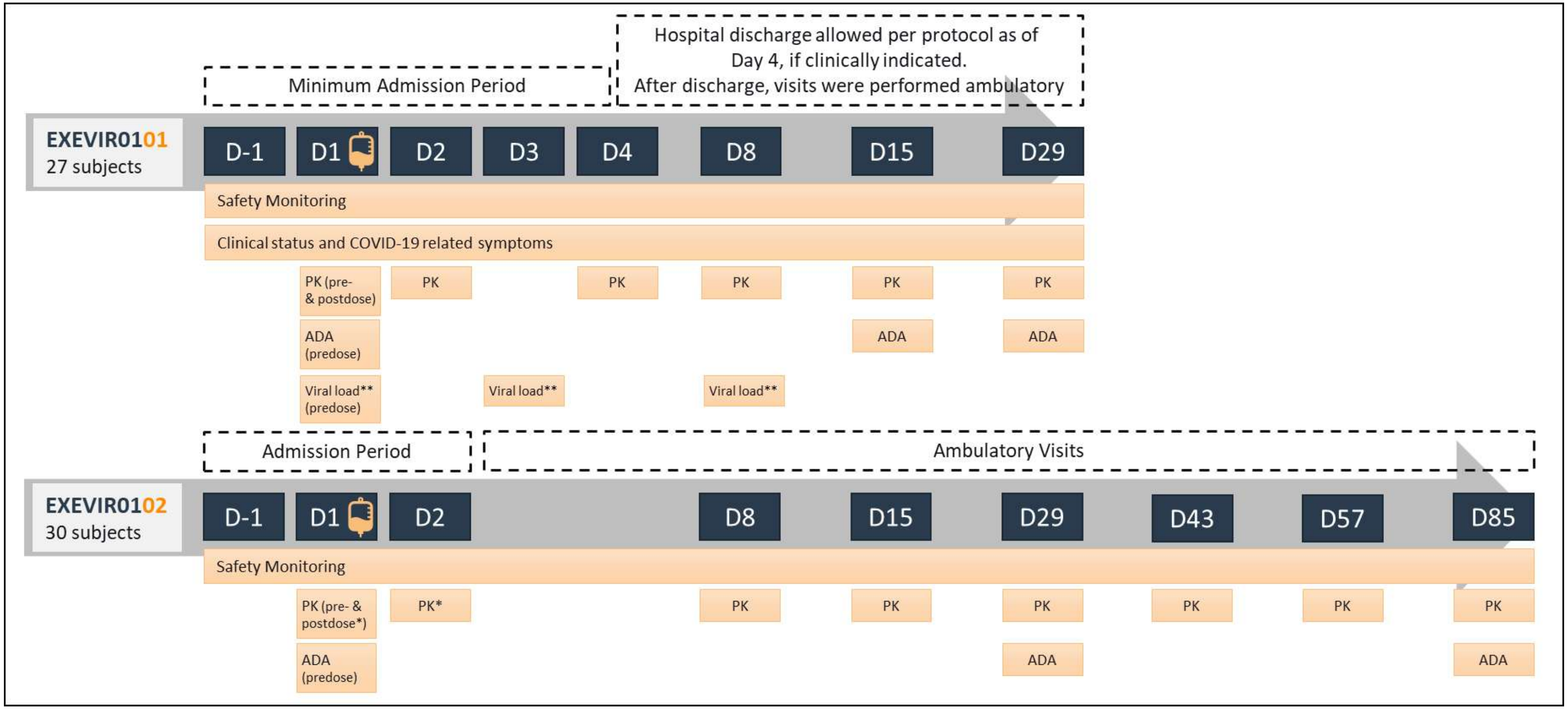

* PK blood sampling at 1.5, 2, 7.5, 11.5, and 25.5 hours after start of IV infusion.
**Nasopharyngeal swabs collected pre-dose, at days 3 and 8 and at discharge.

Results

Safety

Table C: Summary of Treatment-Emergent Adverse Events

System Organ Class<br>Preferred term	Cohort 1<br>250 mg<br>N=8<br>n (%) E	Cohort 2<br>500 mg<br>N=10<br>n (%) E	Cohort 3<br>1000 mg<br>N=9<br>n (%) E	Overall<br><br>N=27<br>n (%) E
**Any TEAE**	2 (25.0) 3	3 (30.0) 9	2 (22.2) 3	7 (25.9) 15
**Investigations**	1 (12.5) 1	1 (10) 2	2 (22.2) 3	4 (14.8) 6
Alanine aminotransferase increased	0	0	1 (11.1) 1	1 (3.7) 1
Aspartate aminotransferase increased	0	0	1 (11.1) 1	1 (3.7) 1
C-reactive protein increased	0	1 (10) 1	0	1 (3.7) 1
Neutrophil count increased	0	1 (10) 1	0	1 (3.7) 1
Platelet count increased	1 (12.5) 1	0	0	1 (3.7) 1
Transaminases increased	0	0	0	1 (3.7) 1
**General disorders and administration site conditions**	1 (12.5) 1	1 (10.0) 1	0	2 (7.4) 2
Chills	1 (12.5) 1	0	0	1 (3.7) 1
Oedema peripheral	0	1 (10.0) 1	0	1 (3.7) 1
**Respiratory, thoracic, and mediastinal disorders**	1 (12.5) 1	1 (10.0) 3	0	2 (7.4) 4
Chronic obstructive pulmonary disease	0	1 (10.0) 1	0	1 (3.7) 1
Hyperventilation	0	1 (10.0) 1	0	1 (3.7) 1
Pulmonary embolism	1 (12.5) 1	0	0	1 (3.7) 1
Wheezing	0	1 (10.0) 1	0	1 (3.7) 1
**Cardiac disorders**	0	1 (10.0) 1	0	1 (3.7) 1
Tachycardia	0	1 (10.0) 1	0	1 (3.7) 1
**Gastrointestinal disorders**	0	1 (10.0) 1	0	1 (3.7) 1
Abdominal pain upper	0	1 (10.0) 1	0	1 (3.7) 1
**Immune system disorders**	0	1 (10.0) 1	0	1 (3.7) 1
Cytokine storm	0	1 (10.0) 1	0	1 (3.7) 1

Abbreviations: E = number of events; N = Number of participants dosed in cohort; n = number of participants with characteristic; TEAE = treatment-emergent adverse event; % = Calculated as (n/N*100).

PK/PD modeling

Based on the probabilistic exposure-response model, the median serum concentrations required for a 95 % probability of being a responder, defined as a 4-fold (log 10) reduction in viral load was 26.9 µg/mL. Based on the 97.5th percentile (in a more conservative approach), the 95 % probability of response was associated with a serum concentration of 50.4 µg/mL (Table D).

Table D: XVR011 serum concentrations vs viral load knockdown, based on modelling results from the hamster studies

Viral Load Knockdown Compared to Median Controls (log10)	Serum Concentration (µg/mL)		
	CP95 (2.5 percentile)	CP95	CP95 (97.5 percentile)
2-fold	11.7	17.3	41.6
3-fold	14.1	20.7	46.4
4-fold	19.1	26.9	50.4

To predict clinical PK and PD outcomes, simulations were performed using the established PK/PD model. However, in order to evaluate interindividual variability of PK and PD outcomes, a coefficient of variation of 30 % was assumed for both CL and V1. At each dose, 200 participants were simulated. Negligible impact of the time of I.V. infusion was assumed. Simulation results for a 70-kg human are shown in Figure 1, overlaid with the threshold concentrations defined by the PK/PD hamster model.

Figure B: Simulated human PD data, overlaid with threshold concentrations from PK/PD hamster. PK: PK: pharmacokinetics; PD: pharmacodynamics; IV: intravenous; CP95: 97.4 percentile **model.**

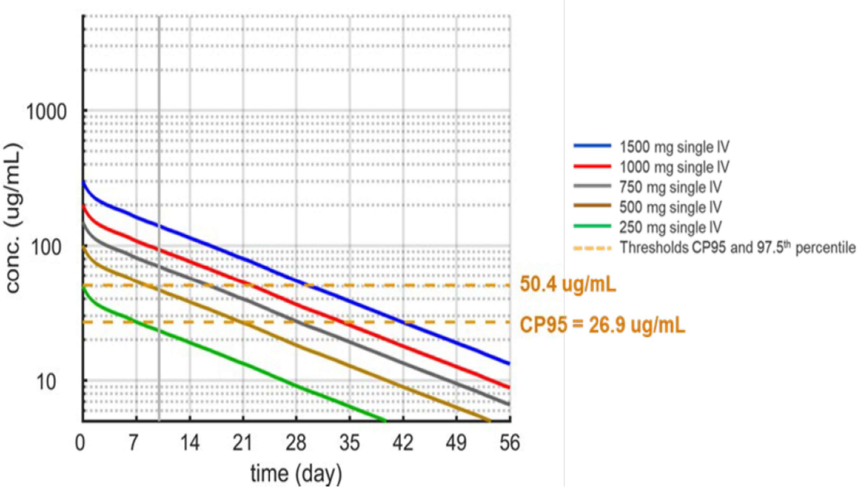

PK simulations taking into account inter-patient PK variability show that XVR011 doses of 250-1000 mg should provide sufficient separation in terms of exposure.

Table E: Key model building steps

Run no	Reference run	Model description	ObjF	dObjF	p-value
243		Same residual error in healthy volunteers and patients	1920.8798		
237	243	Final model	1908.0057	-12.8741	<0.001
238	237	Separate CL healthy volunteers and patients	1907.1565	-0.8492	0.3568
239	237	Separate V_c healthy volunteers and patients	1907.668	-0.3377	0.5612
240	237	Separate V_p healthy volunteers and patients	1904.3196	-3.6861	0.0549

CL=clearance; V_c=volume of central compartment; V_p=volume of peripheral compartment; ObjF=objective function value; dObjF=change in ObjF from reference run

Figure C: Observed vs Predicted concentrations, population PK model.

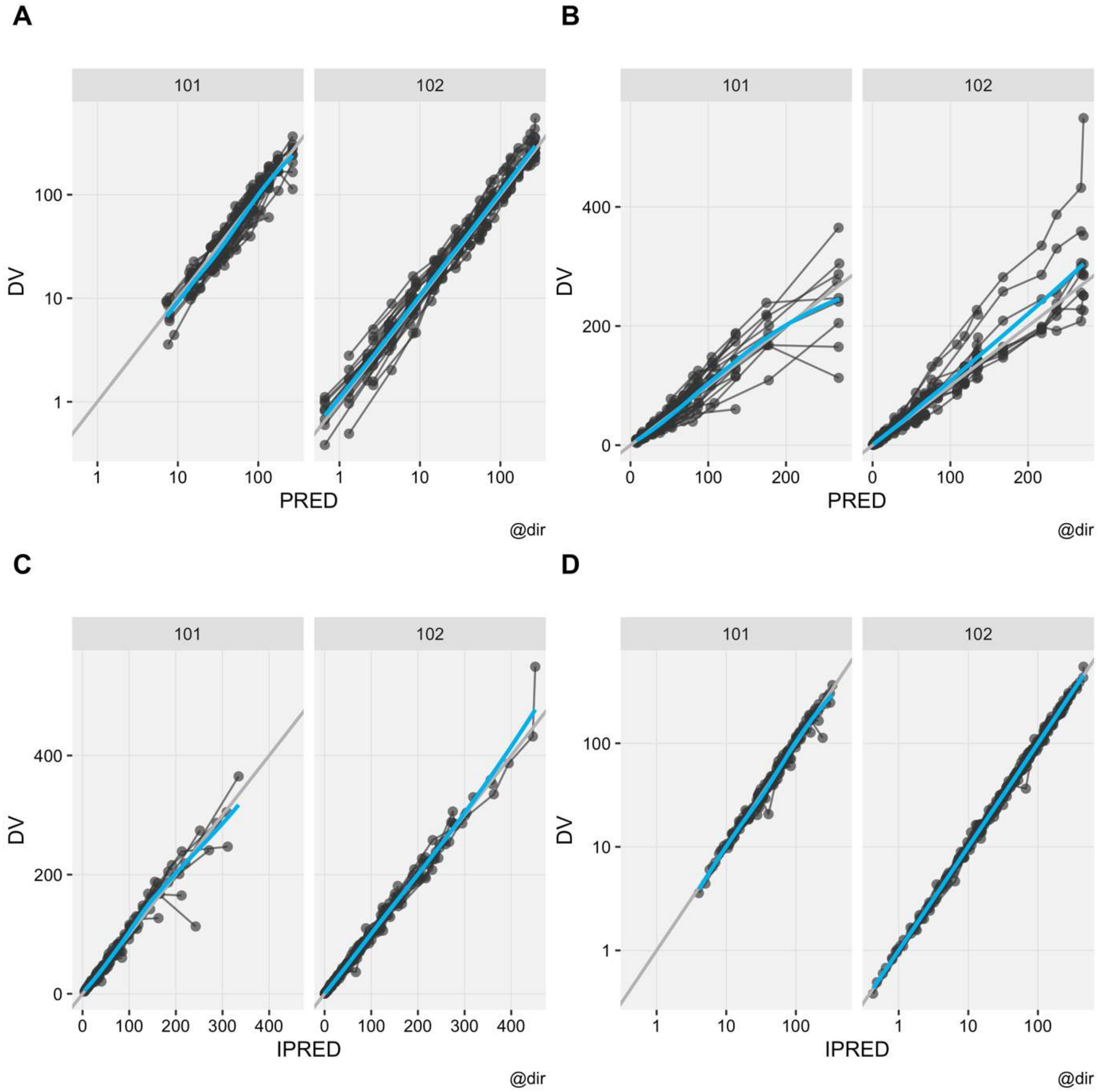

A: Observations (DV) vs population predictions (PRED) on log scale. B : DV vs PRED, linear scale. C: DV vs individual predictions (IPRED), log scale. D: DV vs IPRED, linear scale. Blue lines are loess smooths, grey lines represent unity. 101: EXEVIR101 phase 1b study (hospitalized COVID-19 patients); 102: EXEVIR102 (healthy volunteers phase 1a study).

Figure D: Residuals versus Time and Predictions, population PK model

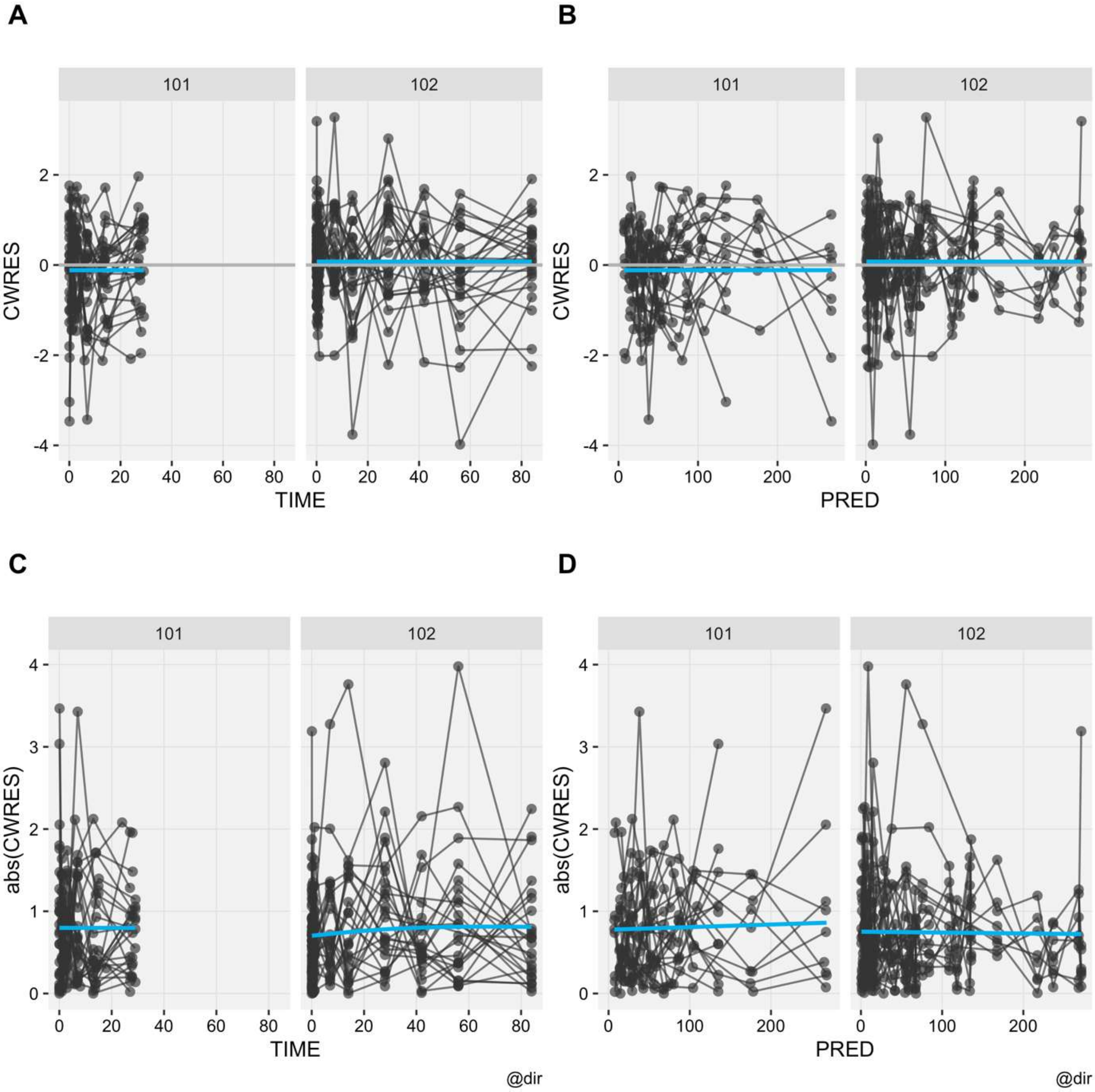

A: Conditional weighted residuals (CWRES) vs time since dosing. B: CWRES vs PRED. C : absolute CWRES vs time since dosing. D : absolute CWRES vs PRED. Blue lines are loess smooths, grey lines represent y=0. PK: pharmacokinetics.

Figure E: Visual predictive check by dose and study, population PK model.

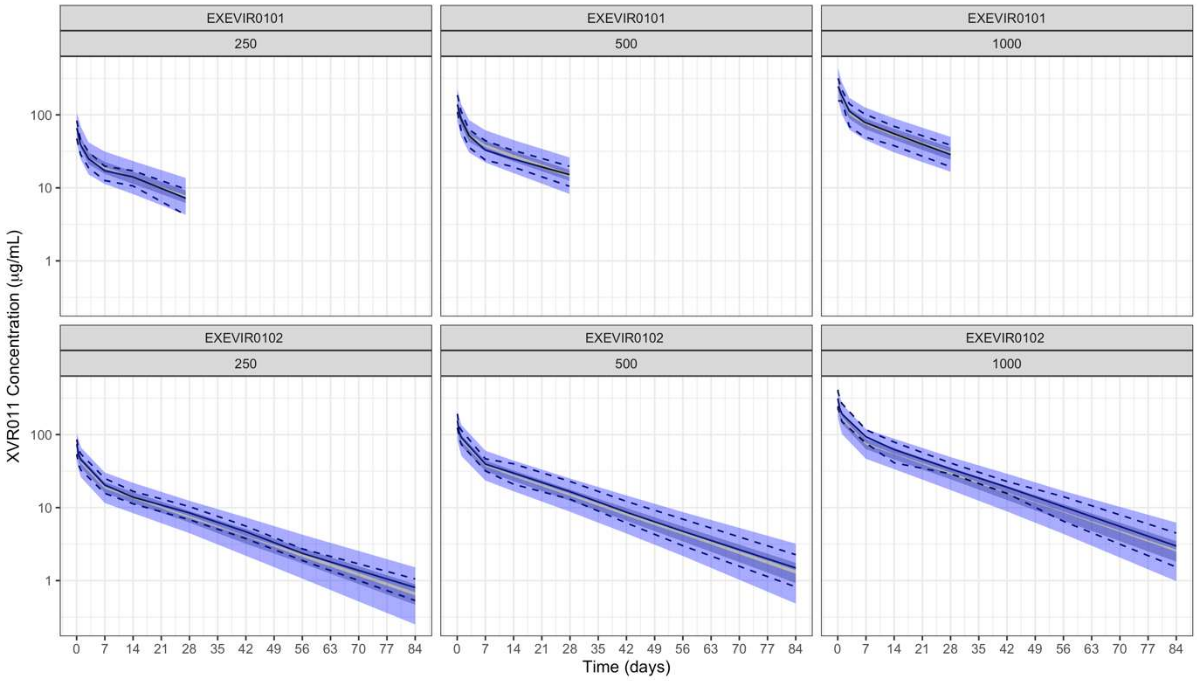

Solid lines show median observed concentrations, dashed lines show 10th and 90th percentiles of observed concentrations. Areas show model predictions: Grey area shows the 95% prediction interval for the median, blue areas show the 95% prediction interval for the 10th and 90th percentile. PK: pharmacokinetics.

Table F: Statistical Analysis of Dose-proportionality of XVR011 in Study EXEVIR0102 (PK Set)

Parameter	Slope	Standard Error	95% CI Lower	95% CI Upper	p-value
C_{max}	1.045	0.082	0.876	1.214	0.5840
AUC_{0-t}	1.049	0.069	0.905	1.194	0.4837
AUC_{0-inf}	1.046	0.070	0.901	1.191	0.5203

Parameters taking value 1 would mean dose proportionality. AUC_{0-inf}=area under the serum concentration-time curve from time 0 to infinity; AUC_{0-t}=area under the serum concentration-time curve from time 0 to time of last quantifiable concentration; CI=confidence interval; C_{max}=maximum serum concentration; 95% CI: 95% confidence interval.

Table G: Time to hospital discharge

Characteristic	Cohort 1 250 mg (N = 9) n (%)	Cohort 2 500 mg (N = 9) n (%)	Cohort 3 1000 mg (N = 9) n (%)	Overall (N = 27) n (%)
Number of Participants Discharged by Day 29 (n [%])	7 (77.8)	9 (100)	8 (88.9)	24 (88.9)
Number of Censored Cases (n [%])	2 (22.2)	0	1 (11.1)	3 (11.1)
Censoring Reasons:				
ICE2: Forbidden Concomitant Medications (n [%])	1 (11.1)	0	1 (11.1)	2 (7.4)
End of Study (n [%])	1 (11.1)	0	0	1 (3.7)
Death (n [%])	0	0	0	0
Treatment Failure (n [%])	0	0	0	0
KM Estimates (95% CI) (days)				
25th percentile	8.0 (5.0; 14.0)	8.0 (8.0; 14.0)	6.0 (5.0; 7.0)	7.0 (5.0; 8.0)
Median	14.0 (5.0; NC)	14.0 (8.0; 14.0)	7.0 (5.0; 8.0)	8.0 (8.0; 14.0)
75th percentile	16.0 (10.0; NC)	14.0 (8.0; NC)	8.0 (7.0; NC)	14.0 (10.0; NC)

Abbreviations: CI = Confidence Interval; IMP = Investigational medicinal product; KM = Kaplan-Meier; N = Number of participants in the cohort; n = number of participants in the specific category; %: Calculated as (n/N*100); NC: not claculated.

ICE2 = participant received other medication alongside IMP, which affects the primary variable

Table H: History of previous SARS-CoV-2 vaccinations in the phase 1b EXEVIR0101 clinical trial.

Dose (mg)	Patients previously vaccinated (n/N (%))	Type of SARS-CoV-2 vaccine received, # doses	Average time since last vaccine dose (d)
250	3/9 (38%)	Comirnaty or Vaxzeria, 2 doses	191
500	8/9 (80%)	Comirnaty, Vaxzeria or Spikevax, 2 doses; Janssen, 1 dose	198
1000	7/9 (78%)	Comirnaty, Vaxzeria or Sinopharm, 2 or 3 doses; Janssen, 1 dose	155

Table I: Individual efficacy outcomes *versus* ADA titers, in EXEVIR 101 phase 1b study in COVID-19 hospitalized patients. A. Overall view; B. Detailed view on time to mild COVID symptoms; C. Detailed view on duration of oxygen supplementation. SaO_2: oxygen saturation of the blood. MRD: minimal required dilution.

A.

Dose (mg)	Subject No	Viral load decrease from Day1 baseline (RT-qPCR in nasopharyngeal swabs)			Time to mild COVID-19 symptoms (d)	Time to COVID-19 symptom resolution (d)	Time to recovery (d)	Time to hospital discharge (d)	Lowest SaO_2 (%)	Duration of oxygen support (d)	ADA titer (negative value replaced by MRD 43)		
		d3	d8	Dischage							d0	d14	d28/29
250	1	-0.6	1	not reported	29	29	29	29	92	19	43	43	43
	2	negative	not reported	2	7	29	29	29	93	6	43	43	43
	3	2.2	not reported	1.7	29	29	4	5	94	36	43	43	43
	4	1.2	not reported	negative	1	30	8	8	96	0	43	43	43
	5	0.4	positive but BQL	negative	2	30	10	10	95	0	43	43	43
	6	0.15	2.6	negative	1	28	5	16	96	0	43	43	43
	7	2.5	negative	negative	1	28	4	14	96	0	43	43	43
	8	1.4	2.4	negative	1	28	3	14	93	0	43	43	43
	9	-0.2	not reported	positive but BQL	1	14	2	8	93	0	43	43	43
500	10	1.1	negative	negative	1	30	4	16	95	0	43	43	43
	11	1.3	2.4	negative	3	28	14	14	97	0	43	60	43
	12	1.6	negative	positive but BQL	2	28	14	14	94	0	43	43	43
	13	0.8	3.4	positive but BQL	8	28	12	14	96	0	292	155	287
	14	-0.8	not reported	positive but BQL	4	16	8	8	96	0	43	43	43
	15	0.5	not reported	positive but BQL	4	30	8	8	95	0	43	43	43
	16	1	1.3	negative	14	27	11	14	93	0	43	553	156
	17	negative	not reported	negative	1	15	8	8	97	0	43	43	43
	18	1.2	not reported	2.1	8	29	8	8	96	0	43	43	43
1000	19	not reported	not reported	not reported	4	22	8	8	96	0	43	43	43
	20	0.6	not reported	1.2	1	15	7	8	95	0	43	215	123
	21	0.2	3.2	1.2	1	16	6	6	95	0	43	43	43
	22	1.4	positive but BQL	positive but BQL	14	29	29	29	95	0	43	258	56
	23	-0.2	3.9	2.3	1	22	8	5	95	0	43	43	43
	24	2.2	not reported	negative	7	15	7	7	97	0	43	43	43
	25	0.2	not reported	1.1	1	16	7	7	95	0	43	43	43
	26	-0.4	not reported	1.7	7	19	7	7	96	0	43	43	43
	27	0.5	negative	positive but BQL	3	29	5	6	96	0	43	43	43

B.

Time to mild COVID symptoms (d)	
ADA+	ADA-
1	1
1	1
2	1
3	1
4	1
8	1
14	1
14	1
29	1
	2
	3
	4
	4
	7
	7
	7
	8
	29

C.

Duration O2 supplementation (d)	
ADA+	ADA-
0	0
0	0
0	0
0	0
0	0
0	0
0	0
0	0

36	0<br>0<br>0<br>0<br>0<br>0<br>0<br>0<br>6<br>19

Table J: Individual medical history at start of study, and associated medication, *versus* ADA titers, in EXEVIR101 phase 1b study in COVID-19 hospitalized patients

Dose (mg)	SubjectNo	Gender	Age (y)	Medical history @ start of study	Concomittant medication resulting from medical history	ADA titer (negative value replaced by MRD 43)		
						d0	d14	d28/29
250	1	M	55	-		43	43	43
	2	F	58	T2D *mild* (since 2003)	Simvastatin 40mg QD + D-cure 25000 IU/wk (since 2011)	43	43	43
	3	F	55	COPD *mild* + migraine *moderate* (since 2007); Hypercholesterolemia *mild* + gastritis *mild* (since 2011); Peripheral arterial occlusive disease *mild* + carotid artery stenosis *mild* (since 2018); Anxiety *mild* + hypertension *mild* (since 2021)	Simvastatin 25mg QD + Acetylcysteine 600mg QD + aspirin 80mg QD + azprazolam 0,5mg PRN + lorazepam 2,5 mg PRN + Trazodone 100mg PRN + Duovent HFA 10mL TID inhalation + Flixotide 250mg PRN inhalation + dexpanthenol 40mg QD + Trimbow 100puff BID (since d-23); Perindopril 5mg QD + valproate sodium/ valproic acid 500mg PRN + prothypendyl HCl 80mg QD (since d-22); Magaldrate 10mL TID (since d-12)	43	**43**	43
	4	F	57	T2D *moderate* (since 2019)		43	43	43
	5	F	74	Hypertension *moderate* (since 2012) chronic cardiac failure *mild* (since 2014); Hypertensive cardiomyopathy *mild* (since 2015); Osteoporosis *mild* (since 2018)	Indapamide 1,5mg QD (since 2012); Comirnaty d-238 + d-217	43	43	43
	6	F	72	Allergy to amoxicillin, cefazolin, ciprofloxacin, gentamicin, pentoxifylline, euphyllin (since 2005); Varicose (since 2006); Hypertension *moderate* & chronic cardiac failure *moderate* (since 2010); Obesity *mild* (since 2021)	Lisinopril 10mg QD + Indapamide 1,5mg QD + Bisoprolol fumarate 5mg QD (since 2010)	43	43	43
	7	F	66	Allergy to vitamin B6, ketamine & intravenous anesthetics (since 1988); Hypertension *moderate* (since 2012); Chronic cardiac failure *moderate* (since 2015)	Indapamide 2,5mg QD + Bisoprolol fumarate 2,5mg QD (since 2012); Comirnaty d-175 + d-154	43	43	43
	8	M	69	Hypertension *moderate* & hypertensive cardiomyopathy *mild* (since 2006); Osteoarthritis *moderate* (since 2014)	Aspirin 75mg QD + Lozartan 50mg QD + Torasemid 5mg QD (since 2006)	43	43	43
	9	M	54	Obesity *mild* (since 2002)	Vaxzevria d-258 + d-202	43	43	43
500	10	M	73	Allergy to bismuth (vicair, vicalin) and streptomycin (since 1967); Hypertension *moderate* (since 2018); Chronic cardiac failure *moderate* + hypertensive cardiomyopathy *moderate* (since 2020); Obesity *mild* (since 2021)	Lisinopril 10mg QD + Lercanidipine 10mg QD (since 2018); Vaxzevria d-282 + d-238	43	43	43
	11	F	73	Varicose (since 1995); Hypertension *moderate* (since 2001); Duodenal ulcer *mild*(since 2008); Chronic cardiac failure *moderate* (since 2010)	Bisoprolol 5mg QD + Lisinopril 10mg QD + Inapamide 1,5mg QD (since 2001); Actovegin 200mg PRN (since 2012); Comirnaty d-174 + d-130; Nifedipime 10mg PRN (since 2021)	43	**60**	43
	12	F	73	-	Vaxzevria May & Jul 2021	43	43	**43**
	13	M	69	-	Janssen vaccine d-161	**292**	**155**	**287**
	14	M	28	-	-	43	43	43
	15	M	49	Allergic rhinits *mild* (2017); Hypertension *mild* (since 2020)	Enalapril 5mg BID (since 2020); Vaxzevria d-281 + d-218	43	43	43
	16	M	64	Hypertension *mild* (since 2022)	Spikevax May & June 2021;	43	**553**	**156**
	17	F	26	-	Vaxzevria d-310 + d-254	43	43	43
	18	F	32	Varicose *moderate* (since 2017); Saphenectomy on the right (in 2017)	Vaxzevria d-305 + d-250	43	43	43
1000	19	F	48	Had atrial fibrillation *mild* (between 2012-2019); Had cardiac ablation (in 2019); Hypertension *moderate* (since 2020); Chronic cardiac failure *moderate* (since 2021); Obesity *mild* (since 2021)	Metoprolol 100mg BID + valsartan 80mg QD (since 2020); Vaxzevria d-299 + d-264	43	**43**	43
	20	M	70	Hypertension *moderate* (since 2007); Hypertensive cardiomyopathy *moderate* + chronic cardiac failure *moderate* (since 2011); Adenomecty (in 2019)	Perindopril 1tablet QD (2019); Janssen COVID vx (d-183)	43	**215**	**123**
	21	F	64	Obesity *mild* (since 2010); Hypertension *moderate* (since 2016); Uterine leiomyoma (2015-2021); Hysterectomy (in 2021)	Exforge 1tablet QD + Nebivolol 5mg QD + Indapamide 1,5mg QD (since 2016); Sinopharm COVID vx Aug & Sep 2021	43	43	43
	22	F	68	Uterine leiomyoma + hysterectomy (in 2010); Sinus tachycardia *mild* (since 2017)	Bisoprolol 2,5mg QD (since 2016); Vaxzevria d-255 + d-198	43	**258**	**56**
	23	M	64	Duodenal ulcer (in 1978); Hypertension *mild* + hypertensive cardiomyopathy *mild* & obesity *mild* (since 2016)	Indapamide 1,5mg QD + betaxolol 20mg QD + ramipril 5mg QD (since 2016);	43	**43**	43
	24	F	27	Obesity *mild* (since 1996); Hypertension mild (since 2021)	Vaxzevria d-312 + d-243; Comirnaty d-61	43	43	43
	25	M	61	Ovarian cyst + cystectomy (in 1987); Allergic rhinitis *mild* (since 2017)	-	43	43	43
	26	F	60	Angia pectoris *moderate* + Hypertension *moderate* (since 2015); Hypertensive cardiomyopathy *moderate* + Bundle branch block left *mild* (since 2017); Hypercholesterolaemia *mild* (2022)	Comirnaty d-181 & d-147	43	43	43
	27	F	53	-	Aspirin 75mg QD + Lercanidipine HCl 10mg QD + Losartan K 50mg QD (since 2015); Metoprolol tartrate 25mg QD (since 2017); Janssen COVID vx (d-145); Rosuvastatin 10mg QD (-1 month)	43	43	43

F: female; M: male; T2D: type II diabetes ; COPD: chronic obstructive pulmonary disease; QD : once-per-day; IU: international unit; MRD: minimal required dilution.

Allergies and history of SARS-CoV-2 vaccinations are greyed out.
