# Supplementary material for: Characterization of the VHH-Fc construct rimteravimab in healthy adults and patients hospitalized for mild-to-moderate COVID-19: Two Phase 1 randomized clinical trials
Source: PLoS Med. 2026 May 29;23(5):e1004609. doi: 10.1371/journal.pmed.1004609 (PMC13221044; doi:10.1371/journal.pmed.1004609)
Supplement: S2 Checklist — (PDF) [file pmed.1004609.s003.pdf]

CONSERVE Checklists

Use CONSERVE-CONSORT for completed trial reports and CONSERVE-SPIRIT for trial protocols.

**CONSERVE-CONSORT Extension: 27/03/2026 - EXEVIR0101**			
**Item**	**Item Title**	**Description**	**Page No.**
I.	Extenuating Circumstances	Describe the circumstances and how they constitute extenuating circumstances.	Methods, Study Design and Procedure s
II.	Important Modifications	a. Describe how the modifications are important modifications.	Methods, Study Design and Procedure s
		b. Describe the impacts and mitigating strategies, including their rationale and implications for the trial.	(see below)
		c. Provide a modification timeline.	Methods, Study Design and Procedure s
III.	Responsible Parties	State who planned, reviewed and approved the modifications.	Methods, Study Design and Procedure s
IV.	Interim data	If modifications were informed by trial data, describe how the interim data were used, including whether they were examined by study group, and whether the individuals reviewing the data were blinded to the treatment allocation.	/
**CONSORT Number and Item**		For each row, if important modifications occurred check “direct impact” and/or “mitigating strategy” and describe the changes in the trial manuscript or supplement. Check “no change” for items that are unaffected in the extenuating circumstance.	**Page No.**

		No Change	Impact*	Mitigating Strategy**	
1	Title and abstract	/			
2	Introduction	/			
3	Methods: Trial Design	/			
4	Methods: Participants	/			
5	Methods: Interventions	/			
6	Methods: Outcomes	/			
7	Methods: Sample Size	/			
8-10	Methods: Randomisation	/			
11	Methods: Blinding	/			
12	Methods: Statistical methods	/			
13	Results: Participant flow	/			
14	Results: Recruitment		Part 2 not initiated	/	Study Design and Procedure s
15	Results: Baseline data		Part 2 not initiated	/	Study Design and Procedure s
16	Results: Numbers analysed		Part 2 not initiated	/	Study Design and Procedure s
	Results: Outcomes and estimation		Part 2 not initiated	/	Study Design and Procedure s
18	Results: Ancillary analyses	/			
19	Results: Harms	/			
20	Discussion: Limitations	/			
21	Discussion: Generalisability	/			

	Other information: Registration	/			
24	Other information: Protocol	/			
25	Other information: Funding	/			

*Aspects of the trial that are directly affected or changed by the extenuating circumstance and are not under the control of investigators, sponsor or funder.
**Aspects of the trial that are modified by the study investigators, sponsor or funder to respond to the extenuating circumstance or manage the direct impacts on the trial.

CONSERVE-SPIRIT Extension: [DATE]					
**Item**	**Item Title**	**Description**			**Page No.**
I.	Extenuating Circumstances	Describe the circumstances and how they constitute extenuating circumstances.			
II.	Important Modifications	a. Describe how the modifications are important modifications.			
		b. Describe the impacts and mitigating strategies, including their rationale and implications for the trial.			(see below)
		c. Provide a modification timeline.			
III.	Responsible Parties	State who planned, reviewed and approved the modifications.			
IV.	Interim data	If modifications were informed by trial data, describe how the interim data were used, including whether they were examined by study group, and whether the individuals reviewing the data were blinded to the treatment allocation.			
**SPIRIT Item and Number**		For each row, if important modifications occurred, check one or both of "impact" and/or "mitigating strategy" and describe the changes in the protocol. Check "no change" for items that are unaffected in the extenuating circumstance.			**Page No.**
		**No Change**	**Impact***	**Mitigating Strategy****	
1	Title				
2	Trial registration				
3	Protocol version				
4	Funding				
5	Roles and responsibilities				
6	Background and rationale				
7	Objectives				
8	Trial design				
9	Study setting				
10	Eligibility criteria				
11	Interventions				
12	Outcomes				

13	Participant timeline				
14	Sample size				
15	Recruitment				
16	Allocation				
17	Blinding (masking)				
18	Data collection methods				
19	Data management				
20	Statistical methods				
21	Data monitoring				
22	Harms				
23	Auditing				
24	Research ethics approval				
25	Protocol amendments				
26	Consent or assent				
27	Confidentiality				
28	Declaration of interests				
29	Access to data				
30	Ancillary and post-trial care				
31	Dissemination policy				
32	Informed consent materials				
33	Biological specimens				
*Aspects of the trial that are directly affected or changed by the extenuating circumstance and are not under the control of investigators, sponsor or funder.<br>**Aspects of the trial that are modified by the study investigators, sponsor or funder to respond to the extenuating circumstance or manage the direct impacts on the trial.					
