# Supplementary material for: Characterization of the VHH-Fc construct rimteravimab in healthy adults and patients hospitalized for mild-to-moderate COVID-19: Two Phase 1 randomized clinical trials
Source: PLoS Med. 2026 May 29;23(5):e1004609. doi: 10.1371/journal.pmed.1004609 (PMC13221044; doi:10.1371/journal.pmed.1004609)
Supplement: S1 Protocol — (PDF) [file pmed.1004609.s004.pdf]

CLINICAL STUDY PROTOCOL

A PHASE 1 SINGLE ASCENDING DOSE STUDY IN HEALTHY SUBJECTS TO EVALUATE SAFETY, TOLERABILITY, AND PHARMACOKINETICS OF XVR011

CONFIDENTIAL

Sponsor code: EXEVIR0102
PRA code: EVB21546-21546X
EudraCT number: 2021-003707-17
IND number: Not applicable

Single ascending dose (SAD) study with XVR011 in healthy subjects

Investigational product:	XVR011
Clinical phase:	Phase 1 study
Indication to be studied:	Not applicable
Sponsor:	ExeVir Bio BV<br>Rijvisschestraat 120<br>Ghent, 9052<br>Belgium
Contract Research Organization and Clinical Site:	PRA Health Sciences (PRA) – Early Development Services (EDS)<br>Van Swietenlaan 6<br>9728 NZ Groningen<br>The Netherlands

Principal Investigator: Salah Hadi, MD
PRA Group BV, a PRA Health Sciences Company
Phone: +31 50 402 2524
Fax: +31 50 402 2223
E-mail: HadiSalah@prahs.com

Version 1.0, 15 Jul 2021

This study will be performed in compliance with the principles of Good Clinical Practice.

Strictly Confidential. © 2021, PRA Health Sciences. All rights reserved.

SPONSOR AUTHORIZATION OF CLINICAL STUDY PROTOCOL

The Sponsor and the Contract Research Organization agree to conduct the study as outlined in this clinical study protocol. Any modification of the clinical study protocol must be agreed upon by the Sponsor and the Contract Research Organization and must be documented in writing.

Name/Position:	Date:	Signature:
Sponsor: ExeVir Bio BV		
Dominique Tersago, MD<br>Chief Medical Officer	……………………	Dominique Tersago (Signature)<br>Digitally signed by Dominique Tersago (Signature)<br>Date: 2021.07.16 09:23:09 +02'00'<br>………………………

Strictly Confidential. © 2021, PRA Health Sciences. All rights reserved.

AUTHORIZATION OF CLINICAL STUDY PROTOCOL BY CONTRACT RESEARCH ORGANIZATION

The Sponsor and the Contract Research Organization agree to conduct the study as outlined in this clinical study protocol. Any modification of the clinical study protocol must be agreed upon by the Sponsor and the Contract Research Organization and must be documented in writing.

Name/Position:	Date:	Signature:

Contract Research Organization: PRA Health Sciences – Early Development Services

Salah Hadi, MD<br>Principal Investigator<br>PRA Group BV, a PRA Health Sciences Company	……………………	………………………

Strictly Confidential. © 2021, PRA Health Sciences. All rights reserved.

SERIOUS ADVERSE EVENT CONTACT INFORMATION

In case of a serious adverse event (see Appendix 8.2), the Principal Investigator will send a report within 24 hours of notification to:

Sponsor
Dominique Tersago, MD
Chief Medical Officer
ExeVir Bio BV
Rijvisschestraat 120
Ghent, 9052
Belgium
Phone: +32 2 899 87 37
Fax: +32 2 899 87 38
E-mail: dtersago@exevir.com

Contract Research Organization
Parexel Safety Services
France (Paris) SAE Mailbox: Medical_Paris@parexel.com
France (Paris) Fax numbers: +33 1 44 90 32 75 or +33 1 44 90 35 34
SAE answering service: +33 1 44 90 32 90

Strictly Confidential. © 2021, PRA Health Sciences. All rights reserved.

CONTACT INFORMATION

Sponsor
ExeVir Bio BV
Rijvisschestraat 120
Ghent, 9052
Belgium

Sponsor's Medical Expert
Dominique Tersago, MD
Chief Medical Officer
Phone: +32 2 899 87 37
Mobile: +32 475 24 25 09
Fax: +32 2 899 87 38
E-mail: dtersago@exevir.com

Contract Research Organization and Clinical Site
PRA–EDS
Van Swietenlaan 6
9728 NZ Groningen
The Netherlands
Phone: +31 50 402 2222
Fax: +31 50 402 2223

Principal Investigator
Salah Hadi, MD
Phone: +31 50 402 2524
Fax: +31 50 402 2223
E-mail: HadiSalah@prahs.com

Laboratories
Clinical Laboratory
PRA-EDS
Van Swietenlaan 6
9728 NZ Groningen
The Netherlands
Phone: +31 50 402 2555
Fax: +31 50 402 2223

Laboratory for Analysis of Serum Samples for XVR011 and Antidrug Antibodies Against XVR011
LGC Bioanalytical Laboratory
Large Molecule Bioanalysis
Newmarket Road, Fordham
Cambridgeshire, CB7 5WW
United Kingdom
Phone: +44 1638 722965

Strictly Confidential. © 2021, PRA Health Sciences. All rights reserved.

Medical Screening Centers

PRA-EDS
Van Swietenlaan 6
9728 NZ Groningen
The Netherlands
Phone: +31 50 850 5798
Fax: +31 50 850 5782

PRA-EDS
Yin Yang Building
Euclideslaan 1
3584 BL Utrecht
The Netherlands
Phone: +31 50 851 5830

Study Monitor

PRA
Yin Yang Building
Euclideslaan 1
3584 BL Utrecht
The Netherlands
Phone: +31 50 851 5830

Pharmacovigilance

Parexel Safety Services
France (Paris) SAE Mailbox: Medical_Paris@parexel.com
France (Paris) Fax numbers: +33 1 44 90 32 75 or +33 1 44 90 35 34
SAE answering service: +33 1 44 90 32 90

Strictly Confidential. © 2021, PRA Health Sciences. All rights reserved.

SYNOPSIS

Study Title
A PHASE 1 SINGLE ASCENDING DOSE STUDY IN HEALTHY SUBJECTS TO EVALUATE SAFETY, TOLERABILITY, AND PHARMACOKINETICS OF XVR011

Short Study Title
SAD study with XVR011 in healthy subjects

Study Codes

Sponsor code	: EXEVIR0102
PRA code	: EVB21546-21546X
EudraCT number	: 2021-003707-17

Sponsor
ExeVir Bio BV, Rijvisschestraat 120, Ghent, 9052, Belgium

Sponsor's contact	: Ellen Jansen, MS, Clinical Operations Lead<br>Dominique Tersago, MD, Chief Medical Officer

Contract Research Organization and Clinical Site
PRA-EDS, Van Swietenlaan 6, 9728 NZ Groningen, The Netherlands

Principal Investigator
Salah Hadi, MD

Objectives

Primary	: To evaluate the safety and tolerability of XVR011 after an intravenous (iv) infusion of single ascending doses in healthy subjects.
Secondary	: To evaluate the pharmacokinetic (PK) profile of XVR001 after an iv infusion of single ascending doses in healthy subjects.
Exploratory	: To evaluate the immunogenicity of XVR011 after an iv infusion of single ascending doses in healthy subjects.

Design and Treatments
This will be a Phase 1, randomized, double-blinded, single-center, placebo-controlled, SAD study in a maximum of 3 groups of 10 healthy male and/or female subjects each.

The following treatments are planned to be administered:
Group 1: A single iv infusion of 250 mg XVR011 (n=8) or matching placebo (n=2) on Day 1
Group 2: A single iv infusion of 500 mg XVR011 (n=8) or matching placebo (n=2) on Day 1
Group 3: A single iv infusion of 1000 mg XVR011 (n=8) or matching placebo (n=2) on Day 1

In each group, subjects will be dosed according to a sentinel dosing design to ensure optimal safety. This means that, in each group, initially 2 subjects will be dosed: 1 subject with XVR011 and 1 subject with placebo. If the safety and tolerability results of the first 24 hours following dosing in the initial subjects are acceptable to the Investigator, the remaining 8 subjects (7 active and 1 placebo) of the group will be dosed.

Dose escalation will only proceed when none of the stopping criteria have been met and when the safety and tolerability up to Day 8 of the last preceding dose group, as well as any significant adverse events (AEs) in earlier groups and available PK data, have been reviewed and considered acceptable to the Investigator and the Sponsor and, if deemed necessary, after a statement of no objection on the dose escalation report

Strictly Confidential. © 2021, PRA Health Sciences. All rights reserved.

from the Independent Ethics Committee. The minimum number of subjects required to decide on dose escalation is 8 subjects.

Study Schedule*

Screening	: Between Day -21 and Day -1 (admission)
Assessment period	: An anticipated single period in the clinical research center from Day -1 (admission) to approximately 24 hours after ending the study drug infusion (Day 2). Subjects will return to the clinical research center for ambulatory visits on Days 8, 15, 29, 43, and 57 (±1 day each)
End of study	: Day 85 (±2 days) (ambulatory visit)

Subjects

30 healthy male and/or female subjects (10 subjects per group)

Main Criteria for Inclusion

Age	: 18 to 65 years, inclusive, at screening
Body mass index	: 18.0 to 30.0 kg/m^2, inclusive, at screening
Sex	: Male or female
Health status	: Healthy

Study Drug

<u>Active Medication</u>

Active substance	: XVR011, a humanized heavy chain-only antibody fragment linked to an effector-function partially silenced, crystallizable fragment (Fc) part of a human immunoglobulin G subclass 1 (IgG1)
Activity	: XVR011 binds to the spike protein of the severe acute respiratory syndrome coronavirus 2 (SARS-CoV-2) and, through steric hindrance, prevents the virus from binding to the angiotensin-converting enzyme 2 (ACE2) receptor, thereby preventing it from entering the cell
In development for	: Coronavirus disease 2019 (COVID-19); it is intended as an antiviral treatment to halt infection in the patient and thereby reduce the chance of progression to more severe disease
Strength	: 50 mg/mL
Dosage form	: Solution for iv infusion
Manufacturer	: Provided by the Sponsor

<u>Placebo (Visually Matching Active Medication)</u>

Substance	: 0.9% (w/v) sodium chloride sterile solution
Activity	: Not applicable
Strength	: Not applicable
Dosage form	: Solution for iv infusion
Manufacturer	: Sourced by pharmacy at PRA

Variables*

Safety	: Adverse events, clinical laboratory, vital signs, 12-lead electrocardiograms, physical examinations, local tolerability, and immunogenicity measurements (exploratory)
Pharmacokinetics	: Serum XVR011 concentrations<br>Serum PK parameters for XVR011 estimated using noncompartmental analysis, as appropriate: C_{pre}, C_{max}, t_{max}, $AUC_{0\text{-Day 8}}$, $AUC_{0\text{-t}}$, $AUC_{0\text{-inf}}$, $\%AUC_{extra}$, k_{el}, $t_{1/2}$, CL, V_z, MRT, and time of serum concentration above 26.9 μg/mL and above 50.4 μg/mL

Strictly Confidential. © 2021, PRA Health Sciences. All rights reserved.

* The timing and number of safety and PK assessments may be changed during the study depending on emerging study results.

Statistical Methods

Sample size calculation	:	The sample size has been selected to provide information on safety, tolerability, PK, and immunogenicity following single iv infusion of XVR011. Any p-values to be calculated according to the statistical analysis plan will be interpreted in the perspective of the explorative character of this study.
Safety parameters	:	Descriptive statistics for all relevant safety data.
PK parameters	:	Descriptive statistics for all relevant PK data. A power model on C_{max} and AUC will be performed to determine dose proportionality.
Immunogenicity parameters (exploratory)	:	Descriptive statistics for all relevant antidrug antibody (ADA) data. If a sizable number of subjects have ADA formation, the relationship between ADA positive and ADA negative subjects with regard to AUC and time of serum concentration above 26.9 µg/mL and above 50.4 µg/mL will be explored.

Strictly Confidential. © 2021, PRA Health Sciences. All rights reserved.

Table 1 Schedule of Assessments

Visit	Screening	Assessment Period[a]									EOS
**Study Day**	**Days -21 to -1**	**Day -1**	**Day 1 (predose)**	**Day 1**	**Day 2**	**Day 8 (±1 day)**	**Day 15 (±1 day)**	**Day 29 (±1 day)**	**Day 43 (±1 day)**	**Day 57 (±1 day)**	**Day 85 (±2 days) or early termination**
Admission		X									
Confinement		X	X	X	X						
Discharge					X						
Ambulatory	X					X	X	X	X	X	X
Informed Consent	X										
Medical History	X	X									
Demographics	X										
Body Weight and BMI Calculation	X										
Height	X										
Serology (HBsAg, anti-HCV, anti-HIV 1 and 2)	X										
SARS-CoV-2 PCR test		X			X			X		X	X
Drug and Alcohol Screen	X	X									
Serum Pregnancy Test (Females Only)	X	X									X
FSH Test (Females Only)	X										
Clinical Laboratory[b]	X	X	X		X	X	X	X			X
Physical Examination[c]	X										X
Vital Signs[d]	X		X	X	X	X	X	X			X
12-lead ECG[e]	X	X	X	X	X			X			X
Local Tolerability[f]			X	X	X						
Eligibility Check	X	X	X								
Study Drug Administration[g]				X							
Blood Sampling for PK in Serum[h]			X	X	X	X	X	X	X	X	X
Blood Sampling for ADAs in Serum			X					X			X
Previous/Concomitant Medication	X ----- CONTINUOUS MONITORING------X										
AE Monitoring		X ----- CONTINUOUS MONITORING------X									

ADA=antidrug antibody; AE=adverse event; BMI=body mass index; ECG=electrocardiogram; EOS=end of study; FSH=follicle-stimulating hormone; HBsAg=hepatitis B surface antigen; HCV=hepatitis C virus; iv=intravenous; PCR=polymerase chain reaction; PK=pharmacokinetic(s); SARS-CoV-2=severe acute respiratory syndrome coronavirus 2

a Subjects will be admitted to the clinical research center on Day -1 and will remain until approximately 24 hours after ending the study drug infusion (Day 2). Subjects will return to the clinical research center for ambulatory visits on Days 8, 15, 29, 43, and 57 (±1 day each) and for the EOS visit on Day 85 (±2 days).

b Clinical laboratory tests (including clinical chemistry, hematology, coagulation, and urinalysis) will be performed at screening; at admission; at predose; at 24 hours after the start of the iv infusion; at Days 8, 15, and 29; and at the EOS/early termination visit. At the discretion of the Investigator, additional clinical laboratory samples may be taken as needed. In case of an infusion-related reaction that is possibly related to complement activation (fast onset and symptoms such as flushing, rash, dyspnea, chest pain, back pain, and/or subjective distress), additional serum samples will be taken during the reaction and after cessation of the reaction. The serum samples will be stored frozen for potential analysis of, eg, complement activity.

c Complete physical examinations will be conducted at screening and at the EOS/early termination visit. Symptom-driven physical examinations may be conducted at any time at the Investigator's discretion.

d Systolic and diastolic blood pressure and heart rate will be recorded after resting for at least 5 minutes in the supine position. Body temperature and respiratory rate will also be measured. Vital signs will be collected at screening; at predose; at 5 minutes and 15 minutes after the start of the iv infusion; every 15 minutes from 15 minutes until 4 hours after the start of the iv infusion; every 30 minutes from 4 hours until 6 hours after the start of the iv infusion; thereafter at 8, 12, 16, and 24 hours after the start of the iv infusion; at Days 8, 15, and 29; and at the EOS/early termination visit.

e Single 12-lead ECG after resting for at least 5 minutes in the supine position: at screening; at admission; at predose; at 1, 2, 4, 8, 12, and 24 hours after the start of the iv infusion; at Day 29; and at the EOS/early termination visit.

f Local tolerability (ie, examination of infusion site for potential reactions): at Day 1 predose and at 8 and 24 hours after the start of the iv infusion.

g Study drug will be administered via an iv infusion of 90-minute duration while subjects are in a supine or semi-supine position. Close monitoring by the clinical staff per standard site procedures during the infusion and for at least 120 minutes after the end of infusion is required.

h Blood sampling for PK of XVR011 in serum: at predose; at 1.5 hours after the start of the iv infusion (ie, immediately after the end of the iv infusion); at 2, 7.5, 11.5, and 25.5 hours after the start of the iv infusion; and at all following visit days until EOS.

TABLE OF CONTENTS

TITLE PAGE 1

SPONSOR AUTHORIZATION OF CLINICAL STUDY PROTOCOL 2

AUTHORIZATION OF CLINICAL STUDY PROTOCOL BY CONTRACT RESEARCH ORGANIZATION 3

SERIOUS ADVERSE EVENT CONTACT INFORMATION 4

CONTACT INFORMATION 5

SYNOPSIS 7

TABLE OF CONTENTS 12

TABLE OF TABLES 15

TABLE OF FIGURES 15

LIST OF ABBREVIATIONS 16

1. INTRODUCTION 18
   1.1 Background 18
      1.1.1 Nonclinical Summary 19
      1.1.2 Clinical Summary 20
   1.2 Risk-benefit Assessment 20
   1.3 Study Rationale 22

2. OBJECTIVES 23
   2.1 Primary 23
   2.2 Secondary 23
   2.3 Exploratory 23

3. INVESTIGATIONAL PLAN 24
   3.1 Overall Study Design and Plan 24
      3.1.1 Type of Study 24
      3.1.2 Screening Period 24
      3.1.3 Assessment Period 25
      3.1.4 End of Study/Early Termination 25

Strictly Confidential. © 2021, PRA Health Sciences. All rights reserved.

3.2 Discussion of Study Design 25
3.2.1 Dose Escalation 25
3.2.1.1 Stopping Rules for Dose Escalation 26
3.2.1.1.1 Within-group Stopping Rules 26
3.2.1.1.2 Between-group Stopping Rules 26
3.2.2 Sentinel Dosing 27
3.2.3 Other 27
3.2.4 COVID-19 Risk Mitigation 28
3.3 Selection of Study Population 29
3.3.1 Inclusion Criteria 29
3.3.2 Exclusion Criteria 30
3.3.3 Removal of Subjects from Assessment 31
3.3.3.1 Stopping Rules for Individual Subjects 32
3.4 Treatments 32
3.4.1 Treatments Administered 32
3.4.2 Identity of Investigational Product 33
3.4.3 Method of Assigning Subjects to Treatment Groups 33
3.4.4 Selection of Doses in the Study 34
3.4.5 Timing of Doses in the Study 35
3.4.6 Meals During the Study 35
3.4.7 Blinding 35
3.4.8 Concomitant Medication and Other Restrictions During the Study 36
3.4.9 Treatment Compliance 37
3.5 Pharmacokinetic and Safety Measurements and Variables 37
3.5.1 PK and Safety Measurements 37
3.5.1.1 PK Measurements 37
3.5.1.2 Safety and Tolerability Measurements 38
3.5.1.2.1 AEs 38
3.5.1.2.2 Clinical Laboratory 38
3.5.1.2.3 Vital Signs 39
3.5.1.2.4 ECGs 39
3.5.1.2.5 Physical Examination 40
3.5.1.2.6 Local Tolerability 40
3.5.1.2.7 Immunogenicity Measurements (Exploratory) 40
3.5.1.3 Total of Blood Volume 40
3.5.2 Appropriateness of Measurements 40
3.5.2.1 Timing of Assessments 41
3.5.3 PK and Safety Variables 41
3.5.3.1 PK Variables 41
3.5.3.2 Safety Variables 41
3.5.4 Drug Concentration Measurements 42
3.5.5 Retention of Blood and Urine Samples 42
3.6 Statistical Procedures and Determination of Sample Size 42
3.6.1 Analysis Sets 42
3.6.1.1 Safety Set 42
3.6.1.2 PK Set 42

Strictly Confidential. © 2021, PRA Health Sciences. All rights reserved.

3.6.2 Statistical and Analytical Plan for Pharmacokinetic, Safety, and Exploratory Evaluation 42
3.6.2.1 PK Evaluation 43
3.6.2.2 Evaluation of Safety and Tolerability 43
3.6.2.2.1 AEs 43
3.6.2.2.2 Clinical Laboratory 43
3.6.2.2.3 Vital Signs, ECGs, and Physical Examinations 43
3.6.2.2.4 Immunogenicity Measurements (Exploratory) 43
3.6.3 Interim Analysis 44
3.6.4 Determination of Sample Size 44
3.7 Data Quality Assurance 44

4. ETHICS 45
4.1 IEC 45
4.2 Ethical Conduct of the Study 45
4.3 Subject Information and Consent 46
4.4 Privacy 46

5. STUDY ADMINISTRATIVE STRUCTURE 47
5.1 Distribution of Activities 47
5.1.1 Preparation of Study Drug 47
5.1.2 Laboratory Assessments 47
5.1.3 Electronic Case Report Form Design 47
5.1.4 Data Management 47
5.1.5 Statistics 47
5.1.6 CSR Writing 47
5.2 Documentation 47
5.2.1 Archiving 47
5.2.2 Recording of Data in Source Documents and Electronic Case Report Forms 48

6. CONFIDENTIALITY AND PUBLICATION POLICY 49

7. REFERENCES 50

8. APPENDICES 52
8.1 Drug Accountability 52
8.2 Adverse Events and Serious Adverse Events Evaluation and Reporting 52
8.2.1 AEs 52
8.2.2 SAEs 53
8.2.3 Suspected Unexpected Serious Adverse Reactions 54
8.2.4 Follow-up of SAEs 55
8.3 Pregnancy 55

Strictly Confidential. © 2021, PRA Health Sciences. All rights reserved.

TABLE OF TABLES

Table 1 Schedule of Assessments 10
Table 2 Guidance in Case of an Infusion Reaction Based on Severity of the Reaction 32
Table 3 Intravenous Infusion Details 33
Table 4 Number and Volume of Blood Samples and Total Blood Volume Collected per Subject 40
Table 5 PK Parameters 41

TABLE OF FIGURES

Figure 1 Study Design Overview 24

Strictly Confidential. © 2021, PRA Health Sciences. All rights reserved.

LIST OF ABBREVIATIONS

ACE2	angiotensin-converting enzyme 2
ADA	antidrug antibodies
ADL	activities of daily living
AE	adverse event
ARDS	acute respiratory distress syndrome
BMI	body mass index
C1q	complement component 1q
CA	Competent Authority
COVID-19	coronavirus disease 2019
CSP	clinical study protocol
CSR	clinical study report
CTCAE	Common Terminology Criteria for Adverse Events
CTD	Clinical Trial Directive
DER	dose escalation report
ECG	electrocardiogram
eCRF	electronic case report form
EDS	Early Development Services
EMA	European Medicines Agency
EOS	end of study
Fc	crystallizable fragment
FSH	follicle-stimulating hormone
GCP	Good Clinical Practice
HBsAg	hepatitis B surface antigen
HCV	hepatitis C virus
IB	Investigator’s Brochure
ICF	informed consent form
ICH	International Council for Harmonisation
IEC	Independent Ethics Committee
IgG1	immunoglobulin G subclass 1
iv	intravenous
MEB	Medicine Evaluation Board
MedDRA	Medical Dictionary for Regulatory Activities
MERS	Middle East respiratory syndrome
PCR	polymerase chain reaction
PK	pharmacokinetic(s)
PKPD	pharmacokinetic-pharmacodynamic
PRA	PRA Health Sciences
SAD	single ascending dose
SAE	serious adverse event
SAP	statistical analysis plan
SARS	severe acute respiratory syndrome
SARS-CoV-1	severe acute respiratory syndrome coronavirus 1
SARS-CoV-2	severe acute respiratory syndrome coronavirus 2
SOC	system organ class
SOP	standard operating procedure

Strictly Confidential. © 2021, PRA Health Sciences. All rights reserved.

SUSAR	suspected unexpected serious adverse reaction
TEAE	treatment-emergent adverse event
TCR	tissue cross-reactivity
VHH	variable domain of a heavy chain-only antibody fragment
WHO	World Health Organization
WMA	World Medical Association
WMO	Wet Medisch-Wetenschappelijk Onderzoek Met Mensen (Medical Research Involving Human Subjects Act)

Note: Definitions of pharmacokinetic (PK) parameters are provided in Section 3.5.3.

Strictly Confidential. © 2021, PRA Health Sciences. All rights reserved.

1. INTRODUCTION

XVR011 is being developed by ExeVir Bio BV (hitherto referred to as “Sponsor”) as a single intravenous (iv) infusion for the treatment of coronavirus disease 2019 (COVID-19).

XVR011 is a humanized heavy chain-only antibody fragment linked to an effector-function partially silenced, crystallizable fragment (Fc) part of a human immunoglobulin G subclass 1 (IgG1). It binds to the spike protein of the severe acute respiratory syndrome coronavirus 2 (SARS-CoV-2) and, through steric hindrance, prevents the virus from binding to the angiotensin-converting enzyme 2 (ACE2) receptor, thereby preventing it from entering the cell. It is intended as an antiviral treatment to halt infection in the patient and thereby reduce the chance of progression to more severe disease. The partially silenced, Fc function means that XVR011 should not bind to the Fc receptor on immune cells and is expected to avoid immune-mediated exacerbation (antibody-dependent enhancement) of the disease.

The proposed population is patients with COVID-19 in whom the viral load is causing mild to moderate disease and who require medical care or supplemental oxygen in the hospital setting but who are not in need of mechanical ventilation or intensive care. The aim of treatment with XVR011 is to neutralize the virus, achieve faster clinical recovery, and thereby prevent progression to severe complications such as acute respiratory distress syndrome (ARDS) or other major organ dysfunctions.

1.1 Background

SARS-CoV-2 has demonstrated high transmissibility and infectivity, and its widespread infection has led to the declaration of a global pandemic by the World Health Organization (WHO). [1] Although the majority (80%) of people with COVID-19 exhibit mild symptoms and recover spontaneously, approximately 14% of individuals will require hospitalization, and 30% of hospitalized patients will develop respiratory failure, shock, and/or multiple organ system dysfunction, thus requiring admission to a critical care unit. [2]

The RNA genome of SARS-CoV-2 encodes for 4 major structural proteins (spike, envelope, membrane, and nucleocapsid), approximately 16 nonstructural proteins, and 5 to 8 accessory proteins. Among these, the spike protein plays a crucial role in viral attachment, fusion, entry, and transmission. [3] The viral spike protein binds to host cell-bound ACE2, which is the primary mechanism by which the severe acute respiratory syndrome (SARS) coronavirus infects human cells. The virus then rapidly replicates in the upper respiratory tract, with viral load reaching a peak within the first week of symptom onset and later in the lower respiratory tract. [4]

Some studies suggest a peak in viral load occurs in the prodromal stage of the infection. Furthermore, a large number of infected individuals remain asymptomatic. Symptoms are variable both in type and severity. The most common symptoms are fever, dry cough, and fatigue. Other less common symptoms include loss of taste or smell, nasal congestion, conjunctivitis, sore throat, headache, muscle or joint pain, skin rash,

Strictly Confidential. © 2021, PRA Health Sciences. All rights reserved.

nausea/vomiting, diarrhea, chills, or dizziness. [5] Hospitalization is necessary mainly for people who develop respiratory distress. This can rapidly progress and become very severe, requiring invasive, mechanical ventilation and intensive care treatment. The pathogenesis of severe COVID-19 is an aberrant immune response with overproduction of pro-inflammatory cytokines and damage to the lung parenchyma. [6] Host factors such as older age, male gender, and underlying medical conditions appear to be predisposing factors to development of severe disease.

Several classes of drugs are currently being evaluated or developed for the management of COVID-19, including antivirals (eg, remdesivir, favipiravir), antibodies (eg, convalescent plasma, hyperimmune immunoglobulins), anti-inflammatory agents (eg, dexamethasone, statins), targeted immunomodulatory therapies (eg, tocilizumab, sarilumab, anakinra, ruxolitinib), anticoagulants (eg, heparin), and antifibrotics (eg, tyrosine kinase inhibitors). [7]

Viral neutralizing antibodies induced by vaccines or the virus are critical in the control of viral infection. Monoclonal antibodies have been shown to successfully block viral binding to host cells in other members of the coronavirus family (SARS and Middle East respiratory syndrome [MERS]). [8, 9]

XVR011 is the variable domain of a heavy chain-only antibody fragment (VHH) linked to a partially silenced, Fc fragment of a human IgG1. The VHH component was derived from a heavy chain-only antibody from a llama inoculated with severe acute respiratory syndrome coronavirus 1 (SARS-CoV-1, ie, SARS) spike protein. When SARS-CoV-2 (ie, COVID-19) was identified, the VHH was found to be cross-reactive to its spike protein. The VHH binds to an epitope on the receptor-binding domain of the SARS-CoV-2 spike protein and through steric hindrance, blocks the interaction of the spike protein with the ACE2 receptor and thereby prevents the virus from entering the cell and replicating. The binding site is partially hidden and is therefore expected to be less susceptible to genetic pressure and viral mutation. The amino acid sequence of the VHH was further humanized and optimized to increase its antiviral potency. In XVR011, 2 VHHs are linked to a human IgG1 Fc fragment to achieve a half-life similar to that of naturally occurring antibody, ie, approximately 21 days. The Fc has been modified with a LALA mutation to silence the effector function. This has been done to minimize the potential for antibody-dependent enhancement of disease. In patients with SARS-CoV-2 infection, the XVR011 virus complex is expected to be cleared by a nonspecific mechanism such as nonspecific pinocytosis. In healthy subjects in whom there is no target mediated clearance, elimination is assumed to be primarily through intracellular catabolism by lysosomal degradation to amino acids after uptake by either pinocytosis or an unspecific fluid phase endocytosis. Target-mediated clearance is not expected to result in any meaningful difference in exposure between patients and healthy subjects. Therefore the exposure in healthy subjects is considered to be predictive of that in patients.

Further details are provided in the latest version of the Investigator's Brochure (IB).[10]

1.1.1 Nonclinical Summary

The antiviral activity of XVR011 has been demonstrated in neutralization assays in vitro with both the live virus and a pseudovirus, which expresses the spike protein. IC50 and

Strictly Confidential. © 2021, PRA Health Sciences. All rights reserved.

IC90 concentrations for neutralization are in the nanomolar and picomolar ranges respectively, indicating that XVR011 is a highly potent molecule. This has been confirmed and demonstrated in vivo in a number of studies using the Syrian Golden Hamster model of the SARS-CoV-2 infection. Various dose levels, 2 viral isolates, and both treatment and prophylactic setting were evaluated. Viral load was assessed using viral RNA and infectious viral particle measurements primarily in the lung. Additionally, lung tissue was histologically examined. Significant reduction in viral RNA and absence of infectious viral particles were observed in a dose-related manner.

Since XVR011 targets a viral protein, it is not expected to bind to any human proteins. Several nonclinical safety assessments have been performed so far, and no safety signals were observed that were considered related to XVR011. Human tissue cross-reactivity (TCR) was not observed in a standard Good Laboratory Practice human TCR study using a full panel of human tissues. No off-target binding to human proteins was observed in a human protein expressing cell microarray assay. In addition, no cytokine release was observed when XVR011 was incubated with whole blood from healthy human donors in an ex vivo cytokine release assay. Silencing of the Fc part of XVR011 was confirmed for all Fc gamma receptors through surface plasmon resonance binding studies and with a functional assay of antibody-dependent cellular cytotoxicity. Complement component 1q (C1q) binding of XVR011 was similar to the positive control. Complement-dependent cytotoxicity activity reduction is still being evaluated. Given the additional steps needed for actual complement activation, eg, binding to the hexamer of C1q in vivo and subsequent complement membrane attack complex formation, it is also expected that complement-mediated Fc effects are silenced.

A detailed description of the nonclinical studies is provided in the latest version of the IB. [10]

1.1.2 Clinical Summary

Currently a dose-finding, safety, and efficacy study of XVR011 added to standard of care in patients hospitalized for COVID-19 is recruiting in parallel to this study (EXEVIR0101; ClinicalTrials.gov Identifier: NCT04884295). The study is a 2-part study in which the first part (Phase 1) evaluates the safety of XVR011 and aims to identify the recommended Phase 2 dose in patients. The second part (Phase 2) follows the first part and evaluates whether XVR011 added to standard of care in patients hospitalized for mild to moderate COVID-19 is a safe, well-tolerated, and effective treatment.

1.2 Risk-benefit Assessment

There is no expected clinical benefit for the healthy subjects who will participate in this study. The information obtained in this study can be used for the further clinical development of XVR011. Based on the potential benefit of XVR011, it is being developed for patients with COVID-19. More detailed information about the expected benefits and risks and potential adverse events (AEs) of XVR011 may be found in the latest version of the IB. [10]

Possible clinical risks for healthy subjects linked to the study treatment XVR011 are immune reactions to a foreign protein and/or infusion reactions. As with any protein, there is a potential that the immune system will recognize it as foreign and trigger a

Strictly Confidential. © 2021, PRA Health Sciences. All rights reserved.

hypersensitivity reaction. This may vary from mild to moderate signs and symptoms but may also be a severe or life-threatening anaphylactic/anaphylactoid reaction. Signs and symptoms may include hypotension, tachycardia, bronchospasm, dyspnea, edema/angioedema, dizziness, headache, nausea, abdominal pain, fever, and/or rash. These risks will be mitigated by:

- Ensuring emergency equipment/medication are available within the treatment area.
- Informing the subject of possible signs/symptoms of hypersensitivity prior to the injection/infusion and advising the subject to immediately report if any of these occur.
- Close monitoring by the clinical staff per standard site procedures during the infusion and for at least 120 minutes afterwards is required. Sentinel subjects should be monitored for 24 hours after the infusion (see Section 3.2.2).
- Interrupting/stopping the injection/infusion immediately in case of any sign or symptom of an infusion reaction.
- Administering appropriate treatment, according to institutional guidelines and, depending on the severity of the symptoms, may include antihistamines, corticosteroids, bronchodilators, vasopressors, and other treatments as per Investigator judgment.

Possible risks for healthy subjects related to the required study procedures are discomfort after the nasal swab for COVID-19 polymerase chain reaction (PCR) testing and the side effects from blood sample collection. The subject might experience redness, induration, or bruises after blood sample collection. These side effects will be mitigated by having all study assessments performed by well-trained staff to minimize discomfort and by taking all measures to avoid side effects.

Overall, on the basis of the available nonclinical and clinical data and prior knowledge, the risk-benefit profile of XVR011 is judged acceptable for the proposed clinical study.

The risk-benefit assessment for the subjects receiving XVR011 remains unchanged in relation to the COVID-19 pandemic as available nonclinical results do not suggest that administration of XVR011 will lead to suppression or modulation of the immune system. In addition, the mode of action does not appear to have a substantial effect on the respiratory or cardiovascular system critically affected by a SARS-CoV-2 infection. As the subjects to be included in this study are in general young to middle-aged without major comorbidities, the study population is not considered to be a high-risk population for serious COVID-19 disease. Only persons with a negative SARS-CoV-2 test at admission to the clinical research center will be allowed to participate in the study. In addition, all appropriate measures to prevent SARS-CoV-2 infection during the study will be taken as detailed in Section 3.2.4. To avoid potential interference with immunity induced by vaccination with vaccines that use the SARS-CoV-2 spike protein or elements thereof, subjects must either be fully vaccinated against COVID-19 at least 14 days prior to study drug administration or be willing to delay planned vaccination until 90 days after study drug administration.

Strictly Confidential. © 2021, PRA Health Sciences. All rights reserved.

1.3 Study Rationale

This clinical study in healthy subjects is designed to evaluate the pharmacokinetic (PK) parameters, safety, and tolerability of a single dose of XVR011 administered as an iv infusion in 3 sequential, ascending dose groups. It will be a blinded, placebo-controlled evaluation. This study will be conducted in parallel with a Phase 1/2 clinical study (EXEVIR0101, NCT04884295) in patients hospitalized with mild to moderate COVID-19. The justification for the healthy subject study at this time is that it allows more intense blood sampling to be done for the PK parameters, particularly in the first 24 hours postdose, than is feasible in the patient study. It will also provide an assessment of safety and tolerability that is not confounded by the disease for which XVR011 is intended as treatment.

Recruitment for Part 1 (the Phase 1 component) of the patient study, which is being conducted in 3 European countries (Belgium, Italy, and Portugal), is unpredictable due to the low rates of infection in those countries. Given the continuing unmet needs of patients with COVID-19 and the potential of XVR011 to be an effective antiviral treatment, also against the SARS-CoV-2 variants of concern, the approach of conducting a patient and a healthy subject Phase 1 study in parallel to ensure timely data for the Phase 2 component of the patient study is considered warranted.

Strictly Confidential. © 2021, PRA Health Sciences. All rights reserved.

2. OBJECTIVES

2.1 Primary

To evaluate the safety and tolerability of XVR011 after an iv infusion of single ascending doses in healthy subjects.

2.2 Secondary

To evaluate the PK profile of XVR001 after an iv infusion of single ascending doses in healthy subjects.

2.3 Exploratory

To evaluate the immunogenicity of XVR011 after an iv infusion of single ascending doses in healthy subjects.

Strictly Confidential. © 2021, PRA Health Sciences. All rights reserved.

3. INVESTIGATIONAL PLAN

3.1 Overall Study Design and Plan

3.1.1 Type of Study

This will be a Phase 1, randomized, double-blind, single-center, placebo-controlled, single ascending dose (SAD) study in a maximum of 3 groups of 10 healthy male and/or female subjects each:

- Group 1: A single iv infusion of 250 mg XVR011 (n=8) or matching placebo (n=2)
- Group 2: A single iv infusion of 500 mg XVR011 (n=8) or matching placebo (n=2)
- Group 3: A single iv infusion of 1000 mg XVR011 (n=8) or matching placebo (n=2)

The treatments planned in this study are further described in Section 3.4.1.

In each group, subjects will be dosed according to a sentinel dosing design to ensure optimal safety. This means that, in each group, initially 2 subjects will be dosed: 1 subject with XVR011 and 1 subject with placebo. If the safety and tolerability results of the first 24 hours following dosing in the initial subjects are acceptable to the Investigator and the stopping criteria are not met (see Section 3.2.1.1), the remaining 8 subjects (7 active and 1 placebo) of the group will be dosed.

More details concerning this dosing design can be found in Section 3.2.

The study design overview is presented in Figure 1.

Figure 1 Study Design Overview

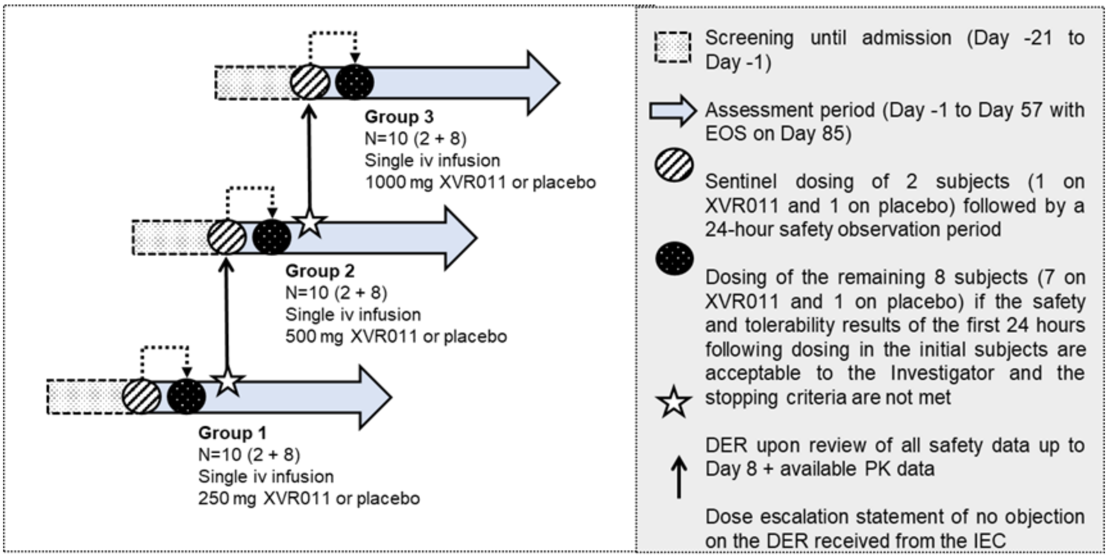

DER=dose escalation report; EOS=end-of-study; IEC=Independent Ethics Committee; iv=intravenous; PK=pharmacokinetic

3.1.2 Screening Period

Subjects will report to the medical screening facility/clinical site for the eligibility screening (see Section 3.3 for inclusion and exclusion criteria) within 21 days prior to drug administration.

Strictly Confidential. © 2021, PRA Health Sciences. All rights reserved.

Subjects will sign the study-specific informed consent form (ICF) prior to any study-specific screening procedures being performed. The written informed consent will be obtained for all subjects regardless of their eligibility for the study; the signed ICFs will be retained and archived at PRA Health Sciences (PRA) and a copy will be provided to the subject.

Eligibility screening will consist of the assessments as presented in the schedule of assessments (Table 1).

3.1.3 Assessment Period

Subjects will be admitted to the clinical research center on Day -1, which is the day prior to Day 1, the day of study drug administration. The next morning (Day 1), XVR011 or placebo will be administered as a single iv infusion. Subjects will be discharged approximately 24 hours after ending the study drug infusion (Day 2) and after completion of the assessments. Subjects will return to the clinical research center for ambulatory visits on Days 8, 15, 29, 43, and 57 (±1 day each).

Assessments during the assessment period will be performed as presented in the schedule of assessments (Table 1).

3.1.4 End of Study/Early Termination

The end of study (EOS) assessments will be performed on Day 85 (±2 days). In case a subject is withdrawn or voluntarily withdraws from the study after having received the study drug, EOS assessments will be performed at an early termination visit as soon as possible.

Assessments during EOS/early termination will be performed as presented in the schedule of assessments in Table 1.

3.2 Discussion of Study Design

3.2.1 Dose Escalation

An escalating-dose study design was chosen for this study to allow considered increase of the dose level after assessment of safety and tolerability of each preceding dose group. A dose escalation report (DER) will be provided by the Investigator to the Independent Ethics Committee (IEC) following completion of each dose level. Upon review of the protocol by the IEC, a decision will be taken whether a statement of no objection following review of the DER from the IEC is required to dose escalate. Based on the nature of XVR011 (ie, a protein), special attention will be paid to assessment of immune reactions and/or infusion reactions.

The study encompasses 3 dose groups, reflecting the dose levels being evaluated in the patient study (EXEVIR0101). There will be 8 subjects receiving active treatment and 2 subjects receiving placebo treatment per dose group. The first 2 subjects will be sentinel subjects, 1 on XVR011 and 1 on placebo treatment, and will be dosed concurrently. An observation period of 24 hours will be respected, and if no adverse reactions occur that meet the stopping criteria (Section 3.2.1.1), then the remaining 8

Strictly Confidential. © 2021, PRA Health Sciences. All rights reserved.

subjects of that dose group will be dosed (7 XVR011 and 1 placebo). The minimum number of subjects required to decide on dose escalation is 8 subjects. Any subjects not randomized (maximum of 2 subjects) may be enrolled at a later stage at the discretion of the Sponsor. Dose escalation dose will only proceed when none of the stopping criteria have been met and when the safety and tolerability up to Day 8 of the last preceding dose group, as well as any significant AEs in earlier groups and available PK data, have been reviewed and considered acceptable to the Investigator and the Sponsor and, if deemed necessary, after a statement of no objection on the DER from the IEC. When applicable, the available PK data will be provided as summary statistics and anonymized individual PK profiles in the DER, if data allow, in order to keep the study blinded. Based on emerging PK and safety data, lower or other intermediate dose(s) may be used, for instance when anticipated that stopping criteria will be met after a planned dose escalation.

3.2.1.1 Stopping Rules for Dose Escalation

3.2.1.1.1 Within-group Stopping Rules

The treatment will be unblinded if a sentinel subject experiences any of the following adverse reactions:

- A serious adverse reaction (ie, a serious AE considered at least possibly related to the study drug administration)
- A ≥Grade 3 nonserious adverse reaction (ie, severe nonserious AEs considered at least possibly related to the study drug administration)

If the treatment is XVR011, then none of the remaining subjects in that dose group will be dosed. If the treatment is placebo, then the remaining subjects in that dose group may be dosed.

3.2.1.1.2 Between-group Stopping Rules

The next dose group will only start after review by the Investigator and Sponsor of all available safety and PK data from the preceding dose group(s) when all subjects in the last preceding dose group have completed the Day 8 assessments. Any additional data beyond Day 8 that are available will also be taken into consideration. Dose escalation to the next group will be halted at any time if 1 of the following circumstances occurs and it is determined by the Investigator that the occurrence is at least possibly related to the administration of study drug:

- Any serious adverse reaction (ie, a serious AE considered at least possibly related to the study drug administration) or ≥Grade 3 infusion-related reaction in 1 or more subject who received XVR011
- ≥Grade 3 nonserious adverse reactions (ie, severe nonserious AEs considered at least possibly related to the study drug administration) in 2 or more subjects who received XVR011, or in 1 subject in the sentinel group of a group who received XVR011
- A Grade 2 (nonserious) adverse reaction belonging to the same system organ class (SOC) in 3 or more subjects who received XVR011
- A Grade 2 (nonserious) adverse reaction across all SOCs in 4 or more subjects who received XVR011

Strictly Confidential. © 2021, PRA Health Sciences. All rights reserved.

- Other findings that, at the discretion of the Investigator and/or Sponsor's Medical Monitor, indicate that further dosing should be stopped

When stopping rules for a group are met, the randomization code for subjects meeting the stopping rules will be unblinded. If after unblinding it is concluded that subjects on active medication meet the stopping rules, dosing in the group will be stopped and no further dose escalation will be performed. If a subsequent integrated analysis of available data leads to the conclusion that further careful escalation is warranted, a substantial amendment is needed before continuation of the study.

3.2.2 Sentinel Dosing

XVR011 is in the early stage of clinical development; hence, a sentinel dosing design in all 3 successive groups has been used to ensure safety. This means that, in each group, initially 2 subjects will be dosed. One of these subjects will receive the active medication XVR011 and the other subject will receive placebo. The subjects will be closely observed by the Investigator for the first 24 hours following study drug administration. The general tolerability of the study drug will be monitored during this time, and the electrocardiogram (ECG) and vital sign recordings will be reviewed. In addition, any reported AEs will be considered by the Investigator. If the stopping criteria are not met (see stopping rules in Section 3.2.1.1), the remaining 8 subjects (7 active and 1 placebo) of the group will be dosed.

3.2.3 Other

The EOS (ie, Day 85, 84 days after study drug administration) is based on the anticipated half-life of 21 days and absence of a duration of action prolonged beyond serum exposure.

Treatment (XVR001 or placebo) will be blinded to the investigational site staff, the subject, and the Sponsor. The purpose of including placebo-treated subjects is to assess whether safety findings observed were due to XVR011 or to study procedures, and not for a formal statistical comparison between active and placebo subjects.

The study will be performed in different groups of subjects since the number of doses to be tested and all assessments associated with these sessions are regarded as too extensive to be performed in a single group of subjects participating repeatedly.

See Section 3.6.4 for sample size determination.

The Investigator will take all the usual precautions necessary for studies at an early stage in the development of a new drug.

Healthy subjects have been chosen as the study population due to the study design and the low risk of clinically significant toxicity at anticipated exposure levels. Moreover, use of healthy subjects as opposed to patients will allow a clearer interpretation of the study results, as there will be no confounding factors resulting from changes in disease state and/or concomitant medications.

Strictly Confidential. © 2021, PRA Health Sciences. All rights reserved.

3.2.4 COVID-19 Risk Mitigation

This study will be conducted in accordance with guidance from the Competent Authority (CA) in the Netherlands (Centrale Commissie Mensgebonden Onderzoek [English translation: Central Committee on Research Involving Human Subjects]) on conducting Phase 1 trials in clinical research units in the Netherlands during the COVID-19 pandemic.

During the entire study, the clinical research center will implement all recommendations issued by the Dutch government with respect to minimizing the risk of disease spreading (eg, social distancing, disinfection, hygiene, and wearing of personal protection equipment by study staff). Details on specific procedures are described in the site specific manual.

In cases where subjects are not able to attend study visits due to an infection with SARS-CoV-2, the Investigator will discuss with the Sponsor potential mitigation approaches (including, but not limited to, extending the visit window, conducting evaluations via video link or phone call, and/or allowing for safety procedures to be conducted at a local facility). The rationale (eg, the specific limitation imposed by the SARS-CoV-2 infection that led to the inability to perform the protocol-specified assessment) and the outcome of the discussion will be documented in the electronic case report form (eCRF).

In addition, the following containment measures will be taken during the study:

- PCR testing for SARS-CoV-2 will be performed at the time points indicated in the schedule of assessments (Table 1).
- If a subject is tested to be SARS-CoV-2 positive on Day -1, the subject will be excluded from participation (see Exclusion Criterion #16) and referred for counseling.
- A subject should not be admitted if there was any known contact with a person who tested positive for SARS-CoV-2 or a COVID-19 patient within 2 weeks prior to admission to the clinical research center (see Exclusion Criterion #16).
- Physical examinations will be limited as much as considered possible by the Investigator.
- If a subject becomes ill and/or is tested to be SARS-CoV-2 positive after administration of the study drug, the subject will be isolated from other study subjects. Per standard measures for follow-up of SARS-CoV-2 in PCR-positive study subjects, subjects should be discharged from the clinical research center as soon as possible. After completion of an appropriate quarantine period according to local regulations and a negative PCR test result, the subject may return to the clinical research center to complete remaining (follow-up) assessments.

These COVID-19 risk mitigation measures will be kept in place for as long as the pandemic is ongoing, as defined by country- and site-specific regulations. Once the pandemic has ended, SARS-CoV-2 testing may be omitted at the discretion of the Investigator.

Strictly Confidential. © 2021, PRA Health Sciences. All rights reserved.

To avoid potential interference with immunity induced by vaccination with vaccines that use the SARS-CoV-2 Spike protein or elements thereof, subjects must either be fully vaccinated against COVID-19 at least 14 days prior to study drug administration or be willing to delay planned vaccination until 90 days after study drug administration (see Inclusion Criterion #6).

3.3 Selection of Study Population

A total of 30 healthy male and/or female subjects are planned to be included in the study.

3.3.1 Inclusion Criteria

Note: Restrictions that apply to the period after admission are described in Section 3.4.8, except when they concern a statement of willingness.

The following inclusion criteria must be met for a subject to be eligible for inclusion in the study:

1. Sex : Male or female; females may be of childbearing potential or of nonchildbearing potential (ie, surgically sterilized, physiologically incapable of becoming pregnant, or at least 1 year postmenopausal [amenorrhea duration of 12 consecutive months and confirmed by a serum follicle-stimulating hormone {FSH} test at screening]).
2. Age : 18 to 65 years, inclusive, at screening.
3. Body mass index (BMI) : 18.0 to 30.0 kg/m^2, inclusive, at screening.
4. Weight : ≥50 kg.
5. In good physical and mental health on the basis of medical history, physical examination, clinical laboratory, ECG, and vital signs, as judged by the Investigator.
6. Subject is fully vaccinated against COVID-19 at least 14 days prior to study drug administration or is willing to delay his/her planned vaccination until 90 days after study drug administration.
7. Females must not be pregnant or lactating. Nonpregnancy will be confirmed for all females by a serum pregnancy test at screening, at admission, and at the EOS visit.
8. Female subjects of childbearing potential who have a fertile male sexual partner must agree to use adequate contraception from screening until 90 days after study drug administration. Adequate contraception is defined as using hormonal contraceptives or an intrauterine device combined with at least 1 of the following forms of contraception: a diaphragm, a cervical cap, or a condom. Total abstinence from heterosexual intercourse, in accordance with the lifestyle of the subject, is also acceptable.
9. Male subjects, if not surgically sterilized, must agree to use adequate contraception and not donate sperm from admission to the clinical research center until 90 days after study drug administration. Adequate contraception for the male subject (and his female partner, if she is of childbearing potential) is defined as using hormonal contraceptives or an intrauterine device combined with at least 1 of the following forms of contraception: a diaphragm, a cervical cap, or a condom. Total abstinence

Strictly Confidential. © 2021, PRA Health Sciences. All rights reserved.

from heterosexual intercourse, in accordance with the lifestyle of the subject, is also acceptable.

10. All prescribed medication must have been stopped at least 14 days prior to admission to the clinical research center. An exception is made for hormonal contraceptives, which may be used throughout the study.
11. All over-the-counter medication, vitamin preparations and other food supplements, or herbal medications (eg, St. John's wort) must have been stopped at least 7 days prior to admission to the clinical research center. An exception is made for paracetamol, which is allowed up to admission to the clinical research center.
12. Ability and willingness to abstain from alcohol from 48 hours (2 days) prior to screening and admission to the clinical research center.
13. Ability and willingness to sign the ICF.

3.3.2 Exclusion Criteria

Note: Restrictions that apply to the period after admission are described in Section 3.4.8, except when they concern a statement of willingness.

A subject who meets any of the following exclusion criteria will not be eligible for inclusion in the study:

1. Previous participation in the current study.
2. Employee of PRA or the Sponsor.
3. History of relevant drug allergies (eg, allergy or hypersensitivity reaction to any monoclonal antibody or to any components of the study drug) and/or food allergies.
4. Using tobacco products within 60 days prior to study drug administration.
5. History of alcohol abuse or drug addiction within 12 months prior to the screening visit (including soft drugs like cannabis products).
6. Positive drug and alcohol screen (opiates, methadone, cocaine, amphetamines [including ecstasy], cannabinoids, barbiturates, benzodiazepines, gamma-hydroxybutyric acid, tricyclic antidepressants, and alcohol) at screening or admission to the clinical research center.
7. Average intake of more than 24 units of alcohol per week (1 unit of alcohol equals approximately 250 mL of beer, 100 mL of wine, or 35 mL of spirits).
8. Positive screen for hepatitis B surface antigen (HBsAg), hepatitis C virus (HCV) antibodies, or HIV 1 and 2 antibodies.
9. Participation in a drug study within 30 days prior to drug administration in the current study, or 90 days if the investigational medicinal product was a monoclonal antibody. Participation in more than 4 other drug studies in the 12 months prior to drug administration in the current study.
10. Donation or loss of more than 450 mL of blood within 60 days prior to drug administration, or of more than 1.5 L of blood for male subjects/more than 1.0 L of blood for female subjects within 10 months prior to study drug administration.
11. Significant and/or acute illness within 14 days prior to study drug administration that may impact safety assessments, in the opinion of the Investigator.
12. History of malignancy within 5 years before the screening visit (exceptions are squamous and basal cell carcinomas of the skin or malignancy that, in the opinion of the Investigator with written concurrence with the Sponsor's Medical Monitor, is considered cured with minimal risk of recurrence).

Strictly Confidential. © 2021, PRA Health Sciences. All rights reserved.

13. History of COVID-19 infection within 2 months prior to the date of the screening visit.
14. History of increased bleeding risk.
15. Unsuitable veins for infusion or blood sampling.
16. Positive nasopharyngeal PCR test for SARS-CoV-2 on Day -1 or if there was any known contact with a person who tested positive for SARS-CoV-2 or with a COVID-19 patient within 2 weeks prior to admission to the clinical research center.
17. Any condition that would jeopardize the subject's appropriate participation in this study.

Please note that subjects should refrain from consumption of any foods containing poppy seeds within 48 hours (2 days) prior to screening and admission to the clinical research center to avoid false positive drug screen results. The use of alcohol is not allowed within 48 hours (2 days) prior to admission to the clinical research center and during the stay in the clinical research center. In addition, subjects should refrain from strenuous exercise within 96 hours (4 days) prior to screening and admission, as this could result in abnormal clinical laboratory values.

3.3.3 Removal of Subjects from Assessment

Participation in the study is strictly voluntary. A subject has the right to withdraw from the study at any time for any reason.

The Investigator has the right to terminate participation of a subject for any of the following reasons: difficulties in obtaining blood samples, violation of the protocol, severe AEs or serious adverse events (SAEs), or for any other reason relating to the subject's safety or the integrity of the study data.

If a subject is withdrawn or voluntarily withdraws from the study prematurely, the Sponsor/study monitor will be informed immediately. If there is a medical reason for withdrawal, the subject will remain under the supervision of the Investigator until satisfactory health has returned.

A subject who is withdrawn or voluntarily withdraws from the study for any reason after having received the study drug, whether related to the study drug or not, will be considered an early-termination subject. If a subject is withdrawn for a reason related to the study drug, according to the judgment of the Investigator, the early-termination subject will not be replaced. If a subject is withdrawn for a reason not related to the study drug, the early-termination subject may be replaced after mutual agreement between the Sponsor and PRA. The decision regarding the replacement of subjects will be documented.

PRA will make every effort to ensure that early-termination subjects who have received the study drug complete the EOS assessments.

After signing the ICF, subjects who drop out or withdraw for any reason without successfully completing all screening evaluations as well as subjects who drop out or withdraw prior to administration of the study drug will be considered screening failures.

Strictly Confidential. © 2021, PRA Health Sciences. All rights reserved.

3.3.3.1 Stopping Rules for Individual Subjects

The treatment is administered as a single iv infusion. As with any protein, infusion reactions/hypersensitivity reactions may occur. This may vary from mild to moderate signs and symptoms, but may also be a severe or life-threatening anaphylactic/anaphylactoid reaction. Signs and symptoms may include hypotension, tachycardia, bronchospasm, dyspnea, edema/angioedema, dizziness, headache, nausea, abdominal pain, fever, and rash. Therefore, stopping rules apply for interrupting and/or permanently discontinuing the infusion as presented in Table 2.

Table 2 Guidance in Case of an Infusion Reaction Based on Severity of the Reaction

Infusion-Related Reaction	Study Treatment Actions	Other Treatment (See Also Institutional Standard Practice for Infusion Reactions)
Grade 1 - mild	Interrupt the infusion and restart at the discretion of the Investigator with a reduced rate of infusion. If the reaction worsens after restarting, the infusion must be permanently stopped.	Consider symptomatic treatment.
Grade 2 - moderate	Permanently stop infusion.	Administer symptomatic treatment (eg, antihistamines, NSAIDs).
Grade 3 - severe	Permanently stop infusion.	Administer antihistamines, corticosteroids, bronchodilators, iv fluids, vasopressors, and other treatments as per Investigator judgment.
Grade 4 - life-threatening	Permanently stop infusion.	Administer antihistamines, corticosteroids, bronchodilators, iv fluids, vasopressors and other life support treatments as per Investigator judgment.

iv=intravenous; NSAID=nonsteroidal anti-inflammatory drug

Severity of infusion-related reactions will be defined using the most current version of the Common Terminology Criteria for Adverse Events (CTCAE) 5-point scale (see Appendix 8.2.1).

3.4 Treatments

3.4.1 Treatments Administered

The following treatments are planned to be administered according to the randomization code (see Section 3.4.3):

- Group 1: A single iv infusion of 250 mg XVR011 or matching placebo
- Group 2: A single iv infusion of 500 mg XVR011 or matching placebo
- Group 3: A single iv infusion of 1000 mg XVR011 or matching placebo

Based on evolving data, the dose levels of Groups 2 and 3 may be adjusted. Escalation to the next dose will be no more than 2-fold. The dose will be administered using an iv

Strictly Confidential. © 2021, PRA Health Sciences. All rights reserved.

bag containing 0.9% (w/v) sodium chloride sterile solution, over an infusion time of 90 minutes (Table 3), and delivered through an iv administration set with a sterile, nonpyrogenic, low-protein binding 0.2 µm in-line or add-on filter.

Table 3 Intravenous Infusion Details

	XVR011 Dose	Infusion Volume	XVR011 Concentration	Infusion Time	Infusion Rate	
	mg	mL	mg/mL	min	mg/min	mL/min
Group 1	250	100	2.50	90	2.78	1.11
Group 2	500	100	5.00	90	5.56	1.11
Group 3	1000	100	10.00	90	11.11	1.11

3.4.2 Identity of Investigational Product

Active Medication

Active substance : XVR011, a humanized heavy chain-only antibody fragment linked to an effector function partially silenced, Fc part of a human IgG1

Activity : XVR011 binds to the spike protein of SARS-CoV-2 and, through steric hindrance, prevents the virus from binding to the ACE2 receptor, thereby preventing it from entering the cell

In development for : COVID-19; it is intended as an antiviral treatment to halt infection in the patient and thereby reduce the chance of progression to more severe disease

Strength : 50 mg/mL

Dosage form : Solution for iv infusion

Manufacturer : Provided by the Sponsor

Placebo (Visually Matching Active Medication)

Active substance : 0.9% (w/v) sodium chloride sterile solution

Activity : Not applicable

Strength : Not applicable

Dosage form : Solution for iv infusion

Manufacturer : Sourced by pharmacy at PRA

XVR011 will be provided by the Sponsor in 20 mL neutral type I glass Schott vials (filled with 500 mg of XVR011 in 10 mL extractable volume). Each vial will be labeled per country requirement by the PRA Pharmacy. Commercially available 0.9% (w/v) sodium chloride iv bags and accessories need to be sourced by the PRA Pharmacy.

For details concerning drug storage and drug accountability, see Appendix 8.1.

3.4.3 Method of Assigning Subjects to Treatment Groups

After obtaining informed consent, subjects will receive a screening number and will be screened according to the inclusion and exclusion criteria. Subjects who have met all eligibility criteria will receive a subject number upon inclusion in the study (Subject Numbers 01 to 30). They will receive the subject number just prior to dosing according

Strictly Confidential. © 2021, PRA Health Sciences. All rights reserved.

to the randomization code. The subject number will ensure identification throughout the study after study drug administration. Replacement subjects will receive the number of the subject to be replaced, increased by 100 (eg, 101 would be the replacement number for Subject 01), and will be administered the same treatment.

Subjects will be assigned to a dose group based on their availability while trying to admit subjects with equal sex distribution across dose groups. Treatments within a dose group will be assigned according to the randomization code. Sentinel dosing will be applied in each group. For the 2 sentinel subjects, randomization will ensure that 1 subject will receive XVR011 and the other subject will receive placebo. For the remaining 8 subjects in each group, randomization will ensure that 7 subjects will receive XVR011 and 1 subject will receive placebo.

The randomization code will be produced by the Biostatistics Department of PRA. The PRA study biostatistician will create a draft randomization list and a peer biostatistician will review and approve that draft list. The final randomization list will be created, reviewed, and approved by 2 designated biostatisticians who are not members of the study team. After the final randomization list has been approved, it will be transferred to the PRA Pharmacy and kept in a restricted area to which only the PRA Pharmacy staff has access. Two unblinded biostatisticians will also have access to the randomization list. Code break envelopes will be prepared and made available to the clinical staff for emergency unblinding (see Section 3.4.7).

After signing the ICF, subjects who drop out or withdraw for any reason without successfully completing all screening evaluations as well as subjects who drop out or withdraw prior to administration of the study drug will be considered screening failures. Such subjects, and also subjects who are eligible for inclusion in the study but do not receive the study drug (eg, reserve subjects), will not receive a subject number, and only applicable data will be entered in the eCRFs.

3.4.4 Selection of Doses in the Study

The starting dose in the patient study (EXEVIR0101; ClinicalTrials.gov Identifier: NCT04884295) is 250 mg based on a pharmacokinetic-pharmacodynamic (PKPD) model demonstrating an exposure response relationship for serum concentrations and viral load in a hamster model of disease. Simulated human PK profiles were generated based on a nonhuman primate PK study. A serum threshold concentration was determined, which was associated with a 4-fold log 10 decrease in viral load. This combined with the PK profiles supports the starting dose of 250 mg as giving serum concentrations above the threshold for 7 days at the median. An expected minimally efficacious dose is necessary for the patient study. The medium and higher dose levels of 500 mg and 1000 mg reflect serum concentrations that are above the 97.5^{th} percentile of the serum concentration threshold for at least 7 days.

These dose levels will be evaluated in the proposed 3 dose groups of this healthy subject study (EXEVIR0102). The justification of 250 mg as the starting dose in healthy subjects is derived from the lack of safety signal arising in the nonclinical studies, including the nonhuman primate PK study that evaluated a highest dose of 50 mg/kg. The human equivalent dose for this is in the order of 1000 mg, the highest dose level proposed to

Strictly Confidential. © 2021, PRA Health Sciences. All rights reserved.

be evaluated in the last dose group of this study. The starting dose of 250 mg is a factor 4× lower, which is considered an acceptable reduction for this type of compound. In addition, the iv infusion will be administered over 90 minutes. This means that at the starting dose of 250 mg, XVR011 is administered at a rate of 2.78 mg/minute. Subjects will be carefully monitored throughout the infusion, allowing for rapid intervention should infusion-related reactions occur. Each dose group will also have 2 sentinel subjects (1 XVR011 and 1 placebo) with a 24-hour postdose observation period prior to dosing the remaining subjects in that group.

3.4.5 Timing of Doses in the Study

On Day 1, the study drug will be administered to subjects between 08:00 hours and 12:00 hours in the morning. Breakfast is not standardized and will be given within a maximum of 1 hour before dosing and should be completed before dosing.

The subject will be closely monitored by the clinical staff according to standard site procedures during the iv infusion and for at least 120 minutes afterwards. Sentinel subjects should be monitored for 24 hours after the infusion (Section 3.2.2).

3.4.6 Meals During the Study

A fasting period of at least 4 hours is required before obtaining clinical laboratory blood samples at the timepoints for clinical laboratory specified in the schedule of assessment (Table 1).

With the exception of the restrictions described in Section 3.4.8 and the timing described in Section 3.4.5, there are no special requirements related to food and beverage intake. When not fasting, meals and snacks will be provided according to PRA standard operating procedures (SOPs).

3.4.7 Blinding

In each group, 8 subjects will be randomly assigned to receive a single iv infusion containing XVR011 on Day 1, and 2 subjects will be randomly assigned to receive a single iv infusion without XVR011 (placebo) on Day 1 according to the randomization code.

The following controls will be employed to maintain the double-blind status of the study:

- The iv infusion bags containing active drug or placebo will be indistinguishable in appearance. In the event that a color difference between the XVR011 solution and placebo is observed, the infusion iv bags and infusion lines will be blinded by the pharmacist; eg, by adding yellow foil to the outside of the infusion bags.
- The randomization code will be provided to the pharmacist at PRA for dispensing purposes and kept in the pharmacy, accessible to the pharmacist and the pharmacy technician only.

Individual code break envelopes will be provided for all subjects by PRA. Each sealed envelope containing the randomization code will be kept in a medication storage room, which is locked with restricted access. To manage the subject's condition in case of a medical emergency, the Investigator is allowed to break the code to determine whether

Strictly Confidential. © 2021, PRA Health Sciences. All rights reserved.

a subject received XVR011 or placebo. If opened, the name of the person who opened it, the date and time of opening, and the reason for opening must be written on the envelope. The Sponsor will be informed in case of unblinding.

The laboratory where the PK samples will be analyzed will receive a copy of the randomization code from the pharmacy since only samples of subjects who have received the active drug XVR011 will be analyzed.

3.4.8 Concomitant Medication and Other Restrictions During the Study

Note: Restrictions that apply to the period before admission are described in Section 3.3.1 and Section 3.3.2.

The use of all prescribed medication is not allowed from admission to the clinical research center until the EOS visit. An exception is made for hormonal contraceptives, which are allowed throughout the study. The use of all over-the-counter medication, vitamin preparations and other food supplements, or herbal medications (eg, St. John's wort) is not allowed from admission to the clinical research center until the EOS visit. An exception is made for paracetamol; from admission onwards, the Investigator may permit a limited amount of paracetamol for the treatment of headache or any other pain. Other medication to treat AEs may only be prescribed if deemed necessary by the Investigator. If medication is used, the name of the drug, the dose, dosage regimen, and the start and end dates will be recorded in the eCRF.

The use of tobacco products is not allowed from admission to the clinical research center until the EOS visit.

The use of alcohol is not allowed within 48 hours (2 days) prior to admission to the clinical research center and during the stay in the clinical research center.

Strenuous exercise is not allowed within 96 hours (4 days) prior to admission and on days when clinical laboratory samples are taken (ie, Days 8, 15, and 29, and the EOS visit), as this could result in abnormal clinical laboratory values. It is also not allowed during the stay in the clinical research center.

Subjects should not consume any foods containing poppy seeds within 48 hours (2 days) prior to admission to the clinical research center as this could cause false positive drug screen results.

Male subjects, if not surgically sterilized, are required to use adequate contraception (see description below) and not donate sperm from admission to the clinical research center until 90 days after study drug administration.

Female subjects of childbearing potential with a fertile male sexual partner are required to use adequate contraception (see description below) from screening until 90 days after study drug administration.

Adequate contraception is defined as using hormonal contraceptives or an intrauterine device combined with at least 1 of the following forms of contraception: a diaphragm, a

Strictly Confidential. © 2021, PRA Health Sciences. All rights reserved.

cervical cap, or a condom. Total abstinence from heterosexual intercourse, in accordance with the lifestyle of the subject, is also acceptable.

Subjects must not donate blood during the study until the EOS visit (other than the blood sampling planned for this study).

3.4.9 Treatment Compliance

The study drug will be administered in the clinical research center. To ensure treatment compliance, administration of the study drug will be supervised by the Investigator or authorized designee. Compliance will be further confirmed by bioanalytical assessment of XVR011 in serum samples (see Section 3.5.4).

The following information regarding study drug administration will be recorded in the eCRF:

- Infusion start/stop times.
- The iv volume administered; it will be documented if the full volume of the study drug infusion was administered. If the full volume was not administered, eg, in case of interruption due to an infusion-related reaction, then an approximation of the infused volume must be made and documented.
- The infusion rate.

3.5 Pharmacokinetic and Safety Measurements and Variables

3.5.1 PK and Safety Measurements

The schedule of assessments is presented in Table 1.

The timing of assessments may be changed based on data from preceding groups. Based on emerging data, fewer assessments may be performed for all subjects while not changing the duration of stay, changing the number of visits, or exceeding the maximum allowed volume of blood drawn in this study (see Section 3.5.1.3). Additional assessments, including specialist referrals, may be performed if it is considered clinically necessary by the Investigator for individuals on a case-by-case basis.

3.5.1.1 PK Measurements

At the time points defined in the schedule of assessments (Table 1), blood samples will be taken for analysis of XVR011 in serum samples. The blood samples will be taken via an indwelling iv catheter or by direct venipuncture. The exact times of blood sampling will be recorded in the eCRF.

The last time point for PK sampling was determined using the lower limit of quantification of the PK assay (0.2 µg/mL) and considering that XVR011 will still be detectable out to 84 days postdose.

Details on sample collection, handling, storage, and shipping will be described in the laboratory manual prepared by PRA.

Strictly Confidential. © 2021, PRA Health Sciences. All rights reserved.

3.5.1.2 Safety and Tolerability Measurements

Safety and tolerability assessments will consist of AEs, clinical laboratory, vital signs, 12-lead ECG, physical examination, local tolerability, and immunogenicity measurements. Assessments will be performed in accordance with the schedule of assessments (Table 1).

3.5.1.2.1 AEs

AEs will be recorded from admission until completion of the EOS visit. AEs occurring between screening and admission (as reported by the subjects at admission) will be recorded as medical history. Any clinically significant observations, as determined by the Investigator, in results of clinical laboratory, local tolerability, vital signs, 12-lead ECGs, or physical examinations will be recorded as AEs.

A treatment-emergent AE (TEAE) is defined as any event not present prior to administration of the study drug or any event already present that worsens in either severity or frequency following exposure to the study drug. An AE that occurs prior to administration of the study drug will be considered a pretreatment AE.

At several time points before and after study drug administration, subjects will be asked nonleading questions to determine the occurrence of AEs. Subjects will be asked in general terms about any AEs at regular intervals during the study. In addition, all AEs reported spontaneously during the course of the study will be recorded. All answers will be interpreted by the Investigator using the Medical Dictionary for Regulatory Activities (MedDRA; most recent version) for AEs and will be recorded in the eCRF.

The severity of the AEs will be rated using the most current version of the CTCAE 5-point scale as mild (Grade 1), moderate (Grade 2), severe (Grade 3), life-threatening (Grade 4), or death (Grade 5); the relationship between the AEs and the study drug will be indicated as related or not related. Details on rating the severity of AEs and relationship to the study drug are given in Appendix 8.2.1.

Pregnancy of female subjects and female partners of male subjects will be monitored along with follow-up, if warranted (see Appendix 8.3).

3.5.1.2.2 Clinical Laboratory

Blood and urine samples for clinical laboratory assessments will be collected according to PRA SOPs.

The following parameters will be measured:

- Clinical chemistry (serum quantitatively): total bilirubin, alkaline phosphatase, gamma glutamyl transferase, aspartate aminotransferase, alanine aminotransferase, lactate dehydrogenase, creatinine, urea, total protein, glucose, inorganic phosphate, sodium, potassium, calcium, chloride, and C-reactive protein.
- Hematology (blood quantitatively): leukocytes, erythrocytes, hemoglobin, hematocrit, thrombocytes, absolute partial automated differentiation (lymphocytes, monocytes, eosinophils, basophils, and neutrophils), mean corpuscular volume, mean corpuscular hemoglobin, and mean corpuscular hemoglobin concentration.

Strictly Confidential. © 2021, PRA Health Sciences. All rights reserved.

- Coagulation (blood quantitatively): prothrombin time (reported in seconds and as international normalized ratio), activated partial thromboplastin time, and fibrinogen.
- Urinalysis (urine qualitatively): hemoglobin, urobilinogen, ketones, glucose, and protein.
- Serology: HBsAg, HCV antibodies, and HIV 1 and 2 antibodies.
- Drug and alcohol screen: opiates, methadone, cocaine, amphetamines (including ecstasy), cannabinoids, barbiturates, benzodiazepines, gamma-hydroxybutyric acid, tricyclic antidepressants, and alcohol.
- Pregnancy test (females only): β-human chorionic gonadotropin in serum.
- FSH test (females only).
- Nasal and throat mucosal cell samples will be collected according to PRA work instructions detailed in the site specific manual. The samples will be tested for SARS-CoV-2 virus using PCR tests.

In case of an infusion-related reaction that is possibly related to complement activation (fast onset and symptoms such as flushing, rash, dyspnea, chest pain, back pain, and/or subjective distress), additional serum samples will be taken during the reaction and after cessation of the reaction. The serum samples will be stored frozen for potential analysis of, eg, complement activity.

In case of unexplained or unexpected clinical laboratory test values, the tests will be repeated as soon as possible and followed up until the results have returned to the normal range and/or an adequate explanation for the abnormality is found. The clinical laboratory will clearly mark all laboratory test values that are outside the normal range, and the Investigator will indicate which of these deviations are clinically significant. Clinically significant laboratory result deviations will be recorded as AEs, and the relationship to the treatment will be indicated (see also Appendix 8.2.1).

Details on sample collection, handling, storage, and shipping will be described in the laboratory manual prepared by PRA.

3.5.1.2.3 Vital Signs

Vital signs include systolic and diastolic BP, heart rate, body temperature, and respiratory rate. Systolic and diastolic blood pressure and heart rate are to be recorded after the subject has been resting for at least 5 minutes in the supine position. These assessments will be made using an automated device whenever possible. Body temperature and respiratory rate will also be measured.

3.5.1.2.4 ECGs

A standard 12-lead ECG will be recorded after the subject has been resting for at least 5 minutes in the supine position. The ECG will be recorded using an ECG machine equipped with computer-based interval measurements. The following ECG parameters will be recorded: heart rate, PR-interval, QRS-duration, QT-interval, QTc-interval (Fridericia's), and the interpretation of the ECG profile by the Investigator.

Strictly Confidential. © 2021, PRA Health Sciences. All rights reserved.

3.5.1.2.5 Physical Examination

Complete and symptom-driven physical examinations will be performed according to PRA SOPs. In addition, body weight and height will be measured according to PRA SOPs.

3.5.1.2.6 Local Tolerability

The Investigator or designee will examine the infusion site at regular time points to assess the local tolerability of the XVR011 infusions at the infusion site. Any potential infusion site reactions will be recorded as an event in the AE eCRF.

3.5.1.2.7 Immunogenicity Measurements (Exploratory)

Blood samples will be taken into serum tubes to determine the presence of (neutralizing) antidrug antibodies (ADAs). The blood samples will be taken via an indwelling iv catheter or by direct venipuncture. The exact times of blood sampling will be recorded in the eCRF.

Details on sample collection, handling, storage, and shipping will be described in the laboratory manual prepared by PRA.

3.5.1.3 Total of Blood Volume

Table 4 presents the number and volume of blood samples and the total volume of blood that will be collected throughout the study per subject.

If deemed necessary by the Investigator or the Sponsor, the number and/or volume of blood samples per assessment may be increased, as long as the total volume of blood drawn for a subject does not surpass 500 mL (except when extra blood samples need to be taken for safety reasons).

Table 4 Number and Volume of Blood Samples and Total Blood Volume Collected per Subject

Assessment	Maximum # Samples	Volume of Blood per Sample (mL)	Total Volume of Blood (mL)
Pharmacokinetics XVR011 in Serum	12	2	24
Clinical Chemistry	8	3.5	28
Hematology	8	3	24
Coagulation	8	4.5	36
Serology	1	5	5
Immunogenicity	3	4.5	13.5
Total Volume of Blood Drawn			**130.5**

3.5.2 Appropriateness of Measurements

The assessments that will be made in this study are standard and generally recognized as reliable, accurate, and relevant.

Strictly Confidential. © 2021, PRA Health Sciences. All rights reserved.

3.5.2.1 Timing of Assessments

For PK, predose samples will be obtained between waking up and the start of the iv infusion. Postdose samples up to 20 minutes after the start of the iv infusion will be obtained with a time window of ±1 minute. Thereafter, postdose samples will be obtained with time margins of ±5% of the time that has passed since the start of the iv infusion.

For safety assessments, predose assessments will be performed between waking up and the start of the iv infusion. For safety assessments up to 2.5 hours after the start of the iv infusion, a time window of ±15 minutes is allowed. Thereafter, serial postdose assessments (eg, multiple assessments within any given day) will be performed with time margins of ±10% of the time that has passed since the start of the iv infusion.

When assessments are planned for the same time, the order of the assessments should be arranged in such a way that PK blood sampling will be done after the ECG and vital signs recordings have been conducted, with PK blood sampling exactly on time.

3.5.3 PK and Safety Variables

3.5.3.1 PK Variables

The PK variables include the serum concentrations of XVR011 and their PK parameters. The PK parameters for XVR011 will be calculated by using noncompartmental analysis (performed by PRA) from the serum concentration-time data for XVR011 and will include, but are not limited to, the parameters as given in Table 5. A complete list of the PK parameters will be provided in the statistical analysis plan (SAP).

Table 5 PK Parameters

Parameter	Description
C_{pre}	Predose serum concentration
C_{max}	Maximum observed serum concentration
t_{max}	Time to attain maximum observed serum concentration
$AUC_{0\text{-Day 8}}$	Area under the serum concentration-time curve from time 0 to Day 8
$AUC_{0\text{-}t}$	Area under the serum concentration-time curve up to time t, where t is the last point with concentrations above the lower limit of quantification
$AUC_{0\text{-inf}}$	Area under the serum concentration-time curve from time 0 to infinity
$\%AUC_{extra}$	Percentage of estimated part for the calculation of $AUC_{0\text{-inf}}$
k_{el}	Terminal elimination rate constant
$t_{1/2}$	Terminal elimination half-life
CL	Clearance
V_z	Volume of distribution at terminal phase
MRT	Mean residence time
Time of serum concentration above 26.9 µg/mL	
Time of serum concentration above 50.4 µg/mL	

3.5.3.2 Safety Variables

The safety variables to be measured include, but are not limited to, the variables given below. A complete list of safety variables will be provided in the SAP.

Strictly Confidential. © 2021, PRA Health Sciences. All rights reserved.

- AEs
- Clinical laboratory
- Vital signs
- 12-lead ECG
- Physical examination
- Local tolerability
- Immunogenicity (exploratory)

3.5.4 Drug Concentration Measurements

The analysis of XVR011 in serum samples will be performed at the LGC Bioanalytical Laboratory using a validated mass spectrometry method. The bioanalytical report for the determinations will be included as an appendix in the clinical study report (CSR).

3.5.5 Retention of Blood and Urine Samples

Blood and urine samples remaining after clinical laboratory assessments have been performed will not be stored for future use and will be destroyed after analysis as per laboratory procedure.

Blood samples remaining after PK and immunogenicity assessments have been performed will be stored at a facility selected by the Sponsor to be used for research purposes such as evaluation of the activity of XVR011, identification of exploratory biomarkers that are predictive of activity, or other exploratory evaluations that may help to characterize the molecular mechanisms of XVR011. The samples will be stored for a maximum period of 3 years, after which the samples will be destroyed.

3.6 Statistical Procedures and Determination of Sample Size

3.6.1 Analysis Sets

3.6.1.1 Safety Set

The Safety Set includes all subjects who have started infusion with XVR011. Subjects will be analyzed according to the study treatment they actually received. A precise definition of "actually received" will be added in the SAP.

3.6.1.2 PK Set

The PK Set includes all subjects in the safety analysis set with sufficient bioanalytical assessment results to calculate reliable estimates of the PK parameters.

3.6.2 Statistical and Analytical Plan for Pharmacokinetic, Safety, and Exploratory Evaluation

A SAP will be generated by the Biostatistics Department of PRA; the SAP will be finalized prior to database lock. Full details of the analysis to be performed will be included in the SAP. Any deviation from the SAP will be reported in the section "Changes in Planned Analysis" in the CSR.

Strictly Confidential. © 2021, PRA Health Sciences. All rights reserved.

3.6.2.1 PK Evaluation

All XVR011 concentrations and PK parameters will be listed and summarized using descriptive statistics in tabular and/or graphical form. Descriptive statistics will include: n, mean, SD, minimum, median, maximum, geometric mean, and coefficient of variation. An exception to this is t_{max}, where median, minimum, and maximum will be presented.

A power model on C_{max}, AUC_{0-t}, and AUC_{0-inf} will be performed to provide estimates for the different dose levels. Selected PK parameters will be compared across each dose level to assess dose-proportionality.

The PK data from the current study will be used in population-PK analysis performed by the Sponsor. This evaluation will not be included in the CSR.

3.6.2.2 Evaluation of Safety and Tolerability

3.6.2.2.1 AEs

A listing of all individual AEs will be provided. Summary tables of TEAEs will be presented by SOC based on the MedDRA terminology list (preferred terms): 1 containing the number of TEAEs (frequency of occurrence and number and percentage of subjects experiencing the event) by treatment and 1 containing the number of drug-related TEAEs (frequency of occurrence and number and percentage of subjects experiencing the event) per treatment. Additional tables of total counts by treatment and relationship and by treatment and severity will be given.

The 6 subjects who receive placebo will be considered as a single placebo group and will be used as the control group for evaluation of AEs.

3.6.2.2.2 Clinical Laboratory

Clinical laboratory data will be listed accompanied by an indication if the parameter is outside the reference range. A summary of all data outside the reference range of the clinical laboratory will be provided. Clinical laboratory data will be presented descriptively, where applicable.

3.6.2.2.3 Vital Signs, ECGs, and Physical Examinations

Vital signs, ECG parameters, and physical examination findings will be listed and presented descriptively, where applicable.

3.6.2.2.4 Immunogenicity Measurements (Exploratory)

Levels of ADAs will be reported as continuous endpoints.

If a sizable number of subjects have ADA formation, the relationship between ADA positive and ADA negative subjects with regard to AUC and time of serum concentration above 26.9 µg/mL and above 50.4 µg/mL will be explored.

Strictly Confidential. © 2021, PRA Health Sciences. All rights reserved.

3.6.3 Interim Analysis

When the last subject has completed the Day 8 assessments, a topline analysis of all available and cleaned safety and PK data may be performed. Planned analyses to be included in the topline will be listed in the SAP.

3.6.4 Determination of Sample Size

The sample size has been selected to provide information on safety, tolerability, PK, and immunogenicity following single iv infusion of XVR011. Any p-values to be calculated according to the SAP will be interpreted in the perspective of the explorative character of this study.

3.7 Data Quality Assurance

The study may be audited by the Quality Assurance Department at PRA to assess adherence to the clinical study protocol (CSP) and Quality System. During the conduct of the study, process-related audits may be performed. An audit certificate will be provided in the appendices of the final CSR outlining any audits and other related activities performed.

The clinical research site will be monitored by the study monitor to ensure correct performance of the study procedures and to assure that the study is conducted according to the relevant regulatory requirements. The eCRF entries will be verified with the source documentation, if applicable (in some cases data are captured directly on the eCRF; in those cases, source data verification is not necessary).

Regulatory authorities, the IEC, and/or the Sponsor’s clinical quality assurance group may request access to all source documents, eCRFs, and other study documentation for on-site audit or inspection. Direct access to these documents must be guaranteed by the Investigator, who must provide support at all times for these activities.

Quality control principles will be applied throughout the performance of this study. Review procedures will be followed at PRA for all documents that are generated in relation with the study.

An explanation will be given for all missing, unused, and spurious data in the relevant sections of the CSR.

Strictly Confidential. © 2021, PRA Health Sciences. All rights reserved.

4. ETHICS

4.1 IEC

The submission package including the CSP and the ICFs will be submitted for review and approval by the IEC of the foundation “Beoordeling Ethiek Biomedisch Onderzoek” (English translation: “Evaluation of Ethics in Biomedical Research”) (Dr. Nassaulaan 10, 9401 HK Assen, the Netherlands) prior to the eligibility screening. The composition of the IEC is in accordance with the recommendations of the WHO, the International Council for Harmonisation (ICH) E6(R2) Guideline for Good Clinical Practice (GCP), [12] and the EU Clinical Trial Directive (CTD) 2001/20/EC [13] (see below). The submission package will also be submitted to the CA in the Netherlands for a statement of no objection.

PRA will keep the IEC informed about the progress of the study. All changes in research activities and all unanticipated problems involving risks to human subjects will be immediately reported to the IEC. In accordance with Section 10, Subsection 1 of the Dutch law on Medical Research in Human Subjects (WMO, revised Dec 2015), [14] PRA will inform the subjects and the IEC if anything occurs on the basis of which it appears that the disadvantages of participation may be significantly greater than was foreseen in the research proposal, or if further recruitment of subjects in the study has been put on hold for that reason, whichever occurs first. The study may be suspended pending further review by the IEC, except insofar as suspension would jeopardize the subjects’ health. PRA will take care that all subjects are kept informed.

No changes will be made to the study without IEC approval, except when required to eliminate apparent immediate hazards to human subjects.

A DER will be provided by the Investigator to the IEC following completion of each dose level. Escalation to the next dose will only proceed if the safety and tolerability of the previous dose are acceptable to the Investigator and the Sponsor and, if deemed necessary by the IEC following their review of the protocol, after a statement of no objection of the DER from the IEC.

Notification of the end of the study will be sent by PRA to the IEC and to the CA in the Netherlands within 90 days after completion of follow-up for the last subject. In case a study is temporarily halted, PRA will notify the IEC immediately, including the reason for this. In case a study is ended prematurely, PRA will notify the IEC and the CA in the Netherlands within 15 days, including the reasons for the premature termination. A summary of the results of the study will be sent by PRA to the CA and the IEC within 1 year after the end of the study.

4.2 Ethical Conduct of the Study

This study will be conducted in accordance with the ethical principles that have their origin in the World Medical Association (WMA) Declaration of Helsinki, adopted by the 18th WMA General Assembly, Helsinki, Finland, Jun 1964, and subsequent amendments. [15]

Strictly Confidential. © 2021, PRA Health Sciences. All rights reserved.

This study is also designed to comply with ICH E6(R2) Guideline for GCP [12] and the EU CTD Directive 2001/20/EC, [13] as incorporated into Dutch Law. [14]

Guidelines adopted by the ICH and other relevant international guidelines, recommendations, and requirements will be taken into account as comprehensively as possible, as long as they do not violate local law.

Whenever the term "Investigator" is noted in the CSP text, it may refer to either the Investigator at the site or an appropriately qualified, trained, and delegated individual of the investigational site.

4.3 Subject Information and Consent

All subjects will be informed verbally and in writing regarding the objectives, procedures, and risks of study participation. The subjects will be required to sign the Dutch or English version of the ICF before any study-related procedures are started. The ICF contains information about the objectives of the study, the procedures followed during the study, and the risks and restrictions of the study, with special reference to possible side effects of the study drug and potential interactions. In addition, insurance coverage provided during the study is explained. The elements addressed in the ICF are according to the ICH E6(R2) Guideline for GCP. [12]

4.4 Privacy

All personal details will be treated as confidential by the Investigator and staff at PRA, the Sponsor, and any subcontractors involved, and handling of personal data will be in compliance with the EU General Data Protection Regulation. [16]

Strictly Confidential. © 2021, PRA Health Sciences. All rights reserved.

5. STUDY ADMINISTRATIVE STRUCTURE

5.1 Distribution of Activities

5.1.1 Preparation of Study Drug

The study drug will be prepared at the PRA Pharmacy.

5.1.2 Laboratory Assessments

The analysis of XVR011 in serum samples and the analysis of ADAs in serum samples will be performed at LGC Bioanalytical Laboratory.

The analysis of clinical laboratory samples will be performed at the PRA Clinical Laboratory.

5.1.3 Electronic Case Report Form Design

The eCRF design will be performed with the computer program Oracle Clinical (Oracle, Redwood Shores, Redwood City, CA, US) by the Database Programming Department of PRA.

5.1.4 Data Management

Data management will be performed with the computer programs Oracle Clinical (Oracle, Redwood Shores, Redwood City, CA, US), SAS® (SAS Institute Inc, Cary, NC, US), and EXACT (Kinship EXACT™, Kinship Technologies, a technology subsidiary of PRA) by the Data Management Department of PRA.

5.1.5 Statistics

Randomization will be performed by the Biostatistics Department of PRA.

A SAP will be provided by the Biostatistics Department of PRA. The safety analysis and the statistical evaluation of PK parameters will be conducted by the Biostatistics Department of PRA. Upon dose escalation, PK data will be sent directly to and anonymized by an independent PRA biostatistician, in order to maintain the blind. Statistical analysis will be performed with the computer program SAS® (SAS Institute Inc, Cary, NC, US). PK parameters will be calculated using Phoenix WinNonlin (Certara, Princeton, NJ, US). Additional PK computations can also be performed in SAS®. Population-PK analysis will be performed by the Sponsor.

5.1.6 CSR Writing

The CSR, structured in accordance with the guideline “Structure and Content of Clinical Study Reports-ICH E3,” [17] will be written by PRA.

5.2 Documentation

5.2.1 Archiving

All documents concerning the study will be kept on file in the central archives of PRA for at least 25 years after conduct of the study. The Sponsor will receive the completed eCRFs (upon request, as PDF file).

Strictly Confidential. © 2021, PRA Health Sciences. All rights reserved.

5.2.2 Recording of Data in Source Documents and Electronic Case Report Forms

Wherever possible, all data will be entered directly into the eCRFs. Source documents will be used in some cases.

A data management plan will be written by the Data Management Department of PRA, which will be finalized prior to performing any data validation. An appendix to the data management plan (origin of source data list for data entry) will identify any data to be recorded directly in the eCRF (ie, no prior written or electronic record of data) and which data should be considered source data.

Strictly Confidential. © 2021, PRA Health Sciences. All rights reserved.

6. CONFIDENTIALITY AND PUBLICATION POLICY

All information generated in this study is considered highly confidential and must not be disclosed to any person or entity not directly involved with the study unless prior written consent is gained from the Sponsor. However, authorized regulatory officials, the Sponsor, and its authorized representatives are allowed full access to the records.

All study subjects must be informed that their personal study-related data will be used by the Sponsor in accordance with local data protection law. The level of disclosure must also be explained to the subject, who will be required to give consent for their data to be used as described in the ICF. The subjects must be informed that their medical records may be examined by auditors or other authorized personnel appointed by the Sponsor, by appropriate IEC members, and by inspectors from regulatory authorities.

Identification of subjects and eCRFs shall be by unique subject numbers only.

All personal details will be treated as confidential by the Investigator and staff at PRA.

All relevant aspects regarding publication will be part of the contract (or similar document) between the Sponsor and PRA.

Strictly Confidential. © 2021, PRA Health Sciences. All rights reserved.

7. REFERENCES

1. Wang L, Wang Y, Ye D, Liu Q. Review of the 2019 novel coronavirus (SARS-CoV-2) based on current evidence. Int J Antimicrob Agents. 2020;55(6):105948. doi:10.1016/j.ijantimicag.2020 .105948.
2. Lother SA, Abassi M, Agostinis A, et al. Post-exposure prophylaxis or pre-emptive therapy for severe acute respiratory syndrome coronavirus 2 (SARS-CoV 2): study protocol for a pragmatic randomized-controlled trial. Can J Anaesth. 2020;67(9):1201-1211. doi:10.1007/s12630-020-01684-7.
3. Wu C, Liu Y, Yang Y, et al. Analysis of therapeutic targets for SARS-CoV-2 and discovery of potential drugs by computational methods. Acta Pharm Sin B. 2020 May;10(5):766-788. doi: 10.1016/j.apsb.2020.02.008.
4. Cevik M, Tate M, Lloyd O Maraolo AE, Schafers J, Ho A; SARS-CoV-2, SARS-CoV, and MERS-CoV viral load dynamics, duration of viral shedding, and infectiousness: a systematic review and meta-analysis; Lancet (Microbe) 2021;2(1):e13-e22. doi: 10.1016/S2666-5247(20)30172-5.
5. World Health Organization. Coronavirus disease (COVID-19) Q&As. Last updated 10 Nov 2020. Available at: https://www.who.int/emergencies/diseases/novel-coronavirus-2019/question-and-answers-hub/q-a-detail/coronavirus-disease-covid-19.
6. Cevik M, Kuppalli K, Kindrachuk J, Peiris M. Virology, transmission, and pathogenesis of SARS-CoV-2. BMJ. 23 Oct 2020; 371:m3862. doi: 10.1136/bmj.m3862.
7. Wiersinga WJ, Rhodes A, Cheng AC, Peacock SJ, Prescott HC. Pathophysiology, Transmission, Diagnosis, and Treatment of Coronavirus Disease 2019 (COVID-19): A Review. JAMA. 2020;324(8):782-93. doi:10.1001/jama.2020.12839.
8. Jiang S, Hillyer C, Du L. Neutralizing Antibodies against SARS-CoV-2 and Other Human Coronaviruses. Trends Immunol. 2020 May;41(5):355-359. doi: 10.1016/j.it.2020.03.007.
9. Park SY, Lee JS, Son JS, et al. Post-exposure prophylaxis for Middle East respiratory syndrome in healthcare workers. J Hosp Infect. 2019;101(1):42-46. doi:10.1016/j.jhin.2018.09.005.
10. Investigator's Brochure XVR011 Version 1.0 dated 19 Jan 2021.
11. Davda JP, Dodds MG, Gibbs MA, Wisdom W, Gibbs J. A model-based meta-analysis of monoclonal antibody pharmacokinetics to guide optimal first-in-human study design. MAbs. 2014 Jul-Aug;6(4):1094-102. doi: 10.4161/mabs.29095
12. International Council for Harmonisation of Technical Requirements for Registration of Pharmaceuticals for Human Use. ICH Harmonised Tripartite Guideline, E6(R2): Integrated Addendum to ICH E6(R1): Guideline for Good Clinical Practice. Adopted by the European Medicines Agency (EMA), Committee for Human Medicinal Products, Document Reference EMA/CHMP/ICH/135/1995), 14 Jun 2017.
13. Directive 2001/20/EC of the European Parliament and of the Council of 4 Apr 2001 on the approximation of the laws, regulations and administrative provisions of the Member States relating to the implementation of Good Clinical Practice in the conduct of clinical trials on medicinal products for human use.

Strictly Confidential. © 2021, PRA Health Sciences. All rights reserved.

14. Medical Research Involving Human Subjects Act (WMO, Wet Medisch-Wetenschappelijk Onderzoek met Mensen), revised Dec 2015.
15. World Medical Association (WMA) Declaration of Helsinki – Ethical Principles for Medical Research Involving Human Subjects (18th WMA General Assembly 1964), revised at 64th WMA General Assembly, Fortaleza, Brazil, Oct 2013.
16. The General Data Protection Regulation. Regulation (EU) 2016/679 of the European Parliament and the Council of the European Union, 27 Apr 2016, applicable as of 25 May 2018.
17. International Council for Harmonisation of Technical Requirements for Registration of Pharmaceuticals for Human Use. ICH Harmonised Tripartite Guideline, E3: Structure and Content of Clinical Study Reports. Note for Guidance on Structure and Content of Clinical Study Reports, Adopted by the Committee for Human Medicinal Products, European Medicines Agency (EMA), Document Reference CPMP/ICH/137/95, Jul 1996.
18. International Council for Harmonisation of Technical Requirements for Registration of Pharmaceuticals for Human Use. ICH Harmonised Tripartite Guideline, E2A: Clinical Safety Data Management: Definitions and Standards for Expedited Reporting. Note for Guidance on Clinical Safety Data Management, Adopted by the Committee for Human Medicinal Products, European Medicines Agency (EMA), Document Reference CPMP/ICH/377/95, Jun 1995.

Strictly Confidential. © 2021, PRA Health Sciences. All rights reserved.

8. APPENDICES

8.1 Drug Accountability

Upon receipt of the study drug, it will be inspected and counted by the responsible pharmacist. If necessary, all study drug will be repacked per dosing occasion and labeled according to PRA SOPs.

The study drug will be kept in the PRA Pharmacy or in a locked and secured storage facility accessible to the pharmacist and the pharmacy assistant only.

The responsible pharmacist will keep an inventory. This will include a description of the formulation and the quantity of study drug received for the study and a record of what is dispensed, to whom, and when.

On termination of the study, the responsible pharmacist will conduct a final inventory of the study drug supply and will record the results of this inventory in the Drug Accountability Form. Unused study drug will be returned to the Sponsor at the end of the study or will be locally destroyed according to PRA standard procedures.

8.2 Adverse Events and Serious Adverse Events Evaluation and Reporting

8.2.1 AEs

An AE is any unfavorable and unintended sign (including an abnormal laboratory finding), symptom, or disease temporally associated with the use of a medical treatment or procedure that may or may not be considered related to the medical treatment or procedure. AE definitions will be followed as stated in the “Note for Guidance on Clinical Safety Data Management: Definitions and Standards for Expedited Reporting” (ICH topic E2A). [18]

All AEs reported by the subjects or apparent from their physical appearance during the clinical study will be reported on the AE eCRF page.

The severity of AEs will be graded using the most current version of the CTCAE 5-point scale:

- **Mild (Grade 1)**: Asymptomatic or mild symptoms; clinical or diagnostic observations only; intervention not indicated
- **Moderate (Grade 2)**: Minimal, local, or noninvasive intervention indicated; limiting age-appropriate instrumental activities of daily living (ADL)
- **Severe (Grade 3)**: Severe or medically significant but not immediately life-threatening; hospitalization or prolongation of hospitalization indicated; disabling; limiting self-care ADL
- **Life-threatening (Grade 4)**: Life-threatening consequences; urgent intervention indicated
- **Death (Grade 5)**: Death related to AE

If an AE has multiple aspects, the aspect with the highest severity will be graded.

Strictly Confidential. © 2021, PRA Health Sciences. All rights reserved.

It is emphasized that the term severe is a measure of severity; thus, a severe AE is not necessarily serious. For example, itching for several days may be rated as severe, but may not be clinically serious.

In addition, clinically significant changes in physical examination findings and abnormal objective test findings (eg, clinical laboratory, vital signs, ECG) should also be recorded as AEs. Test findings and physical examination findings can result in AEs if they:

- Are associated with accompanying symptoms, and/or
- Require additional diagnostic testing or medical/surgical intervention, and/or
- Lead to a change in study dosing or discontinuation from the study; result in the addition of significant additional concomitant drug treatment or other therapy, and/or
- Lead to any of the outcomes included in the definition of an SAE, and/or
- Are considered to be an AE by the Investigator or Sponsor

Reporting as an AE should not be triggered by:

- Merely repeating an abnormal test, or
- Any abnormal test result that is determined to be an error

The relationship of any AE to the study drug will be assessed and graded as related or not related.

Related means:

- The AE follows a reasonable temporal sequence to study drug administration and cannot be reasonably explained by the subject's clinical state or other factors (eg, disease under study, concurrent diseases, or concomitant medications)
- The AE follows a reasonable temporal sequence to study drug administration and is a known reaction to the drug under study or a related chemical group, or is predicted by known pharmacology

Not Related means:

- The AE does not follow a reasonable sequence from study drug administration or can be reasonably explained by the subject's clinical state or other factors (eg, disease under study, concurrent diseases, and concomitant medications)

8.2.2 SAEs

An SAE is any untoward medical occurrence that, on the basis of medical and scientific judgment:

- Results in death, or
- Is life-threatening (this refers to an event in which the patient was at risk of death at the time of the event; it does not refer to an event which hypothetically might have caused death if it were more severe), or
- Requires inpatient hospitalization for a medical reason or prolongation of existing hospitalization (this refers to hospital admission required for treatment of the AE) (Note: this does not include confinement in, for example, a respite unit, a skilled

Strictly Confidential. © 2021, PRA Health Sciences. All rights reserved.

nursing unit, a rehabilitation facility, the clinical research center, or confinement due to planned or unplanned reason unrelated to study), or
- Results in persistent or significant disability/incapacity, or
- Is a congenital anomaly/birth defect

Medical and scientific judgment should be exercised in deciding whether expedited reporting is appropriate in other situations, such as important medical events that may not be immediately life-threatening or result in death or hospitalization, but may jeopardize the patient or may require intervention to prevent one of the other outcomes listed in the definition above. These should also usually be considered serious. Examples of such events are intensive treatment in an emergency room or at home for allergic bronchospasm, blood dyscrasias, or convulsions that do not result in hospitalization.

SAEs will be collected from admission until the EOS visit. SAEs that are related to the investigational drug and continue beyond the normal collection period (ie, are ongoing at the time a subject exits the study) will be followed until resolution or until stabilized with sequelae. SAEs that begin after the subject's participation in the study is complete, but that the Investigator considers to be related to study drug, may be reported at any time.

The Investigator or clinical site personnel must notify the Sponsor's Medical Monitor and Parexel Safety Services of all SAEs, regardless of relationship to the investigational drug, within 24 hours of clinical site personnel becoming aware of the event. The Investigator will provide the initial notification by sending a completed "SAE Notification Form," which must include the Investigator's assessment of the relationship of the event to the investigational drug and must be signed by the Investigator.

In addition, notification is sent by Parexel to the IEC and the subject's general practitioner.

Follow-up information, or new information regarding an ongoing SAE, must be provided promptly to the Sponsor's Medical Monitor and Parexel Safety Services.

All SAE reports should be sent to the contacts provided on Page 4: SAE Contact Information.

8.2.3 Suspected Unexpected Serious Adverse Reactions

An SAE that is also an unexpected adverse drug reaction is called a suspected unexpected serious adverse reaction (SUSAR). Unexpected adverse reactions are adverse reactions of which the nature or severity is not consistent with the applicable product information (eg, IB for an unapproved investigational medicinal product or the Summary of Product Characteristics for an authorized medicinal product).

The Sponsor or its representative (eg, PRA if agreed to before start of the study) will promptly report (expedited reporting) the following SUSARs to the IEC:
- SUSARs that have arisen in the current clinical study that was assessed by the IEC

Strictly Confidential. © 2021, PRA Health Sciences. All rights reserved.

- SUSARs that have arisen in other clinical studies of the same Sponsor and with the same medicinal product, and that could have consequences for the safety of the subjects involved in the current clinical study that was assessed by the IEC

The Sponsor or its representative will promptly report (expedited reporting) all SUSARs to the CA and the Medicine Evaluation Board (MEB) of the country where this study is conducted and to the CAs in other Member States, as applicable.

SUSARs that have already been reported to the EMA Eudravigilance database do not have to be reported again to the CA and the MEB because they have direct access to the Eudravigilance database.

Expedited reporting will occur no later than 15 calendar days after the Sponsor or its representative has first knowledge of the adverse reactions. For fatal or life-threatening cases, the term will be maximally 7 calendar days for a preliminary report with another 8 days for completion of the report.

8.2.4 Follow-up of AEs

Follow-up of AEs will continue until resolution, stabilization, or death. In case of ongoing AEs at database closure, the data obtained at database closure will be used in the statistical analysis. The follow-up of the AE will be documented in the source documents and will be described in the final CSR only if considered relevant by the Investigator.

8.3 Pregnancy

A female clinical study subject must be instructed to immediately inform the Investigator if she becomes pregnant during the study. Pregnancies occurring up to 90 days after the study drug administration must also be reported to the Investigator. The Investigator will make arrangements for the subject to be counseled by a specialist to discuss the risks of continuing with the pregnancy and the possible effects on the fetus. Monitoring of the subject should continue until the outcome of the pregnancy is known.

The Investigator should report all pregnancies of female clinical study subjects to the Sponsor within 1 working day of becoming aware of them.

If the Investigator becomes aware of a pregnancy occurring in the partner of a male subject participating in the study up to 90 days after study drug administration of the male subject, the pregnancy should be reported to the Sponsor within 1 working day of obtaining written consent from the pregnant partner. The Investigator may make arrangements for the partner to be counseled by a specialist to discuss the risks of continuing with the pregnancy and the possible effects on the fetus. Monitoring of the partner should continue until the outcome of the pregnancy is known.

Strictly Confidential. © 2021, PRA Health Sciences. All rights reserved.

Signature Page for VV-TMF-973856 v1.0

Reason for signing: Approved	Name: Nabil Djari<br>Role: Safety<br>Date of signature: 15-Sep-2021 07:41:46 GMT+0000

Signature Page for VV-TMF-973856 v1.0
