# Supplementary material for: Characterization of the VHH-Fc construct rimteravimab in healthy adults and patients hospitalized for mild-to-moderate COVID-19: Two Phase 1 randomized clinical trials
Source: PLoS Med. 2026 May 29;23(5):e1004609. doi: 10.1371/journal.pmed.1004609 (PMC13221044; doi:10.1371/journal.pmed.1004609)
Supplement: S2 Protocol — (PDF) [file pmed.1004609.s005.pdf]

Title Page

Protocol Title:	A 2-part Clinical Study Including a First-in-Human, Open-label, Single Ascending Dose Part (Phase 1) Followed by a Randomised, Double-blind, Placebo-controlled Part (Phase 2) to Evaluate the Efficacy and Safety of XVR011 in Patients Hospitalised for Mild to Moderate COVID-19
Short Title:	Dose-Finding, Safety, and Efficacy Study of XVR011 Added to Standard of Care in Patients Hospitalised for COVID-19
Compound:	XVR011
Indication:	COVID-19
Study Sponsor:	ExeVir Bio BV<br>Rijvisschestraat 120<br>Ghent, 9052<br>Belgium
Protocol Number:	EXEVIR0101
Study Phase:	Phase 1/Phase 2
EudraCT number:	2020-005299-36
Protocol Version:	6.0
Approval Date:	22 Nov 2021

Sponsor Signatory

Declaration of Sponsor

Protocol Title: A 2-part Clinical Study Including a First-in-Human, Open-label, Single Ascending Dose Part (Phase 1) Followed by a Randomised, Double-blind, Placebo-controlled Part (Phase 2) to Evaluate the Efficacy and Safety of XVR011 in Patients Hospitalised for Mild to Moderate COVID-19

Protocol Number: EXEVIR0101

This study will be conducted in compliance with the clinical study protocol (and amendments), the World Medical Association Declaration of Helsinki, International Council for Harmonisation of Technical Requirements for Pharmaceuticals for Human Use (ICH) Guideline for Good Clinical Practice E6 (R2), applicable Food and Drug Administration (FDA) regulatory requirements, and local regulations governing the conduct of clinical studies.

Sponsor Signatory:

Dominique Tersago (Signature)

Digitally signed by Dominique Tersago (Signature)
Date: 2021.11.23 08:49:55 +01'00'

Dominique Tersago, MD
Chief Medical Officer

Date

Medical Monitor Name and Contact Information will be provided separately.

Table of Contents

Sponsor Signatory 2
Table of Contents 3
1 Protocol Summary 8
1.1 Synopsis 8
1.2 Schemas 17
1.3 Schedule of Activities (SoA) 19
2 Introduction 24
2.1 Background 24
2.1.1 Summary of Non-clinical Studies 27
2.1.2 Summary of Clinical Studies 28
2.2 Study Rationale 28
2.3 Benefit/Risk Assessment 29
2.3.1 Risk Assessment 29
2.3.2 Benefit Assessment 31
2.3.3 Overall Benefit: Risk Conclusion 31
3 Objectives and Endpoints 32
4 Study Design 36
4.1 Overall Design 36
4.2 Scientific Rationale for Study Design 37
4.3 Justification for Dose 39
4.4 End of Study Definition 40
5 Study Population 41
5.1 Inclusion Criteria 41
5.2 Exclusion Criteria 42
5.3 Lifestyle Considerations 44
5.4 Screen Failures 44
5.4.1 Screening and Enrolment Log and Subject Identification Numbers 44
6 Study Treatment 45
6.1 Study Treatments Administered 45

6.2 Preparation, Handling, Storage, and Accountability 47
6.3 Measures to Minimise Bias 49
6.3.1 Part 1 49
6.3.2 Part 2 49
6.4 Study Treatment Compliance 50
6.5 Concomitant Therapy 50
6.5.1 Standard of Care for COVID-19 51
6.6 Dose Modification and Stopping/Suspension Rules 52
6.6.1 Dose Escalation and Stopping/Suspension Rules During Part 1 53
6.6.2 Dose Selection for and Start of Part 2 54
6.7 Discharge from the Hospital and Follow-up 55
6.8 Treatment After the End of the Study 55
7 Discontinuation of Study Treatment and Subject Discontinuation 56
7.1 Discontinuation of Study Treatment 56
7.2 Subject Discontinuation/Withdrawal from the Study 56
7.3 Loss of Subjects to Follow-Up 56
8 Study Assessments and Procedures 58
8.1 Efficacy Assessments 59
8.1.1 Clinical Status According to 8-point Ordinal Scale 59
8.1.2 Respiratory Parameters 60
8.1.3 Viral Load 60
8.1.4 COVID-19 Related Symptoms 60
8.2 Safety Assessments 60
8.2.1 Physical Examinations 61
8.2.2 Vital Signs 61
8.2.3 Electrocardiograms 61
8.2.4 Clinical Safety Laboratory Assessments 61
8.3 Adverse Events and Serious Adverse Events 63
8.3.1 Time Period and Frequency for Collecting AE and SAE Information 63
8.3.2 Method of Detecting AEs and SAEs 63
8.3.3 Follow-up of AEs and SAEs 64
8.3.4 Regulatory Reporting Requirements for SAE 64
8.3.5 Pregnancy 64
8.3.6 Adverse Events of Special Interest 64
8.4 Treatment of Overdose 66

8.5 Pharmacokinetics ... 67
8.6 Pharmacodynamics ... 67
8.7 Genetics ... 68
8.8 Biomarkers ... 68
8.9 Immunogenicity Assessments ... 68
8.10 Medical Resource Utilisation and Health Economics ... 69
9 Statistical Considerations ... 70
9.1 Statistical Hypotheses ... 70
9.2 Sample Size Determination ... 70
9.3 Populations for Analyses ... 71
9.4 Statistical Analyses ... 72
9.4.1 General Considerations ... 72
9.4.2 Primary Efficacy Endpoint ... 72
9.4.3 Secondary Efficacy Endpoints ... 73
9.4.4 Safety Analyses ... 73
9.4.5 Pharmacokinetic Analyses ... 74
9.4.6 Other Analyses ... 75
9.5 Interim Analyses ... 75
9.6 Data Monitoring Committee ... 75
10 Supporting Documentation and Operational Considerations ... 76
10.1 Appendix 1: Regulatory, Ethical, and Study Oversight Considerations ... 76
10.1.1 Regulatory and Ethical Considerations ... 76
10.1.2 Financial Disclosure ... 76
10.1.3 Informed Consent Process ... 77
10.1.4 Data Protection ... 77
10.1.5 Committees Structure (IDMC) ... 78
10.1.6 Dissemination of Clinical Study Data ... 78
10.1.7 Data Quality Assurance ... 79
10.1.8 Source Documents ... 80
10.1.9 Study and Site Start and Closure ... 80
10.1.10 Publication Policy ... 81
10.1.11 Protocol Approval and Amendment ... 81
10.1.12 Liability and Insurance ... 82
10.2 Appendix 2: 8-Point Ordinal Scale ... 83

10.3 Appendix 3: Assessment of 14 Common COVID-19-Related Symptoms: Items and Response Options 84
10.4 Appendix 4: Adverse Events: Definitions and Procedures for Recording, Evaluating, Follow-up, and Reporting 85
10.4.1 Definition of AE 85
10.4.2 Definition of SAE 86
10.4.3 Recording and Follow-up of AE and SAE 88
10.4.1 Reporting of SAE 90
10.5 Appendix 5: Contraceptive Guidance and Collection of Pregnancy Information 93
10.5.1 Definitions 93
10.5.2 Contraception Guidance 94
10.5.3 Pregnancy Testing 95
10.5.4 Collection of Pregnancy Information 95
10.6 Appendix 6: Abbreviations and Trademarks 97
11 References 100
Investigator Agreement Page 105

List of Figures

Figure 1–1: Study Design Overview 17
Figure 1–2: Study Diagram 18

List of Tables

Table 1-1 Schedule of Activities – Part 1 19
Table 1–2 Schedule of Activities – Part 2 21
Table 2–1 Risk Assessment 29
Table 3–1 Objectives and Endpoints for Part 1 32
Table 3–2 Objectives and Endpoints for Part 2 34
Table 6–1 Study Treatments Administered 45
Table 6–2 IV Infusion Details for Part 1 46
Table 6–3 Guidance in Case of an Infusion Reaction Based on Severity Of The Reaction 47
Table 6–4 Restricted concomitant medications Part 1 only 52
Table 6–5 Forbidden concomitant medications 52

Table 8–1 Protocol-Required Safety Laboratory Assessments 62

Table 8–2 Guidance in case of an infusion reaction based on severity of the reaction 66

Table 9–1 Determination of Sample Size with Different Time to Recovery Assumptions for Placebo and XVR011 Treatment.. 70

Table 9–2 Populations for Analysis 71

1 Protocol Summary

1.1 Synopsis

Protocol Title: A 2-part Clinical Study Including a First-in-Human, Open-label, Single Ascending Dose Part (Phase 1) Followed by a Randomised, Double-blind, Placebo-controlled Part (Phase 2) to Evaluate the Efficacy and Safety of XVR011 in Patients Hospitalised for Mild to Moderate COVID-19

Sponsor Protocol No.: EXEVIR0101

Study Phase: Phase 1/Phase 2

Sponsor: ExeVir Bio BV

Rationale:

Coronavirus disease 2019 (COVID-19) is an infectious disease primarily affecting the respiratory system; it is caused by the severe acute respiratory syndrome coronavirus 2 (SARS-CoV-2) virus and can lead to significant morbidity and mortality. It has rapidly evolved into a global pandemic for which treatments and vaccines are urgently being developed.

Infection with SARS-CoV2 occurs through viral binding to the host cells of the respiratory tract, primarily through interaction of the viral spike protein with the host cell angiotensin converting enzyme 2 (ACE2) receptor. The virus then rapidly replicates in the upper respiratory tract with viral load reaching a peak within the first week of symptom onset and later in the lower respiratory tract. Some studies suggest a peak in viral load already in the prodromal stage of the infection. Furthermore, a large number of infected people remain asymptomatic. Symptoms are variable both in type and severity. The most common symptoms are fever, dry cough, and fatigue. Other less common ones include loss of taste or smell, nasal congestion, conjunctivitis, sore throat, headache, muscle or joint pain, skin rash, nausea/vomiting, diarrhoea, chills, or dizziness. Hospitalisation is needed mainly for people who develop respiratory distress. This can rapidly progress and become very severe, requiring invasive, mechanical ventilation and intensive care treatment. The pathogenesis of severe COVID-19 is an aberrant immune response with overproduction of pro-inflammatory cytokines and damage to the lung parenchyma. Host factors such as older age, male gender and underlying medical conditions appear to be predisposing factors to development of severe disease. The approach of neutralising the virus by directly targeting its ability to bind to the host cell via the spike protein is a rational approach in arresting the cascade of infection (viral replication) and pulmonary/systemic inflammation.

XVR011 is a single domain (heavy chain only) antibody fragment (VHH) which is linked to the Fc part of a human immunoglobulin G subclass 1 (IgG1). The VHH component was derived from a heavy chain only antibody from a llama, which had been inoculated with SARS-CoV-1 spike protein. When SARS-CoV-2 was identified, the VHH was found to be cross reactive to its spike protein. The VHH binds to an epitope on the receptor-binding domain of the SARS-CoV-2 spike protein and through steric hindrance, blocks the interaction of the spike protein with the ACE2 receptor and thereby prevents the virus from entering the cell and replicating. The binding site is partially hidden and is expected to be less susceptible to genetic pressure and viral mutation. The amino acid sequence of the VHH was further humanized and optimized to increase its antiviral potency. In XVR011, two VHH's are linked to a human IgG1 crystallizable fragment (Fc) fragment with the aim of achieving a half-life similar to that of naturally occurring antibody, i.e. approx. 21 days. The Fc has been modified to partially silence the effector function. This has been done to minimise the potential for antibody-dependent disease enhancement. The XVR011-virus complex is expected to be cleared by non-specific mechanisms such as non-specific pinocytosis.

Rapid neutralisation of SARS-CoV-2 before the immune system has reached the point where an excessive inflammatory reaction has been triggered may potentially reduce the morbidity and mortality associated with COVID-19. The potential of XVR011 to achieve this is being evaluated in this Phase 1/2 clinical trial in patients with symptomatic COVID-19 who are hospitalised but in whom the inflammatory component does not yet require intensive care treatment or mechanical ventilation. The objective is to neutralise the virus more rapidly and achieve a more rapid recovery, reduction in the need for medical care as well as prevent the need for mechanical ventilation and/or intensive care.

Objectives and Endpoints:

PART 1 Objectives	PART 1 Endpoints
**Primary**<br>To evaluate the safety of a single intravenous XVR011 infusion in subjects hospitalised for COVID-19	• Proportion of subjects with Grade ≥ 3 treatment-related AEs within 28-day FU period<br>• Proportion of subjects with AEs (all and serious) of any grade and independent of causality within 28-day FU period<br>• Proportion of subjects with infusion-related reactions within 24 hours of treatment<br>• Proportion of subjects with hypersensitivity reactions within 28-day FU period
**Secondary**<br>To evaluate the efficacy of a single intravenous XVR011 infusion in subjects hospitalised for COVID-19	• Time to recovery (i.e., clinical status reaching level 1 to 3 of the 8-point ordinal scale) within 28-day FU period<br>• Total duration of oxygen supplementation within 28-day FU period<br>• Proportion of subjects requiring mechanical ventilation within 28-day FU period<br>• Proportion of subjects requiring ICU transfer within 28-day FU period<br>• Time to hospital discharge<br>• Proportion of subjects with clinical status evolving to each level of the 8-point ordinal scale at Days 2, 3, 4, 8, 15, 22 and Day 29, compared to baseline<br>• Proportion of subjects with COVID-19 related symptoms<br>• All-cause mortality rate within 28-day FU period
To evaluate the antiviral activity of a single intravenous XVR011 infusion in subjects hospitalised for COVID-19	• Change from baseline (Day 1 pre-dose) in the viral load (RT-qPCR) of nasopharyngeal samples at Day 1, Day 3, Day 8 and day of discharge

PART 1 Objectives	PART 1 Endpoints
**Exploratory**<br>To evaluate the effect of a single intravenous XVR011 infusion on disease biomarkers in subjects hospitalised for COVID-19	• Blood biomarkers (including but not limited to CRP, LDH, D-dimers, fibrinogen, absolute neutrophil count, lymphocyte count) at baseline (Day 1 pre-dose), on Days 2, 3, 4, 8, 15, 22 and on day of discharge<br>• Optional blood sample for future exploratory biomarker testing at baseline (Day 1 pre-dose) and Day 3
To evaluate the pharmacokinetics (PK) of XVR011, following a single intravenous infusion in subjects hospitalised for COVID-19	• Blood concentrations of XVR011 and PK parameters (Cmax, AUC, $t_{1/2}$) on Days 1, 2, 4, 8, 15 and Day 29.
To evaluate the immunogenicity of XVR011, following a single intravenous infusion in subjects hospitalised for COVID-19	• ADAs and nAbs at baseline (Day 1 pre-dose), Day 15, and Day 29

ADA = anti-drug antibody; AE = adverse event; AUC = area under the plasma concentration-time curve; Cmax = maximum observed plasma concentration; COVID-2019= coronavirus disease 2019; CRP = C-reactive protein; FU = follow-up; ICU = intensive care unit; LDH = lactate dehydrogenase; nAb = neutralising anti-drug antibody; RT-qPCR = reverse transcriptase quantitative polymerase chain reaction; $t_{1/2}$ = apparent plasma terminal elimination half-life.

PART 2 Objectives	PART 2 Endpoints
**Primary**<br>To evaluate the efficacy of a single intravenous XVR011 infusion in subjects hospitalised for COVID-19, compared to placebo	• Time to sustained recovery (i.e., clinical status reaching level 1 to 3 of the 8-point ordinal scale) within 28-day FU period; where recovery must be confirmed to be maintained at level 3 or lower for 14 consecutive days
**Secondary**<br>To further evaluate the efficacy of a single intravenous XVR011 infusion in subjects hospitalised for COVID-19, compared to placebo	• Proportion of subjects who are alive and do not require any form of mechanical ventilation [a] at Day 8<br>• Proportion of subjects requiring any form of mechanical ventilation [a] within 28-day FU period<br>• Proportion of subjects requiring ICU transfer within 28-day FU period<br>• Time to sustained hospital discharge (i.e. discharge with no re-hospitalisation after initial discharge for treatment of COVID-19 related events in the following 14 days) within the 90 day FU period<br>• Total duration of oxygen supplementation within 28-day FU period<br>• Proportion of subjects with clinical status evolving to each level of the 8-point ordinal scale at Days 2, 3, 4, 8, 15, 22 and Day 29, compared to baseline<br>• Proportion of subjects with COVID-19 related symptoms<br>• All-cause mortality rate within 28-day FU period
To evaluate the antiviral activity of a single intravenous XVR011 infusion in subjects hospitalised for COVID-19, compared to placebo	• Change from baseline (Day 1 pre-dose) in the viral load (RT-qPCR) of nasopharyngeal samples at Day 1, Day 3, Day 8 and day of discharge

PART 2 Objectives	PART 2 Endpoints
To evaluate the safety of a single intravenous XVR011 infusion in subjects hospitalised for COVID-19, compared to placebo	• Proportion of subjects with Grade ≥ 3 treatment-related AEs within 28-day FU period<br>• Proportion of subjects with AEs (all and serious) of any grade and independent of causality within 28-day FU period<br>• Proportion of subjects with infusion-related reactions within 24 hours of treatment<br>• Proportion of subjects with hypersensitivity reactions within 28-day FU period
**Exploratory**<br>To evaluate the effect of a single intravenous XVR011 infusion on disease biomarkers in subjects hospitalised for COVID-19	• Blood biomarkers (including but not limited to CRP, LDH, D-dimers, fibrinogen, absolute neutrophil count, lymphocyte count) at baseline (Day 1 pre-dose), on Days 2, 3, 4, 8, 15, 29 and on day of discharge<br>• Optional blood sample for future exploratory biomarker testing at baseline (Day 1 pre-dose) and Day 3
Additional evaluation of COVID-19 symptoms during the extended follow up period.	• COVID-19 related symptoms on Day 60 and Day 90
Characterise viral genotype	• At baseline and Day 8
To determine the PK parameters of XVR011, following a single intravenous infusion in subjects hospitalised for COVID-19	• Blood concentrations of XVR011 and PK parameters (Cmax, AUC, $t1_{/2}$) on Days 1, 4, 8, 15, 29, 60 and 90.
To evaluate the immunogenicity of XVR011, following a single intravenous infusion in subjects hospitalised for COVID-19, compared to placebo	• ADAs and nAbs at baseline (Day 1 pre-dose), Day 15 and Day 29.

[a] Any form of mechanical ventilation includes but not limited to high flow oxygen therapy, continuous positive airway pressure, invasive mechanical ventilation

ADA = anti-drug antibody; AE = adverse event; AUC = area under the plasma concentration-time curve; Cmax = maximum observed plasma concentration; COVID-2019= coronavirus disease 2019; CRP = C-reactive protein; FU = follow-up; ICU = intensive care unit; LDH = lactate dehydrogenase; nAb = neutralising anti-drug antibody; RT-qPCR = reverse transcriptase quantitative polymerase chain reaction; $t1_{/2}$ = apparent plasma terminal elimination half-life.

Overall Design:

This is a 2-part Phase 1/2 clinical study to evaluate the efficacy and safety of XVR011 in subjects hospitalised for COVID-19.

Part 1 (Phase I): Part 1 consists of an open-label, single ascending dose, Phase 1 study to evaluate the safety of XVR011 administered as a single intravenous infusion, at 3 dose levels, and evaluate the PK, pharmacodynamic (PD), and initial efficacy of the treatment. Eligible subjects will be enrolled in 2 successive cohorts of 9 subjects, each. Two subjects in each cohort will be sentinel subjects monitored for at least 24 hours (first sentinel subject will be monitored for 24 hours before the second sentinel subject is treated and monitored for 24 hours) before the remaining subjects can be dosed and decision to open Cohort 2 will depend on the review of data collected from subjects enrolled in Cohort 1 after the last subject was treated and monitored for 24 hours. The same strategy will be followed for Cohort 3 (see Section 6.6.1 for details).

A phase 1a study (protocol EXEVIR0102) in healthy subjects is being conducted in parallel with Part 1 of this study. It is evaluating the same dose levels, namely 250, 500 and 1000 mg as a single IV infusion in a dose ascending design, with a placebo control. Per dose level, 8 subjects receive a single IV infusion of XVR011, and 2 subjects receive a single IV infusion if placebo in a blinded manner. The study will generate PK data and safety data not confounded by the disease state. An interim analysis of study EXEVIR0102 will be performed when all dose groups have received treatment and when the last dose group (1000 mg) has completed the 8-day follow-up. As discussed in section 4.3 Justification for Dose, the main driver for the dose determination is the target serum concentration and number of days this is exceeded, in addition to safety. The PK parameters from the healthy volunteers are considered predictive for the patient setting. The safety profile will be reviewed of the 3 different dose levels in the healthy subject study.

The IDMC will be asked to make a recommendation to progress to Part 2 (phase 2 design) of this study with the recommended dose based on the interim analysis of the Phase 1a study in 30 healthy subjects, supplemented with the available data from Part 1 of this study, even if not all Part 1 cohorts have reached the planned enrolment. This is due to recruitment challenges in Part 1 of this study resulting from low hospitalisation rate and reluctance of sites to participate and enrol patients in a phase I trial.

Part 2 (Phase 2): Part 2 consists of a double-blind, randomised, placebo-controlled Phase 2 study to evaluate the efficacy, safety, PK, and PD of XVR011 administered at the recommended dose level based on the IDMC review and recommendation. A total of 408 eligible subjects will be randomised in a 2:1 ratio to receive either XVR011 (272 subjects) or placebo (136 subjects).

Standard of care (SoC), according to local requirements will be allowed, except if listed in Table 6.5 Forbidden Concomitant Medications. Stratification will be done at randomisation for country and for fully vaccinated/prior SARS-CoV-2 infection. Subgroup analyses will include factors such as disease state, baseline serology, baseline viral load levels and duration of COVID-19 symptoms prior to study treatment.

For both Part 1 and Part 2 the study will consist of 3 periods: Screening (Day -1), Treatment period (Day 1), and Endpoint Assessment period (7 to 8 visits, Day 2 to Day 29); for Part 2 there will be an additional Follow-up period (2 visits, Day 60 and Day 90):

- **Screening (Day -1)**: Subjects who are hospitalised and provide informed consent will undergo screening assessments as soon as possible after presentation with COVID-19 to evaluate their eligibility based on the inclusion and exclusion criteria. If eligible, the subject may be randomised (e.g for Part 1 this means IWRS treatment registration).

- **Treatment period (Day 1)**: Within 12 hours of randomisation, a single intravenous infusion of study treatment will be started under medical supervision for the whole duration of the infusion and for two hours after the end of the infusion. This treatment will be administered in addition to SoC treatment.

- **Endpoint assessment period (Day 2 to Day 29)**: Subjects will remain hospitalised until at least 24 hours after study treatment administration. Hospital discharge after this period is at the discretion of the Investigator and will be based on the clinical condition of the subject. Study assessments will be performed every day up to Day 4 and then weekly until Day 29. Subjects who are discharged from the hospital before the Day 29 visit will return to the study site for each study visit until Day 29 (see Section 6.7 for details). Day 29 is considered as the EOS for part 1.

- **Extended follow-up period (Day 30 to Day 90) for part 2 only:** Subjects will return to the study site for 2 additional visits, on day 60 and day 90. Day 90 is considered as the EOS for part 2.

Disclosure Statement:

- **Part 1**: This is a sequential treatment study with 3 dose cohorts that are open-label.

- **Part 2**: This is a parallel treatment study with 2 treatment arms, that are subject- and Investigator-blinded.

Number of Subjects:

- **Part 1:** Up to 27 eligible subjects will be enrolled in 3 successive cohorts of 9 subjects each.

- **Part 2:** Approximately 408 eligible subjects will be randomised to 1 of the 2 study treatments (272 subjects in the XVR011 arm, 136 subjects in the placebo arm).

Note: "Enrolled" means a subject's, or their legally acceptable representative's, agreement to participate in a clinical study following completion of the informed consent process. Potential subjects who are screened for the purpose of determining eligibility for the study, but do not participate in the study, are considered "screen failures".

Treatment Groups and Duration:

The total duration of subject participation is 30 days for Part 1 and 91 days for Part 2, including screening (1 day), treatment period (1 day), endpoint assessment period (28 days), extended follow up Part 2 only (61 days). All subjects will receive a single dose of study treatment on Day 1 of the study.

- **Part 1:**
  - Cohort 1 (dose level 1): Single dose of XVR011 250 mg in 25 mL, administered as a 25-minute intravenous infusion at a rate of 10 mg/min.

  - Cohort 2: (dose level 2) Single dose of XVR011 500 mg in 50 mL, administered as a 50-minute intravenous infusion at a rate of 10 mg/min.

  - Cohort 3: (dose level 3) Single dose of XVR011 1000 mg in 100 mL, administered as a 100-minute intravenous infusion at a rate of 10 mg/min.

- **Part 2:**
  - XVR011 arm: Single dose of XVR011 (dose level 1, 2, or 3 of Part 1) administered as an intravenous infusion with the infusion time and rate corresponding to the selected dose level of Part 1.

  - Placebo arm: Single dose of placebo administered as an intravenous infusion with the infusion time and rate corresponding to the selected dose level of Part 1.

Data Monitoring Committee: Yes

1.2 Schemas

Figure 1–1: Study Design Overview

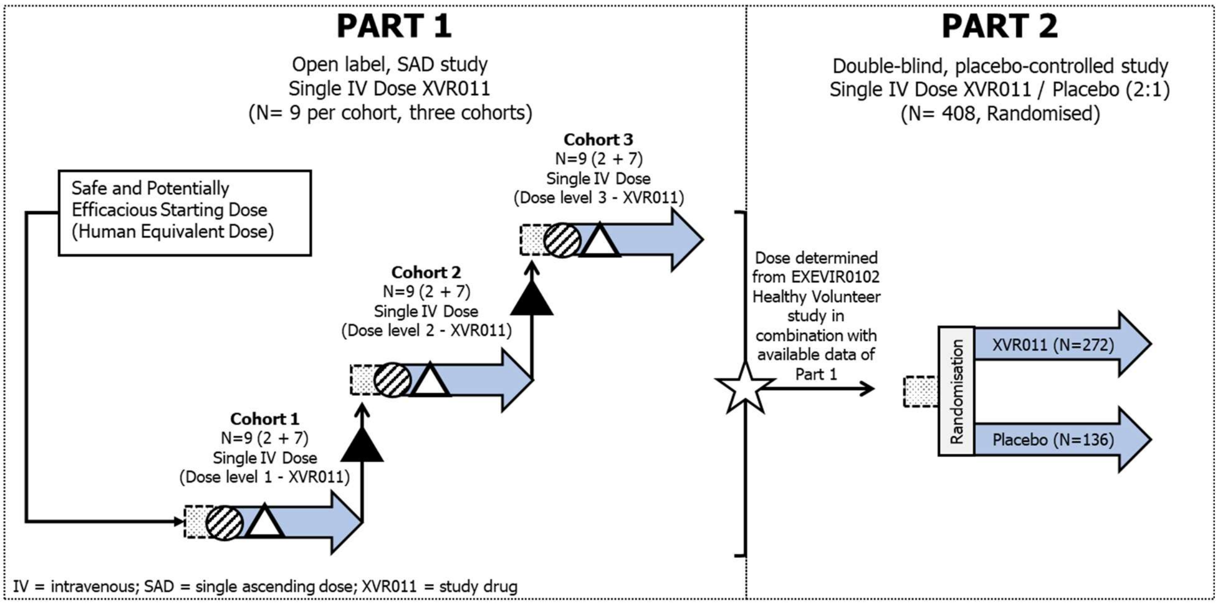

Screening (Day -1) — Treatment Period (Day 1), Follow-up Period (Day 2 to Day 29), Extended Follow-up period (Day 30 to Day 90, part 2 only) Discharge (Day ≥2), EoS (Day 29 part 1, Day 90 part 2)

Sentinel monitoring: close monitoring of two sequential sentinel subjects for 24 hours after treatment. If neither have any SAE considered at least possibly related to the treatment (serious adverse reaction) and if only one sentinel subject has a Grade ≥ 3 treatment-related AE during this period, then after Sponsor review, the remaining 7 subjects will be enrolled and treated.

△ If one sentinel subject has a serious adverse reaction, or if both sentinel subjects have a Grade ≥3 treatment-related AE, enrolment will be suspended and IDMC will review the data and make a recommendation regarding study continuation.

If at any point during the Cohort, a serious adverse reaction is reported in 1 or more subjects, or Grade ≥ 3 treatment related AEs are reported in 2 or more subjects, enrolment will be suspended pending the outcome of an IDMC review

↑ Dose escalation: enrolment in the next cohort will start after Sponsor review, if no subjects in the previous cohort have any serious adverse reactions and if no more than one subject has any Grade ≥3 treatment-related AEs.

▲ If 1 or more of the 9 subjects have a serious adverse reaction, or if 2 or more of the 9 subjects have a Grade ≥3 treatment-related AE, the IDMC will review the data and will make a recommendation on starting the next Cohort.

☆ The Independent Data Monitoring Committee (IDMC) will assess the interim safety and PK data from study EXEVIR0102 (healthy subjects) with the available safety, tolerability, efficacy, PK and PD data from part 1 and will make a recommendation on continuing to Part 2 and will recommend the XVR011 dose for Part 2.

Figure 1–2: Study Diagram

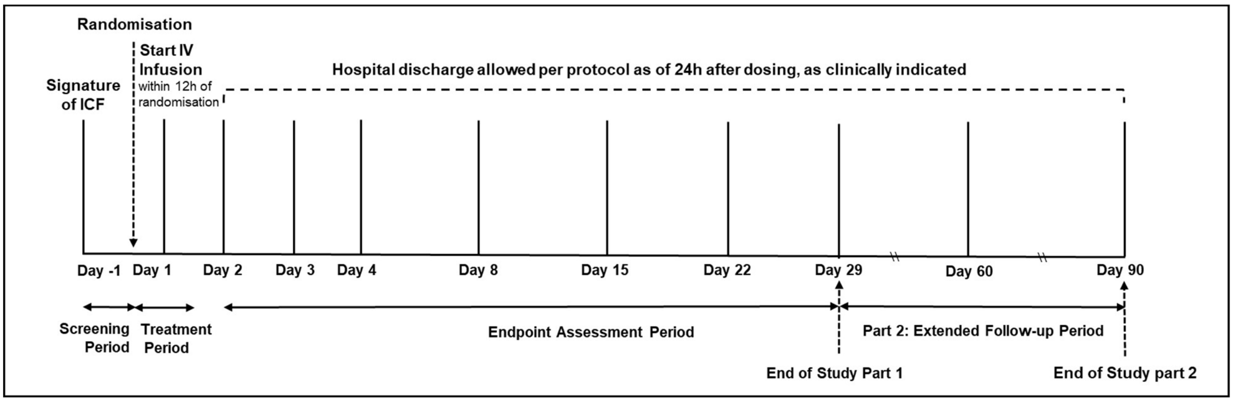

1.3 Schedule of Activities (SoA)

Table 1-1 Schedule of Activities – Part 1

	Screening [a]	Treatment period	Endpoint Assessment Period							
**Visit**	**1**	**2**	**3**	**4**	**5**	**6**	**7**	**8**	**Discharge**	**EOS / ED**
**Study Day (window of allowance)**	**-1**	**1**	**2 (±2 h)**	**3 (±4 h)**	**4 (±4 h)**	**8 (±24 h)**	**15 (±24 h)**	**22 (±24 h)**	**≥2 to <30**	**29 (±24 h)**
**Administrative procedures**										
Informed consent	X									
Eligibility criteria	X									
Demography	X									
Medical history	X									
IWRS treatment registration		X								
**Safety**										
Vital signs	X	X	X	X	X	X	X	X	X	X
Physical examination	X	X (pre-dose)[d]	X[d]	X[d]	X[d]				X[d]	X[d]
12-Lead ECG	X								X	X
Adverse events[e]	X	X	X	X	X	X	X	X	X	X
Concomitant medication	X	X	X	X	X	X	X	X	X	X
**Laboratory tests**										
Safety laboratory parameters (haematology, clinical chemistry, coagulation parameters, estimated creatinine clearance)	X	X (pre-dose)[j]	X	X	X	X	X	X	X	X
Biomarkers		X (pre-dose)	X	X	X	X	X	X	X	
Optional blood sample for future exploratory biomarker testing (central lab)		X (pre-dose)		X						
Pregnancy test (WOCBP only)[f]	X									X

	Screening [a]	Treatment period	Endpoint Assessment Period							
**Visit**	**1**	**2**	**3**	**4**	**5**	**6**	**7**	**8**	**Discharge**	**EOS / ED**
**Study Day (window of allowance)**	**-1**	**1**	**2 (±2 h)**	**3 (±4 h)**	**4 (±4 h)**	**8 (±24 h)**	**15 (±24 h)**	**22 (±24 h)**	**≥2 to <30**	**29 (±24 h)**
FSH (post-menopausal women only)[g]	X									
Anti-SARS-CoV-2 antibody test		X (pre-dose)								
Viral load nasopharyngeal sample		X (pre-dose)		X		X			X	
**Efficacy**										
Clinical status (8-point ordinal scale)		X (pre-dose)	Daily until hospital discharge and at each visit after hospital discharge							
Respiratory parameters[b]		X (pre-dose)	X	X	X	X	X	X	X	X
COVID-19-related symptoms[h]		X (pre-dose)	X	X	X	X	X	X	X	X
**Pharmacokinetics**										
PK blood sample[i]		X pre- and post-dose[i]	X[i]		X[i]	X[i]	X[i]			X[i]
ADA and nAb blood samples		X (within 60 min pre-dose)					X			X
**Study treatment Infusion**										
Study treatment infusion		X[c]								

ADA = anti-drug antibody; AE = adverse event; COVID-19 = coronavirus disease 2019; ECG = electrocardiogram; ED = early discontinuation; EOS = end of study, FSH = follicle stimulating hormone; IWRS = interactive web response system; nAb = neutralising anti-drug antibody; PK = pharmacokinetics; SAE = serious adverse event; SARS-CoV-2 = severe acute respiratory syndrome coronavirus 2, WOCBP = woman of childbearing potential.

a. Screening assessments will be done as soon as possible after presentation with COVID-19.
b. Oxygen supplementation details (duration and type), respiratory rate, oxygen saturation on room air and with oxygen supplementation.
c. Study treatment administration within 12 hours of IWRS treatment registration and under medical supervision for the whole duration of the infusion and for two hours after the end of the infusion. Subjects will be closely monitored for 24 hours after receiving XVR011.
d. Symptom- or history-directed examination.
e. The Investigator will make an assessment of intensity for each AE and SAE reported during the study, according to National Cancer Institute Common Terminology Criteria for Adverse Events (NCI-CTCAE) version 5.0.
f. Women of childbearing potential (Appendix 5) will have a urine pregnancy test. If found positive, will have a serum pregnancy test.

g. If applicable in the judgement of the Investigator, menopausal status should be confirmed by a FSH level in the post-menopausal range, particularly in post-menopausal women who are not using hormonal contraception or hormonal replacement therapy.

h. The Investigator or designee will perform signs and symptom assessment using the Food and Drug Administration (FDA) sign and symptoms scoring system (assessment of 14 common COVID-19-related symptoms).

i. PK samples will be collected within 60 minutes pre-dose, 30 ± 15 minutes post-dose (from the end of infusion) on Day 1, 24 ± 2 hours post-dose on Day 2, 72 ± 2 hours post-dose on Day 4, on Day 8, on Day 15, and on Day 29.

j. Day 1 (baseline) safety laboratory parameters do not need to be repeated if the corresponding screening laboratory parameters have been done within 24 hours of the start of treatment.

Table 1–2 Schedule of Activities – Part 2

	Screening[a]	Treatment period	Endpoint Assessment period							Extended FU		Discharge	ED
**Visit**	**1**	**2**	**3**	**4**	**5**	**6**	**7**	**8**	**9**	**10**	**EOS**		
**Study Day (window of allowance)**	**-1**	**1**	**2 (±2 h)**	**3 (±4 h)**	**4 (±4 h)**	**8 (± 1 d)**	**15 (± 1 d)**	**22 (± 1 d)**	**29 (± 1 d)**	**60 (±3 d)**	**90 (±3 d)**	**≥2 to <90**	
**Administrative procedures**													
Informed consent	X												
Eligibility criteria	X												
Demography	X												
Medical history (including previous SARS-CoV-2 infection(s))	X												
Current COVID-19 History	X												
Randomisation		X											

	Screening[a]	Treatment period	Endpoint Assessment period							Extended FU		Discharge	ED
**Visit**	**1**	**2**	**3**	**4**	**5**	**6**	**7**	**8**	**9**	**10**	**EOS**		
**Study Day (window of allowance)**	**-1**	**1**	**2 (±2 h)**	**3 (±4 h)**	**4 (±4 h)**	**8 (± 1 d)**	**15 (± 1 d)**	**22 (± 1 d)**	**29 (± 1 d)**	**60 (±3 d)**	**90 (±3 d)**	**≥2 to <90**	
**Safety**													
Vital signs	X	X	X	X	X	X	X	X	X			X	X[k]
Physical examination	X	X (pre-dose)[d]	X[d]	X[d]	X[d]			X[d]	X[d]			X[d]	X[dk]
12-Lead ECG	X											X	X[k]
Adverse events[e]	X	X	X	X	X	X	X	X	X	X	X	X	X
Concomitant medication (including prior COVID-19 vaccination)	X	X	X	X	X	X	X	X	X	X	X	X	X
**Laboratory tests**													
Safety laboratory parameters (haematology, clinical chemistry, coagulation parameters, estimated creatinine clearance)	X	X (pre-dose)[j]	X		X	X	X	X	X			X	X[k]
Biomarkers		X (pre-dose)	X	X	X	X	X	X	X			X	
Optional blood sample for future exploratory biomarker testing (central lab)		X (pre-dose)		X									
Pregnancy test (WOCBP only)[f]	X								X	X	X		X
FSH (post-menopausal women only)[g]	X												
Anti-SARS-CoV-2 antibody test		X (pre-dose)											
Viral load nasopharyngeal sample		X (pre-dose)		X		X						X	
**Efficacy**													
Clinical status (8-point ordinal scale)		X (pre-dose)	Daily until hospital discharge and at each visit after hospital discharge										
Respiratory parameters[b]		X (pre-dose)	X	X	X	X	X	X	X	X	X	X	X
COVID-19-Related Symptoms[h]		X (pre-dose)	X	X	X	X	X	X	X	X	X	X	X

	**Screening[a]**	**Treatment period**	**Endpoint Assessment period**							**Extended FU**		**Discharge**	**ED**
**Visit**	**1**	**2**	**3**	**4**	**5**	**6**	**7**	**8**	**9**	**10**	**EOS**		
**Study Day (window of allowance)**	**-1**	**1**	**2** (±2 h)	**3** (±4 h)	**4** (±4 h)	**8** (± 1 d)	**15** (± 1 d)	**22** (± 1 d)	**29** (± 1 d)	**60** (±3 d)	**90** (±3 d)	**≥2 to <90**	
**Pharmacokinetics**													
PK blood sample[i]		X pre- and post-dose[i]			X[i]	X[i]	X[i]		X[i]	X[i]	X[i]		X[i]
ADA and nAb blood samples		X (within 60 min pre-dose)					X		X				X[k]
**Study treatment Infusion**													
Study treatment infusion		X[c]											

ADA = anti-drug antibody; COVID-19 = coronavirus disease 2019; ECG = electrocardiogram; ED = early discontinuation; EOS = end of study, nAb = neutralising anti-drug antibody; PK = pharmacokinetics; SARS-CoV-2 = severe acute respiratory syndrome coronavirus 2, WOCBP = woman of childbearing potential.

a. Screening assessments will be done as soon as possible after presentation with COVID-19.
b. Oxygen supplementation details (duration and type), respiratory rate, oxygen saturation on room air and with oxygen supplementation.
c. Study treatment administration within 12 hours of randomisation and under medical supervision for the whole duration of the infusion and for two hours after the end of the infusion.
d. Symptom- or history-directed examination.
e. The Investigator will make an assessment of intensity for each AE and SAE reported during the study, according to National Cancer Institute Common Terminology Criteria for Adverse Events (NCI-CTCAE) version 5.0.
f. Women of childbearing potential (Appendix 5) will have a urine pregnancy test. If found positive, will have a serum pregnancy test.
g. If applicable in the judgement of the Investigator, menopausal status should be confirmed by a FSH level in the post-menopausal range, particularly in post-menopausal women who are not using hormonal contraception or hormonal replacement therapy.
h. The Investigator or designee will perform signs and symptom assessment using the Food and Drug Administration (FDA) sign and symptoms scoring system (assessment of 14 common COVID-19-Related Symptoms).
i. The PK samples will be collected within 60 minutes pre-dose, 30 ± 15 minutes post-dose (from the end of infusion) on Day 1, 72 ± 2 hours post-dose on Day 4, on Day 8, on Day 15, Day 29, Day 60 and on Day 90. Samples are also taken at ED.
j. Day 1 (baseline) safety laboratory parameters do not need to be repeated if the corresponding screening laboratory parameters have been done within 24 hours of the start of treatment.
k. These assessments only need to be performed if the ED takes place during the Endpoint Assessment Period.

2 Introduction

XVR011 is being developed by ExeVir Bio BV (hitherto referred to as "Sponsor") as a single intravenous infusion for the treatment of coronavirus disease 2019 (COVID-19).

XVR011 is a single domain antibody linked to a partially silenced crystallizable fragment (Fc) part of a human immunoglobulin G subclass 1 (IgG1). It binds to the spike protein of the severe acute respiratory syndrome coronavirus 2 (SARS-CoV-2) and, through steric hindrance, prevents the virus from binding to the angiotensin-converting enzyme 2 (ACE2) receptor, thereby preventing it from entering the cell. It is intended as an antiviral treatment to halt infection in the patient and thereby reduce the chance of progression to more severe disease. The partially silenced Fc function means that it should not bind to the Fc receptor on immune cells and is expected to avoid immune-mediated exacerbation (antibody-dependent enhancement) of the disease.

The proposed population is patients, in whom the viral load is causing mild to moderate disease and who require medical care or supplemental oxygen in the hospital setting but who are not in need of mechanical ventilation or intensive care. The aim of treatment with XVR011 is to neutralise the virus, achieve faster clinical recovery, and thereby prevent progression to severe complications such as acute respiratory distress syndrome (ARDS) or other major organ dysfunctions.

2.1 Background

SARS-CoV-2 has demonstrated high transmissibility and infectivity, and its widespread infection has led to the declaration of a global pandemic by the World Health Organization (WHO) (Wang et al 2020). Although the majority (80%) of people with COVID-19 exhibit mild symptoms and recover spontaneously, approximately 14% of individuals will require hospitalisation, and 30% of hospitalised patients will develop respiratory failure, shock, and/or multiple organ system dysfunction, thus requiring admission to a critical care unit (Lother et al 2020).

The RNA genome of SARS-CoV-2 encodes for 4 major structural proteins (spike, envelope, membrane, and nucleocapsid), approximately 16 non-structural proteins, and 5 to 8 accessory proteins. Among them, the spike protein plays a crucial role in viral attachment, fusion, entry, and transmission (Wu et al 2020). The viral spike protein binds to host cell-bound ACE2, which is the primary mechanism by which the SARS-coronavirus infects human cells. The virus then rapidly replicates in the upper respiratory tract, with viral load reaching a peak within the first week of symptom onset and later in the lower respiratory tract (Cevik et al 2020b).

Some studies suggest a peak in viral load already in the prodromal stage of the infection. Furthermore, a large number of infected people remain asymptomatic. Symptoms are variable both in type and severity. The most common symptoms are fever, dry cough, and fatigue. Other less common ones include loss of taste or smell, nasal congestion, conjunctivitis, sore throat, headache, muscle or joint pain, skin rash, nausea/vomiting, diarrhoea, chills or dizziness (WHO/Coronavirus disease Q&A/2020.11). Hospitalisation is needed mainly for people who develop respiratory distress. This can rapidly progress and become very severe, requiring invasive, mechanical ventilation and intensive care treatment. The pathogenesis of severe COVID-19 is an aberrant immune response with overproduction of pro-inflammatory cytokines and damage to the lung parenchyma (Cevik et al 2020a). Host factors such as older age, male gender and underlying medical conditions appear to be predisposing factors to development of severe disease.

Knowledge and understanding of SARS-CoV-2 infection and progression to symptomatic disease has evolved since it was first described and the need for an antiviral treatment in a hospitalised patient setting remains and has become more clearly relevant. Viral load can persist beyond the initially assumed timeframe and is present in lungs of severely ill patients (Blot 2021). A metanalysis of viral load dynamics suggests that viral load peaks in the upper respiratory tract within the 1st week of symptoms, but in the 2nd week of symptoms in the lower respiratory tract (Cevik 2021). In the context of viral shedding studies, live virus has not been demonstrated beyond day 9 of symptoms. These are however a limited number of studies with sample sizes of 9 to 155 patients. Furthermore, it is apparent that more severe disease occurs due to an inadequate immune response in which binding but not neutralising antibodies may play a role with an inability to control the infection (Osuchowski 2021). Several classes of drugs had initially been evaluated or developed for the management of COVID-19, including antivirals (e.g., remdesivir, favipiravir), antibodies (e.g., convalescent plasma, hyperimmune immunoglobulins), anti-inflammatory agents (dexamethasone, statins), targeted immunomodulatory therapies (e.g., tocilizumab, sarilumab, anakinra, ruxolitinib), anticoagulants (e.g., heparin), and antifibrotics (e.g., tyrosine kinase inhibitors) (Wiersinga et al 2020).

Viral neutralising antibodies induced by vaccines or the virus are critical in the control of viral infection. Monoclonal antibodies have been shown to successfully block viral binding to host cells in other members of the coronavirus family (SARS and MERS) (Jiang et al 2020; Park et al 2018). A number of neutralising monoclonal antibodies have been given Emergency Use Authorisation by the FDA and EMA for use in an outpatient setting. However no antiviral treatment, apart from the early approval for remdesivir by both the FDA and EMA, has been approved for use in patients hospitalised for COVID-19. The evidence for a clinical benefit of remdesivir is considered insufficient by the WHO to recommend its use. The Infectious Disease Society of America (IDSA) currently has a conditional recommendation (low certainty of evidence) for use of remdesivir in

patients on supplemental oxygen but not on mechanical ventilation or extra-corporeal membrane oxygenation. Clinical data have recently emerged from the UK-RECOVERY study which demonstrate a benefit of treatment with a neutralising mAb (casirivimab/imdevimab cocktail) in hospitalised patients who were seronegative at baseline. The 28-day mortality rate was reduced to 24% for the mAb treatment arm compared to 30% in the standard of care only arm (rate ratio of 0.80, 95% CI 0.70-0.91), (Horby 2021). Key secondary endpoints also showed a treatment benefit: shorter median duration of hospitalisation if 13 vs. 17 days and proportion of patients discharged from hospital within 28 days of 64 vs. 58% (rate ratio of 083, 95% CI 0.75-0.92), mAb vs SOC only treatment respectively. Use of ventilation was also lower for the mAb vs. SOC only treatment arm with 28 vs. 32% (rate ratio of 0.87, 95% CI 0.77-0.98) respectively for patients who were not receiving invasive mechanical ventilation at randomisation. More recently, it was announced that the casirivimab/imdevimab study 2066 (NCT04426695) achieved its primary endpoint of viral load reduction within seven days of treatment in patients who had not mounted a natural antibody response of their own (seronegative) and who required low-flow or no supplemental oxygen.

The UK-RECOVERY study data also show a difference at baseline in the median time since symptom onset between the seronegative and seropositive subgroups: 7 and 10 days respectively (interquartile ranges of 4 – 10 days and 7 – 13 days), which suggests that time since symptom onset can serve as a screening tool to enrich for the seronegative population. Due to confounding or antibodies from previous infections as well as vaccination it is preferred not to use baseline serology status as an eligibility criterion for this study.

XVR011 is a single domain (heavy chain only) antibody fragment (VHH) which is linked to the Fc part of a human IgG1. The VHH component was derived from a heavy chain only antibody from a llama, which had been inoculated with severe acute respiratory syndrome coronavirus 1 (SARS-CoV-1) spike protein. When SARS-CoV-2 was identified, the VHH was found to be cross reactive to its spike protein. The VHH binds to an epitope on the receptor-binding domain of the SARS-CoV-2 spike protein and through steric hindrance, blocks the interaction of the spike protein with the ACE2 receptor and thereby prevents the virus from entering the cell and replicating. The binding site is partially hidden and is expected to be less susceptible to genetic pressure and viral mutation. The amino acid sequence of the VHH was further humanized and optimized to increase its antiviral potency. In XVR011, two VHH's are linked to a human IgG1 Fc fragment to achieve a half-life similar to that of naturally occurring antibody, i.e., approximately 21 days. The Fc has been modified to partially silence the effector function. This has been done to minimise the potential for antibody-dependent enhancement of disease. The XVR011-virus complex is expected to be cleared by non-specific mechanism such as non-specific pinocytosis.

Further details are provided in the latest version of the Investigator's Brochure (IB) (Investigator's Brochure).

2.1.1 Summary of Non-clinical Studies

The antiviral activity of XVR011 has been demonstrated in neutralisation assays *in vitro* with both the live virus and a pseudovirus, which expresses the spike protein. IC_{50} (IC_{90}) concentrations for neutralisation are in the nanomolar and picomolar ranges respectively, indicating XVR011 is a highly potent molecule. This has been confirmed and demonstrated *in vivo* in a number of studies using the Syrian Golden Hamster model of the SARS-CoV-2 infection. Various dose levels, two viral isolates and both treatment and prophylactic setting were evaluated. Viral load was assessed using viral RNA and infectious viral particle measurements primarily in the lung. Additionally, lung tissue was histologically examined. Significant reduction in viral RNA and absence of infectious viral particles was observed in a dose-related manner.

Importantly, XVR011 has maintained *in vitro* neutralisation capacity against the delta variant. All mutations observed in the RBD of the current rapidly spreading variants of interest (VOI) and variants of concern (VOC) are distant from the XVR011 binding site. Therefore, no impact of these mutations on the neutralization potency of XVR011 is expected. This was confirmed *in vitro* in a plaque reduction neutralization test (PRNT) using authentic viral isolates of the SARS-CoV-2 Alpha (B.1.1.7), Beta (B.1.351,) Delta (B.1.617.2) and Gamma (P.1) VOC, which show maintained neutralising activity.

XVR011 targets a viral protein and is not expected to bind to any human proteins. It is therefore expected not to have any effect other than neutralisation of the virus. The lack of binding to human proteins has been evaluated in a human tissue cross reactivity study as well as in a cell microarray assay which expresses approx. 5475 human proteins. Both of these studies confirmed that there is no cross-reactivity to human tissue nor binding to human proteins, respectively.

The modification to partially silence the Fc effector function is a known modification that has been evaluated in clinical trials of monoclonal antibodies in acute rejection of organ transplants. A functional assay of antibody-dependent cytotoxicity showed significantly less cytotoxicity for XVR011 compared to the molecule with an active Fc. Binding studies of XVR011 demonstrated a much lower affinity of XVR011 to the human Fc gamma receptors compared to a functional Fc.

A detailed description of the non-clinical studies is provided in the latest version of the IB (Investigator's Brochure).

2.1.2 Summary of Clinical Studies

A phase 1 clinical study (protocol EXEVIR0102) is being conducted in healthy subjects in parallel with part 1 of this study. It evaluates XVR011 as a single dose intravenous (IV) infusion and is being conducted in The Netherlands. It is a phase 1a, randomised, double-blinded, single-center, placebo-controlled, single ascending dose study in 3 groups of 10 healthy male and/or female adult subjects each. The objectives of the study are to assess the safety profile and pharmacokinetics of XVR011. Per dose cohort, 8 subjects receive XVR011 and 2 subjects receive placebo. The study is evaluating 250 mg, 500 mg and 1000 mg as a single IV infusion. Subjects were admitted to the clinical site on the day prior to study drug administration and remained at the site for 24 h after study drug infusion. After being discharged, they returned for ambulatory visits on Days 8, 15, 29, 43, 57, and Day 85.

To date, all 3 dose groups have been enrolled and have been administered study drug. The first dose group, 250 mg, has completed the study. The 500 mg and 1000 mg dose groups are still in the follow up period. There have been no reports of SAEs or infusion related reactions.

2.2 Study Rationale

Rapid neutralisation of SARS-CoV-2 before the immune system has reached the point where an excessive inflammatory reaction has been triggered may potentially reduce the morbidity and mortality associated with COVID-19. The potential of XVR011 to achieve this is being evaluated in this Phase 1/2 clinical trial in patients with symptomatic COVID-19 who are hospitalised but in whom the inflammatory component does not yet require intensive care treatment or mechanical ventilation. The objective is to neutralise the virus more rapidly and achieve a more rapid recovery, reduce the need for medical care as well as prevent the need for intensive care.

This study includes an open-label dose-finding part (Phase 1) to evaluate the safety of XVR011 and support identification of the recommended Phase 2 dose, followed by a randomised double-blind part (Phase 2) to evaluate the efficacy and safety of XVR011 compared to placebo.

As it remains important to treat as early as possible in the course of the infection and onset of pneumonia, the eligibility criteria aim to define a study population most likely to benefit in the hospital setting from an antiviral treatment (in addition to standard of care), but still allow sufficient flexibility for feasible recruitment. The oxygen saturation level is widely used as a criterium for hospital admission, however the cut-off is very variable, both across countries but also over time. Therefore the inclusion criterium evaluating pulmonary involvement uses duration of dyspnoea and/or tachypnoea with an allowed maximum of 3 days between onset and randomisation. This is complemented by the exclusion of subjects whose respiratory compromise

requires high flow oxygen therapy, any other form of non-invasive mechanical ventilation, or invasive mechanical ventilation.

For practical feasibility at the sites, the screening assessments need to be done as soon as possible after presentation with COVID-19 to evaluate eligibility based on the inclusion and exclusion criteria. If eligible, the subject may be randomised (e.g for Part 1 this means IWRS treatment registration). Within 12 hours of randomisation, a single intravenous infusion of study treatment must be started. Sites are actively encouraged to screen and treat as quickly as possible.

2.3 Benefit/Risk Assessment

There are potential benefits and risks with the administration of any new investigational medicine as the effects are not fully known. Based on the potential benefit of XVR011, it is being developed for patients with COVID-19. More detailed information about the expected benefits and risks and potential adverse events (AEs) of XVR011 may be found in the latest version of the IB (Investigator's Brochure).

2.3.1 Risk Assessment

Preliminary data from an ongoing Phase 1 study in healthy subjects evaluating the same dose levels as this study, reveal no infusion related adverse events, serious adverse events or ≥ grade 3 adverse events for all 3 dose groups evaluated, namely 250 mg, 500 mg and 1000 mg single IV infusion. Each dose groups consists of 8 subjects administered XVR011 and 2 subjects administered placebo in a blinded manner. The follow-up for these preliminary data is up to and including Day 85 post-dose for the 250 mg dose group (study completed), up to and including Day 43 visit post-dose for the 500 mg dose group and up to an including Day 29 visit post-dose for the 1000 mg dose group.

Table 2–1 Risk Assessment

Potential Risk of Clinical Significance	Summary of Data/Rationale for Risk	Mitigation Strategy
**Study Treatment XVR011**		
Potential clinical risks:<br>• Immune reaction to a foreign protein<br>• Infusion reaction	As with any protein, there is a potential that the immune system recognizes it as foreign and triggers a hypersensitivity reaction. This may vary from mild to moderate signs and symptoms but may also be a severe or life-threatening	• Ensure emergency equipment/medication is available within the treatment area.<br>• Prior to the infusion, inform the subject of possible signs/symptoms of hypersensitivity and advise the subject to

Potential Risk of Clinical Significance	Summary of Data/Rationale for Risk	Mitigation Strategy
	anaphylactic/anaphylactoid reaction.<br>Signs and symptoms may include hypotension, tachycardia, bronchospasm, dyspnoea, oedema/angio-oedema, dizziness, headache, nausea, abdominal pain, fever, rash	immediately report if any of these occur.<br>• Close monitoring by a health care professional during the infusion and for at least 2 hours afterwards is required.<br>• The infusion must be interrupted/stopped immediately in case of any sign or symptom of an infusion reaction.<br>• Depending on the severity of the symptoms, appropriate treatment should be administered according to institutional guidelines and may include antihistamines, corticosteroids, bronchodilators, vasopressors and other treatments as per Investigator judgement.
**Study Procedures**		
• Viral load testing	• The nasal swab procedure used to obtain the COVID-19 sample is uncomfortable.	• A well-trained staff will be employed to minimise the discomfort.
• Blood sample collection	• The subject might experience, redness, induration, or bruises after blood sample collection	• All measures will be taken to avoid these symptoms.
**Other**		

Potential Risk of Clinical Significance	Summary of Data/Rationale for Risk	Mitigation Strategy
• Standard of care treatment	• Since XVR011 is an investigational medicinal product and its efficacy is not yet established, the standard of care is administered to preserve the safety of the subjects.	• As per institutional standard practice.

2.3.2 Benefit Assessment

This study being the first clinical evaluation of XVR011 in patients, there is no clinical data available yet regarding the therapeutic effect of XVR011 in patients with COVID-19.

The aim of treatment with XVR011 is to neutralise the virus, to reduce the severity of the disease, and to prevent progression to severe complications such as ARDS or other major organ dysfunctions.

Non-clinical data are available, which show significant reduction in viral load in an animal model (Syrian Golden Hamster) of SARS-CoV-2 infection, as well as *in vitro* data which show neutralisation of the virus, including the Delta VOC. All dose levels being evaluated in this study have been selected to have a potential clinical efficacy. See Section 4.3.

2.3.3 Overall Benefit: Risk Conclusion

Taking into account the measures taken to minimise risk to subjects participating in this study, the potential risks identified in association with XVR011 are justified by the anticipated benefits that may be afforded to subjects with COVID-19.

3 Objectives and Endpoints

Table 3–1 Objectives and Endpoints for Part 1

PART 1 Objectives	PART 1 Endpoints
**Primary**<br>To evaluate the safety of a single intravenous XVR011 infusion in subjects hospitalised for COVID-19	• Proportion of subjects with Grade ≥ 3 treatment-related AEs within 28-day FU period<br>• Proportion of subjects with AEs (all and serious) of any grade and independent of causality within 28-day FU period<br>• Proportion of subjects with infusion-related reactions within 24 hours of treatment<br>• Proportion of subjects with hypersensitivity reactions within 28-day FU period
**Secondary**<br>To evaluate the efficacy of a single intravenous XVR011 infusion in subjects hospitalised for COVID-19	• Time to recovery (i.e., clinical status reaching level 1 to 3 of the 8-point ordinal scale) within 28-day FU period<br>• Total duration of oxygen supplementation within 28-day FU period<br>• Proportion of subjects requiring mechanical ventilation within 28-day FU period<br>• Proportion of subjects requiring ICU transfer within 28-day FU period<br>• Time to hospital discharge<br>• Proportion of subjects with clinical status evolving to each level of the 8-point ordinal scale at Days 2, 3, 4, 8, 15, 22 and Day 29, compared to baseline<br>• Proportion of subjects with COVID-19 related symptoms<br>• All-cause mortality rate within 28-day FU period
To evaluate the antiviral activity of a single intravenous XVR011 infusion in subjects hospitalised for COVID-19	• Change from baseline (Day 1 pre-dose) in the viral load (RT-qPCR) of nasopharyngeal samples at Day 1, Day 3, Day 8 and day of discharge

PART 1 Objectives	PART 1 Endpoints
**Exploratory**<br>To evaluate the effect of a single intravenous XVR011 infusion on disease biomarkers in subjects hospitalised for COVID-19	• Blood biomarkers (including but not limited to CRP, LDH, D-dimers, fibrinogen, absolute neutrophil count, lymphocyte count) at baseline (Day 1 pre-dose), on Days 2, 3, 4, 8, 15, 22 and on day of discharge<br>• Optional blood sample for future exploratory biomarker testing at baseline (Day 1 pre-dose) and Day 3
To evaluate the pharmacokinetics (PK) of XVR011, following a single intravenous infusion in subjects hospitalised for COVID-19	• Blood concentrations of XVR011 and PK parameters (C_{max}, AUC, $t_{1/2}$) on Days 1, 2, 4, 8, 15 and Day 29.
To evaluate the immunogenicity of XVR011, following a single intravenous infusion in subjects hospitalised for COVID-19	• ADAs and nAbs at baseline (Day 1 pre-dose), Day 15, and Day 29

ADA = anti-drug antibody; AE = adverse event; AUC = area under the plasma concentration-time curve; C_{max} = maximum observed plasma concentration; COVID-2019= coronavirus disease 2019; CRP = C-reactive protein; FU = follow-up; ICU = intensive care unit; LDH = lactate dehydrogenase; nAb = neutralising anti-drug antibody; RT-qPCR = reverse transcriptase quantitative polymerase chain reaction; $t_{1/2}$ = apparent plasma terminal elimination half-life.

Table 3–2 Objectives and Endpoints for Part 2

PART 2 Objectives	PART 2 Endpoints
**Primary**<br>To evaluate the efficacy of a single intravenous XVR011 infusion in subjects hospitalised for COVID-19, compared to placebo	• Time to sustained recovery (i.e., clinical status reaching level 1 to 3 of the 8-point ordinal scale) within 28-day FU period; where recovery must be confirmed to be maintained at level 3 or lower for 14 consecutive days
**Secondary**<br>To further evaluate the efficacy of a single intravenous XVR011 infusion in subjects hospitalised for COVID-19, compared to placebo	• Proportion of subjects who are alive and do not require any form of mechanical ventilation [a] at Day 8<br>• Proportion of subjects requiring any form of mechanical ventilation [a] within 28-day FU period<br>• Proportion of subjects requiring ICU transfer within 28-day FU period<br>• Time to sustained hospital discharge (i.e. discharge with no re-hospitalisation after initial discharge for treatment of COVID-19 related events in the following 14 days) within the 90 day FU period<br>• Total duration of oxygen supplementation within 28-day FU period<br>• Proportion of subjects with clinical status evolving to each level of the 8-point ordinal scale at Days 2, 3, 4, 8, 15, 22 and Day 29, compared to baseline<br>• Proportion of subjects with COVID-19 related symptoms<br>• All-cause mortality rate within 28-day FU period
To evaluate the antiviral activity of a single intravenous XVR011 infusion in subjects hospitalised for COVID-19, compared to placebo	• Change from baseline (Day 1 pre-dose) in the viral load (RT-qPCR) of nasopharyngeal samples at Day 1, Day 3, Day 8 and day of discharge

PART 2 Objectives	PART 2 Endpoints
To evaluate the safety of a single intravenous XVR011 infusion in subjects hospitalised for COVID-19, compared to placebo	• Proportion of subjects with Grade ≥ 3 treatment-related AEs within 28-day FU period<br>• Proportion of subjects with AEs (all and serious) of any grade and independent of causality within 28-day FU period<br>• Proportion of subjects with infusion-related reactions within 24 hours of treatment<br>• Proportion of subjects with hypersensitivity reactions within 28-day FU period
**Exploratory**<br>To evaluate the effect of a single intravenous XVR011 infusion on disease biomarkers in subjects hospitalised for COVID-19	• Blood biomarkers (including but not limited to CRP, LDH, D-dimers, fibrinogen, absolute neutrophil count, lymphocyte count) at baseline (Day 1 pre-dose), on Days 2, 3, 4, 8, 15, 29 and on day of discharge<br>• Optional blood sample for future exploratory biomarker testing at baseline (Day 1 pre-dose) and Day 3
Additional evaluation of COVID-19 symptoms during the extended follow up period.	• COVID-19 related symptoms on Day 60 and Day 90
Characterise viral genotype	• At baseline and Day 8
To determine the PK parameters of XVR011, following a single intravenous infusion in subjects hospitalised for COVID-19	• Blood concentrations of XVR011 and PK parameters (C_{max}, AUC, $t_{1/2}$) on Days 1, 4, 8, 15, 29, 60 and 90.
To evaluate the immunogenicity of XVR011, following a single intravenous infusion in subjects hospitalised for COVID-19, compared to placebo	• ADAs and nAbs at baseline (Day 1 pre-dose), Day 15 and Day 29.

[a] Any form of mechanical ventilation includes but not limited to high flow oxygen therapy, continuous positive airway pressure, invasive mechanical ventilation

ADA = anti-drug antibody; AE = adverse event; AUC = area under the plasma concentration-time curve; C_{max} = maximum observed plasma concentration; COVID-2019= coronavirus disease 2019; CRP = C-reactive protein; FU = follow-up; ICU = intensive care unit; LDH = lactate dehydrogenase; nAb = neutralising anti-drug antibody; RT-qPCR = reverse transcriptase quantitative polymerase chain reaction; $t_{1/2}$ = apparent plasma terminal elimination half-life.

4 Study Design

4.1 Overall Design

This is a 2-part Phase 1/2 clinical study to evaluate the efficacy and safety of XVR011 in subjects hospitalised for COVID-19.

Part 1 (Phase I): Part 1 consists of an open-label, single ascending dose, Phase 1 study to evaluate the safety of XVR011 administered as a single intravenous infusion, at 3 dose levels, and evaluate the PK, pharmacodynamic (PD), and initial efficacy of the treatment. Eligible subjects will be enrolled in 2 successive cohorts of 9 subjects, each. Two subjects in each cohort will be sentinel subjects monitored for at least 24 hours (first sentinel subject will be monitored for 24 hours before the second sentinel subject is treated and monitored for 24 hours) before the remaining subjects can be dosed and decision to open Cohort 2 will depend on the review of data collected from subjects enrolled in Cohort 1 after the last subject was treated and monitored for 24 hours. The same strategy will be followed for Cohort 3 (see Section 6.6.1 for details).

A phase 1a study (protocol EXEVIR0102) in healthy subjects is being conducted in parallel with Part 1 of this study. It is evaluating the same dose levels, namely 250, 500 and 1000 mg as a single IV infusion in a dose ascending design, with a placebo control. Per dose level, 8 subjects receive a single IV infusion of XVR011, and 2 subjects receive a single IV infusion if placebo in a blinded manner. The study will generate PK data and safety data not confounded by the disease state. An interim analysis of study EXEVIR0102 will be performed when all dose groups have received treatment and when the last dose group (1000 mg) has completed the 8-day follow-up. As discussed in section 4.3 Justification for Dose, the main driver for the dose determination is the target serum concentration and number of days this is exceeded, in addition to safety. The PK parameters from the healthy volunteers are considered predictive for the patient setting. The safety profile will be reviewed of the 3 different dose levels in the healthy subject study.

The IDMC will be asked to make a recommendation to progress to Part 2 (phase 2 design) of this study with the recommended dose based on the interim analysis of the Phase 1a study in 30 healthy subjects, supplemented with the available data from Part 1 of this study, even if not all Part 1 cohorts have reached the planned enrolment. This is due to recruitment challenges in Part 1 of this study resulting from low hospitalisation rate and reluctance of sites to participate and enrol patients in a phase I trial.

Part 2 (Phase 2): Part 2 consists of a double-blind, randomised, placebo-controlled Phase 2 study to evaluate the efficacy, safety, PK, and PD of XVR011 administered at the recommended dose level based on the IDMC review and recommendation. A total of 408 eligible subjects will be

randomised in a 2:1 ratio to receive either XVR011 (272 subjects) or placebo (136 subjects). Standard of care (SoC), according to local requirements will be allowed, except if listed in Table 6.5 Forbidden Concomitant Medications. Stratification will be done at randomisation for country and for fully vaccinated/prior SARS-CoV-2 infection. Subgroup analyses will include factors such as disease state, baseline serology, baseline viral load levels and duration of COVID-19 symptoms prior to study treatment.

For both Part 1 and Part 2 the study will consist of 3 periods: Screening (Day -1), Treatment period (Day 1), and Endpoint Assessment period (7 to 8 visits, Day 2 to Day 29); for Part 2 there will be an additional Follow-up period (2 visits, Day 60 and Day 90):

- **Screening (Day -1)**: Subjects who are hospitalised and provide informed consent will undergo screening assessments as soon as possible after presentation with COVID-19 to evaluate their eligibility based on the inclusion and exclusion criteria. If eligible, the subject may be randomised (e.g for Part 1 this means IWRS treatment registration).

- **Treatment period (Day 1)**: Within 12 hours of randomisation, a single intravenous infusion of study treatment will be started under medical supervision for the whole duration of the infusion and for two hours after the end of the infusion. This treatment will be administered in addition to SoC treatment.

- **Endpoint assessment period (Day 2 to Day 29)**: Subjects will remain hospitalised until at least 24 hours after study treatment administration. Hospital discharge after this period is at the discretion of the Investigator and will be based on the clinical condition of the subject. Study assessments will be performed every day up to Day 4 and then weekly until Day 29. Subjects who are discharged from the hospital before the Day 29 visit will return to the study site for each study visit until Day 29 (see Section 6.7 for details). Day 29 is considered as the EOS for part 1.

- **Extended follow-up period (Day 30 to Day 90) for part 2 only:** Subjects will return to the study site for 2 additional visits, on day 60 and day 90. Day 90 is considered as the EOS for part 2.

4.2 Scientific Rationale for Study Design

This 2-part study consists of an open-label, single ascending dose, Phase 1 study to evaluate the safety, PK, PD and initial efficacy of XVR011 administered at 3 dose levels. Due to low rates of infection and hospitalisation at the Part 1 investigational sites and consequent recruitment challenges, a healthy subject Phase 1a study is being conducted in parallel which evaluates the

same 3 dose levels: 250 mg, 500 mg and 1000 mg. The safety and PK data from the healthy subject study, together with available data from Part 1 of this study will determine the recommended dose of XVR011, that will be used in a double-blind, randomised, placebo-controlled Phase 2 study to evaluate the efficacy, safety, and PK/PD of XVR011.

The dose-finding part of this first-in-human study (Part 1) follows the recommendations of the European Medicines Agency Guideline on strategies to identify and mitigate risks for first-in-human clinical trials with investigational medicinal products (EMEA/CHMP/SWP/28367/07). Refer to Section 4.3 for the selection of the starting dose.

For Part 2, a double-blind, placebo-controlled design is appropriate and standard for an efficacy and safety study. This design will minimise bias and provide reference data (i.e., data from placebo-treated subjects), which will aid in the interpretation of results.

The primary efficacy endpoint for Part 2 is the time to sustained recovery within a 28-day follow up period, where recovery is defined based on an 8-point ordinal scale for COVID-19 clinical status used in previous studies involving subjects hospitalised for COVID-19 (Beigel et al 2020), and which is sustained for at least 14 consecutive days.

The proposed population is subjects, in whom the viral load is causing mild to moderate symptoms and who require supportive therapy such as hospitalised monitoring, treatment or supplemental oxygen but who are not in need of mechanical ventilation (i.e., level 4 or 5 of the 8-point ordinal scale). The aim of treatment with XVR011 is to neutralise the virus, to reduce the severity of the disease, and to prevent progression to severe complications such as ARDS or other major organ dysfunctions. Recovery is defined as reaching level 3 of the 8-point ordinal scale (i.e., subjects are hospitalised but no longer require supplemental oxygen or ongoing medical care for COVID-19) or less (subjects are no longer hospitalised for treatment of COVID-19). See also Section 2.1 and Section 2.2.

Study treatment will be administered in addition to SoC to limit the risk of disease progression for subjects randomised to placebo. For Part 2, concomitant use of remdesivir will be allowed as SoC, according to local guidelines.

Other small molecule antiviral agents such as molnupiravir and PF-07321332/ ritonavir may be continued at the discretion of the Investigator if they have been started prior to hospital admission for treatment of COVID-19 in accordance with their authorised use. However, they are not allowed if they are to be started once the subject is in hospital and in screening or already enrolled in the study.

Treatment with a SARS-CoV-2 neutralising monoclonal antibody for the current infection is not allowed with 4 weeks before and during the study, because of the similar mechanism of action and long half-life.

Stratification will be done at randomisation for country and for fully vaccinated/prior SARS-CoV-2 infection.

Subgroup analyses will include factors such as disease state, baseline serology, baseline viral load levels and duration of COVID-19 symptoms prior to study treatment.

Secondary endpoints to further evaluate efficacy include total duration of oxygen supplementation, proportion of subjects requiring mechanical ventilation or ICU transfer, subject-reported assessment of COVID-19-related symptoms following FDA Guidance for Industry (September 2020) and changes from baseline in viral load to evaluate antiviral activity.

Safety assessments will include standard adverse event reporting, physical examination, vital signs and ECGs, as well as the assessment of infusion related reactions (IRR) and hypersensitivity reaction.

4.3 Justification for Dose

In Part 1, the Phase 1 component of this protocol, 3 dose levels will be evaluated in a sequential manner, starting with the lowest dose. The dose determination is based on a combination of PK simulations to predict the PK profile of XVR011 in humans and a dose response model based on the antiviral efficacy of XVR011 in the hamster viral infection model. The simulated human PK profiles are based on PK data from a cynomolgus PK study of a single IV administration of 2 dose levels of XVR011 (5 mg/kg and 50 mg/kg). The hamster studies evaluated various doses, two viral isolates, therapeutic and prophylactic settings as well as RNA and infectious viral particle endpoints and lung histopathology. They correlate the *in vivo* efficacy with serum concentrations and allow for various sources of variability. The data from the hamster viral infection studies show a binary response, i.e. responder or non-responder when using a desired viral load decrease relative to plasma concentrations of XVR011. Fitting a logistic regression function to these data allow a threshold concentration to be determined corresponding to a specified probability of being a responder. The median serum concentration required for a 95% probability of being a responder is 27 µg/mL. An even more conservative approach is to use the upper bound (97.5th percentile) of the 95% probability of response corresponding to a serum concentration of 50 µg/mL.

Considering the simulated PK profiles of various doses as well as a period of at least 10 days within which it is considered relevant for the intended clinical setting to maintain serum

concentrations above the threshold level, the following doses (administered as a single IV infusion) are to be evaluated in Phase 1 of this study:

- Cohort 1 dose: 250 mg. This dose is expected to result in exposure above the median threshold value 27 µg/mL for 7 days.
- Cohort 2 dose: 500 mg. This dose is expected to result in exposure above the upper bound threshold value of 50 µg/mL for 7 days.
- Cohort 3 dose: 1000 mg. This dose is expected to result in exposure above the upper bound threshold value of 50 µg/mL for 21 days.

4.4 End of Study Definition

A subject is considered to have completed the study if he/she has completed all phases of the study, including the EOS visit.

The end of the study is defined as the date of the last visit of the last subject in the study.

5 Study Population

Prospective approval of protocol deviations to recruitment and enrolment criteria, also known as protocol waivers or exemptions, is not permitted.

The study population will include adult male and female subjects who tested positive for SARS-CoV-2 by RT-PCR or antigen test and who require medical care for COVID-19 in hospital setting (i.e., level 4 or 5 of the 8-point ordinal scale), but do not require non-invasive mechanical ventilation (includes for example high flow oxygen therapy, positive pressure ventilation), invasive mechanical ventilation or intensive care treatment.

5.1 Inclusion Criteria

Subjects are eligible to be included in the study only if all of the following criteria apply:

Demography

1. Is ≥18 years of age inclusive, at the time of signing the informed consent.
2. Is male or female

Type of Subject and Disease Characteristics

3. Has ongoing SARS-CoV-2 infection confirmed by positive RT-PCR test and/or positive antigen test ≤72 hours of randomisation*. If the positive test result is older than 72 hours, a positive re-test (RT-PCR or antigen) during screening is required.
4. If dyspnoea and/or tachypnoea are present, an onset of at most 3 days prior to randomisation* is allowed.
5. Requires medical care in hospital for COVID-19 (i.e., level 4 or 5 of the 8-point ordinal scale).

**For Part 1 randomisation means IWRS treatment registration.*

Contraception

Contraceptive use by men or women should be consistent with local regulations regarding the methods of contraception for those participating in clinical studies.

Male Subjects:

6. A male subject must agree to use contraception as detailed in Appendix 5 of this protocol during the treatment period and for at least 90 days after the last dose of study treatment.

Female Subjects:

7. A female subject is eligible to participate if she is not pregnant (see Appendix 5), not breastfeeding, and at least one of the following conditions applies:
   - Not a woman of childbearing potential (WOCBP) as defined in Appendix 5.

     OR

   - A WOCBP who agrees to follow the contraceptive guidance in Appendix 5 during the treatment period and for at least 90 days after the last dose of study treatment.

Informed Consent

8. Is able and willing to give written informed consent for the study, or if due to COVID-19 restrictions written consent is not possible, verbal informed consent in the presence of an impartial witness will be obtained and documented in the medical record.

5.2 Exclusion Criteria

Subjects are excluded from the study if any of the following criteria apply:

Medical Conditions

1. Has a resting respiratory rate ≥ 30 per minute without oxygen supplementation.
2. Has severe or critical COVID-19 requiring non-invasive (e.g. high flow oxygen therapy) or invasive mechanical ventilation and/or intensive care treatment.
3. Has ongoing clinically significant thromboembolic event or coagulopathy, according to clinical, laboratory and/or radiological assessment.
4. Has a neutrophil to lymphocyte ratio > 4.
5. Has alanine aminotransferase (ALT) level >5 × upper limit of normal (ULN) or an aspartate aminotransferase (AST) level >5 × ULN. Known severe liver disease such as cirrhosis with Child-Pugh Class B or C.
6. Has renal impairment with estimated creatinine clearance < 30 mL/min/1.73 m^2, or is receiving continuous renal replacement therapy, haemodialysis.
7. Has any other medical condition, which in the opinion of the Investigator, would impact the safety of the subject or interfere with the subject’s ability to comply with the study and follow-up procedures.

8. Has active malignancy for which cytotoxic or cytostatic treatment is being administered or has been administered less than 3 months prior to enrolment, or which is in (pre-)terminal stage.
9. Has active chronic hepatitis B or C.

Prior/Concomitant Therapy

10. Has received any of the following therapies:
    a. Any SARS-CoV-2 human intravenous immunoglobulin (hIVIG), convalescent plasma (convalescent plasma therapy) from a person who recovered from SARS-CoV-2 infection within 30 days prior to the study treatment administration.
    b. Any other investigational or authorised biological medical product with a SARS-CoV-2 neutralising activity including but not limited to any monoclonal antibody within 30 days prior to the study treatment administration.
11. Has received an investigational or approved vaccination against SARS-CoV-2 within 14 days before study treatment start for the first vaccination dose and 7 days for subsequent vaccination doses, including 'booster shots'.

Other Exclusions

12. Has known allergy or hypersensitivity reaction to any monoclonal antibody or to any components of study treatment.
13. For WOCBP:
    a. Pregnancy or a positive urine pregnancy test (within 72 hours prior to treatment). If the urine test is positive or cannot be confirmed as negative, a serum pregnancy test is required, and the subject will be excluded if the serum pregnancy test is positive.
    b. Breastfeeding.

Exclusion Criteria only Applicable to Part 1

14. Has received or is receiving concomitant treatment with medicinal products with potential or demonstrated anti-SARS-CoV-2 (antiviral) activity, including but not limited to remdesivir, within 30 days prior to the study treatment administration.
15. Has renal impairment with estimated creatinine clearance < 60 mL/min/1.73 m^2.

16. Has morbid obesity (body mass index > 35 kg/m^2), uncontrolled diabetes, cardiac insufficiency, uncontrolled high blood pressure, or any clinically significant (in the opinion of the Investigator) hepatic, pulmonary, gastrointestinal, genitourinary, endocrine, immunologic, metabolic, neurologic, or haematological disease.

5.3 Lifestyle Considerations

No lifestyle restrictions are required.

5.4 Screen Failures

Screen failures are defined as subjects who consent to participate in the clinical study but are not subsequently entered in a cohort (Part 1) or randomly assigned to study intervention (Part 2). A minimal set of screen failure information is required to ensure transparent reporting of screen failure subjects to meet the Consolidated Standards of Reporting Trials (CONSORT) publishing requirements and to respond to queries from regulatory authorities. Minimal information includes demography, reason for screen failure (e.g., eligibility requirements failed), and any SAEs.

Individuals who do not meet the criteria for participation in this study (screen failure) may not be rescreened.

5.4.1 Screening and Enrolment Log and Subject Identification Numbers

The subject’s enrolment will be recorded in the Screening and Enrolment Log.

Upon enrolment, each subject will receive a unique subject identification number.

6 Study Treatment

Study treatment is defined as any investigational treatment(s), marketed product(s), or placebo intended to be administered to a study subject according to the study protocol.

XVR011 is for intravenous infusion only. It must not be administered as an intravenous push or bolus injection. Emergency resuscitative treatments, such as antihistamines, corticosteroids, bronchodilators, vasopressors must be available within the treatment area.

6.1 Study Treatments Administered

Table 6–1 Study Treatments Administered

Arm/ Cohort Name:	XVR011 Dose level 1	XVR011 Dose level 2	XVR011 Dose level 3	Placebo
**Study Treatment Name:**	XVR011			Placebo
**Type:**	Biologics			-
**Dosage Formulation:**	Solution for infusion			-
**Unit Dose Strength(s):**	50 mg/mL (500 mg of XVR011 in 10 mL)			-
**Dosage Level(s):**	250 mg	500 mg	1000 mg	Placebo
**Route of Administration:**	IV infusion			
**Use:**	Experimental			Placebo-comparator
**IMP and NIMP:**	IMP	IMP	IMP	NIMP
**Sourcing:**	XVR011 provided centrally by the Sponsor.<br>0.9% sodium chloride IV bag and accessories provided centrally by the Sponsor.			0.9% sodium chloride IV bag and accessories provided centrally by the Sponsor.
**Dosing Instructions:**	Study treatment diluted in 0.9% sodium chloride sterile solution for IV infusion to a concentration of 10 mg/mL (see Section 6.2 for details).			

**Packaging and Labelling:**	Study treatment will be provided in 20 mL neutral type I glass Schott vials (filled with 10 mL extractable volume). Each vial will be labelled per country requirement. Commercially available IV bags will be used for the dilution of XVR011.		Commercially available IV bags will be used.

Abbreviations: IV = intravenous; IMP = investigational medicinal product; NIMP = non-investigational medicinal product.

The single dose treatment will be administered by a health care professional as an IV infusion (Table 6–2). The subject will be closely monitored by a health care professional during the IV infusion and for at least 120 minutes afterward. Sentinel subjects in Part 1 should be monitored for 24 hours after the infusion Prior to the infusion, the subject should be informed of possible signs/symptoms of hypersensitivity and advised to immediately report if any of these occur.

The concentration of the prepared study treatment infusion is 10 mg/mL.

Table 6–2 IV Infusion Details for Part 1

Dose	IV infusion volume	IV infusion rate	IV infusion time
250 mg (Dose Level 1)	25 mL	1.0 mL/minute (10 mg/minute)	25 minutes
500 mg (Dose Level 2)	50 mL		50 minutes
1000 mg (Dose Level 3)	100 mL		100 minutes

Abbreviation: IV = intravenous.

For Part 2, one dose will be selected from the 3 doses evaluated in Part 1, with the corresponding IV infusion volume.

The study treatment will be administered as an infusion using a sterile, non-pyrogenic, low-protein binding 0.2 µm in-line or add-on filter. After administration, the line should be flushed with 0.9% sodium chloride sterile solution for injection.

It will be documented if the full volume of the study treatment infusion was administered. If not the full volume, e.g. in case of interruption due to an infusion related reaction, then an approximation of the infused volume must be made and documented.

Infusion reactions: as with any protein, infusion reactions/hypersensitivity reactions may occur. This may vary from mild to moderate signs and symptoms but may also be a severe or life-threatening anaphylactic/anaphylactoid reaction. Signs and symptoms may include hypotension, tachycardia, bronchospasm, dyspnoea, oedema/angio-oedema, dizziness, headache, nausea, abdominal pain, fever, rash.

Guidance in case of an infusion reaction based on severity of the reaction is summarised in Table 6–3.

Table 6–3 Guidance in Case of an Infusion Reaction Based on Severity Of The Reaction

Infusion reaction	Study treatment actions	Other treatment (see also institutional standard practice for infusion reactions)
Grade 1 - mild	Reduce rate of infusion	Consider symptomatic treatment
Grade 2 - moderate	Interrupt infusion<br>Restart infusion if signs/symptoms have responded well to symptomatic treatment<br>Infusion should be restarted at a slower rate:<br>If symptoms worsen or recur, permanently stop infusion<br>If symptoms do not worsen or do not recur, continue infusion	Administer symptomatic treatment e.g. antihistamines, NSAIDs wait for signs/symptoms to improve before re-starting infusion
Grade 3 - severe	Permanently stop infusion	Administer antihistamines, corticosteroids, bronchodilators, IV fluids, vasopressors and other treatments as per Investigator judgement
Grade 4 – life-threatening	Permanently stop infusion	Administer antihistamines, corticosteroids, bronchodilators, IV fluids, vasopressors and other life support treatments as per Investigator judgement

Abbreviation: NSAID = nonsteroidal anti-inflammatory drugs

6.2 Preparation, Handling, Storage, and Accountability

The dose will be administered using an IV bag containing 0.9% (w/v) sodium chloride sterile solution, for an infusion concentration of 10 mg/mL, and delivered through an IV administration set with a sterile, non-pyrogenic, low-protein binding 0.2 μm in-line or add-on filter.

For Part 1, the study treatment is open-label XVR011 and the study treatment infusion bag is prepared at the hospital (central) pharmacy in controlled and aseptic conditions according to the instructions in the investigational medicinal product (IMP) handling instructions.

For Part 2, study treatment is blinded and is either XVR011 or placebo and is prepared at the hospital (central) pharmacy in controlled and aseptic conditions by an unblinded pharmacist as a covered IV infusion bag according to the instructions in the IMP handling instructions.

For placebo treatment, the covered IV bag with only 0.9% sodium chloride solution will be used.

The pharmacist or designee must maintain a log to confirm appropriate temperature conditions have been maintained during transit for all study treatment received, and any discrepancies are reported and resolved before the use of the study treatment.

The IV bags of study treatment (both XVR011 and placebo) will be covered to mask the slight colour difference between the XVR011 solution and placebo to maintain the study blind.

The study treatment infusion bag/cover will be identified with a sticker specifying the protocol number, subject ID number, date and time of preparation. The study treatment infusion must be administered as soon as possible after preparation and no later than 2 hours of the date and time of preparation noted on the bag/cover.

Only subjects enrolled in the study may receive study treatment and only authorised site staff may supply or administer study treatment. All study treatments must be stored in a secure, environmentally controlled, and monitored (manual or automated) area in accordance with the labelled storage conditions with access limited to the Investigator and authorised site staff.

The Investigator, institution, or the head of the medical institution (where applicable) is responsible for study treatment accountability, reconciliation, and record maintenance (e.g., receipt, reconciliation, and final disposition records).

Regarding storage, XVR011 will be provided as a single use vial with one vial per package. The vial must be stored in the package in which it is provided. The package should be stored at -20°C at the central pharmacy. For further details on storage, see the IMP handling instructions.

Vials will be prepared at the central pharmacy and may be discarded after use after accountability check. For details on IMP accountability, see the IMP handling instructions.

Further guidance and information for the final disposition of unused study treatment are provided in the IMP handling instructions.

6.3 Measures to Minimise Bias

6.3.1 Part 1

Part 1 is a sequential open-label study; potential bias will be reduced by the review of data by an IDMC.

6.3.2 Part 2

6.3.2.1 Randomisation and Blinding

Part 2 is a randomised double-blind study.

All subjects will be randomised centrally using an interactive response technology (IRT) to ensure that treatment assignment is unbiased and concealed from study subjects and the Investigator. The Investigator will be provided with usernames and passwords for randomisation purposes and to unblind the study treatment in case of emergency, where he/she considers it essential to know what treatment the subject was taking. The IRT will be designed to send a confirmation (by fax and/or notification email) to the site for every transaction performed by the Investigator.

Each subject will be assigned to one of the 2 arms of the study according to the randomisation schedule provided by the Parexel Informatic Manager utilising a validated computer program. Details of the procedure are described in the IRT Manual provided to all sites.

Subjects will be randomly assigned in a 2:1 ratio to receive study treatment (XVR011 or placebo). Stratification will be done at randomisation for country and for fully vaccinated/prior SARS-CoV-2 infection. Any EMA approved, FDA approved or WHO recommended vaccine will be taken into account. The original recommended vaccination frequency (either 1 or 2 injections depending on the vaccine) will be considered as fully vaccinated, i.e. the more recent recommendations for a booster shot are not required for the stratification definition.

The placebo IV bag will present the same external appearance with the same composition, except for the active ingredient. IV bag cover will be added to keep the blinding on the IV bag.

Randomisation data will be kept strictly confidential, accessible only to authorised persons, until the time of unblinding of the allocated treatment of all study subjects after locking the database upon termination of the study.

Subjects, Investigators and/or employees of the investigational site will remain blinded to each subject’s assigned study treatment throughout the course of the study, unless in case of emergency.

In order to maintain this blind, an otherwise uninvolved third party (unblinded pharmacist) will be responsible for the reconstitution and dispensation of all study treatment and will endeavour to ensure that there are no differences in time taken to dispense following randomisation.

The Sponsor will also be blinded during the study as they will not have direct access to the randomisation list or to the medication list. Specific sponsor representatives, independent of the study team will be unblinded to perform specific unblinded tasks.

In the event of a quality assurance audit, the auditor(s) will be allowed access to unblinded study treatment records at the site(s) to verify that randomisation/dispensing has been done accurately.

6.3.2.2 Procedures for breaking the blind

In case of emergency, unblinding of the treatment code will be done through IRT (IWRS). The treatment group will be disclosed, and confirmation will follow (by fax and/or notification email). The IRT will promptly notify the Sponsor whenever a treatment code is unblinded.

An emergency medical contact service will be also available for part 2. The emergency phone number will be available on the subject ID card for the subjects enrolled in Part 2. This out-of-business-hours service allows the non-investigator healthcare professionals to access information on a 24-hour basis about study treatment being received by their subjects and providing the subject's study treatment allocation (i.e. unblinding).

6.4 Study Treatment Compliance

Subjects are dosed at the site and will receive study treatment directly from the Investigator or designee, under medical supervision. The date and time of infusion, IV volume, infusion rate, and any infusion interruptions and restart will be recorded in the source documents and recorded in the CRF. The dose of study treatment and study subject identification will be confirmed at the time of dosing by a member of the study site staff other than the person administering the study treatment.

6.5 Concomitant Therapy

Any medication or vaccine (including over-the-counter or prescription medicines, vitamins, and/or herbal supplements) that the subject is receiving at the time of enrolment or receives during the study must be recorded along with:

- reason for use
- dates of administration including start and end dates
- dosage information including dose and frequency

The Medical Monitor should be contacted if there are any questions regarding concomitant or prior therapy.

6.5.1 Standard of Care for COVID-19

Study treatments (XVR011 or placebo) should be administered in addition to SoC treatment per local institutional standards at the study site.

Owing to the extensive global effort to investigate and develop pharmacological and biological treatments of COVID-19 (including antiviral, immune-based, and adjunctive therapies), treatment recommendations are frequently updated and may vary across regions. The following treatment options are currently recommended by several regulatory authorities and/or expert panels:

- **Dexamethasone**: On 2 September 2020, based on a systematic review and meta-analysis of the results of 8 randomised controlled trials, WHO published a strong recommendation for the use of systemic corticosteroids in severely ill patients with COVID-19 and a conditional recommendation not to use systemic corticosteroids in patients with non-severe COVID-19 (WHO/2019-nCoV/Corticosteroids/2020.1, WHO REACT Working Group et al 2020). On 25 September 2020, the Infectious Diseases Society of America (IDSA) guideline panel also suggested dexamethasone for patients with severe COVID-19 (SpO_2 ≤94% on room air, including patients on supplemental oxygen). If dexamethasone is not available, then alternative glucocorticoids may be used. The IDSA guideline panel advises against glucocorticoids for patients with COVID-19 without hypoxemia requiring supplemental oxygen (Bhimraj et al 2020).
- **Remdesivir**: In May 2020, the FDA issued an emergency use authorisation (EUA) for remdesivir for the treatment of suspected or laboratory-confirmed COVID-19 in adults and children hospitalised with severe disease (FDA News Release 2020a). In June 2020, the FDA had warned against the use of remdesivir in combination with hydroxychloroquine (FDA News Release 2020b). In July 2020, the European Commission granted conditional marketing authorisation for remdesivir for the treatment of COVID-19 in adults and adolescents from 12 years of age with pneumonia who require supplemental oxygen (European Commission 2020), following recommendation from the EMA (EMA 2020), making remdesivir the first authorised COVID-19 antiviral treatment in the EU. In October 2020, the FDA approved remdesivir for use in adult and paediatric patients 12 years of age and older and weighing at least 40 kg (about 88 pounds) for the treatment of COVID-19 requiring hospitalisation (FDA News Release 2020c). In November 2020, the WHO issued a conditional recommendation against the use of remdesivir in hospitalised patients, regardless of disease severity, as there is

currently no evidence that remdesivir improves survival and other outcomes in these patients (WHO Living Guideline: Therapeutics and COVID-19, 20 November 2020).

See Table 6–4 for restricted concomitant medications and Table 6–5 for forbidden concomitant medications.

Table 6–4 Restricted concomitant medications Part 1 only

Medication	Restriction
Drugs showing possible anti-SARS-CoV-2 (antiviral) activity (including but not limited to remdesivir)	Within 30 days prior to study treatment administration and during the study up to the EOS

COVID-19 = coronavirus disease 2019; EOS = end of study; SARS-CoV-2 = severe acute respiratory syndrome coronavirus 2

Table 6–5 Forbidden concomitant medications

Medication	Restriction
SARS-CoV-2 hIVIG	Within 30 days prior to study treatment administration and during the study up to the EOS
Convalescent plasma (convalescent plasma therapy) from a person who recovered from SARS-CoV-2 infection	
Investigational or authorised biological medical product with a SARS-CoV-2 neutralising activity including but not limited to any monoclonal antibody	
Small molecule anti-SARS-CoV-2 medical product	From signature of ICF up to EOS

EOS = end of study; hIVIG = human intravenous immunoglobulin, mAb = monoclonal antibody, SARS-CoV-2 = severe acute respiratory syndrome coronavirus 2

6.6 Dose Modification and Stopping/Suspension Rules

An IDMC will be established and an IDMC charter agreed prior to screening of any subjects for the study. The IDMC charter will describe the role and responsibilities of the IDMC and will reflect the stopping/suspension rules described in this section. The IDMC will review the safety of study subjects throughout the study as described in the IDMC charter. At pre-specified time points described in this section, the IDMC will do a formal review of available data and make a recommendation with supporting rationale to the Sponsor who will act accordingly. There are currently no stopping/suspension rules foreseen in Part 2, however the IDMC will be requested to specify in their recommendation after Part 1, if these should be implemented. If so, then the IDMC should endorse a sponsor proposal or make a proposal for such stopping/suspension rules for Part 2.

Occurrence of below noted serious adverse reactions or Grade ≥3 treatment-related AEs will be considered irrespective of whether or not they are in the same system-organ-class.

6.6.1 Dose Escalation and Stopping/Suspension Rules During Part 1

Two subjects in Cohort 1 will act as sequential sentinel subjects with at least 24 hours between the treatment of the first and the second sentinel subject. Each sentinel subject will be closely monitored for 24 hours after receiving XVR011. The Sponsor will confirm that relevant adverse reactions as defined below have or have not occurred in this 24-hour period.

- If neither of the two sentinel subjects experience any serious adverse reactions (i.e. SAEs considered at least possibly related to the treatment) and if at most 1 sentinel subject experiences a Grade ≥ 3 treatment-related AE during this period, then after sponsor review, the remaining 7 subjects will be enrolled and treated.
- If one or more of the two sentinel subjects experience a serious adverse reaction or if both sentinel subjects experience a Grade ≥ 3 treatment-related AE, enrolment will be suspended pending a review of the data by the IDMC, who will make a recommendation regarding study continuation.
- If at any point during the Cohort, serious adverse reactions are reported in 1 or more subjects, or Grade ≥ 3 treatment related AEs are reported in 2 or more subjects, enrolment will be suspended pending the outcome of an IDMC review.

After all 9 subjects are enrolled and treated in Cohort 1 and the last subject has been treated and monitored for 24 hours, the sponsor will confirm that all relevant adverse reactions as defined below are reported.

- If 1 or more of the 9 subjects in Cohort 1 experience a serious adverse reaction, or if 2 or more of the 9 subjects experience a Grade ≥ 3 treatment-related AE, the IDMC will review available data to make a recommendation on starting Cohort 2.
- If no subject has experienced a serious adverse reaction and if < 2 subjects have experienced a Grade ≥ 3 treatment-related AE in Cohort 1, enrolment in Cohort 2 will start after sponsor review only.

In any case, the first two subject enrolled in Cohort 2 will be sentinel subjects, and the enrolment of the subjects will follow the same rules as for Cohort 1.

After all 9 subjects are enrolled and treated in Cohort 2 and the last subject has been treated and monitored for 24 hours, the sponsor will confirm that all relevant adverse reactions as defined below are reported.

- If 1 or more of the 9 subjects in Cohort 2 experience a serious adverse reaction or, if 2 or more of the 9 subjects experience a Grade ≥ 3 treatment-related AE, the IDMC will review available data to make a recommendation on starting Cohort 3.
- If no subject has experienced a serious adverse reaction and if < 2 subjects have experienced a Grade ≥ 3 treatment-related AE in Cohort 2, enrolment in Cohort 3 will start after sponsor review only.

In any case, the first two subject enrolled in Cohort 3 will be sentinel subjects, and the enrolment of the subjects will follow the same rules as for Cohort 2.

6.6.2 Dose Selection for and Start of Part 2

A phase 1a study (protocol EXEVIR0102) in healthy subjects is being conducted in parallel with part 1 of this study. It is evaluating the same dose levels, namely 250, 500 and 1000 mg as a single IV infusion in a dose ascending design, with a placebo control. Per dose level, 8 subjects receive a single IV infusion of XVR011 and 2 subjects receive a single IV infusion of placebo in a blinded manner. The study will generate PK data and safety data not confounded by the disease state. An interim analysis of study EXEVIR0102 will be performed when all dose groups have received treatment and when the last dose group (1000 mg) has completed the 8-day follow-up. As discussed in section 4.3 Justification for Dose, the main driver for the dose determination is the target serum concentration and number of days this is exceeded, in addition to safety. The PK parameters from the healthy subjects are considered predictive for the patient setting. The safety profile will be reviewed of the 3 different dose levels in the healthy subject study.

The IDMC will be asked to make a recommendation to progress to Part 2 (Phase 2 design) of this study with the recommended dose based on the interim analysis of the Phase 1a study in 30 healthy subjects, supplemented with the available data from Part 1 of this study, even if not all Part 1 cohorts have reached the planned enrolment. This is due to recruitment challenges in Part 1 of this study resulting from low hospitalisation rate and reluctance of sites to participate and enrol patients in a Phase 1 clinical study.

6.7 Discharge from the Hospital and Follow-up

Subjects may be discharged from hospital at the earliest 24 hours after study treatment administration and at the Investigator's discretion if their condition improves. All Visit 3 (Day 2) assessments must be performed before hospital discharge.

Study assessments will be performed every day up to Day 4 and then weekly until Day 29. For Part 2 there are 2 additional visits, namely on Day 60 and Day 90. After discharge from hospital, subjects will return to the study site for each per protocol study visit until the EOS (Day 29 for Part 1 / Day 90 for Part 2).

Subjects who are discharged from hospital before the EOS (Day 29 for Part 1 and Day 90 for Part 2) and outside the per protocol planned visit schedule must have an additional visit to perform the discharge assessments.

Subjects who are discharged from the hospital before the EOS (Day 29 for Part 1 and Day 90 for Part 2) on the day of a per protocol planned visit (e.g. Day 2, Day 3, Day 4, Day 8, Day 15, Day 22, Day 29 (part 2 only) and Day 60 (part 2 only) must have the discharge assessments done in addition to the per protocol planned visit assessments if not yet covered (assessments only need to be performed once).

6.8 Treatment After the End of the Study

After the end of the study, no further treatment with XVR011 is expected. Subjects will continue receiving SoC treatment as clinically indicated.

7 Discontinuation of Study Treatment and Subject Discontinuation

7.1 Discontinuation of Study Treatment

Study treatment is administered as a single IV infusion on Day 1. This infusion must be permanently stopped in case of Grade 3 or 4 infusion related reactions (see Section 6.1).

7.2 Subject Discontinuation/Withdrawal from the Study

A subject may withdraw from the study at any time at his/her own request or may be withdrawn at any time at the discretion of the Investigator for safety, behavioural, or administrative reasons. The subject will be definitively discontinued from both the study treatment and the study at that time.

If the subject withdraws consent for disclosure of future information, the Sponsor may retain and continue to use any data collected before such a withdrawal of consent.

If a subject withdraws from the study, he/she may request the destruction of any samples taken and not tested, and the Investigator must document this in the site study records.

At the time of discontinuing from the study, if possible, an early discontinuation (ED) visit should be conducted, as shown in the SoA. Refer to the SoA for data to be collected at the time of study discontinuation and follow-up and for any further evaluations that need to be completed.

When a subject participation has been terminated prior to completing the study, the reason is recorded as follows:

- Pregnancy
- Adverse event
- Treatment failure
- Protocol violation
- Lost to follow-up
- Subject withdrew consent at own request
- Other

7.3 Loss of Subjects to Follow-Up

A subject will be considered lost to follow-up if he or she repeatedly fails to return for scheduled visits and is unable to be contacted by the study site.

The following actions must be taken if a subject fails to return to the clinic for a required study visit:

- The site must attempt to contact the subject and reschedule the missed visit as soon as possible (and within the visit window, where one is defined) and counsel the subject on the importance of maintaining the assigned visit schedule and ascertain whether or not the subject wishes to and/or should continue in the study.
- In cases in which the subject is deemed lost to follow-up, the Investigator or designee must make every effort to regain contact with the subject (where possible, 3 telephone calls and, if necessary, a certified letter to the subject's last known mailing address or local equivalent methods). These contact attempts should be documented in the subject's medical record.
- Should the subject continue to be unreachable, he/she will be considered to have withdrawn from the study.

Discontinuation of specific sites or of the study as a whole is handled as part of the appendix on Governance, Appendix 1, Section 10.1.9.

8 Study Assessments and Procedures

Study procedures and their timing are summarised in the SoA (Table 1-1for Part 1 and Table 1–2 for Part 2). Protocol waivers or exemptions are not allowed.

Adherence to the study design requirements, including those specified in the SoA, is essential and required for study conduct.

All screening evaluations must be completed and reviewed to confirm that potential subjects meet all eligibility criteria. The Investigator will maintain a screening log to record details of all subjects screened and to confirm eligibility or record reasons for screening failure, as applicable.

Procedures conducted as part of the subject's routine clinical management (e.g., blood count) and obtained before signing of the ICF may be utilised for screening or baseline purposes provided the procedures met the protocol-specified criteria and were performed within the time frame defined in the SoA.

The maximum amount of blood collected from each subject over the duration of the study, including any extra assessments that may be required per protocol, will not exceed 500 mL.

Repeat or unscheduled samples may be taken for safety reasons or for technical issues with the samples.

Informed Consent

Informed consent must be documented, according to Appendix 1, Section 10.1.3.

Eligibility Criteria

All inclusion and exclusion criteria will be reviewed by the Investigator or designee to ensure that the subject qualifies for the study.

Medical History

A medical history will be obtained by the Investigator or qualified designee. The medical history will collect all active conditions and any prior condition that the Investigator considers to be clinically relevant, including previous SARS-CoV-2 infection(s).

Current COVID-19 History

Details regarding COVID-19 signs and symptoms with date of onset and stop date or ongoing will be collected.

The confirmation of the current SARS-CoV-2 infection by positive RT-PCR test and/or positive antigen test, will be captured.

The presence of anti-SARS-CoV-2 antibodies in blood samples will be determined at baseline by a central laboratory.

Prior and Concomitant Medications Review

The Investigator or qualified designee will review prior medication use and record prior medications taken by the subject within 30 days prior to screening. COVID-19 vaccination details will be recorded without limitation in time.

The Investigator or qualified designee will record medication, if any, taken by the subject during the study through the last visit. Concomitant medications will be recorded throughout the full duration of the study (or longer if related to an SAE).

8.1 Efficacy Assessments

8.1.1 Clinical Status According to 8-point Ordinal Scale

The clinical status will be assessed at the timepoints specified in the SoA (Table 1-1for Part 1 and Table 1–2 for Part 2), using an 8-point ordinal scale (see Appendix 2), previously used in Study NCT04280705 (Beigel et al 2020).

Recovery will be defined as achieving a clinical status ≤ 3 on the 8-point ordinal scale. This will be considered 'sustained recovery' if the clinical status remains ≤ 3 for the subsequent 14 consecutive days. Time to sustained recovery will be defined as the time between treatment administration and the first occurrence of recovery which is subsequently shown to be sustained..

Deterioration in the clinical condition of the subject such that intensive care is required will be considered a treatment failure and the end of study visit should be performed.

8.1.2 Respiratory Parameters

Oxygen supplementation details, respiratory rate and oxygen saturation (on room air and with oxygen supplementation) will be assessed at the timepoints specified in the SoA (Table 1-1for Part 1 and Table 1–2 for Part 2):

- All non-invasive and invasive oxygen supplementation, including all forms of mechanical ventilation will be reported. The following information will be collected: type, flow rate and start & stop dates.
- Respiratory rate will be measured as part of vital signs (see Section 8.2.2).
- Oxygen saturation will be measured using pulse oximetry measurements, in accordance with the site standard operating procedures, on a medical-grade medical device. Measurements will be taken with a probe on the fingertip or earlobe (keeping the same methodology throughout the study, if possible) and recorded as percent oxygenated haemoglobin.

8.1.3 Viral Load

The antiviral activity of study treatments will be assessed by measuring the nasopharyngeal viral loads.

Nasopharyngeal swabs will be collected at the timepoints specified in the SoA (Table 1-1for Part 1 and Table 1–2 for Part 2). Samples will be tested for the presence of SARS-CoV-2 using the SARS-CoV-2 RT-qPCR assay. Virologic assessments will include quantitative measures of viral RNA and will be conducted by a central laboratory. At baseline and on Day 8, the genotype of the SARS-CoV-2 virus will be determined.

8.1.4 COVID-19 Related Symptoms

Fourteen key COVID-19-related symptoms will be rated by the subject at the timepoints specified in the SoA (Table 1-1for Part 1 and Table 1–2 for Part 2), using a questionnaire administered by the Investigator or designee (see Appendix 3). The questionnaire will include 1 question per symptom. Each symptom will be rated from 0 (no symptoms) to 3 (severe or ≥ 5/day for vomiting and diarrhoea), except sense of smell and taste rated from 0 (same as usual) to 2 (no sense of smell/taste).

8.2 Safety Assessments

Planned timepoints for all safety assessments are provided in the SoA (Table 1-1for Part 1 and Table 1–2 for Part 2).

8.2.1 Physical Examinations

A complete physical examination (screening only) will include assessments of the cardiovascular, respiratory, musculoskeletal, lymphatic, and neurological systems as well as general appearance, skin, eyes, ears, nose, throat and extremities examinations. Height will be measured and recorded at screening only and weight will be measured and recorded at screening and EOS/ED visit.

A COVID-19 symptom targeted physical examination (all other visits) will be performed to evaluate for status of COVID-19 associated symptoms, focusing at a minimum on respiratory function. Other body systems will be examined depending upon subject’s complaints/symptoms.

Investigators should pay special attention to clinical signs related to previous serious illnesses.

8.2.2 Vital Signs

Oral, tympanic or rectal temperature (keeping the same methodology throughout the study, if possible), pulse rate, respiratory rate, and blood pressure will be assessed before blood collection for laboratory tests.

Blood pressure and pulse measurements will be assessed in a supine position with a completely automated device. Manual techniques will be used only if an automated device is not available.

Blood pressure and pulse measurements should be preceded by at least 5 minutes of rest for the subject in a quiet setting without distractions (e.g., television, cell phones). Pulse will be measured once. Blood pressure will be measured with 2 consecutive readings recorded at intervals of at least 1 minute. The average of the 2 blood pressure readings will be recorded on the CRF.

8.2.3 Electrocardiograms

Single 12-lead ECG will be obtained as outlined in the SoA (Table 1-1for Part 1 and Table 1–2 for Part 2) using an ECG machine that automatically calculates the heart rate and measures PR, QRS and QT intervals.

8.2.4 Clinical Safety Laboratory Assessments

Safety laboratory tests listed in Table 8–1 will be performed by the local laboratory at the timepoints as outlined in the SoA (Table 1-1for Part 1 and Table 1–2 for Part 2). Additional tests may be performed at any time during the study as determined necessary by the Investigator or required by local regulations.

Table 8–1 Protocol-Required Safety Laboratory Assessments

Laboratory Assessments	Parameters
Haematology	White cell blood count with differential count, including absolute neutrophil count [a] and lymphocyte count [a]<br>Red blood cell count and haemoglobin<br>Platelet count
Clinical Chemistry	Sodium, potassium, chloride, calcium, phosphate<br>ALT, AST, ALP, total or direct bilirubin<br>Total protein, albumin<br>Serum creatinine<br>CRP [a] and LDH [a]<br>For Part 2 - additionally[a]:<br>Ferritin<br>cTnT or cTnI (choice dependent on local laboratory)
Coagulation	Prothrombin time and activated partial thromboplastin time<br>D-dimers [a] and fibrinogen [a]
Other screening tests	Estimated creatinine clearance according to Cockcroft-Gault formula

ALP = alkaline phosphatase; ALT=alanine aminotransferase; AST = aspartate aminotransferase; CRP = C-reactive protein; LDH = lactate dehydrogenase.

a. Measured as disease biomarkers (see Section 8.8)

The results of protocol-required safety laboratory assessments should be recorded in the CRF. In addition, the Investigator must review the laboratory report, document this review, and record any clinically relevant changes occurring during the study in the AE section of the CRF. The laboratory reports must be filed with the source documents. Clinically relevant abnormal laboratory findings are those which are not associated with the underlying disease, unless judged by the Investigator to be more severe than expected for the subject’s condition.

All laboratory tests with values considered clinically abnormal during the 28-day FU period should be repeated until the values return to normal or baseline or are no longer considered clinically significant by the Investigator or Medical Monitor.

- If such values do not return to normal/baseline within a period of time judged reasonable by the Investigator, the aetiology should be identified, where possible, and the Sponsor notified.
- All protocol-required laboratory assessments, as defined in Table 8–1, must be conducted in accordance with the laboratory manual and the SoA.
- If laboratory values from laboratory assessments not specified in the protocol and performed at the institution’s local laboratory result in the need for a change in subject management or

are considered clinically relevant by the Investigator (e.g., are considered to be an SAE or an AE or require dose modification), then the results must be recorded in the CRF.

8.3 Adverse Events and Serious Adverse Events

The definitions of AEs and SAEs can be found in Appendix 4.

AEs will be reported by the subject (or, when appropriate, by a caregiver, surrogate, or the subject's legally authorised representative).

The Investigator and any qualified designees are responsible for detecting, documenting, and reporting events that meet the definition of an AE or SAE and remain responsible for following up AE that are serious, considered related to the study treatment or the study, or that caused the subject to discontinue the study (see Section 7).

8.3.1 Time Period and Frequency for Collecting AE and SAE Information

All AEs and SAEs will be collected from the signing of ICF to the EOS visit at the timepoints specified in the SoA (Table 1-1for Part 1 and Table 1–2 for Part 2).

All SAEs will be recorded and reported to the Sponsor or designee within 24 hours, as indicated in Appendix 4. The Investigator will submit any updated SAE data to the Sponsor within 24 hours of it being available.

Investigators are not obliged to actively seek AEs or SAEs after the conclusion of study participation. However, if the Investigator learns of any SAE, including death, at any time after a subject has been discharged from the study, and he/she considers the event to be reasonably related to the study treatment or study participation, the Investigator must promptly notify the Medical Monitor (or the Sponsor after study closure).

8.3.2 Method of Detecting AEs and SAEs

The method of recording, evaluating, and assessing causality of AEs and SAEs and the procedures for completing and transmitting SAE reports are provided in Appendix 4.

Care will be taken not to introduce bias when detecting AEs and/or SAEs. Open-ended and non-leading verbal questioning of the subject is the preferred method to inquire about AE occurrence.

8.3.3 Follow-up of AEs and SAEs

After the initial AE/SAE report, the Investigator is required to proactively follow each subject at subsequent visits/contacts. All SAEs, and non-serious AEs of special interest (as defined in Section 8.3.6), will be followed until resolution, stabilisation, until the event is otherwise explained, or the subject is lost to follow-up (as defined in Section 7.3). Further information on follow-up procedures is given in Appendix 4.

8.3.4 Regulatory Reporting Requirements for SAE

Prompt notification (within 24 hours, see Appendix 4) by the Investigator to the Sponsor of an SAE is essential so that legal obligations and ethical responsibilities towards the safety of subjects and the safety of a study treatment under clinical investigation are met.

The Sponsor has a legal responsibility to notify both the local regulatory authority and other regulatory agencies about the safety of a study treatment under clinical investigation. The Sponsor will comply with country-specific regulatory requirements relating to safety reporting to the regulatory authority, IRB/IEC, and Investigators.

An Investigator who receives an Investigator safety report describing an SAE or other specific safety information (e.g., summary or listing of SAE) from the Sponsor will file it along with the Investigator's Brochure and will notify the IRB/IEC, if appropriate according to local requirements.

8.3.5 Pregnancy

- Details of all pregnancies in female subjects and, if indicated, female partners of male subjects will be collected after the study treatment and until the EOS.
- If a pregnancy is reported, the Investigator should inform the Sponsor within 24 hours of learning of the pregnancy and should follow the procedures outlined in Appendix 5, to collect information and determine the pregnancy outcome.
- Abnormal pregnancy outcomes (e.g., spontaneous abortion, foetal death, stillbirth, congenital anomalies, ectopic pregnancy) are considered to be SAEs.

8.3.6 Adverse Events of Special Interest

Adverse events of special interest include infusion related reactions (including hypersensitivity or anaphylaxis during or within 24 hours of XVR011 infusion and hypersensitivity reactions (other than IRR) and will be reported using the same process as for AEs).

All subjects should be closely observed under medical supervision during the XVR011 infusion administration and for ≥ 120 minutes thereafter. Vital signs, including blood pressure, heart and respiratory rates and body temperature will be evaluated for possible hypersensitivity reactions. Any type of ECG will be performed if a subject experiences cardiac symptom.

Emergency equipment, such as adrenaline, antihistamines, corticosteroids, and respiratory support (inhalational therapy, oxygen, and artificial ventilator) must be available. Medication for infusion reactions (IRR) should be available for immediate use.

As with any protein, infusion reactions (IRR)/hypersensitivity reactions may occur. This may vary from mild to moderate signs and symptoms but may also be a severe or life-threatening anaphylactic/anaphylactoid reaction. Signs and symptoms may include hypotension, tachycardia, bronchospasm, dyspnoea, oedema/angio-oedema, dizziness, headache, nausea, abdominal pain, fever, rash. Guidance in case of an infusion reaction based on severity of the reaction provided in Table 8–2.

Table 8–2 Guidance in case of an infusion reaction based on severity of the reaction

Infusion reaction	Study treatment actions	Other treatment (see also institutional standard practice for infusion reactions)
Grade 1 - mild	Reduce rate of infusion	Consider symptomatic treatment
Grade 2 - moderate	Interrupt infusion<br>Restart infusion if signs/symptoms have responded well to symptomatic treatment<br>Infusion should be restarted at a slower rate:<br>If symptoms worsen or recur, permanently stop infusion<br>If symptoms do not worsen or do not recur, continue infusion	Administer symptomatic treatment e.g. antihistamines, NSAIDs wait for signs/symptoms to improve before re-starting infusion
Grade 3 - severe	Permanently stop infusion	Administer antihistamines, corticosteroids, bronchodilators, IV fluids, vasopressors and other treatments as per Investigator judgement
Grade 4 – life-threatening	Permanently stop infusion	Administer antihistamines, corticosteroids, bronchodilators, IV fluids, vasopressors and other life support treatments as per Investigator judgement

Abbreviation: NSAID = nonsteroidal anti-inflammatory drugs.

The medical staff has to be trained in resuscitation and immediate intensive care has to be easily accessible in case of severe life-threatening events.

8.4 Treatment of Overdose

For this study, any dose of XVR011 greater than the intended dose (i.e., dose level 1, dose level 2 or dose level 3 depending on cohort and study part) within a 24-hour time period will be considered an overdose.

The Sponsor does not recommend specific treatment for an overdose.

In the event of an overdose, the Investigator should:

1 Contact the Medical Monitor immediately.
2 Closely monitor the subject for AE/SAE and laboratory abnormalities until the EOS.
3 Provide appropriate treatment as per the clinical judgement of the Investigator for any such AE/SAE.
4 Obtain plasma samples for PK analysis as per SoA.
5 Document the quantity of the excess dose as well as the duration of the overdosing in the CRF.

8.5 Pharmacokinetics

Serum samples of approximately 1 mL will be collected for measurement of serum concentrations of XVR011 as specified in the SoA. The timing of sampling may be altered during the course of the study based on newly available data (e.g., to obtain data closer to the time of peak plasma concentrations) to ensure appropriate monitoring. Instructions for the collection and handling of biological samples will be provided in a separate laboratory manual. The actual date and time (24-hour clock time) of each sample will be recorded.

Samples will be used to evaluate the PK of XVR011 and processed as described in the laboratory manual. Genetic analyses will not be performed on these samples. Subject confidentiality will be maintained.

During Part 2, drug concentration information that would unblind the study will not be reported to investigative sites or blinded personnel until the study has been unblinded.

Any changes in the timing of timepoints for any planned study assessments must be documented and approved by the relevant study team member and then archived in the Sponsor and site study files but will not constitute a protocol amendment. The IRB/IEC will be informed of any safety issues that require alteration of the safety monitoring scheme or amendment of the ICF.

Samples may be stored for a maximum of 5 years following the last subject's last visit for the study at a facility selected by the Sponsor to enable further analysis of immune responses to XVR011.

8.6 Pharmacodynamics

For the assessment of viral load in nasopharyngeal refer to Section 8.1.3.

For biomarkers refer to Section 8.8.

8.7 Genetics

Genetics are not evaluated in this study.

8.8 Biomarkers

Blood samples for biomarker research are required and will be collected from all subjects in this study as specified in the SoA (Table 1-1for Part 1 and Table 1–2 for Part 2). The following disease biomarkers will be analysed by a local laboratory at the site along with clinical safety laboratory parameters (see Table 8–1):

- C-reactive protein (CRP)
- Lactate dehydrogenase (LDH)
- D-dimers
- Fibrinogen
- Absolute neutrophil count
- Lymphocyte count
- Part 2: additionally, ferritin and cardiac troponin T or I

Two additional samples will be collected at Day 1 and Day 3 and stored for a maximum of 3 years following the last subject's last visit for the study at a facility selected by the Sponsor to enable further analysis of biomarker responses to XVR011. These samples are only collected for subjects who provide specific consent for this. The blood volume required to be collected will be outlined in the inform consent and laboratory handling instructions.

Samples will be tested to evaluate the effect of XVR011 on the disease biomarkers in subjects hospitalised for COVID-19.

8.9 Immunogenicity Assessments

Antibodies to XVR011 will be evaluated in serum samples collected from all subjects, according to the SoA.

Serum samples will be screened for antibodies binding to XVR011 and the titre of confirmed positive samples will be reported. Other analyses may be performed to verify the stability of antibodies to XVR011 and/or further characterise the immunogenicity of XVR011.

The detection and characterisation of antibodies to XVR011 will be performed using a validated assay method by or under the supervision of the Sponsor. Additional samples may be collected to

further characterised and/or evaluated antibodies for their ability to neutralise the activity of the study treatment. For more details see laboratory manual.

Instructions for the collection and handling of biological samples will be provided in a separate laboratory manual.

Samples may be stored for a maximum of 3 years following the last subject’s last visit for the study at a facility selected by the Sponsor to enable further analysis of immune responses to XVR011.

8.10 Medical Resource Utilisation and Health Economics

Health Economics/Medical Resource Utilisation and Health Economics parameters are not evaluated in this study.

9 Statistical Considerations

9.1 Statistical Hypotheses

Part 1: No formal hypothesis is postulated for Part 1.

Part 2: In Part 2, capacity of XVR011 in reducing time to recovery (sustained) as compared to placebo will be tested in a superiority setting. The primary endpoint will be tested using the stratified log-rank test. It is assumed that the placebo and active cumulative survival curves for TTR do not follow an exponential distribution. Moreover, the HR cannot be assumed as constant during the 28-day FU period.

9.2 Sample Size Determination

Part 1: There is no formal sample size calculation for Part 1. Considering the target of XVR011 is a viral protein and the absence of any binding with human proteins in preclinical studies, 9 subjects per cohort are considered adequate as an initial assessment of the safety of a single dose of XVR011 in this population.

Part 2: The sample size for the Phase 2 part of the study is based on the primary endpoint, time to recovery defined as reaching level 1 to 3 of the 8-point scale within 28-day FU period and which is maintained at or below level 3 for the subsequent, consecutive 14 days; a 2:1 randomisation with a one-sided alpha of 0.025, 80% power and allowing for a 15% drop-out rate. The sample size required to test the primary endpoint is obtained by comparing with the log-rank test, the piecewise exponential cumulative survivals described in Table 9–1 and is 408 subjects allowing 275 events to be observed. A total of 408 subjects will be randomised, 272 to the XVR011 arm and 136 to the placebo arm.

Table 9–1 Determination of Sample Size with Different Time to Recovery Assumptions for Placebo and XVR011 Treatment

	Placebo Cumulative "Survival"*	XVR011 Cumulative "Survival"*	HR
**Day 1**	100%	100%	
**Day 5**	80%	50%	3.11
**Day 7**	50%	25%	1.47
**Day 29**	20%	20%	0.24

* "Survival" refers to absence of recovery

Note: Sample size scenarios: Primary endpoint time to recovery, 2:1 randomisation, 1-sided alpha = 0.025 and power 80%.

9.3 Populations for Analyses

For purposes of analysis, the following analysis sets are defined:

Table 9–2 Populations for Analysis

**Population (Analysis Set)**	**Description**
Enrolled Analysis Set (EAS)	All subjects who signed the ICF (including screening failures).
Full Analysis Set (FAS)	The FAS is defined under an intent-to-treat population principle.<br>**Part 1**: The FAS comprises all subjects to whom study treatment has been dispensed.<br>**Part 2**: The FAS comprises all subjects to whom study treatment has been randomised. Statistical analyses will be based on study treatment groups as per randomisation, irrespective of the study treatment actually received.
Per Protocol Analysis Set (PPS), if applicable	**Part 1**: The PPS comprises all the FAS subjects who received study treatment.<br>**Part 2**: The PPS comprises all the FAS subjects who received study treatment and have a positive RT-PCR test at baseline (central laboratory).<br>In both Part 1 and Part 2, a subject will be completely excluded from the PPS in case of protocol deviations affecting study analyses.
Safety Analysis Set (SAF)	The safety analysis set consists of all subjects who started infusion with study treatment. Subjects will be analysed according to the study treatment they actually received. A precise definition of "as actually received" will be added in the statistical analysis plan (SAP).
PK Analysis Set (PKS)	The PK analysis set consists of all subjects in the safety analysis set with at least one drug concentration value.

EAS = enrolled analysis set; FAS = full analysis set; ICF = informed consent form; PK = pharmacokinetics; PPS = per protocol analysis set.

In SAF, safety data of subjects will be listed and summarised under the treatment actually received independently of total amount of the treatment received. As an example, a subject who was randomised into the control arm (placebo) but received EXVR011 by mistake, will be considered as:

- a placebo-treated subject in the FAS and PPS
- an EXVR011 treated subject in the SAF

The FAS will be the primary analysis set for all efficacy analyses and the PPS will be used to demonstrate robustness of results for the primary efficacy endpoints.

9.4 Statistical Analyses

The SAP will be developed and finalised as early as possible after finalisation of the study protocol. Below is a description of planned statistical analyses. Further details are presented in the SAP.

9.4.1 General Considerations

All statistical analyses will be performed by Parexel. All statistical analysis will be performed using the latest available version of SAS® (SAS Institute Inc., Cary, North Carolina, United States of America [USA]), version 9.2 or higher.

Continuous data will be summarised by treatment group using descriptive statistics (number, mean, median, standard deviation [SD], minimum, and maximum). Categorical data will be summarised by treatment group using frequency tables (frequency and percent).

Unless other way specified, baseline will be taken as the last available assessment prior to dosing on Day 1.

All statistical tests will be 2-sided and will be performed at the 5% level of significance, thus 95% confidence intervals (CIs) will be computed, unless otherwise stated.

A protocol deviation is defined as any change, divergence, or departure from the study design or procedures of a study protocol. Protocol deviations can lead to the exclusion of a subject from the PPS. Protocol deviations will be reviewed and classified at the Blinded Data Review Meeting (BDRM) prior to unblinding; the exclusion of subjects from PPS will be decided at BDRM.

Unless otherwise stated, missing data will not be imputed.

9.4.2 Primary Efficacy Endpoint

For Part 1, refer to the safety analyses in Section 9.4.4.

For Part 2, the primary analysis for the primary endpoint (i.e., time to sustained recovery within the 28-day FU period) will be tested using the stratified (stratification factors used at randomisation) log-rank test in the FAS (if applicable) for the comparison of the 2 treatment arms. Kaplan-Meier curves will be presented, and median time to recovery and 95% CI will be calculated based on the Kaplan-Meier estimates. The Bayesian piecewise exponential model (Ibrahim et al 2001) will be used to calculate the time variant hazard ratios and 95% CI's. In this model, the stratification factors at the time of randomisation (if applicable) will be used as covariates. In these subjects with mild to moderate COVID-19 disease, no mortality or small mortality rate is

anticipated; in case of death events, a censoring time will be imputed to handle deaths similarly to cases of no recovery by Day 29.

Further details on the methodology applied for the statistical analysis will be described in the protocol and statistical analysis plan (SAP).

9.4.3 Secondary Efficacy Endpoints

Proportion of subjects who are alive and do not require any form of mechanical ventilation at Day 8. Proportion of subjects requiring non-invasive or invasive, mechanical ventilation, proportion of subjects requiring intensive care unit (ICU) transfer, and all-cause mortality rate within 28-day FU period will be presented.

Total duration (days) of oxygen supplementation within 28-day FU period, change from baseline in the nasopharyngeal viral load (RT-qPCR) will be reported as continuous endpoints.

Proportion of subjects with clinical status evolving to each level of the 8-point ordinal scale at Days 2, 3, 4, 8, 15, 22, and Day 29, compared to baseline, will be tabulated in a shift table.

Time to event secondary endpoints will be analysed using a methodology similar to the analysis of the primary efficacy endpoint.

In Part 1, the secondary endpoints will be summarised descriptively.

In Part 2, the comparison between XVR011 and placebo will be conducted for every secondary endpoint and resulting p-values will be presented.

9.4.4 Safety Analyses

All safety summaries and analyses will be made on the safety analysis set.

AEs will be coded with Medical Dictionary for Regulatory Activities (MedDRA).

AEs will be summarised by study part and treatment group, below incidences will be provided for the 28-day follow-up period:

- Proportion of subjects with any AEs
- Proportion of subjects with any AEs by system organ class (SOC) and preferred term (PT)
- Proportion of subjects with any AEs by SOC and PT and worst toxicity grade
- Proportion of subjects with Grade ≥ 3 AEs
- Proportion of subjects with Grade ≥ 3 AEs by SOC and PT

- Proportion of subjects with serious AEs (SAEs)
- Proportion of subjects with SAEs by SOC and PT
- Proportion of subjects with SAEs by SOC and PT and worst toxicity grade
- Proportion of subjects with SUSARs
- Proportion of subjects with treatment-related AEs
- Proportion of subjects with treatment-related AEs by SOC and PT
- Proportion of subjects with treatment-related AEs by SOC and PT and worst toxicity grade
- Proportion of subjects with treatment-related Grade ≥ 3 AEs
- Proportion of subjects with treatment-related Grade ≥ 3 AEs by SOC and PT
- Proportion of subjects with any AEs leading to discontinuation
- Proportion of subjects with any AEs leading to hospitalisation
- Proportion of subjects with treatment-related SAEs
- Proportion of subjects with treatment-related SAEs by SOC and PT
- Proportion of subjects with treatment-related SAEs by SOC and PT and worst toxicity grade
- Proportion of subjects with IRR (i.e., within 24 hours of start of infusion)
- Proportion of subjects with hypersensitivity reactions (other than those reported as IRRs) within 28-day FU period

Observed values of clinical safety laboratory parameters and vital signs will be summarised by study part, treatment group and by timepoints using descriptive statistics. Shift tables for change from baseline will be provided.

Shift table for changes from baseline in 12-lead ECGs (i.e., shifting from a normal to an abnormal clinical assessment or vice versa) will be presented as well.

9.4.5 Pharmacokinetic Analyses

Pharmacokinetic analyses will be performed by Parexel on behalf of ExeVir Bio BV using non-compartmental analysis.

When possible, the following PK parameters will be assessed for XVR011:

- C_{max}: maximum observed plasma concentration
- AUC: area under the plasma concentration-time curve
- $t_{1/2}$: apparent plasma terminal elimination half-life

XVR011 plasma concentrations and PK parameters will be listed and summarised by XVR011 treatment groups and study parts.

9.4.6 Other Analyses

Levels of ADAs and nAbs will be reported as continuous endpoints.

Biomarker exploratory analyses will be described in the SAP.

9.5 Interim Analyses

An interim analysis will be conducted when all subjects enrolled in Part 1 have completed the Day 4 visit, including all available safety, tolerability, preliminary efficacy, PK and PD data.

The SAP will describe the planned interim analyses in greater detail.

9.6 Data Monitoring Committee

An IDMC will review data and provide recommendations during the study. For details on the IDMC, refer to Appendix 10.1.5.

10 Supporting Documentation and Operational Considerations

10.1 Appendix 1: Regulatory, Ethical, and Study Oversight Considerations

10.1.1 Regulatory and Ethical Considerations

This study will be conducted in accordance with the protocol and with:

- Consensus ethical principles derived from international guidelines, including the Declaration of Helsinki and Council for International Organisations of Medical Sciences (CIOMS) International Ethical Guidelines.
- Applicable ICH Good Clinical Practice (GCP) Guidelines.
- Applicable laws and regulations.

The protocol, protocol amendments, ICF, Investigator Brochure, and other relevant documents (e.g., advertisements) must be submitted to an IRB/IEC by the Investigator and reviewed and approved by the IRB/IEC before the study is initiated.

Any amendments to the protocol will require IEC/IRB approval before implementation of changes made to the study design, except for changes necessary to eliminate an immediate hazard to study subjects.

The Investigator will be responsible for the following:

- Providing written summaries of the status of the study to the IRB/IEC annually or more frequently in accordance with the requirements, policies, and procedures established by the IRB/EC.
- Notifying the IRB/IEC of SAE or other significant safety findings as required by IRB/IEC procedures.
- Overall conduct of the study at the site and adherence to requirements of 21 CFR, ICH GCP guidelines, the IRB/IEC, European regulation 536/2014 for clinical studies (if applicable), and all other applicable local regulations.

10.1.2 Financial Disclosure

Investigators and sub-Investigators will provide the Sponsor with sufficient, accurate financial information in accordance with local regulations to allow the Sponsor to submit a complete and accurate financial certification or disclosure statements to the appropriate regulatory authorities. Investigators are responsible for providing information on financial interests during the course of the study and for 1 year after completion of the study.

10.1.3 Informed Consent Process

The Investigator or his/her representative will explain the nature of the study to the subject or his/her legally authorised representative and answer all questions regarding the study.

Subjects must be informed that their participation is voluntary. Subjects or their legally authorised representative will be required to sign a statement of informed consent that meets the requirements of 21 CFR 50, local regulations, ICH guidelines, Health Insurance Portability and Accountability Act (HIPAA) requirements, where applicable, and the IRB/IEC or study site.

The medical record must include a statement that written informed consent was obtained before the subject was enrolled in the study and the date the written consent was obtained. The authorised person obtaining the informed consent must also sign the ICF. If due to COVID-19 restrictions written consent is not possible, verbal informed consent in the presence of an impartial witness will be obtained and documented in the medical record.

Subjects must be re-consented to the most current version of the ICF(s) during their participation in the study.

A copy of the ICF(s) must be provided to the subject or the subject’s legally authorised representative.

If a protocol amendment is required, the ICF may need to be revised to reflect the changes to the protocol. If the ICF is revised, it must be reviewed and approved by the appropriate IEC/IRB and signed by all subjects subsequently enrolled in the study as well as those currently enrolled in the study.

10.1.4 Data Protection

Subjects will be assigned a unique identifier by the Sponsor. Any subject records or datasets that are transferred to the Sponsor will contain the identifier only; subject names or any information which would make the subject identifiable will not be transferred.

The subject must be informed that his/her personal study-related data will be used by the Sponsor in accordance with local data protection law. The level of disclosure must also be explained to the subject, who will be required to give consent for their data to be used as described in the informed consent.

The subject must be informed that his/her medical records may be examined by Clinical Quality Assurance auditors or other authorised personnel appointed by the Sponsor, by appropriate IRB/IEC members, and by inspectors from regulatory authorities.

10.1.5 Committees Structure (IDMC)

An IDMC will be responsible for closely reviewing the data collected during Part 1 and providing their recommendations in the following situations:

- During enrolment of Cohort 1, Cohort 2 or Cohort 3 (Part 1), if one or both of the 2 sentinel subjects experience a serious adverse reaction or if both sentinel subjects experience Grade ≥ 3 treatment-related AEs, enrolment will be suspended pending a review of the data by the IDMC, who will make a recommendation regarding study continuation.
- If at any point during Cohort 1, Cohort 2 or Cohort 3, a serious adverse reaction is reported in 1 or more subjects, or Grade ≥ 3 treatment related AEs are reported in 2 or more subjects, enrolment will be suspended pending the outcome of an IDMC review.
- After all 9 subjects are enrolled and treated in Cohort 1 and the last subject has been treated and monitored for 24 hours, if 1 or more of the 9 subjects experience a serious adverse reaction, or if 2 or more of the 9 subjects experience a Grade ≥ 3 treatment-related AE, the IDMC will review available data to make a recommendation on starting Cohort 2.
- After all 9 subjects are enrolled and treated in Cohort 2 and the last subject has been treated and monitored for 24 hours, if 1 or more of the 9 subjects experience a serious adverse reaction, or if 2 or more of the 9 subjects experience a Grade ≥ 3 treatment-related AE, the IDMC will review available data to make a recommendation on starting Cohort 3.

Due to the recruitment challenge of low hospitalisation rates in the limited number of sites participating in Part 1 of this study, the IDMC will be asked to make a recommendation to progress to Part 2 of this study (with or without modifications to the study design and protocol), based on the interim analysis of the Phase 1a study in healthy subjects, supplemented with the available data from Part 1 of this study, even if not all Part 1 cohorts have reached the planned enrolment.

Further information is provided in the IDMC Charter.

10.1.6 Dissemination of Clinical Study Data

When the clinical study report is completed, the Sponsor will provide the major findings of the trial to the Investigator. A summary of the trial results will also be posted in a publicly accessible database (e.g., www.ClinTrials.gov). The results may also be submitted for publication.

10.1.7 Data Quality Assurance

- All subject data relating to the study will be recorded on electronic CRF unless transmitted to the Sponsor or designee electronically (e.g., laboratory data). The Investigator is responsible for verifying that data entries are accurate and correct by physically or electronically signing the CRF.
- The Investigator must maintain accurate documentation (source data) that supports the information entered in the CRF.
- The Investigator must permit study-related monitoring, audits, IRB/IEC review, and regulatory agency inspections and provide direct access to source data documents.
- Monitoring details describing strategies (e.g., risk-based initiatives in operations and quality such as Risk Management and Mitigation Strategies and Analytical Risk-Based Monitoring), methods, responsibilities, and requirements, including handling of noncompliance issues and monitoring techniques (central, remote, or on-site monitoring) are provided in the Monitoring Plan and Risk Management Plan.
- The Sponsor or designee is responsible for the data management of this study, including quality checking of the data.
- The Sponsor assumes accountability for actions delegated to other individuals (e.g., Contract Research Organisations).
- Study monitors will perform ongoing source data verification to confirm that data entered into the CRF by authorised site personnel are accurate, complete, and verifiable from source documents; that the safety and rights of subjects are being protected; and that the study is being conducted in accordance with the currently approved protocol and any other study agreements, ICH GCP, and all applicable regulatory requirements.
- Records and documents, including signed ICF, pertaining to the conduct of this study must be retained by the Investigator for at least 2 years after the last approval of a marketing application or 15 years from completion of the study (whichever is longer), unless local regulations or institutional policies require a longer retention period. No records may be destroyed during the retention period without the written approval of the Sponsor. No records may be transferred to another location or party without written notification to the Sponsor.
- All data generated by the site personnel will be captured electronically at each study site using eCRFs. Data from external sources (such as laboratory data) will be imported into the database. Once the eCRF clinical data have been submitted to the central server at the independent data centre, corrections to the data fields will be captured in an audit trail. The reason for change, the name of the person who performed the change, together with the time and date will be logged to provide an audit trail.

- If additional corrections are needed, the responsible monitor or data manager will raise a query in the EDC application. The appropriate staff at the study site will answer queries sent to the Investigator. The name of the staff member responding to the query, and time and date stamp will be captured to provide an audit trail. Once all source data verification is complete, and all queries are closed, the monitor will freeze the eCRF page.
- The specific procedures to be used for data entry and query resolution using the EDC system/eCRF will be provided to study sites in a training manual. In addition, site personnel will receive training on the EDC system/eCRF.

10.1.8 Source Documents

Source documents provide evidence for the existence of the subject and substantiate the integrity of the data collected. Source documents are filed at the Investigator's site.

Data reported on the CRF or entered in the eCRF that are transcribed from source documents must be consistent with the source documents, or the discrepancies must be explained. The Investigator may need to request previous medical records or transfer records, depending on the study. Also, current medical records must be available.

10.1.9 Study and Site Start and Closure

The study start date is the date on which the clinical study will be open for the recruitment of subjects.

The first act of recruitment is the first subject signing consent.

The Sponsor designee reserves the right to close the study site or terminate the study at any time for any reason at the sole discretion of the Sponsor. Study sites will be closed upon study completion. A study site is considered closed when all required documents and study supplies have been collected and a study site closure visit has been performed.

The Investigator may initiate study site closure at any time, provided there is reasonable cause and sufficient notice is given in advance of the intended termination.

Reasons for the early closure of a study site by the Sponsor or Investigator may include but are not limited to:

- Failure of the Investigator to comply with the protocol, the requirements of the IRB/IEC or local health authorities, the Sponsor's procedures, or GCP guidelines
- Inadequate recruitment of subjects by the Investigator

- Discontinuation of further study treatment development

If the study is prematurely terminated or suspended, the Sponsor shall promptly inform the Investigators, the IECs/IRBs, the regulatory authorities, and any contract research organisation(s) (CRO) used in the study of the reason for termination or suspension, as specified by the applicable regulatory requirements. The Investigator shall promptly inform the subject and should assure appropriate subject therapy and/or follow-up.

10.1.10 Publication Policy

- The results of this study will be published by the Sponsor in collaboration with Investigators.
- Investigators may publish or present other subject data or case reports at scientific meetings. If this is foreseen, the Investigator agrees to submit all manuscripts or abstracts to the Sponsor before submission. This allows the Sponsor to protect proprietary information and to provide comments.
- The Sponsor will comply with the requirements for publication of study results. In accordance with standard editorial and ethical practice, the Sponsor will generally support publication of multicentre studies only in their entirety and not as individual site data. In this case, a coordinating Investigator will be designated by mutual agreement.
- Authorship will be determined by mutual agreement and in line with International Committee of Medical Journal Editors authorship requirements.

10.1.11 Protocol Approval and Amendment

Before the start of the study, the study protocol and/or other relevant documents will be approved by the IEC/IRB/Competent Authorities, in accordance with local legal requirements. The Sponsor must ensure that all ethical and legal requirements have been met before the first subject is enrolled in the study.

This protocol is to be followed exactly. To alter the protocol, amendments must be written, receive approval from the appropriate personnel, and receive IRB/IEC/Competent Authority approval prior to implementation (if appropriate). In the US: Following approval, the protocol amendment(s) will be submitted to the IND under which the study is being conducted.

Administrative changes (not affecting the subject benefit/risk ratio) may be made without the need for a formal amendment. All amendments will be distributed to all protocol recipients, with appropriate instructions.

10.1.12 Liability and Insurance

The Sponsor will take out reasonable third-party liability insurance cover in accordance with all legal requirements. The civil liability of the Investigator, the persons instructed by him or her and the hospital, practice, or institute in which they are employed, and the liability of the Sponsor with respect to financial loss due to personal injury and other damage that may arise as a result of the carrying out of this study are governed by the applicable law.

The Sponsor will arrange for subjects participating in this study to be insured against financial loss due to personal injury caused by the pharmaceutical products being tested or by medical steps taken in the course of the study.

10.1.12.1 Access to Source Data

During the study, a monitor will make site visits to review protocol compliance, compare eCRF entries and individual subject's medical records, assess drug accountability, and ensure that the study is being conducted according to pertinent regulatory requirements. The eCRF entries will be verified with source documentation. The review of medical records will be performed in a manner to ensure that subject confidentiality is maintained.

Checking of the eCRF entries for completeness and clarity, and cross-checking with source documents, will be required to monitor the progress of the study. Moreover, regulatory authorities of certain countries, IRBs, IECs, and/or the Sponsor's Clinical Quality Assurance Group may wish to carry out such source data checks and/or on-site audit inspections. Direct access to source data will be required for these inspections and audits; they will be carried out giving due consideration to data protection and medical confidentiality. The Investigator assures Parexel and the Sponsor of the necessary support at all times.

10.2 Appendix 2: 8-Point Ordinal Scale

The worst clinical status (i.e., the highest ordinal scale) of the day will be recorded.

Score	Description
1	Not hospitalised, no limitations of activities
2	Not hospitalised, limitation of activities, home oxygen requirement, or both
3	Hospitalised, not requiring supplemental oxygen and no longer requiring ongoing medical care (used if hospitalisation was extended for infection-control reasons)
4	Hospitalised, not requiring supplemental oxygen but requiring ongoing medical care (SARS-CoV-2 – related or other medical conditions)
5	Hospitalised, requiring any supplemental oxygen
6	Hospitalised, requiring non-invasive ventilation or use of high-flow oxygen devices
7	Hospitalised, receiving invasive mechanical ventilation or ECMO
8	Death

10.3 Appendix 3: Assessment of 14 Common COVID-19-Related Symptoms: Items and Response Options

**Items**<br>For items 1–10, sample item wording could be: "What was the severity of your [insert symptom] at its worst over the last 24 hours?"	**Response options and scoring** [a]
1. Stuffy or runny nose	None = 0<br>Mild = 1<br>Moderate = 2<br>Severe = 3
2. Sore throat	
3. Shortness of breath (difficulty breathing)	
4. Cough	
5. Low energy or tiredness	
6. Muscle or body aches	
7. Headache	
8. Chills or shivering	
9. Feeling hot or feverish	
10. Nausea (feeling like you wanted to throw up)	
11. How many times did you vomit (throw up) in the last 24 hours? [b]	I did not vomit at all = 0<br>1–2 times = 1<br>3–4 times = 2<br>5 or more times = 3
12. How many times did you have diarrhoea (loose or watery stools) in the last 24 hours? [b]	I did not have diarrhoea at all = 0<br>1–2 times = 1<br>3–4 times = 2<br>5 or more times = 3
13. Rate your sense of smell in the last 24 hours	My sense of smell is THE SAME AS usual = 0<br>My sense of smell is LESS THAN usual = 1<br>I have NO sense of smell = 2
14. Rate your sense of taste in the last 24 hours	My sense of taste is THE SAME AS usual = 0<br>My sense of taste is LESS THAN usual = 1<br>I have NO sense of taste = 2

Note: Score values are included in the table for ease of reference; the score values will not be presented within the response options presented to trial patients to avoid confusing patients.

The response options shown for items 11 and 12 are intended only for use with a 24-hour recall period.

Source: FDA Guidance for Industry 2020

10.4 Appendix 4: Adverse Events: Definitions and Procedures for Recording, Evaluating, Follow-up, and Reporting

10.4.1 Definition of AE

AE Definition
• An AE is any untoward medical occurrence in a patient or clinical study subject, temporally associated with the use of a study treatment, whether or not considered related to the study treatment.<br>• NOTE: An AE can therefore be any unfavourable and unintended sign (including an abnormal laboratory finding), symptom, or disease (new or exacerbated) temporally associated with the use of a study treatment.

Events Meeting the AE Definition
• Any abnormal laboratory test results (haematology, clinical chemistry, or urinalysis) or other safety assessments (e.g., ECG, radiological scans, vital signs measurements), including those that worsen from baseline, considered clinically significant in the medical and scientific judgement of the Investigator (e.g., not related to progression of underlying disease).<br>• Exacerbation of a chronic or intermittent pre-existing condition including either an increase in frequency and/or intensity of the condition.<br>• New conditions detected or diagnosed after study treatment administration even though it may have been present before the start of the study.<br>• Signs, symptoms, or the clinical sequelae of a suspected drug-drug interaction.<br>• Signs, symptoms, or the clinical sequelae of a suspected overdose of either study treatment or a concomitant medication. Overdose per se will not be reported as an AE/SAE unless it is an intentional overdose taken with possible suicidal/self-harming intent. Such overdoses should be reported regardless of sequelae.<br>• "Lack of efficacy" or "failure of expected pharmacological action" per se will not be reported as an AE or SAE. Such instances will be captured in the efficacy assessments. However, the signs, symptoms, and/or clinical sequelae resulting from lack of efficacy will be reported as AE or SAE if they fulfil the definition of an AE or SAE.

**Events NOT Meeting the AE Definition**
• Any clinically significant abnormal laboratory findings or other abnormal safety assessments which are associated with the underlying disease, unless judged by the Investigator to be more severe than expected for the subject's condition.<br>• The disease/disorder being studied or expected progression, signs, or symptoms of the disease/disorder being studied, unless more severe than expected for the subject's condition.<br>• Medical or surgical procedure (e.g., endoscopy, appendectomy): the condition that leads to the procedure is the AE.<br>• Situations in which an untoward medical occurrence did not occur (social and/or convenience admission to a hospital).<br>• Anticipated day-to-day fluctuations of pre-existing disease(s) or condition(s) present or detected at the start of the study that do not worsen.

10.4.2 Definition of SAE

If an event is not an AE per definition above, then it cannot be an SAE even if serious conditions are met (e.g., hospitalisation for signs/symptoms of the disease under study, death due to progression of disease).

**An SAE is defined as any untoward medical occurrence that, at any dose:**
**a. Results in death**
**b. Is life-threatening**<br>The term 'life-threatening' in the definition of 'serious' refers to an event in which the subject was at risk of death at the time of the event. It does not refer to an event, which hypothetically might have caused death, if it were more severe.

**c.**	**Requires hospitalisation or prolongation of existing hospitalisation**
•	In general, hospitalisation signifies that the subject has been detained (usually involving at least an overnight stay) at the hospital or emergency ward for observation and/or intervention that would not have been appropriate in the physician's office or outpatient setting. Complications that occur during hospitalisation are AE. If a complication prolongs hospitalisation or fulfils any other serious criteria, the event is serious. When in doubt as to whether "hospitalisation" occurred or was necessary, the AE should be considered serious.
•	Hospitalisation for elective intervention of a pre-existing condition that did not worsen from baseline is not considered an AE.
**d.**	**Results in persistent disability/incapacity**
•	The term disability means a *substantial disruption of a person's ability to conduct normal life functions.*
•	This definition is not intended to include experiences of relatively minor medical significance such as uncomplicated headache, nausea, vomiting, diarrhoea, influenza, and accidental trauma (*e.g., sprained ankle) which may interfere with or prevent everyday life functions but do not constitute a substantial disruption.*
**e.**	**Is a congenital anomaly/birth defect**
**f.**	**Other situations**
•	Medical or scientific judgement should be exercised in deciding *whether SAE reporting is appropriate in other situations such as important medical events that may not be immediately life-threatening or result in death or hospitalisation but may jeopardise the subject or may require medical or surgical intervention to prevent one of the other outcomes listed in the above definition. These events should usually be considered serious.*
•	Examples of such events include invasive or malignant cancers, intensive *treatment in an emergency room or at home for allergic bronchospasm, blood dyscrasias or convulsions that do not result in hospitalisation, or development of drug dependency or drug abuse.*

10.4.3 Recording and Follow-up of AE and SAE

AE and SAE Recording
• When an AE/SAE occurs, it is the responsibility of the Investigator to review all documentation (e.g., hospital progress notes, laboratory, and diagnostics reports) related to the event.<br>• The Investigator will then record all relevant AE/SAE information in the CRF.<br>• It is not acceptable for the Investigator to send photocopies of the subject's medical records to the Sponsor or CRO in lieu of completion of the AE/SAE CRF page.<br>• There may be instances when copies of medical records for certain cases are requested by the Medical Monitor or Pharmacovigilance Services. In this case, all subject identifiers, with the exception of the subject number, will be blinded on the copies of the medical records before submission to the Medical Monitor or Pharmacovigilance Services.<br>• The Investigator will attempt to establish a diagnosis of the event based on signs, symptoms, and/or other clinical information. In such cases, the diagnosis (not the individual signs/symptoms) will be documented as the AE/SAE.
**Assessment of Intensity**
The Investigator will make an assessment of intensity for each AE and SAE reported during the study according to National Cancer Institute Common Terminology Criteria for Adverse Events (NCI-CTCAE) version 5.0 and assign it to one of the following categories:<br>• Grade 1: Mild; asymptomatic or mild symptoms; clinical or diagnostic observations only; intervention not indicated.<br>• Grade 2: Moderate; minimal, local or non-invasive intervention indicated; limiting age-appropriate instrumental activities of daily living (e.g., preparing meals, shopping for groceries or clothes, using the telephone, managing money).<br>• Grade 3: Severe or medically significant but not immediately life-threatening; hospitalisation or prolongation of hospitalisation indicated; disabling; limiting self-care activities of daily living (e.g., bathing, dressing and undressing, feeding self, using the toilet, taking medications, and not bedridden).<br>• Grade 4: Life-threatening consequences; urgent intervention indicated.<br>• Grade 5: Death related to AE.

An event is defined as ‘serious’ when it meets at least one of the predefined outcomes as described in the definition of an SAE, NOT when it is rated as severe.

**Assessment of Causality**
• The Investigator is obligated to assess the relationship between study treatment and each occurrence of each AE/SAE.<br>• A "reasonable possibility" of a relationship conveys that there are facts, evidence, and/or arguments to suggest a causal relationship, rather than a relationship cannot be ruled out.<br>• The Investigator will use clinical judgement to determine the relationship.<br>• Alternative causes, such as underlying disease(s), concomitant therapy, and other risk factors, as well as the temporal relationship of the event to study treatment administration will be considered and investigated.<br>• The Investigator will also consult the Investigator’s Brochure and/or Product Information, for marketed products, in his/her assessment.<br>• For each AE/SAE, the Investigator **must** document in the medical notes that he/she has reviewed the AE/SAE and has provided an assessment of causality.<br>• There may be situations in which an SAE has occurred, and the Investigator has minimal information to include in the initial report to Pharmacovigilance Services. However, **it is very important that the Investigator always make an assessment of causality for every event before the initial transmission of the SAE data to Pharmacovigilance Services.**<br>• The Investigator may change his/her opinion of causality in light of follow-up information and send an SAE follow-up report with the updated causality assessment.<br>• The causality assessment is one of the criteria used when determining regulatory reporting requirements.

Follow-up of AE and SAE
• The Investigator is obligated to perform or arrange for the conduct of supplemental measurements and/or evaluations as medically indicated or as requested by the Medical Monitor to elucidate the nature and/or causality of the AE or SAE as fully as possible. This may include additional laboratory tests or investigations, histopathological examinations, consultation with other health care professionals, or post-mortem findings if applicable/available.<br>• New or updated information will be recorded in the originally completed CRF.<br>• The Investigator will submit any updated SAE data to the Sponsor within 24 hours of receipt of the information.

10.4.1 Reporting of SAE

SAE Reporting to Parexel Pharmacovigilance Services via Electronic Data Collection Tool
The Investigator must report any SAEs to the Parexel Clinical Studies Safety Centre within 24 hours of becoming aware of the event.<br>• The primary mechanism for reporting an SAE to Parexel Pharmacovigilance Services will be the EDC tool.<br>• If the electronic system is unavailable, then the site will use the paper SAE data collection tool (see next section) in order to report the event within 24 hours.<br>• The site will enter the SAE data into the electronic system as soon as it becomes available.<br>• The Investigator and the Sponsor (or Sponsor's designated agent) will review each SAE report and the Sponsor/Parexel will evaluate the seriousness and the causal relationship of the event to study treatment. In addition, the Sponsor (or Sponsor's designated agent) will evaluate the expectedness according to the reference document (Investigator Brochure or Summary of Product Characteristics). Based on the Investigator and Sponsor's assessment of the event, a decision will be made concerning the need for further action.<br>• After the study is completed at a given site, the EDC tool will be taken off-line to prevent the entry of new data or changes to existing data.

- If a site receives a report of a new SAE from a study subject or receives updated data on a previously reported SAE after the EDC tool has been taken off-line, then the site can report this information on a paper SAE form (see next section) or to the Sponsor by email.
- Specific phone numbers will be provided for each country involved in the study.
- Contacts for SAE reporting can be found in the table below.

**Suspected Unexpected Serious Adverse Reactions (SUSARs)**
Any AE that is serious, associated with the use of the study treatment, and unexpected (SUSAR) has additional reporting requirements, as described below.<br>• If the SUSAR is fatal or life-threatening, associated with study treatment, and unexpected, regulatory authorities and IECs will be notified within 7 calendar days after the Sponsor learns of the event. Additional follow-up (cause of death, autopsy report, and hospital report) information should be reported within an additional 8 days (15 days total).<br>• If the SUSAR is not fatal or life-threatening but is otherwise serious, associated with study treatment, and unexpected, regulatory authorities and IECs will be notified within 15 calendar days after the Sponsor learns of the event.<br>The Sponsor will notify the Investigators in a timely fashion of relevant information about SUSARs that could adversely affect the safety of subjects. Follow-up information may be submitted if necessary.<br>The Sponsor will also provide annual safety updates to the regulatory authorities and IECs responsible for the study. These updates will include information on SUSARs and other relevant safety findings.

Reporting Serious Adverse Events via Paper Form (Back-up Method)

The minimum information required for an initial report is:

- Name of person sending the report (e.g., name, address of Investigator);
- Subject identification (screening/randomisation number, initials, NOT subject name);
- Protocol number;

- Description of SAE;
- Causality assessment, if possible.

However, as far as possible all points on the SAE form should be covered in the initial report, or the completed SAE form itself must be emailed to the Parexel Safety mailbox. In addition, the event must be documented in the eCRF.

After receipt of the initial report, the safety centre will review the information and, if necessary, contact the Investigator, to obtain further information for assessment of the event. Parexel will be responsible for all information processing and reporting according to local legal requirements. Where necessary, Investigators will inform regulatory authorities and/or IEC/IRBs in their own countries.

Parexel International
SAE email: 255435_Safety@Parexel.com
SAE answering service: +33 1 44 90 32 90
Email: drugsafety@Parexel.com

Medical Monitor: 255435_MM@Parexel.com

10.5 Appendix 5: Contraceptive Guidance and Collection of Pregnancy Information

10.5.1 Definitions

Woman of Childbearing Potential (WOCBP)

A woman is considered fertile following menarche and until becoming post-menopausal unless permanently sterile (see below).

If fertility is unclear (e.g., amenorrhea in adolescents or athletes) and a menstrual cycle cannot be confirmed before first dose of study treatment, additional evaluation should be considered.

Women in the following categories are not considered WOCBP:

1. Premenarchal
2. Premenopausal female with one of the following:
   - Documented hysterectomy
   - Documented bilateral salpingectomy
   - Documented bilateral oophorectomy

NOTE: Documentation can come from the site personnel's review of the subject's medical records, medical examination, or medical history interview.

3. Post-menopausal female
   - A post-menopausal state is defined as no menses for 12 months without an alternative medical cause.
     - A high follicle stimulating hormone (FSH) level in the post-menopausal range may be used to confirm a post-menopausal state in women not using hormonal contraception or hormonal replacement therapy (HRT). However, in the absence of 12 months of amenorrhea, a single FSH measurement is required.
   - Females on HRT and whose menopausal status is in doubt will be required to use one of the non-oestrogen hormonal highly effective contraception methods if they wish to continue their HRT during the study. Otherwise, they must discontinue HRT to allow confirmation of post-menopausal status before study enrolment.

10.5.2 Contraception Guidance

Male Subjects

Male subjects with female partners of childbearing potential are eligible to participate if they agree to ONE of the following during the treatment period and for at least 90 days after the last dose:

- Are abstinent from penile-vaginal intercourse as their usual and preferred lifestyle (abstinent on a long-term and persistent basis) and agree to remain abstinent.
- Agree to use a condom and consider contraception for non-pregnant when having penile-vaginal intercourse.

Male subjects with a pregnant or breastfeeding partner must agree to remain abstinent from penile-vaginal intercourse or use a male condom during each episode of penile penetration during the treatment period and for at least 90 days after the last dose.

No sperm donation is allowed from Day 1 to at least 90 days after receiving XVR011

Female Subjects

Female subjects of childbearing potential are eligible to participate if they agree to use a highly effective method of contraception as described in the table below, consistently and correctly during the treatment period and for at least 90 days after the last dose.

No egg donation is allowed from Day 1 to at least 90 days after receiving XVR011

**Highly Effective Contraceptive Methods That Are User Dependent**[1]<br>*Failure rate of <1% per year when used consistently and correctly.*
Combined (oestrogen- and progestin-containing) hormonal contraception associated with inhibition of ovulation<br>• Oral<br>• Intravaginal<br>• Transdermal
Progestogen-only hormonal contraception associated with inhibition of ovulation<br>• Oral<br>• Injectable

**Highly Effective Contraceptive Methods That Are User Independent**[1]
Implantable progestogen-only hormonal contraception associated with inhibition of ovulation<br>• Intrauterine device (IUD)<br>• Intrauterine hormone-releasing system (IUS)<br>• Bilateral tubal occlusion
**Vasectomised Partner**<br>A vasectomised partner is a highly effective contraception method provided that the partner is the sole male sexual partner of the WOCBP and the absence of sperm has been confirmed. If not, an additional highly effective method of contraception should be used.
**Sexual Abstinence**<br>Sexual abstinence is considered a highly effective method only if defined as refraining from heterosexual intercourse during the entire period of risk associated with the study treatment. The reliability of sexual abstinence needs to be evaluated in relation to the duration of the study and the preferred and usual lifestyle of the subject.

NOTES:

1 Typical use failure rates may differ from those when used consistently and correctly. Use should be consistent with local regulations regarding the use of contraceptive methods for subjects participating in clinical studies.

10.5.3 Pregnancy Testing

- WOCBP should only be included after a confirmed menstrual period and a negative highly sensitive urine pregnancy test or serum pregnancy test at screening (Day -1).
- Pregnancy testing should also be performed at the EOS (ie 28 days after the dose of study treatment), or at the time of ED, and as required locally.
- Pregnancy testing will also be performed whenever a menstrual cycle is missed or when pregnancy is otherwise suspected.

10.5.4 Collection of Pregnancy Information

Male Subjects With Partners Who Become Pregnant

- The Investigator will attempt to collect pregnancy information on any male subject’s female partner who becomes pregnant while the male subject is in this study. This applies only to male subjects who receive XVR011.
- After obtaining the necessary signed informed consent from the pregnant female partner directly, the Investigator will record pregnancy information on the appropriate form and submit it to the Sponsor within 24 hours of learning of the partner’s pregnancy. The female partner will also be followed to determine the outcome of the pregnancy. Information on the status of

the mother and child will be forwarded to the Sponsor. Generally, the follow-up will be no longer than 6 to 8 weeks following the estimated delivery date. Any termination of the pregnancy will be reported regardless of foetal status (presence or absence of anomalies) or indication for the procedure.

Female Subjects Who Become Pregnant

- The Investigator will collect pregnancy information on any female subject who becomes pregnant while participating in this study. Information will be recorded on the appropriate form and submitted to the Sponsor within 24 hours of learning of a subject's pregnancy. The subject will be followed to determine the outcome of the pregnancy. The Investigator will collect any follow-up information on the subject and the neonate and the information will be forwarded to the Sponsor. Generally, follow-up will not be required for longer than 6 to 8 weeks beyond the estimated delivery date. Any termination of the pregnancy will be reported, regardless of foetal state (presence or absence of anomalies) or indication for the procedure.
- While pregnancy itself is not considered to be an AE or SAE, any pregnancy complication or elective termination of a pregnancy will be reported as an AE or SAE. A spontaneous abortion is always considered to be an SAE and will be reported as such. Any post-study pregnancy-related SAE considered reasonably related to study treatment by the Investigator will be reported to the Sponsor as described in Section 8.3.4. While the Investigator is not obligated to actively seek this information in former study subjects, he or she may learn of an SAE through spontaneous reporting.

10.6 Appendix 6: Abbreviations and Trademarks

Abbreviation Term	Definitions
ACE2	angiotensin-converting enzyme 2
ADA	anti-drug antibody
AE	adverse event
ALT	alanine aminotransferase
ARDS	acute respiratory distress syndrome
AST	aspartate aminotransferase
BDRM	Blinded Data Review Meeting
BMI	body mass index
CBER	Centre for Biologics Evaluation and Research
CHMP	Committee for Medicinal Products for Human Use
CI	Confidence interval
CIOMS	Council for International Organisations of Medical Sciences
CONSORT	Consolidated Standards of Reporting Trials
COVID-19	coronavirus disease 2019
CRF	case report form(s) (paper or electronic as appropriate for this study)
CRO	contract research organisation
CRP	C-reactive protein
DMC	Data Monitoring Committee
ECG	Electrocardiogram
ED	early discontinuation
EDC	electronic data capture
EMA	European Medicines Agency
EOS	End of study
EU	European Union
EudraCT	European Clinical Trials Database
FAS	full analysis set
FDA	Food and Drug Administration
FSH	follicle stimulating hormone
FU	Follow-up
GCP	Good Clinical Practice
HIPAA	Health Insurance Portability and Accountability Act
HIV	human immunodeficiency virus

HRT	hormone replacement therapy
IB	Investigator's Brochure
ICF	informed consent form
ICH	International Conference on Harmonisation of Technical Requirements for Pharmaceuticals for Human Use
ICU	intensive care unit
IDMC	Independent Data Monitoring Committee
IDSA	Infectious Diseases Society of America
IEC	Independent Ethics Committee
IgG	immunoglobulin G
IgG1	immunoglobulin G subclass 1
IgG1 Fc	crystallizable fragment of the immunoglobulin class G
IMP	investigational medicinal product
IND	Investigational New Drug
IRB	Institutional Review Board
IRR	infusion related reaction
IRT	interactive response technology
LC-MS	liquid chromatography-mass spectrometry
LDH	lactate dehydrogenase
MedDRA	Medical Dictionary for Regulatory Activities
MS	mass spectrometry
nAb	neutralising anti-drug antibody
PD	pharmacodynamic(s)
PK	pharmacokinetic(s)
PPS	per protocol analysis set
RNA	ribonucleic acid
PT	preferred term
RT-qPCR	reverse transcriptase quantitative polymerase chain reaction
RT-PCR	reverse transcriptase polymerase chain reaction
SAE	serious adverse event
SAP	statistical analysis plan
SARS	severe acute respiratory syndrome
SARS-CoV-2	severe acute respiratory syndrome coronavirus 2
SoA	schedule of activities
SoC	standard of care
SOC	system organ class

SUSAR	suspected unexpected serious adverse reaction
ULN	upper limit of normal
USA	United States of America
WHO	World Health Organisation
WOCBP	woman of childbearing potential

11 References

Beigel et al 2020

Beigel JH, Tomashek KM, Dodd LE, Mehta AK, Zingman BS, Kalil AC, et al. Remdesivir for the Treatment of Covid-19 - Final Report. N Engl J Med. 2020: NEJMoa2007764.

Bhimraj et al 2020

Bhimraj A, Morgan RL, Shumaker AH, Lavergne V, Baden L, Cheng VC, et al. Infectious Diseases Society of America Guidelines on the Treatment and Management of Patients with COVID-19. Version 3.3, 25 September 2020. Available at https://www.idsociety.org/COVID19guidelines.

Blot et al 2021

Blot M, et al. Alveolar SARS-CoV-2 Viral Load Is Tightly Correlated With Severity in COVID-19 ARDS. 2021 cid 2021:72 (1 May)

Cevik et al 2020a

Cevik M, Kuppalli K, Kindrachuk J, Peiris M. Virology, transmission, and pathogenesis of SARS-CoV-2. BMJ. 2020 Oct 23;371:m3862. doi: 10.1136/bmj.m3862. PMID: 33097561.

Cevik et al 2020b

Cevik M, Tate M, Lloyd O Maraolo AE, Schafers J, Ho A; SARS-CoV-2, SARS-CoV, and MERS-CoV viral load dynamics, duration of viral shedding, and infectiousness: a systematic review and meta-analysis; The Lancet (Microbe) published online 19 November 2020. DOI:https://doi.org/10.1016/S2666-5247(20)30172-5

Cevik et al 2021

Cevik M et al, SARS-CoV-2, SARS-CoV, and MERS-CoV viral load dynamics, duration of viral shedding, and infectiousness: a systematic review and meta-analysis. Lancet Microbe 2021; 2: e13–22

European Commission 2020

European Commission. Daily News 03 / 07 / 2020: European Commission authorises first treatment against COVID-19 [Internet]. Brussels: European Commission; 2020 [cited 22 July 2020]. Available from: https://ec.europa.eu/commission/presscorner/detail/en/mex_20_1266.

EMA 2020

European Medicines Agency (EMA). First COVID-19 treatment recommended for EU authorisation [Internet]. Amsterdam: EMA; 2020. Updated 25 June 2020. Available at: https://www.ema.europa.eu/en/news/first-covid-19-treatment-recommended-eu-authorisation.

EMEA/CHMP/SWP/28367/07

Committee for Medicinal Products for Human Use (CHMP). Guideline on strategies to identify and mitigate risks for first-in human clinical trials with investigational medicinal products. 2007. Ref. EMEA/CHMP/SWP/28367/07. Available at https://www.ema.europa.eu/en/documents/scientific-guideline/guideline-strategies-identify-mitigate-risks-first-human-clinical-trials-investigational-medicinal_en.pdf

FDA Guidance for Industry 2020

Food and Drug Administration (FDA) / Centre for Drug Evaluation and Research (CDER) / Centre for Biologics Evaluation and Research (CBER) Guidance for Industry. Assessing COVID-19-Related Symptoms in Outpatient Adult and Adolescent Subjects in Clinical Trials of Drugs and Biological Products for COVID-19 Prevention or Treatment. September 2020. Available at https://www.fda.gov/media/142143/download

FDA News Release 2020a

Food and Drug Administration (FDA) News Release. Coronavirus (COVID-19) Update: FDA Issues Emergency Use Authorization for Potential COVID-19 Treatment. 01 May 2020. Available at: https://www.fda.gov/news-events/press-announcements/coronavirus-covid-19-update-fda-issues-emergency-use-authorization-potential-covid-19-treatment

FDA News Release 2020b

Food and Drug Administration (FDA) News Release. Coronavirus (COVID-19) Update: FDA Warns of Newly Discovered Potential Drug Interaction That May Reduce Effectiveness of a COVID-19 Treatment Authorised for Emergency Use. Press announcements. 15 June 2020. Available at: https://www.fda.gov/news-events/press-announcements/coronavirus-covid-19-update-fda-warns-newly-discovered-potential-drug-interaction-may-reduce

FDA News Release 2020c

Food and Drug Administration (FDA) News Release. FDA Approves First Treatment for COVID-19. Available at: https://www.fda.gov/news-events/press-announcements/fda-approves-first-treatment-covid-19

Horby et al 2021

Horby PW et al 2021, Casirivimab and imdevimab in patients admitted to 3 hospital with COVID-19 (RECOVERY): a randomised, 4 controlled, open-label, platform trial. posted 16 June 2021 on medRxiv preprint doi: https://doi.org/10.1101/2021.06.15.21258542 and Supplementary Appendix

Ibrahim et al 2001

Ibrahim, J. G., Chen, M.-H., and Sinha, D. (2001). Bayesian Survival Analysis. New York: Springer-Verlag.

Investigator's Brochure

Investigator's Brochure, XVR011.

Jiang et al 2020

Jiang S, Hillyer C, Du L. Neutralising Antibodies against SARS-CoV-2 and Other Human Coronaviruses. Trends Immunol. 2020 May;41(5):355-359. doi: 10.1016/j.it.2020.03.007. Epub 2020 Apr 2. Erratum in: Trends Immunol. 2020 Apr 24: PMID: 32249063; PMCID: PMC7129017.

Lother et al 2020

Lother SA, Abassi M, Agostinis A, Bangdiwala AS, Cheng MP, Drobot G, et al. Post-exposure prophylaxis or pre-emptive therapy for severe acute respiratory syndrome coronavirus 2 (SARS-CoV-2): study protocol for a pragmatic randomised-controlled trial. Can J Anaesth. 2020;67(9):1201-1211.

Marovich et al 2020

Marovich M, Mascola JR, Cohen MS. Monoclonal antibodies for prevention and treatment of COVID-19.

JAMA. 2020.

NIH COVID-19 Treatment Guidelines Panel 2020

National Institutes of Health (NIH) COVID-19 Treatment Guidelines Panel. Coronavirus Disease 2019 (COVID-19) Treatment Guidelines. Updated on 09 October 2020. Available at https://www.covid19treatmentguidelines.nih.gov/.

Osuchowski et al 2021

Osuchowski MF et al 2021, The COVID-19 puzzle: deciphering pathophysiology and phenotypes of a new disease entity. Lancet Respir Med 2021; 9: 622–42

Park et al 2018

Park SY, Lee JS, Son JS, Ko JH, Peck KR, Jung Y, et al. Post-exposure prophylaxis for Middle East respiratory syndrome in healthcare workers. J Hosp Infect. 2019;101(1):42-46.

Wang et al 2020

Wang L,Wang Y, Ye D, Liu Q. (2020). Review of the 2019 novel coronavirus (SARS-CoV-2) based on current evidence. Int J Antimicrob Agents, 55(6):105948, https://doi.org/10.1016/j.ijantimicag.2020.105948

WHO REACT Working Group et al 2020

WHO Rapid Evidence Appraisal for COVID-19 Therapies (REACT) Working Group, Sterne JAC, Murthy S, Diaz JV, Slutsky AS, Villar J, et al. Association Between Administration of Systemic Corticosteroids and Mortality Among Critically Ill Patients With COVID-19: A Meta-analysis. JAMA. 2020;324(13):1–13.

WHO/2019-nCoV/Corticosteroids/2020.1

World Health Organization (WHO). Corticosteroids for COVID-19. Living guidance. 2020. Updated 2 September 2020. Available at: https://apps.who.int/iris/bitstream/handle/10665/334125/WHO-2019-nCoV-Corticosteroids-2020.1-eng.pdf

WHO/2019-nCoV/clinical/2020.5

World Health Organisation. Clinical management of COVID-19. Interim guidance. Ref. WHO/2019-nCoV/clinical/2020.5 Last updated 27 May 2020. Available at https://www.who.int/publications/i/item/clinical-management-of-covid-19

WHO/Coronavirus disease Q&A/2020.11

World Health Organisation. Coronavirus disease (COVID-19) Q&As. Last updated 10 November 2020. Available at: https://www.who.int/emergencies/diseases/novel-coronavirus-2019/question-and-answers-hub/q-a-detail/coronavirus-disease-covid-19

WHO Living Guideline: Therapeutics and COVID-19, 20 November 2020

World Health Organisation. Therapeutics and COVID-19: living guideline, 20 November 2020. Available at: https://apps.who.int/iris/handle/10665/336729

Wiersinga et al 2020

Wiersinga WJ, Rhodes A, Cheng AC, Peacock SJ, Prescott HC. Pathophysiology, Transmission, Diagnosis, and Treatment of Coronavirus Disease 2019 (COVID-19): A Review. JAMA. 2020;324(8):782-93.

Wu et al 2020

Wu C, Liu Y, Yang Y, Zhang P, Zhong W, Wang Y, Wang Q, Xu Y, Li M, Li X, Zheng M, Chen L, Li H. Analysis of therapeutic targets for SARS-CoV-2 and discovery of potential drugs by computational methods. Acta Pharm Sin B. 2020 May;10(5):766-788. doi: 10.1016/j.apsb.2020.02.008. Epub 2020 Feb 27. PMID: 32292689; PMCID: PMC7102550.

Investigator Agreement Page

Declaration of the Principal or Global Coordinating Investigator

Title: A 2-part Clinical Study Including a First-in-Human, Open-label, Single Ascending Dose Part (Phase 1) Followed by a Randomised, Double-blind, Placebo-controlled Part (Phase 2) to Evaluate the Efficacy and Safety of XVR011 in Patients Hospitalised for Mild to Moderate COVID-19

This study protocol was subjected to critical review and has been approved by the Sponsor. The information it contains is consistent with the current risk/benefit evaluation of the investigational product as well as with the moral, ethical, and scientific principles governing clinical research as set out in the Declaration of Helsinki and the guidelines on Good Clinical Practice.

Principal or Global Coordinating Investigator

____________________________________ ______________________
Signature Date

____
Name (block letters)

____
Title (block letters)

____
Institution (block letters)

____
Phone number

Declaration of the National Coordinating Investigator

Title: A 2-part Clinical Study Including a First-in-Human, Open-label, Single Ascending Dose Part (Phase 1) Followed by a Randomised, Double-blind, Placebo-controlled Part (Phase 2) to Evaluate the Efficacy and Safety of XVR011 in Patients Hospitalised for Mild to Moderate COVID-19

This study protocol was subjected to critical review and has been approved by the Sponsor. The information it contains is consistent with the current risk/benefit evaluation of the investigational product as well as with the moral, ethical, and scientific principles governing clinical research as set out in the Declaration of Helsinki, and the guidelines on Good Clinical Practice.

National Coordinating Investigator

____________________________________ ______________________

Signature Date

____

Name (block letters)

____

Title (block letters)

____

Institution (block letters)

____

Phone number

Declaration of the Investigator

Title: A 2-part Clinical Study Including a First-in-Human, Open-label, Single Ascending Dose Part (Phase 1) Followed by a Randomised, Double-blind, Placebo-controlled Part (Phase 2) to Evaluate the Efficacy and Safety of XVR011 in Patients Hospitalised for Mild to Moderate COVID-19

All documentation for this study that is supplied to me and that has not been previously published will be kept in the strictest confidence. This documentation includes this study protocol, Investigator's Brochure, electronic CRF (eCRF), and other scientific data.

The study will not be commenced without the prior written approval of a properly constituted IRB or IEC. No changes will be made to the study protocol without the prior written approval of the Sponsor and the IRB or IEC, except where necessary to eliminate an immediate hazard to the subjects.

I have read and understood and agree to abide by all the conditions and instructions contained in this protocol.

Responsible Investigator of the Local Study Site

____________________________________ ______________________

Signature Date

____

Name (block letters)

____

Title (block letters)

____

Institution (block letters)

____

Phone number
