# Supplementary material for: Characterization of the VHH-Fc construct rimteravimab in healthy adults and patients hospitalized for mild-to-moderate COVID-19: Two Phase 1 randomized clinical trials
Source: PLoS Med. 2026 May 29;23(5):e1004609. doi: 10.1371/journal.pmed.1004609 (PMC13221044; doi:10.1371/journal.pmed.1004609)
Supplement: Attachment — Submitted filename: PMEDICINE-D-25-00069R1_AnswersToTheReviewers.pdf [file pmed.1004609.s006.pdf]

PMEDICINE-D-25-00069R1
Answers to the Reviewers' Comments

Dear Alison, Dear PLOS Medicine Editorial Board,

On behalf of my co-authors, I would like to thank you and the reviewers for all your insightful comments to improve the quality of our manuscript. Please find our answers below in blue, as well as in the Revised manuscript (version with track changes and 'cleaned' version).

Best wishes,
Florence Herschke, PhD, on behalf of all authors

Please ensure that the trial reporting follows CONSORT 2025 reporting and that claims are tempered and acknowledge the small study size and inherent limitations.

Answer: Thank you for your comment. We have made sure that the trial reporting follows the CONSORT 2025 reporting, and that claims were tempered, acknowledging the small study size and inherent limitations.

Please note: we ask that you clarify the delay in reporting the study findings.

Answer: Thank you for your comment. While it became clear that XVR011 lost its efficacy against Omicron BA.2, Exevir focused all its attention to diligently identify a novel optimized candidate which would neutralize all SARS-CoV-2 variants. Being a startup, there was unfortunately no bandwidth to publish the results of the clinical trials earlier. However, we feel that the results are still relevant for the VHH-Fc community to date, hence our proposed current publication.

Comments from the academic editor:

The weaknesses of the study are mostly covered by the other reviewer's comments, including the small sample size of the Phase 1 a study, which limits conclusions on rare safety signals and efficacy. The Phase 1b was open-label, had no placebo group, was also underpowered, and conducted amid shifting variants and standards of care. Reported "dose-response trends" must be interpreted cautiously. XVR011 lost activity against Omicron BA.2, undermining long-term therapeutic relevance.

Although presented as clinically irrelevant, the relatively high ADA incidence in patients (~30%) raises concern for broader use, especially since immunogenicity is unpredictable in diverse populations.

Answer: Thank you for your comment. No treatment-induced ADA were observed in healthy volunteers in the phase 1a study. In the phase 1b study, treatment-induced ADA were observed in ~30% of patients, however no difference on XVR011 PK, COVID-19 symptoms or AE was observed in comparison to patients where no ADA was detected. The data of this particular study suggest that the treatment-induced ADA are clinically non-relevant. We agree that the studies are of small size and cannot be generalized without further investigations, where immunogenicity should be carefully monitored. This limitation was highlighted in the Discussion as follows: 'The studies presented here have limitations. On one hand, the anticipated small number of participants enrolled, short follow-up due to the early phase 1b study termination - linked to the emergence of Omicron strains and absence of repeated dosing may impact interpretation of safety, PK, PD and immunogenicity signals.'

Some of the authors are employees or contractors of ExeVir Bio, the sponsor — there may be some bias in interpretation.

Answer: Thank you for your comment. Authors' affiliations, and potential conflicts of interest, were disclosed as per PLOS Medicine's guidelines in the original version of the manuscript.

The manuscript is long and somewhat repetitive, with extensive technical detail that might be better placed in supplementary material.

Answer: Thank you for your thoughtful suggestion. In the Methods section, parts of the 'Study design', 'Immunogenicity assessment' and 'Statistical analysis' subsections were moved to the Supplementary information.

Comments from the editors:

* Please revise your title so that it complies with to PLOS Medicine's style. Your title must be nondeclarative and not a question. It should begin with main concept if possible. "Effect of" should be used only if causality can be inferred, i.e., for an RCT. Please place the study design ("A randomized controlled trial," "A retrospective study," "A modelling study," etc.) in the subtitle (ie, after a colon). Please remove the first in human from the title.

Answer: Thank you for your comment. XVR011 was never dosed in humans prior to the two trials described here. Therefore, they do correspond first-in-human studies. The title was thus adapted as follows:
'*First-in-Human characterization of the VHH-Fc construct XVR011 (rimteravimab),* ~~*the first VHH-Fc construct tested in randomized clinical trials*~~ *in healthy adults and patients hospitalized for mild-to-moderate COVID-19: two phase 1*~~*a/b*~~ *randomized clinical trials*'

* Please review your text for claims of novelty or primacy (e.g. 'for the first time') and remove this language. In addition, please check that any use of statistical terms (such as trend or significant) are supported by the data, and if not please remove them.

Answer: Thank you for your comment. To our knowledge, as per PubMed search, no other VHH-Fc construct has been dosed in healthy volunteers. The claims of novelty are added to help the audience understand the importance of the results for the VHH-Fc community. This is also acknowledged by reviewer 1.
The style suggested by reviewer 4 was adopted to avoid misinterpretation.

Please clarify why there are no authors from the Netherlands.

Answer: Thank you for your comment. Salah Hadi, the principal investigator for the phase 1a study, is an author from the Netherlands (exact affiliation: PRA Group BV, a PRA Health Sciences Company, Groningen, The Netherlands).

Please acknowledge the healthcare workers who made the trial possible.

Answer: Thank you for noting, the healthcare workers who made the trial possible have been acknowledged.

Please add the names of the ethics committee and regulatory boards that approved the trials and protocol (s).

Answer: Thank you for your comment. The names of the ethics committee and regulatory boards were described in the Methods section under 'Study ethics' and in Table S2.

Please clarify which boards approved any study amendments and ensure that the amendment(s) are detailed in the protocol, along with dates of amendments.

Answer: Thank you for your comment. The names of the ethics committees and regulatory boards which approved the study amendments were described in the Methods section under 'Study ethics'.
For the phase 1a study, only amendment 1 was submitted, the original version was not submitted. For the phase 1b study, we have added as supplementary information the rationale for the different amendments.

Please state in the manuscript the dates of first and last participant enrolment (first paragraph of the results section).

Answer: Thank you for your comment. The dates were provided in the Methods. They have now been placed in the first paragraph of the results section ('Subject Disposition and Demographics') as requested.

Identify the study sites in the Methods.

Answer: Thank you for your comment. The open study sites have been clarified in the Methods section, while the study sites which actually enrolled patients have been added to the Results section, under 'Subject disposition and demographics'.

Please specify the name of the data safety monitoring board or its members' location.

Answer: Thank you for your comment. For study EXEVIR0102, there was no formal IDMC established. For EXEVIR0101, there is no specific name of the data safety monitoring board. EXEVIR0101's IDMC consisted of 4 independent experts in the fields of infectious diseases, pulmonology/respiratory medicine and/or immunology. The Sponsor was responsible for establishing the IDMC and appointing its members. Identification of the IDMC members and their contact information were provided in Attachment #1 of the IDMC Charter. This information has been added to the Methods section in the Supplementary Information, under 'Study design'.

* Please confirm that your abstract complies with our requirements, including format. There are only three sections: Background, Methods and Findings, and Conclusion. Please provide all the information relevant to this study type https://journals.plos.org/plosmedicine/s/submission-guidelines#loc-abstract In particular, please ensure that your Abstract is CONSORTed according to the CONSORT 2025 guidelines and provide a CONSORT 2025 checklist.

Answer: Thank you for your comment. The abstract has been modified to follow PLOS Medicine guidelines mentioned above. The CONSORT checklist which was originally submitted was updated to follow the 2025 updated requirements.

* If all secondary outcomes described in the protocol are included in the manuscript, please briefly describe them in the Abstract. If only a subset is reported in this study, please clarify why in the Methods and do not report in the Abstract.

Answer: Thank you for your comment. Briefly, for part 1 of study EXEVIR0101, evaluating (a) the efficacy and (b) the antiviral activity of a single intravenous XVR011 infusion in subjects hospitalised for COVID-19) was a secondary outcome. In study EXEVIR0102, the sole secondary objective was to evaluate the pharmacokinetic (PK) profile of XVR011 after an IV infusion of single ascending doses in healthy subjects. All secondary outcomes described in the protocol are now reported in the Abstract.

The Background section of the Abstract must present information that explains the rationale for the study. Please do not begin by saying this is the first trial of a VHH-Fc in this population.

Answer: Thank you for your comment. This was, to our knowledge, the first clinical evaluation of a heavy chain-only VHH-Fc antibody construct in healthy volunteers and for an infectious disease in any study population. Prior to EXEVIR0101 and EXEVIR0102, only 3 VHH-Fc constructs had been tested in the clinic in cancer patients (in China and Russia), but this type of molecule had not yet been evaluated in healthy participants, nor had it been used against infectious diseases. We would like to stress this novelty because it is highly relevant for the antibody therapeutics field. The background of the abstract has been modified to reflect both this innovation, as well as the study rationale.

Please do not begin by saying this is the first trial of a VHH-Fc in this population.
Answer: Thank you for your comment. As explained above, this was to our knowledge, the first clinical evaluation of a VHH-Fc antibody construct for an infectious disease in any study population. It is this novelty and the very important relevance to both the antibody discovery, as well as the infectious diseases field, that motivated us to publish these Phase 1 studies, even though these studies were completed already several years ago. We believe this innovation, in particular the use of this type of molecule for treatment of a respiratory virus, requires its highlighting in both the abstract and manuscript.

Please correct the grammatical errors in the Abstract and use the active voice.

Answer: Thank you for your comment. Grammatical errors have been corrected and we have followed the same formatting as in this article recently published in *PLOS Medicine*: 'Psilocybin-assisted group

psychotherapy and mindfulness-based stress reduction for frontline healthcare provider COVID-19-related depression and burnout: A randomized controlled trial | PLOS Medicine'.

Please state SARS-CoV-2 before Omicron BA.2.

Answer: Thank you for your comment. Wherever Omicron BA.2 was present in the text, SARS-CoV-2 was added before.

Please remove discussion of trends in the Abstract.

Answer: Thank you for your comment. Discussion of trends has been removed in the Abstract.

Please avoid causal claims of causality in the Abstract where they may not be supported and temper your conclusions.

Answer: Thank you for your comment. Claims of causality have been removed in the Abstract and conclusions tempered.

The last sentence of the section Methods and Findings must state the technological limitations of the study.

Answer: Thank you for your comment. The following has been added at the end of the section Methods and Findings: '*The technological limitations of the studies presented here that may impact interpretation of safety, PK, PD and immunogenicity signals include the study size, antigenic shift from SARS-CoV-2 Delta to Omicron that occurred during the course of the Phase 1b study, leading to an early study termination and short follow-up, as well as the absence of repeated dosing. The absence of placebo in the phase 1b, changing standard of care for COVID-19 treatment over time and variable duration since last vaccination are factors potentially impacting the interpretation of the PD results'.*

Please add the clinical trial identifiers after the Abstract.

Answer: Thank you for your comment. Clinical trial identifiers have been added after the Abstract as requested.

Please clarify your statement in the Abstract to explain how it was determined that treatment emergent adverse events were not related to the treatment.

Answer: Thank you for your comment. The rules to assign treatment-emergent AE to study drug or another cause are detailed in the respective study protocols: Paragraph 8.2.1 for the phase 1a EXEVIR0102 trial and Annex 4 for the phase 1b EXEVIR0101 trial. Briefly, treatment-emergent AE were evaluated by the study Investigator to assign relatedness, taking into consideration: (i) the temporal relationship of the event to study treatment administration; (ii) the possible mechanism-of-action association with the study drug; and (iii) whether there may be a plausible explanation due to underlying disease or comorbidities or concomitant medications with the AE as a known side effect.
To limit the Abstract length, this information was added to the Methods section under 'Safety assessments', as follows: '*Assignment of treatment-emergent AE causality took into account: (i) the temporal relationship to XVR011 administration; (ii) the possible mechanism-of-action association with the study drug; and (iii) whether there may be a plausible explanation due to underlying disease or comorbidities or concomitant medications with the AE as a known side effect.*'.

Line 86: add "to our knowledge"

Answer: Thank you for your comment. '*to our knowledge*' has been added.

Line 124: please clarify why the effects of the LALA mutation would be of benefit.

Answer: Thank you for your comment. Potential benefits of the LALA mutation were present in the first version of the manuscript and they have been further clarified as follows: '*The human Fc is engineered with a LALA mutation to strongly reduce the binding to Fc-gamma receptors on immune cells and complement protein C1q, responsible for respectively triggering antibody effector functions like antibody-dependent cell-mediated cytotoxicity (ADCC) and complement-dependent cytotoxicity (CDC). By silencing these interactions, an optimal safety profile was envisioned when this antibody was developed at the start of the COVID-19 pandemic as there was a general theoretical concern for antibody-dependent*

enhancement (ADE) of COVID-19 disease. Currently, both in vivo and epidemiological evidence disprove this phenomenon for SARS-CoV-2'.

Please confirm that the trials were prospectively registered.

Answer: Thank you for your comment. We confirm that the trials were prospectively registered.

Please ensure that the trial numbers are correct and embed links in the Word document.

Answer: Thank you for your comment. The trial numbers were checked once more. Links were now embedded as requested.

Please add more detail to the Introduction on conventional mAb trials for COVID-19.

Answer: Thank you for your comment. Conventional antibodies were briefly introduced in the original version and now we have added additional references and examples to provide a more exhaustive overview. In addition, we also expanded on examples and comparisons of conventional antibodies in the Discussion section.

Please add details on enrolment of patients: were these patients who would have been hospitalized or were they recruited and hospitalized based on the trial protocol?

Answer: Thank you for your comment. As per inclusion criteria, the patients required medical care in hospital for COVID-19 (i.e., level 4 or 5 of the 8-point ordinal scale). This has been clarified in the Methods section.

Please include the patient questionnaire in the supplementary information.

Answer: Thank you for your comment. The patient questionnaire to evaluate for 14 Common COVID-19-Related Symptoms in the phase 1b study was included in Supplementary Information, on p. 84 of the protocol.

Please avoid the use of 'subjects'.

Answer: Thank you for your comment. 'The term 'subjects' was replaced by 'volunteers' or 'patients' where appropriate.

Please explain why there was a SARS-CoV-2-negative participant in the Phase 1b study.

Answer: Thank you for your comment. Patients were screened on Day –1 and their SARS-CoV-2 infection confirmed with a positive RT-qPCR or antigenic test. On Day 1, viral load (RT-qPCR) was again tested, together with a variant analysis. However, one patient in the 1000 mg cohort who had previously tested positive on Day -1 for viral load, tested negative on Day 1. This has now been explained better in the Results section, under 'Subject demographics and disposition'.

In the introduction, please clarify if the VHH-Fc was shown to neutralize SARS-CoV-2 Delta.

Answer: Thank you for your comment. XVR011 was indeed shown to neutralize the SARS-CoV-2 Delta variant, as demonstrated in Table S1. This was added in the Introduction as follows *'(including the Delta variant)'*.

With respect to dose-response trends, please explicitly state that the observed differences were not statistically significant.

Answer: Thank you for your comment. The text was clarified in the Results section, using the wording suggested by Reviewer 4.

Please include a short, non-technical Author Summary of your research to make findings accessible to a wide audience that includes both scientists and non-scientists. The Author Summary should immediately follow the Abstract in your revised manuscript. This text is subject to editorial change and should be distinct from the scientific abstract. Ideally each sub-heading should contain 2-3 single sentence, concise bullet points containing the most salient points from your study. In the final bullet point of 'What Do These Findings Mean?' Please include the main limitations of the study in non-technical language.

Answer: Thank you for your comment. A non-technical Author summary is now included.

Please reduce the Discussion to 1000 words. Focus on the issues of next steps for VHH-Fc constructs, challenges for infection and for elicitation of ADA responses.
The Discussion must include a paragraph on study limitations.

Answer: Thank you for your comment. The Discussion was reduced to 999 words. Could we please ask a clarifying question on what is meant by 'challenges for infection'?
As stated above, XVR011 is the first VHH-Fc construct evaluated in healthy volunteers and COVID-19 patients. Therefore, discussion of safety, PK and immunogenicity were included.
A paragraph on study limitations was present as follows '*The studies presented here have limitations. Both studies had a limited amount of participants, the Phase 1b was not designed to demonstrate statistical efficacy (small sample size, no placebo control), the history of prior COVID-19 infections was unknown, the duration since last vaccination was variable and the patients were treated with different SOC and infected with different variants. Hence, all these factors may impact interpretation of safety, PK, PD and/or immunogenicity signals. Therefore, conclusions must be drawn carefully. Nonetheless, the studies represent a milestone for the VHH-Fc platform and support the design and further development of VHH-Fc antibody constructs for the prevention and/or treatment of COVID-19 and other infectious diseases.*'.

Please adhere to the PLOS Data Availability guidelines.

Answer: Thank you for your comment. The PLOS Data Availability guidelines were followed at all times.

Please avoid the use of both red and green in the same figures.

Answer: Thank you for your comment. Fig. 2, Fig. S2 and Tables S7 & S8 (now S8 & S9) have been reworked to avoid red and green in the same figure.

Please ensure Figure legends are included, for the main text and supplementary data.

Answer: Thank you for your comment. Legends for the main figures can be found directly below the figures in the Main text. Legends for the supplementary figures can be found directly above the figures in the Supplementary Information.

Comments from the reviewers:

Reviewer #1: The research question is an important one to the community of researchers in this general area because it is the first publication of a VHH-Fc tested in randomized clinical trials. By providing a thorough analysis of the safety, tolerability, pharmacokinetics and immunogenicity of the VHH-Fc in two randomized clinical trials, the results of this publication provide a substantial advance over existing knowledge, with clear implications for patient care, public policy, or clinical research agendas.

While other VHH based therapies have been FDA approved and are in different stages of clinical trials this is the first report of a bivalent VHH-Fc construct being evaluated in humans for safety, tolerability, pharmacokinetics and immunogenicity. The construct evaluated was a bivalent VHH-Fc construct (two identical VHHs fused to an Fc silenced for effector function, using the LALA mutation. The VHH is a the previously characterized VHH72 isolated in llamas against SARS-CoV-1 and was demonstrated to bind to an epitope inaccessible to conventional human antibodies and has potent neutralization of SARS-CoV-1 and SARS-CoV-2. The neutralization is based on both direct competition with ACE2 biding to the spike RBD through steric hindrance and by trapping the RBD in the unstable up conformation resulting in shedding if the S1 subunit. While efficacy was evaluated as an exploratory objective as part of the phase 1b study, the study was not set-up detect statistically significant differences from baseline across cohorts and no placebo control was included for the treatment group. In addition, In March 2022, the study was prematurely terminated before Part 2 was initiated due to the loss of neutralization potency by XVR011 against the Omicron variant BA.2 that was spreading worldwide at the time. This investigation of the first VHH-Fc fusion construct in humans for tolerability, safety, pharmacokinetics, and immunogenicity represents a milestone for further development of VHH-Fc constructs as effective therapeutics for infectious disease especially multivalent constructs that are less

likely to be susceptible to antigenic drift and antibody escape.

To improve the manuscript the authors should give more information about data from clinical trials for conventional anti-SARS-CoV-2 antibodies and compare the dose, tolerability, PK/PD, immunogenicity and ADA. The authors should also provide references and explain more about why ADA might be a greater concern with a llama derived VHH based construct in comparison to a humanized version or a conventional human antibody.

Answer: Thank you for your thoughtful comments.
In the Introduction, anti-SARS-CoV-2 antibodies were mentioned in the original version. They have now been better highlighted through examples and an additional reference (Subramaniam S, Saville JW, Feng F, Freiburger L. Therapeutic Antibodies for Infectious Diseases: Recent Past, Present, and Future. *Biochemistry*. 2025;64(16):3487-3494. doi:10.1021/acs.biochem.5c00192), to limit length.
In the Discussion, the following text has been added '*Anti-drug antibodies (ADA) can, but not necessarily, effect the pharmacokinetics, efficacy and/or safety of therapeutic antibodies. Hence, immunogenicity can be a critical challenge in the development of antibody therapeutics, irrespective of a fully human origin or, in this case, a humanized VHH fused to a human Fc. While humanization significantly reduces immunogenicity compared to earlier chimeric or animal-derived antibodies, it doesn't eliminate it entirely, as the antigen-binding complementarity-determining regions (CDRs) can still elicit an immune response. However, development of ADAs against conventional human antibodies also varies widely and is dependent on numerous factors, including antibody structure, formulation and an individual's genetic predisposition, age and disease status.*'.
Also in the Discussion, we added a comparison to a conventional therapeutic antibody for both safety (' *In comparison, the incidence of drug related TEAE for the conventional anti-SARS-CoV-2 antibody bebtelovimab was 28.6% (150 mg), 25% (600 mg), 14.3% (1200 mg), 50% (2400 mg), and 37.5% (placebo) in healthy volunteers*') and for immunogenicity ('*Bebtelovimab, a conventional anti-SARS-CoV-2 antibody, induced 12.5% ADA responses in healthy volunteers*').

While in most cases the data and analyses fully support the claims, The exception is the statement that the construct is working strictly through neutralization. Several studies have shown that the LALA mutation does not completely abrogate all effector function. The authors should provide a reference for or results of effector function evaluation including ADCC, and CDC for this construct to confirm silencing.

Answer: Thank you for your comment. Strong reduction of XVR011 to FcγR binding was demonstrated and already published in Schepens, B. *et al.* An affinity-enhanced, broadly neutralizing heavy chain-only antibody protects against SARS-CoV-2 infection in animal models. *Sci Transl Med* **13**, (2021) (reference 25). This reference has now been better highlighted and the following has been added in the Introduction: '*The human Fc is engineered with a LALA mutation to strongly reduce the binding to Fc-gamma receptors on immune cells and complement protein C1q, responsible for respectively triggering antibody effector functions like antibody-dependent cell-mediated cytotoxicity (ADCC) and complement-dependent cytotoxicity (CDC). By silencing these interactions, an optimal safety profile was envisioned when this antibody was developed at the start of the COVID-19 pandemic as there was a general theoretical concern for antibody-dependent enhancement (ADE) of COVID-19 disease.*' Currently, both in vivo and epidemiological evidence disprove the ADE phenomenon for SARS-CoV-2.

minor issues:
Line 210 on page 9. Right after Figure S2 Error! Reference source not found.. has two periods.

Answer: thank you, this typographical error was corrected.

Page 20 line 535 should be neutralization not neutralisation

Answer: thank you, this typographical error was corrected.

Reviewer #2: This article offers two small studies of a VHH-FC construct. In the first study an escalating dose proved safe and well tolerated. The drug had expected PK behavior. While it was offered to a few subjects late in the course of cOVID-19 infection hospitalized, resistance of the Omicron stopped this

exploration. In addition, other mAB studies (e.g. REGENCOV) demonstrated lack of benefit of antibodies in hospitalized patients. The data are several years old. While the authors end the paper arguing that these results are a milestone in VHH construct biology, it is hard to see how the results lead to this conclusion?

Reviewer #3: In this study, Jansen et al. ran two phase 1 trials testing the safety of a SARS-CoV-2 neutralising VHH-Fc construct in healthy adults and COVID-19 hospitalised patients. They found that the VHH-Fc drug (XVR011) was well tolerated with mild to moderate treatment related adverse events. Anti-drug antibody was detected in ~31% of COVID-19 patients but not in the healthy adults, though ADA was not reported to affect PK or PD of the drug. While there was a dose-response trend to time of resolution of symptoms or hospital discharge with XVR011 treatment, there are quite a few confounding factors that may cause this (described more in comments below). Overall this was a well described clinical study with detailed methods and results.

Major comments:
For the phase 1b study, the dose response trend for the time of resolution of COVID symptoms (TTRCS) or time to hospital discharge (TTHD) may have been confounded by other factors including number of previous vaccinations or infections. If available, the authors should report the number of previous vaccinations and infections and type of prior vaccination in a table. It is important to know whether these factors were well matched between the different groups.

Answer: Thank you for your important comment. The percentage of vaccinees per cohort, the number of prior COVID-19 vaccination, as well as their types and dates, were added in Table S7. There were less vaccinees in the 250 mg cohort (~38%), but a similar percentage in the 500 mg and 1000 mg cohorts (~80%). In vaccinees, the type of vaccine and time since last vaccination were similar across cohorts. On the other hand, visualization of TTRCS and TTHD suggested a potential improvement mainly for the 1000 mg cohort.
The history of symptomatic COVID-19 infections was not gathered for the phase 1b (part 1 of the EXEVIR0101 trial). And we of course do not have access to the exact history of PCR/antigen test-confirmed infections, as there were no tests at the beginning of the pandemic. We agree that this may be a confounding factor. Finally, small study size and SoC was evolving during the course of the phase 1b trial which may have confounded the efficacy results as well.
Overall we agree that there might be confounding factors to the efficacy assessment, which is nevertheless important to report as an endpoint of the phase 1b trial. We have toned down the results using the language suggested by reviewer 4 and added the following: '*Change in circulating variants from Delta to Omicron or recombinant over the course of the study cannot explain this possible trend, as XVR011 is less potent on Omicron than on Delta variants (Table S1). Percentage of patients having received a prior COVID-19 vaccine, number and type of prior COVID-19 vaccinations, as well as date since last vaccination, were similar in the 500 and 1000 mg cohorts, while there were less vaccinees in the 250 mg cohort (Table S7). However, small study size, unknown history of prior COVID-19 infections and evolving standard of care remain confounding factors, and the results should be interpreted with care*.'. Similarly, we have toned down the discussion.

Also, the 1000mg group had more Omicron and recombinant infections than the 250 and 500mg groups, which mostly had Delta infection. Since Omicron and recombinant variants may have had different infection kinetics and outcomes compared to Delta, the time to COVID-19 symptom resolution or time to hospital discharge could have been due to the infecting variant, rather than treatment with XVR011. As such, the authors should tone down the fact that there was a dose-response trend for treatment throughout the article and abstract. Especially given there was no statistical basis for these statements.

Answer: Thank you for this important point. Visualization of TTRCS and TTHD suggested a potential improvement mainly for the 1000 mg cohort, where there were more Omicron and recombinant infections than the 250 and 500mg cohorts, which mostly had Delta infection. On the other hand, XVR011 is far less potent on Omicron variants than on Delta variants (Table S1). Therefore, it should be less efficacious in the 1000 mg cohort than in the 250 and 500 mg cohorts. Therefore, change in circulating variants cannot explain the results observed. This point has been clarified in the Results section, as mentioned in the previous comment.

It was interesting that there was only detectable ADA in the COVID patients and not in the healthy controls. Do the authors know whether the ADA were directed towards the llama VHH portion (VHH72) rather than the human IgG-LALA portion? Can the authors run a similar ADA assay with VHH72 alone and with an IgG-LALA control antibody of another specificity?

Answer: Thank you for your comment. We agree that this is a very interesting question. However, we passed the time limitation of the ICF to do so.

Minor comments:
Is reference 42 a full reference?

Answer: Thank you for your comment. Yes, it corresponds to the documentation created by the Committee for Medicinal Products for Human Use on Cablivi. A link has been added for clarity.

Figure 2 - the mean lines would be clearer if you could make the thin lines more faint or transparent.

Answer: Thank you for your comment. Figure 2 was adapted as suggested.

Reviewer #4: Statistical review
This paper reports two phase I trials of XVR011 in healthy volunteers and mild-moderate covid patients. As is appropriate for phase I trials, much of the results was not subject to hypothesis testing. I had some minor comments on the statistical aspects of the paper:
1. Abstract: "Efficacy in subjects hospitalized with mild to moderate COVID-19 was evaluated as a secondary objective in the Phase 1b study." - it would be useful to add the endpoint used to measure efficacy here.

Answer: Thank you for your comment. The term '*antiviral activity*' has been added to better explain how efficacy was measured, while limiting Abstract length.

2. Abstract: "Although the study was not powered to detect statistically significant differences, a dose-response trend was observed for time to disappearance of COVID-19-related symptoms, as well as for time to hospital discharge." - I did not see that this statement was supported by the results as currently written as it initially made me assume some statistical testing had been done. Perhaps if it were rewritten along the lines of 'Visualisations of time to… suggested a possible dose-response trend', this might be clearer to the reader.

Answer: Thank you for this proposal, which we have adopted.

3. Line 176-177: I am not convinced that simple randomisation would be the approach used here as would mean there's a decent chance that (for example) 2 sentinel subjects would get placebo or dose. I suspect it was a block randomisation approach that ensured the number getting placebo/drug was as required?

Answer: Thank you for your comment. A sentinel dosing design was adopted to ensure safety: 10 volunteers per cohort, the first 2 in each cohort being sentinel – 1 receiving XVR011, 1 receiving placebo. This was stated lines 185-187.

4. Line 288-289: I am not familiar with the power model with mixed effects, can a bit more detail about what the model is estimating, and/or a reference, be added?

Answer: Thank you for your comment. No statistical methods were mentioned in lines 288-289. The power model with mixed effects was mentioned in lines 331-335. The following reference has been added: Sethuraman VS, Leonov S, Squassante L, Mitchell TR, Hale MD. Sample size calculation for the Power Model for dose proportionality studies. Pharm Stat. 2007 Jan-Mar;6(1):35-41. doi: 10.1002/pst.241. PMID: 17323313.

5. Lines 385-386: is Figure S5 the correct reference for this? I did not follow how it connected to the statement. It would be interesting to know what the estimated power parameter was, together with a confidence interval - is this available?

Answer: Thank you for your comment, which we are not sure we understand correctly. Figure S5 is not mentioned lines 385-386. Figure S5 displays the residuals versus time and predictions, as part of the quality check of the population PK model.

6. Line 498: I would recommend this is rewritten similarly to my comment 2.

Answer: Thank you for your thoughtful comment. As suggested, line 498 was rewritten as per comment above.

James Wason

Any attachments provided with reviews can be seen via the following link:

--- ---
General editorial requests:

(Note: not all will apply to your paper, but please check each item carefully)

* We ask every co-author listed on the manuscript to fill in a contributing author statement, making sure to declare all competing interests. If any of the co-authors have not filled in the statement, we will remind them to do so when the paper is revised. If all statements are not completed in a timely fashion this could hold up the re-review process. If new competing interests are declared later in the revision process, this may also hold up the submission. Should there be a problem getting one of your co-authors to fill in a statement we will be in contact. Please do not add or remove authors without first discussing this with the handling editor. You can see our competing interests policy here: http://journals.plos.org/plosmedicine/s/competing-interests.

Answer: Thank you for your comment, which we understand.

* Please upload any figures associated with your paper as individual TIF or EPS files with 300dpi resolution at resubmission; please read our figure guidelines for more information on our requirements: http://journals.plos.org/plosmedicine/s/figures. While revising your submission, we strongly recommend that you use PLOS's NAAS tool (https://ngplosjournals.pagemajik.ai/artanalysis) to test your figure files. NAAS can convert your figure files to the TIFF file type and meet basic requirements (such as print size, resolution), or provide you with a report on issues that do not meet our requirements and that NAAS cannot fix.

After uploading your figures to PLOS's NAAS tool - https://ngplosjournals.pagemajik.ai/artanalysis, NAAS will process the files provided and display the results in the "Uploaded Files" section of the page as the processing is complete.
If the uploaded figures meet our requirements (or NAAS is able to fix the files to meet our requirements), the figure will be marked as "fixed" above. If NAAS is unable to fix the files, a red "failed" label will appear above.
When NAAS has confirmed that the figure files meet our requirements, please download the file via the download option, and include these NAAS processed figure files when submitting your revised manuscript.

Answer: Thank you for your comment, which we have followed.

* Please ensure that the paper adheres to the PLOS Data Availability Policy (see http://journals.plos.org/plosmedicine/s/data-availability), which requires that all data underlying the study's findings be provided in a repository or as Supporting Information. For data residing with a third party, authors are required to provide instructions with contact information (web or email address) for obtaining the data. Please note that a study author cannot be the contact person for the data. PLOS journals do not allow statements supported by "data not shown" or "unpublished results." For such statements, authors must provide supporting data or cite public sources that include it.

Answer: Thank you for your comment. All data are available as Supplementary Information.

* We expect all researchers with submissions to PLOS in which author-generated code underpins the findings in the manuscript to make all author-generated code available without restrictions upon publication of the work. In cases where code is central to the manuscript, we may require the code to be made available as a condition of publication. Authors are responsible for ensuring that the code is reusable and well documented. Please make any custom code available, either as part of your data deposition or as a supplementary file. Please add a sentence to your data availability statement regarding

any code used in the study, e.g. "The code used in the analysis is available from Github [URL] and archived in Zenodo [DOI link]" Please review our guidelines at https://journals.plos.org/plosmedicine/s/materials-software-and-code-sharing and ensure that your code is shared in a way that follows best practice and facilitates reproducibility and reuse. Because Github depositions can be readily changed or deleted, we encourage you to make a permanent DOI'd copy (e.g. in Zenodo) and provide the URL.

Answer: Thank you for your comment. No author-generated code was used in this manuscript.

* Please ensure that the study is reported according to the CONSORT 2025 guideline and include the completed CONSORT 2025 checklist as Supporting Information. When completing the checklist, please use section and paragraph numbers, rather than page numbers. Please add the following statement, or similar, to the Methods: "This study is reported as per CONSORT 2025 guideline (S1 Checklist)."

Answer: Thank you for your comment. The statement "This study is reported as per CONSORT 2025 guideline (S1 Checklist)." was added to the Methods section.

FORMATTING – GENERAL
* Abstract: Please structure your abstract using the PLOS Medicine headings (Background, Methods and Findings, Conclusions). Please combine the Methods and Findings sections into one section.

Answer: Thank you for your comment. The Abstract was formatted as per request.

* At this stage, we ask that you include a short, non-technical Author Summary of your research to make findings accessible to a wide audience that includes both scientists and non-scientists. The Author Summary should immediately follow the Abstract in your revised manuscript. This text is subject to editorial change and should be distinct from the scientific abstract. Ideally each sub-heading should contain 2-3 single sentence, concise bullet points containing the most salient points from your study. In the final bullet point of 'What Do These Findings Mean?', please include the main limitations of the study in non-technical language. Please see our author guidelines for more information: https://journals.plos.org/plosmedicine/s/revising-your-manuscript#loc-author-summary.

Answer: Thank you for your comment. A non-technical Author summary was added after the Abstract.

* Please express the main results with 95% CIs as well as p values. When reporting p values please report as p<0.001 and where higher as the exact p value p=0.002, for example. Throughout, suggest reporting statistical information as follows to improve clarity for the reader "22% (95% CI [13%,28%]; p</=)". Please be sure to define all numerical values at first use.

Answer: Thank you for your comment. 95%CI for the PK parameters were presented in Table S5. Due to the small sample size of the two studies, no calculation of p-values was made in regard to any outcomes.

* Please include page numbers and line numbers in the manuscript file. Use continuous line numbers (do not restart the numbering on each page).

Answer: Thank you for your comment. Page and continuous line numbers were added in the original submission.

* Please cite the reference numbers in square brackets. Citations should precede punctuation.

Answer: Thank you for your comment. Reference numbers were formatted as per request.

FIGURES AND TABLES
* Please provide titles and legends for all figures and tables (including those in Supporting Information files).

Answer: Thank you for your comment. The titles and legends for the main figures were below the figures in the main text, and those for the supplementary figures were above the figures in the Supplementary Information.

* Please define all abbreviations used in each figure/table (including those in Supporting Information

files).

Answer: Thank you for your comment. An abbreviations list was added in the original version, before the Introduction. We have double-checked it for omissions.

* Please consider avoiding the use of red and green in order to make your figure more accessible to those with color blindness.

Answer: Thank you for your comment. Figures S2 and Tables S7 & S8 (now S8 & S9) were reworked to avoid red and green in the same figure.

SUPPLEMENTARY MATERIAL

* Please note that supplementary material will be posted as supplied by the authors. Therefore, please amend it according to the relevant comments outlined here.

Answer: Thank you for your comment. Supplementary materials were reworked as requested.

* Please cite your Supporting Information as outlined here:
https://journals.plos.org/plosmedicine/s/supporting-information

Answer: Thank you for pinpointing to the correct guideline.

REFERENCES

* PLOS uses the numbered citation (citation-sequence) method and first six authors, et al.

Answer: Thank you for your comment. We have followed the requested formatting.

* Please ensure that journal name abbreviations match those found in the National Center for Biotechnology Information (NCBI) databases (http://www.ncbi.nlm.nih.gov/nlmcatalog/journals), and are appropriately formatted and capitalised.

Answer: Thank you for your comment. We have followed the requested formatting.

* Where website addresses are cited, please include the complete URL and specify the date of access (e.g. [accessed: 12/06/2023]).

Answer: Thank you for your comment. We have followed the requested formatting.

* Please also see https://journals.plos.org/plosmedicine/s/submission-guidelines#loc-references for further details on reference formatting.

Answer: Thank you for your comment. We have followed the requested formatting.

RCTs:

* PLOS Medicine requires that all trials be prospectively registered in one of registries recognized by WHO. Please ensure that study registration details are included in the Methods section.

Answer: Thank you for your comment. Trials were prospectively registered in one of the registries recognized by WHO. The study registration details were included in the Methods section and have now been added in the Abstract as per above request.

* Please structure the Methods section using the following sub-headings: Study design and participants, Randomization and masking, Procedures, Outcomes, Statistical analysis.

Answer: Thank you for your comment. We have adhered as best as possible to the proposed formatting, while reducing the length of the Methods section.

* The following outcomes measures [ADD DETAILS AS NEEDED OR DELETE BULLET POINT] appear to differ between the submitted manuscript and the protocol [and/or trial registry]. Please clarify and explain all discrepancies between the paper and protocol. If the outcomes were not prespecified in the

protocol, please define them in the Methods (Outcomes section) as post hoc and explain why they were added. Post-hoc comparisons should be presented as hypothesis generating rather than conclusive.

Answer: Thank you for your comment. We assume that this bullet point should have been deleted.

* Please ensure that all prespecified outcomes (primary, secondary, and exploratory) are listed in the Methods/Outcomes section and indicate whether there are outcomes that are not presented in the current report.

Answer: Thank you for your comment. All prespecified outcomes were presented in the manuscript, both in the Methods and in the Outcomes sections.

* Please specify the dates (Month Day, Year) during which study enrollment and follow up occurred.

Answer: Thank you for your comment. The dates of study enrollment were added. Follow-up was performed as per the time-event schedule displayed in the protocol.

* Please include absolute numbers wherever you report percentages; eg, n/N (%)

Answer: Thank you for your comment. Absolute numbers were reported in the original manuscript.

* Please present the safety data for the study including numbers of specific events and whether or not adverse events are thought to be related to treatment. AEs should be reported in the abstract, per CONSORT and CONSORT-Harms.

Answer: Thank you for your comment. AE were reported in the Abstract, per both clinical trials presented here.

* Please complete the CONSORT checklist (https://www.equator-network.org/reporting-guidelines/consort/) and ensure that all components of CONSORT are present in the manuscript, including how randomization was performed, allocation concealment, blinding of intervention, definition of lost to follow-up, power statement. When completing the checklist, please use section and paragraph numbers, rather than page numbers.

Answer: Thank you for your comment. The CONSORT checklist originally added was adapted to follow the 2025 version.

* Please report your abstract according to CONSORT for abstracts, following the PLOS Medicine abstract structure (Background, Methods and Findings, Conclusions) https://www.equator-network.org/reporting-guidelines/consort-abstracts/

Answer: Thank you for your comment. This format was implemented.

* If your trial had to undergo important modifications in response to extenuating circumstances, please complete the CONSERVE-CONSORT checklist and provide in your Supporting Information; (https://www.equator-network.org/reporting-guidelines/guidelines-for-reporting-trial-protocols-and-completed-trials-modified-due-to-the-covid-19-pandemic-and-other-extenuating-circumstances-the-conserve-2021-statement/). When completing the checklist, please use section and paragraph numbers, rather than page numbers.

Answer: Thank you for your comment. There were no extenuating circumstances for the phase 1a and b studies presented here.

* In keeping with our commitment to Open Science, please include the study protocol document and analysis plan (including any amendments) as Supporting Information to be published with the manuscript if accepted.

Answer: Thank you for your comment. Both were included in the original version.

* Please note that PLOS Medicine requires prospective, public registration of a data sharing plan (as part of mandatory clinical trials registration) for all clinical trials that began enrollment on or after January 1, 2019, in accordance with ICMJE requirements.

Answer: Thank you for your comment. This was mentioned in the original version.

Technical requests

1) Thank you for stating “EJ, VB, FH, AB, MR and DT are/were employees of ExeVir Bio at the time of the work presented in this manuscript. POG, SH, NG and MM were contracted by Exevir to perform the work presented in this manuscript.” Please declare all competing interests beginning with the statement "I have read the journal's policy and the authors of this manuscript have the following competing interests:"

Answer: Thank you for your comment. The above statement was added as requested.

2) Your current Financial Disclosure states, “This work was financially supported by VLAIO (Agentschap Innoveren & Ondernemen), Project HBC.2020.3083, SPW (Service Public de Wallonie)-Recherche, convention 8495 and the European Commission Horizon 2020 Framework Programme, Project 101045949 — XVR011 Phase 2.”. However, your funding information on the submission form indicates one fund. Please ensure that the funders and grant numbers match between the Financial Disclosure field and the Funding Information tab in your submission form. Note that the funders must be provided in the same order in both places as well.

Answer: Thank you for spotting this mistake. It was corrected.

3) We ask that a manuscript source file is provided at Revision. Please upload your manuscript file as a .doc, .docx, .rtf or .tex.

Answer: Thank you for your comment. A version with track changes and a cleaned version will both be provided at Revision.

4) Please upload the main figures as separate figure files in .tif or .eps format. For more information about figure files please see our guidelines:

https://journals.plos.org/plosmedicine/s/figures

https://journals.plos.org/plosmedicine/s/figures#loc-file-requirements

Answer: Thank you for your comment. The main figures will be uploaded as requested here and above.

5) We have noticed that you have uploaded Supporting Information files, but you have not included a list of legends. Please add a full list of legends for your Supporting Information files after the references list.

Answer: Thank you for your comment. The legends for the supplementary figures were added after the references list in the Supporting Information files, as requested.

6) In your Data Availability Statement, please ensure you have provided all necessary access information in complete sentences. Access information should include the name of any data repositories used as well as digital object identifiers (DOIs), accession numbers or codes, or other persistent identifiers for all relevant data. If all data has been provided within your submission files, please use the statement "All relevant data is available within the manuscript and Supporting Information files." The Data Availability Statement will appear on the published version of your manuscript.

Answer: Thank you for your comment, which we had followed.
