# Supplementary material for: Characterization of the VHH-Fc construct rimteravimab in healthy adults and patients hospitalized for mild-to-moderate COVID-19: Two Phase 1 randomized clinical trials
Source: PLoS Med. 2026 May 29;23(5):e1004609. doi: 10.1371/journal.pmed.1004609 (PMC13221044; doi:10.1371/journal.pmed.1004609)
Supplement: Attachment — Submitted filename: AnswersToReviewersComments.pdf [file pmed.1004609.s007.pdf]

PMEDICINE-D-25-00069R2
Answers to the Reviewers' Comments

Dear Alison, Dear PLOS Medicine Editorial Board,

On behalf of my co-authors, I would like to thank you and the reviewers for all your insightful comments to improve the quality of our manuscript. Please find our answers below in blue, as well as in the Revised manuscript (version with track changes and 'cleaned' version).

Best wishes,
Florence Herschke, PhD, on behalf of all authors

Dear Dr. Herschke,

Thank you very much for re-submitting your manuscript "First-in-Human characterization of VHH-Fc construct XVR011 (rimteravimab) in healthy adults and patients hospitalized for mild-to-moderate COVID-19: two Phase 1 randomized clinical trials." (PMEDICINE-D-25-00069R2) for review by PLOS Medicine.

I have discussed the paper with my colleagues and the academic editor and it was also seen again by 3 reviewers. I am pleased to say that provided the remaining editorial and production issues are dealt with we are planning to accept the paper for publication in the journal.

The remaining issues that need to be addressed are listed at the end of this email. Any accompanying reviewer attachments can be seen via the link below. Please take these into account before resubmitting your manuscript:

Please note while forming your response, if your article is accepted, you may have the opportunity to make the peer review history publicly available. The record will include editor decision letters (with reviews) and your responses to reviewer comments. If eligible, we will contact you to opt in or out.

In revising the manuscript for further consideration here, please ensure you address the specific points made by each reviewer and the editors. In your rebuttal letter you should indicate your response to the reviewers' and editors' comments and the changes you have made in the manuscript. Please submit a clean version of the paper as the main article file. A version with changes marked must also be uploaded as a marked up manuscript file.

Please also check the guidelines for revised papers at http://journals.plos.org/plosmedicine/s/revising-your-manuscript for any that apply to your paper.

Answer: Thank you for your comment. The guidelines for revised papers were followed.

If you haven't already, we ask that you provide a short, non-technical Author Summary of your research to make findings accessible to a wide audience that includes both scientists and non-scientists. The Author Summary should immediately follow the Abstract in your revised manuscript. This text is subject to editorial change and should be distinct from the scientific abstract.

Answer: Thank you for your comment. An author summary has been submitted in the previous revision.

In addition to these revisions, you may need to complete some formatting changes, which you will receive in a follow up email. A member of our team will be in touch with a set of requests shortly. If you do not receive a separate email within a few days, please assume that checks have been completed, and no additional changes are required.

We expect to receive your revised manuscript within 1 week. Please email us (plosmedicine@plos.org) if you have any questions or concerns.

We ask every co-author listed on the manuscript to fill in a contributing author statement. If any of the co-authors have not filled in the statement, we will remind them to do so when the paper is revised. If all statements are not completed in a timely fashion this could hold up the re-review process. Should there be a problem getting one of your co-authors to fill in a statement we will be in contact. YOU MUST NOT ADD OR REMOVE AUTHORS UNLESS YOU HAVE ALERTED THE EDITOR HANDLING THE MANUSCRIPT TO THE CHANGE AND THEY SPECIFICALLY HAVE AGREED TO IT.

Please ensure that the paper adheres to the PLOS Data Availability Policy (see http://journals.plos.org/plosmedicine/s/data-availability), which requires that all data underlying the study's findings be provided in a repository or as Supporting Information. For data residing with a third party, authors are required to provide instructions with contact information for obtaining the data. PLOS journals do not allow statements supported by "data not shown" or "unpublished results." For such statements, authors must provide supporting data or cite public sources that include it.

To enhance the reproducibility of your results, we recommend that you deposit your laboratory protocols in protocols.io, where a protocol can be assigned its own identifier (DOI) such that it can be cited independently in the future. Additionally, PLOS ONE offers an option to publish peer-reviewed clinical study protocols. Read more information on sharing protocols at https://plos.org/protocols?utm_medium=editorial-email&utm_source=authorletters&utm_campaign=protocols

Answer: Thank you for your comment. The PLOS Data Availability Policy has been followed.

Please review your reference list to ensure that it is complete and correct. If you have cited papers that have been retracted, please include the rationale for doing so in the manuscript text, or remove these references and replace them with relevant current references. Any changes to the reference list should be mentioned in the rebuttal letter that accompanies your revised manuscript.

Answer: Thank you for your comment. The reference list has been checked and adapted where necessary. The following references have been added:

Relan P, Motaze NV, Kothari K, Askie L, Le Polain O, Van Kerkhove MD, et al. Severity and outcomes of Omicron variant of SARS-CoV-2 compared to Delta variant and severity of Omicron sublineages: a systematic review and metanalysis. BMJ Glob Health. 2023;8. doi:10.1136/bmjgh-2023-012328

31. Lewnard JA, Hong VX, Patel MM, Kahn R, Lipsitch M, Tartof SY. Clinical outcomes associated with SARS-CoV-2 Omicron (B.1.1.529) variant and BA.1/BA.1.1 or BA.2 subvariant infection in Southern California. Nature Medicine 2022 28:9. 2022;28: 1933–1943. doi:10.1038/s41591-022-01887-z

32. Doll MK, Waghmare A, Heit A, Levenson Shakoor B, Kimball LE, Ozbek N, et al. Acute and Post-Acute COVID-19 Outcomes Among Immunologically Naïve Adults During Delta Versus Omicron Waves. medRxiv. 2022; 2022.11.13.22282222-2022.11.13.22282222. doi:10.1101/2022.11.13.22282222

Please note, when your manuscript is accepted, an uncorrected proof of your manuscript will be published online ahead of the final version, unless you've already opted out via the online submission form. If, for any reason, you do not want an earlier version of your manuscript published online or are unsure if you have already indicated as such, please let the journal staff know immediately at plosmedicine@plos.org.

If you have any questions in the meantime, please contact me or the journal staff on plosmedicine@plos.org.

We look forward to receiving the revised manuscript by Feb 27 2026 11:59PM.

Sincerely,

Alison Farrell, Ph.D.
Senior Editor
PLOS Medicine

plosmedicine.org

Requests from Editors:

Use the active voice throughout.

Answer: Thank you for your comment. The active voice has been used throughout.

**Please note the requirements to remove claims of primacy in the General Formatting comments below. This needs to be removed throughout the manuscript and from the title.

Answer: Thank you for your comment. All claims of primacy have been removed throughout the manuscript.

Please keep the tense consistent in the Abstract, e.g. line 78, switch to past tense.
Answer: Thank you for your comment. The error has been corrected.

Please provide additional background context to the Background section of the Abstract. The last sentence should explain the problem tackled in the manuscript.

Answer: Thank you for your comment. The following sentence has been added: 'It was tested in two Phase 1 studies, including either healthy volunteers or hospitalized COVID-19 patients to evaluate safety, tolerability, pharmacokinetics and immunogenicity.'

Secondary objectives can be presented in the Abstract if ALL secondary objectives are reported in the manuscript. If only some are reported in the manuscript, this must be disclosed, and they must be removed from the Abstract. At present the Abstract lacks sufficient detail about how efficacy was assessed.

Answer: Thank you for your comment. All secondary objectives are reported. The following has been added to clarify how efficacy (secondary objective in the phase 1b study) was assessed: 'Clinical score evolved similarly between all dose levels.'.

Line 78: add 'of XVR011' after tolerability.

Answer: Thank you for your comment. 'of XVR011' has been added.

Please indicate length of follow up of treated hospitalized patients in the Abstract.

Answer: Thank you for your comment. The following has been added on line 91: ' and followed up to 29 days'.

Please indicate that descriptive statistics were used.

Answer: Thank you for your comment. The use of descriptive statistics is mentioned in the Methods section under 'Statistical analysis'.

Please revise your financial disclosure to acknowledge that Exevir is a funder given that some of the authors were contracted by Exevir to perform the work. It does not seem correct to indicate that the finders had no role in study design, data collection and analysis, decision to publish, or preparation of the manuscript.

Answer: Thank you for your comment. The following sentences have been added: ' and by ExeVir Bio BV. Apart from ExeVir, the funders had no role in study design, data collection and analysis, decision to publish, or preparation of the manuscript.'.

Author Summary—revise the title please

Answer: Thank you for your comment. The title now simply reads 'AUTHOR SUMMARY'.

Please remove the specifics of antibody halflife from the Author Summary

Answer: Thank you for your comment. The specifics have been deleted.

Introduction: please explain why XVR011 was developed, and targeting which SARS-CoV-2 variant.

Answer: Thank you for your comment. The explanation was present as follows: 'VHH antibodies are being developed as SARS-CoV-2 antibody-based therapeutics, with the goal to access different, potentially more conserved, epitopes on the spike compared to conventional antibodies.'. To clarify, the following has been added in the Introduction: 'VHH72, the VHH used to construct XVR011, had already been discovered prior to the start of the SARS-CoV-2 pandemic. It was isolated from a llama immunized with prefusion-stabilized MERS and SARS-CoV-1 spike proteins and binds to an epitope in the RBD of the spike protein that is difficult to access for human antibodies. At the start of the pandemic, VHH72 was found to also neutralize SARS-CoV-2 with high potency and re-purposed for the development of XVR011.'.

Line 156: Complimentary should be Complementary

Answer: Thank you for your comment. The typographical error has been corrected.

Line 159: add 'individuals' after immunocompromised.

Answer: Thank you for your comment. 'individuals' has been added.

The last sentence of the Introduction is repeated twice. Please delete one sentence and add a clarifying sentence to explain what the two studies aimed to investigate with respect to XVR011

Answer: Thank you for your comment. The repeated sentence has been deleted. The following has been added: ', aiming to explore the safety, tolerability, pharmacokinetics, efficacy and immunogenicity profiles of XVR011'.

Please reorder the Introduction to start with the second paragraph. The first paragraph should be moved later.

Answer: Thank you for your comment. The first and second paragraphs were inverted.

Methods:
Please include the exact participant enrolment dates for the participants of EXEVIR0102 in the first paragraph.

Answer: Thank you for your comment. The dates have been added.

Please explain how participants were recruited and if compensated.

Answer: Thank you for your comment. Healthy volunteers of the phase 1a trial were recruited as per usual via advertisement on PRA Healthcare website and mailing their pool of potential participants. They were compensated. On the other hand, participants of the phase 1b study were recruited in the hospitals associated to the clinical trial sites and were not compensated. This information has been added to the Supplementary Information, under Methods, Study design.

Please include information on participant consent for the two trials in their initial description in the Methods.

Answer: Thank you for your comment. The following sentence is already present in the Methods section, under the first paragraph 'Study Ethics': 'All subjects provided written informed consent before study entry.'.

The Methods need to include a specific section describing the protocol amendments.

Answer: Thank you for your comment. Specific sections on protocol amendments already exist in the Methods section of the Supplementary Information. The rationale for the protocol amendments is described in the table on page 6.

**The manuscript does not mention a placebo control for the Phase 1a in healthy volunteers, it is only evident from Table 2. This needs to be clarified from the outset, including in the Abstract.

Answer: Thank you for your comment. In the abstract, it is stated in the second sentence of the 'Methods and Findings' paragraph. In the Methods section, Under the 'Study design and procedures' section, the following sentence is included: 'EXEVIR0102 (EudraCT number: 2021-003707-17; Figure S1) was a Phase

1a, randomized, double-blind, placebo-controlled single ascending dose study to evaluate the safety and pharmacokinetics of XVR011.'

Results:
Line 318: should primate be plural?

Answer: Thank you for your suggestion. The plural has been adopted.

Line 338: please fix spacing of "6patients"

Answer: Thank you for spotting. The spacing has been fixed.

Line 447: revise statement as per reviewer comments.

Answer: Thank you for your comment. This has been implemented, as explained in the response to the Reviewers' comments.

Line 450: replace 'less' with 'fewer'

Answer: Thank you for your suggestion. 'less' has been replaced with 'fewer'.

Discussion:
Line 518: replace "can, but not necessarily" with "may" (phrasing as written is awkward)

Answer: Thank you for your suggestion. The replacement has been made.

Please temper statements, e.g. 'milestone', It is preferable to discuss further the potential for use of this platform to respond quickly to infectious disease threats, rather than repeat throughout the claim of primacy. The readership of PLOS Medicine is interested in the clinical potential of new therapeutics, which the Discussion should highlight, rather than reiterate all of the results.

Answer: Thank you for your comment. The claims of primacy have been toned down as follows: ; (ii) the variations in transaminase levels have been removed, as non clinically relevant; (iii) 'represent a milestone' has been replaced by 'highlight the potential of'.

Please insert figures according to PLOS Medicine formatting requirements.

Answer: Thank you for your comment. This has been implemented.

Please format references according to PLOS Medicine formatting requirements.

Answer: Thank you for your comment. The references were formatted according to the 'Vancouver' style, stated PLOS Medicine formatting guidelines.

General Formatting
* Please review your text for claims of novelty or primacy (e.g. 'for the first time') and remove this language. In addition, please check that any use of statistical terms (such as trend or significant) are supported by the data, and if not please remove them.

Answer: Thank you for your comment. The claims were added to help the audience understand the scope of the work beyond. They have been toned down.

"* Statistical reporting: Please revise throughout the manuscript, including tables and figures.
- Please report statistical information as follows to improve clarity for the reader ""22% (95% CI [13,28]; p</=)"".

Answer: Thank you for your comment. Formatting has been imported from a recently published article in PLOS Medicine (10.1371/journal.pmed.1004842). Descriptive statistics were largely used rather than statistical tests. Therefore, no p-values were calculated except in Tables S5-6.

- Please separate upper and lower bounds with commas instead of hyphens as the latter can be confused with reporting of negative values.

Answer: Thank you for your comment. This was implemented.

- Please repeat statistical definitions (HR, CI etc.) for each set of parentheses."

Answer: Thank you for your comment. This has been implemented.

* In the last sentence of the Abstract Methods and Findings section, please describe the main limitation(s) of the study's methodology.

Answer: Thank you for your comment. The following sentence has been added: 'Technological limitations of the studies, including study size, absence of placebo for the phase 1b, absence of repeated dosing, evolving SARS-CoV-2 variants and standard-of-care may have affected the interpretation of the safety, tolerability, pharmacokinetic and immunogenicity signals.'.

* Please replace "subject" with participant, patient, individual, or person.

Answer: Thank you for your comment. This is already implemented in the manuscript.

* It appears that one or more study authors is affiliated with one or more of the agencies that funded the study. Thus, the statement "The funders had no role in study design, data collection and analysis, decision to publish, or preparation of the manuscript" does not apply. Please revise the Financial Disclosure accordingly, as in "[Author name] is [author's role] at [funding agency]. The funders had no other role in study design….."

Answer: Thank you for your comment. The financial statement has been modified as stated above.

* Thank you for agreeing to make your data available. At this time, please provide the link to the data repository and accession numbers required for access.

Answer: Thank you for your comment. The following has been added to the manuscript: 'Data can be obtained upon motivated request at info@exevir.com'. The same is being implemented in ClinicalTrials.gov for the phase 1b study record (NCT04884295).

* Please specify whether informed consent was written or oral. Please ensure that the research complies with the PLOS policy in full: https://journals.plos.org/plosmedicine/s/human-subjects-research#loc-patient-privacy-and-informed-consent-for-publication

Answer: Thank you for your comment. The following sentence is already included in the 'Methods' section, under 'Study Ethics': 'All subjects provided written informed consent before study entry.'.

* Please provide titles and legends for all figures and tables (including those in Supporting Information files). Please define all acronyms used in each figure or table in its corresponding legend.

Answer: Thank you for your comment. This has been implemented.

* In the Kaplan-Meier curve(s) please provide the number at risk for each time interval.

Answer: Thank you for your comment. This has been implemented.

* Please consider avoiding the use of red and green in order to make your figure more accessible

Answer: Thank you for your comment. This was already implemented in the previous revision.

* Please confirm that your title complies with PLOS Medicine's style. Your title must be nondeclarative and not a question. It should begin with main concept if possible. "Effect of" should be used only if causality can be inferred, i.e., for an RCT. Please place the study design ("A randomized controlled trial," "A retrospective study," "A modelling study," etc.) in the subtitle (ie, after a colon).

Answer: Thank you for your comment. We confirm that our title complies with PLOS Medicine's style.

* Please confirm that your abstract complies with our requirements, including format (three sections: Background, Methods and Findings, and Conclusions) and providing all the information relevant to this study type https://journals.plos.org/plosmedicine/s/submission-guidelines#loc-abstract

Answer: Thank you for your comment. We confirm that our abstract complies with PLOS Medicine's requirements.

* Please ensure that the Introduction ends with a clear description of the study question or hypothesis.

Answer: Thank you for your comment. As requested above, the following sentence has been added at the end of the Introduction: '... aiming to explore the safety, tolerability, pharmacokinetics, efficacy and immunogenicity profiles of XVR011'.

* Please ensure that all abbreviations are defined at first use throughout the text.

Answer: Thank you for your comment. This is already implemented.

* Please confirm that all numbers presented in the abstract are present and identical to numbers presented in the main manuscript text.

Answer: Thank you for your comment. We confirm that all numbers presented in the abstract are present and identical to numbers presented in the Methods and Results sections of the manuscript.

Clinical Trial Reporting Requirements
* Please complete the CONSORT 2025 checklist and ensure that all components of CONSORT 2025 are present in the manuscript, including how randomization was performed, allocation concealment, blinding of intervention, definition of lost to follow-up, power statement. When completing the checklist, please use section and paragraph numbers, rather than page numbers. The checklist should be included as supporting information, and should be cited in the article.

Answer: Thank you for your comment. The CONSORT 2025 checklist is already added as Supplementary Information.

"* As your trial had to undergo important modifications in response to extenuating circumstances, please complete the CONSERVE-CONSORT checklist and provide in your Supporting Information.
When completing the checklist, please use section and paragraph numbers, rather than page numbers."

Answer: Thank you for your comment. The CONSERVE-CONSORT checklist has been added to the Supplementary Information.

* PLOS Medicine requires that all trials be prospectively registered in one of registries recognized by WHO. Please provide information on study registration in the Methods section.

Answer: Thank you for your comment. This is already implemented in the previous revision. We have added the EudraCT registration number for the phase 1b as well, in the Abstract and Methods sections.

* Your trial was registered after the participants were randomized. Please explain in the paper why your trial was registered late. In your rebuttal letter, please indicate if you are conducting or have conducted any related or similar trials, and confirm that those have been registered.

Answer: Thank you for your comment. On ClincalTrials.gov, the first version of the phase 1b study (NCT04884295) was created on 7 May 2021, prior to first patient enrolled on 26 August 2021. We have not conducted and are not conducting similar trials.

* Some of the outcome measures or methods appear to differ between the submitted manuscript and the trial registry and/or protocol. Please clarify and explain the discrepancy. If the outcomes were not prespecified in the protocol, please indicate that they were post hoc and explain why they were added. Post hoc comparisons should be presented as hypothesis generating rather than conclusive.

Answer: Thank you for your comment. The methods and outcome measures for the primary and secondary endpoints are similar between the protocol, study report and manuscript. In the manuscript, post-hoc comparisons have been added to deepen the analysis of the data. They have been clarified to ensure that they are presented as hypothesis-generating – thank you for this thoughtful note.

* In accordance with ICMJE requirements, PLOS Medicine requires prospective, public registration of a data sharing plan (as part of mandatory clinical trials registration) for all clinical trials that began enrollment on or after January 1, 2019.

Answer: Thank you for your comment. The data sharing plan has been added to the ClinicalTrials.gov public registry (NCT04884295). On the other hand, EudraCT does not publish phase 1 trials involving only adults, like the phase 1a trial (https://eudract.ema.europa.eu/docs/guidance/EudraCT%20FAQ_for%20publication.pdf).

* The trial registration or protocol lists secondary outcomes can you please present those results as part of this manuscript, or indicate why that is not possible? If this is not possible, can you please indicate when you plan to publish those results?

Answer: Thank you for your comment. All secondary outcomes of the phase 1a trial (pharmacokinetics) have been reported. All secondary outcomes of part 1 of the phase 1b trial (antiviral activity, respiratory parameters, COVID-19 related symptoms and clinical status) have been reported in the manuscript. To clarify the results linked to clinical status, the following sentence was added in the Results section: 'Clinical score evolved similarly between all dose levels.'.

* The sample size listed in the submitted manuscript and the trial registry differ. Please explain the discrepancy.

Answer: Thank you for your comment. In the phase 1b trial, 33 patients were screened as indicated in the manuscript and in NCT04884295 on ClinicalTrials.gov. 27 patients were enrolled, as indicated in the manuscript, in EudraCT 2020-005299-36 and in NCT04884295 on ClinicalTrials.gov.

* The main analysis should be intention to treat (ie, all individuals randomized are included in the analysis in the groups to which they were originally assigned. If the study included dropouts, specify whether their data are imputed and if so using what method. Please refer to as modified ITT).

Answer: Thank you for your comment. These changes have been implemented through the Methods and Results sections.

* The CONSORT flowchart should be figure 1, please revise.

Answer: Thank you for your comment. The CONSORT flowchart corresponds to Figure 1.

* In the flow diagram, please indicate the number of individuals in each group analyzed in the ITT analysis.

Answer: Thank you for your comment. The CONSORT flowchart in Figure 1 displays these numbers.

* Please present the safety data for the study including numbers of specific events and whether or not adverse events are thought to be related to treatment.

Answer: Thank you for your comment. The safety data of the Phase 1a healthy volunteer study were summarized in the manuscript, lines 347 – 357 and for the Phase 1b patient study, lines 358 – 390. For the healthy volunteer study, none of the AEs were assessed as related. Headache as the most frequently reported AE is mentioned and the AEs reported in 2 or more volunteers have now been added to the manuscript text. For the patient study, no specific AE was reported in more than one patient. Only the serious AEs and the grade 3 or higher AEs are mentioned specifically. To address the request, a table of treatment-emergent AEs which lists AEs by preferred term from the patient study is provided in the supplementary information. AEs that were assessed as related are identified with a footnote (these are already mentioned in the manuscript).

* In this test of superiority it is not possible to determine that the intervention and control are equivalent, just that the intervention is not superior to the control, please update the manuscript accordingly.

Answer: Thank you for your comment. We do not make any claim of superiority or non-inferiority in the manuscript.

* Causal language - In trials, there is usually a distinction in the language in terms of causal vs associational for primary and secondary trial outcomes. It would be beneficial to use associational language in the discussion and other sections for secondary outcomes.

Answer: Thank you for your comment. This has been implemented throughout the Results and Discussion section.

* Please report your abstract according to CONSORT for abstracts, following the PLOS Medicine abstract structure (Background, Methods and Findings, Conclusions) https://www.equator-network.org/reporting-guidelines/consort-abstracts/

Answer: Thank you for your comment. The abstract is already organised according to PLOS Medicine's format.

* Please include the clinical trial registry number in the abstract.

Answer: Thank you for your comment. This information has been added.

Per CONSORT, please note that only the primary outcome of the trial should be reported in your Abstract. Secondary outcomes should only be included in the Abstract if all secondary outcomes are fully reported. For trials that have many secondary outcomes, the Abstract should be limited to reporting the primary outcome.

Answer: Thank you for your comment. All secondary outcomes have been reported.

Comments from Reviewers:

Reviewer #1: The authors have thoroughly addressed my comments and the comments of the other reviewers.

Reviewer #3:
I thank the authors for responding to my comments. However, I am not sure if I agree with the authors' conclusion that the change in circulating variants cannot explain the shorter duration of symptoms in the 1000mg group. As the authors mentioned, all patients in the 250mg group had Delta, whereas 6/9 patients in 500mg group had Delta and in the 1000mg group, no one had Delta infection (4 had Omicron BA.1, 4 with recombinant and 1 with unknown variant). The 1000mg group also had a shorter time to symptom disappearance and shorter median time to hospital discharge. Since XVR011 has very poor neutralising activity against Omicron BA.1 (IC50 of 9816ng/ml) compared to Delta (IC50 of 13 ng/ml), it should have a much poorer protective effect against Omicron BA.1. How can the authors attribute the reduced time of symptoms and hospitalisations in the 1000mg group to XVR011 then, if XVR011 has such poor neutralising activity against Omicron BA.1? Wouldn't this be more likely due to a change in the infecting variant (Omicron BA.1 vs Delta)?

Answer: Thank you for your thoughtful comment. It is a fair point and we have adapted the Results section by adding the following confounding factors: 'SARS-CoV-2 variant evolving from Delta in the 250 mg cohort to the less pathogenic Omicron BA.1 in the 1000 mg cohort and evolving potency of XVR011 between the Delta and Omicron BA.1 variants'.

Reviewer #4: Thank you to the authors for addressing my previous comments.

The one remaining comment I had was on my previous comment 5 which was related to the line "Statistical analysis of dose-proportionality indicated that there was no significant deviation from dose proportionality for Cmax and AUC (p-values were all >0.05) (Tables 3, S5)." which is now on lines 403-404 of the clean revised version. I see now that I misread that as Figure S5 rather than Table S5 originally. I think the statement could still be more clearly linked to what's shown in table S5 - perhaps saying in the caption of S5 that the parameters taking value 1 would mean dose proportionality?

Other than that, I don't have any additional issues to raise.

Answer: Thank you for your useful comment. 'Parameters taking value 1 would mean dose proportionality' has been added to the legend of Table S5.

Any attachments provided with reviews can be seen via the following link:

In compliance with data protection regulations, you may request that we remove your personal registration details at any time. (Use the following URL: https://www.editorialmanager.com/pmedicine/login.asp?a=r). Please contact the publication office if you have any questions.
