# Supplementary material for: Characterization of the VHH-Fc construct rimteravimab in healthy adults and patients hospitalized for mild-to-moderate COVID-19: Two Phase 1 randomized clinical trials
Source: PLoS Med. 2026 May 29;23(5):e1004609. doi: 10.1371/journal.pmed.1004609 (PMC13221044; doi:10.1371/journal.pmed.1004609)
Supplement: Attachment — Submitted filename: Answer_to_Reviewers_Comments.docx [file pmed.1004609.s008.docx]

Dear Dr. Farrell,

Many thanks for your thoughtful suggestions to improve the quality of our manuscript. They have been integrated directly in the TrackChange version of the Maint Text and Supplementary Information files.

Sincerely,

Florence Herschke, for all co-authors.

Dear Dr. Herschke,

Thank you very much for re-submitting your manuscript "Characterization of VHH-Fc construct XVR011 (rimteravimab) in healthy adults and patients hospitalized for mild-to-moderate COVID-19: two Phase 1 randomized clinical trials." (PMEDICINE-D-25-00069R3) for review by PLOS Medicine.


The remaining issues that need to be addressed are in the document attached to this email. Please note that in addition to the requested changes to the manuscript, we require that you revise the data availability statement to transparently state all restrictions to data access and the process for requesting access.

Please submit a clean version of the paper as the main article file. A version with changes marked must also be uploaded as a marked up manuscript file.


We expect to receive your revised manuscript within 4 days. Please email us (plosmedicine@plos.org) if you have any questions or concerns.

We ask every co-author listed on the manuscript to fill in a contributing author statement. If any of the co-authors have not filled in the statement, we will remind them to do so when the paper is revised. If all statements are not completed in a timely fashion this could hold up the re-review process. Should there be a problem getting one of your co-authors to fill in a statement we will be in contact. YOU MUST NOT ADD OR REMOVE AUTHORS UNLESS YOU HAVE ALERTED THE EDITOR HANDLING THE MANUSCRIPT TO THE CHANGE AND THEY SPECIFICALLY HAVE AGREED TO IT.

Please ensure that the paper adheres to the PLOS Data Availability Policy (see [http://journals.plos.org/plosmedicine/s/data-availability](https://deref-gmx.com/mail/client/e54hvxDjsIc/dereferrer/?redirectUrl=http%3A%2F%2Fjournals.plos.org%2Fplosmedicine%2Fs%2Fdata-availability)), which requires that all data underlying the study's findings be provided in a repository or as Supporting Information. For data residing with a third party, authors are required to provide instructions with contact information for obtaining the data. PLOS journals do not allow statements supported by "data not shown" or "unpublished results." For such statements, authors must provide supporting data or cite public sources that include it.


Please note, when your manuscript is accepted, an uncorrected proof of your manuscript will be published online ahead of the final version, unless you've already opted out via the online submission form. If, for any reason, you do not want an earlier version of your manuscript published online or are unsure if you have already indicated as such, please let the journal staff know immediately at plosmedicine@plos.org.

If you have any questions in the meantime, please contact me or the journal staff on plosmedicine@plos.org.  

We look forward to receiving the revised manuscript by Apr 23 2026 11:59PM.   

Sincerely,

Alison Farrell, Ph.D.
Senior Editor 
PLOS Medicine
plosmedicine.org

__________________________________________________
In compliance with data protection regulations, you may request that we remove your personal registration details at any time. (Use the following URL: [https://www.editorialmanager.com/pmedicine/login.asp?a=r](https://deref-gmx.com/mail/client/-Hki3VQfkAs/dereferrer/?redirectUrl=https%3A%2F%2Fwww.editorialmanager.com%2Fpmedicine%2Flogin.asp%3Fa%3Dr)). Please contact the publication office if you have any questions.
